# Sparteine-Free, Highly Stereoselective Construction of Complex Allylic Alcohols Using 1,2-Metallate Rearrangements

**DOI:** 10.1021/jacsau.3c00114

**Published:** 2023-05-22

**Authors:** Yannick Linne, Maike Birkner, Jan Flormann, Daniel Lücke, Jörg August Becker, Markus Kalesse

**Affiliations:** † Institute of Organic Chemistry, Gottfried Wilhelm Leibniz Universität Hannover, Schneiderberg 1b, 30167 Hannover, Germany; ‡ Institute of Physical Chemistry and Electrochemistry, Gottfried Wilhelm Leibniz Universität Hannover, Callinstraße 3a, 30167 Hannover, Germany; § Centre of Biomolecular Drug Research (BMWZ), Gottfried Wilhelm Leibniz Universität Hannover, Schneiderberg 38, 30167 Hannover, Germany

**Keywords:** polyketides, Hoppe−Matteson−Aggarwal rearrangement, Nozaki−Hiyama−Takai−Kishi reaction, chiral anions, conformational analysis

## Abstract

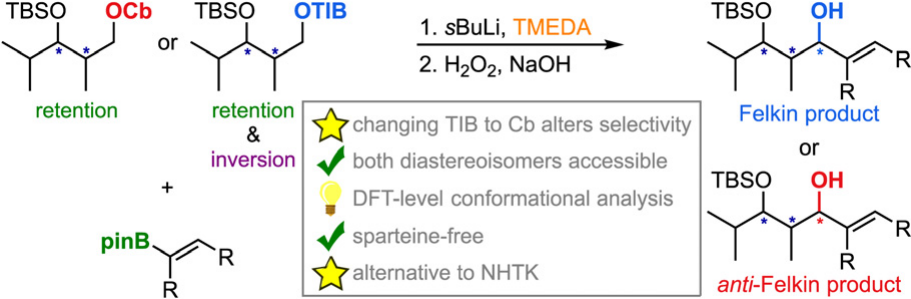

Stereotriads bearing allylic alcohols are privileged structures in natural products, and new methods accessing these in a stereoselective fashion are highly sought after. Toward this goal, we found that the use of chiral polyketide fragments allows for performing the Hoppe–Matteson–Aggarwal rearrangement in the absence of sparteine with high yields and diastereoselectivities, rendering this protocol a highly valuable alternative to the Nozaki–Hiyama–Takai–Kishi reaction. The switch of directing groups in most cases resulted in the reversed stereochemical outcome, which could be explained by conformational analysis on density functional theory level and a Felkin-like model.

## Introduction

**Scheme 1:**
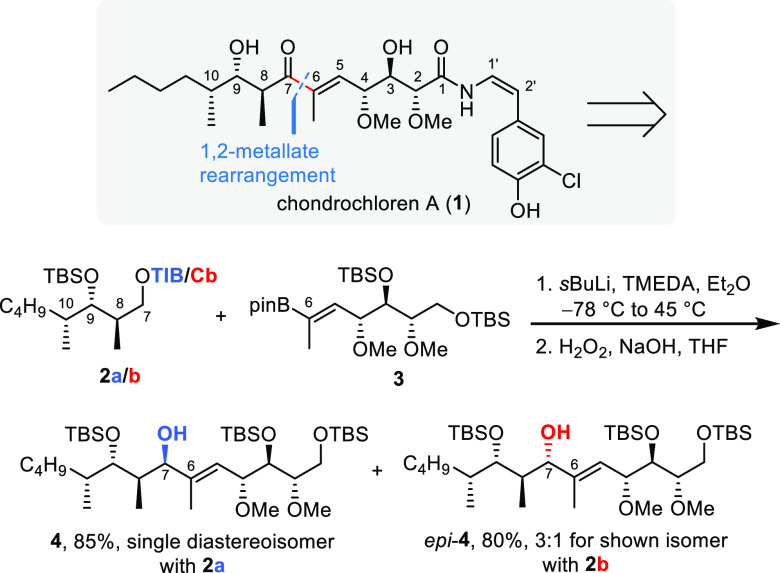
1,2-Metallate Rearrangement in the Course of Our Chondrochloren A (**1**) Synthesis

The synthesis of polyketides relies to a large extent on a distinct set of C–C bond-forming transformation, of which aldol reactions are certainly the most prominent and useful ones as they are able to control up to two new chiral centers during the C–C bond formation. Additions of metalorganic species to aldehydes and ketones are another highly important class of transformations in polyketide chemistry. Amongst those, the Nozaki–Hiyama–Takai–Kishi (NHTK)^1,2^ reaction outperforms organolithium and organomagnesium reagents due to its preference for reacting with aldehydes, unfolding a remarkable functional group tolerance toward ketones, esters, amides, nitriles, acetals, and other functional groups. However, in the course of our chondrochloren A (**1**) synthesis,^3^ we were not able to assemble the C5–C14 motif entirely by employing aldol reactions or organometallic reactions. On the search for alternatives and inspired by the seminal work of Aggarwal,^4−6^ we turned our attention to 1,2-metallate rearrangements using vinyl boronic ester **3** and the N,N-diisopropyl carbamoyl (Cb)- or 2,4,6-triisopropylbenzoyl (TIB)-derivatized polyketide fragment **2**. This setup mimics the NHTK reaction albeit with opposite polarities. In contrast to the NHTK reaction, here the vinyl reagent acts as the electrophile and the lithiated, masked alcohol (Hoppe anion) as the nucleophile. We gratifyingly observed an excellent yield (85%) and selectivity (single diastereoisomer) when using the TIB ester of fragment **2** (Scheme 1). Remarkably, using both enantiomers of sparteine provided significantly lower yields and selectivities compared to conditions employing N,N,N′,N′-tetramethylethylenediamine (TMEDA). Even more surprising was the observation that the switch to the Cb group favored the formation of the opposite diastereoisomer.^3^ Although this was inconsequential for our synthesis as the secondary alcohol was oxidized later on, it served as the starting point to investigate the directing effects in more detail and to evaluate the potential of this extension of the 1,2-metallate rearrangement as a general synthetic strategy. The aim of this paper is twofold: on the one hand, we want to present this extension of the 1,2-metallate rearrangement, which was developed by Aggarwal and based on the work of Matteson and Hoppe. It can be employed as an alternative to the NHTK reaction, and we want to point out that good selectivities and a change in diastereoselectivity can be achieved by employing either the TIB or the Cb group in the absence of sparteine. On the other hand, we are presenting advanced polyketidal structures to outline the potential of this methodology for synthetic applications.

**Scheme 2:**
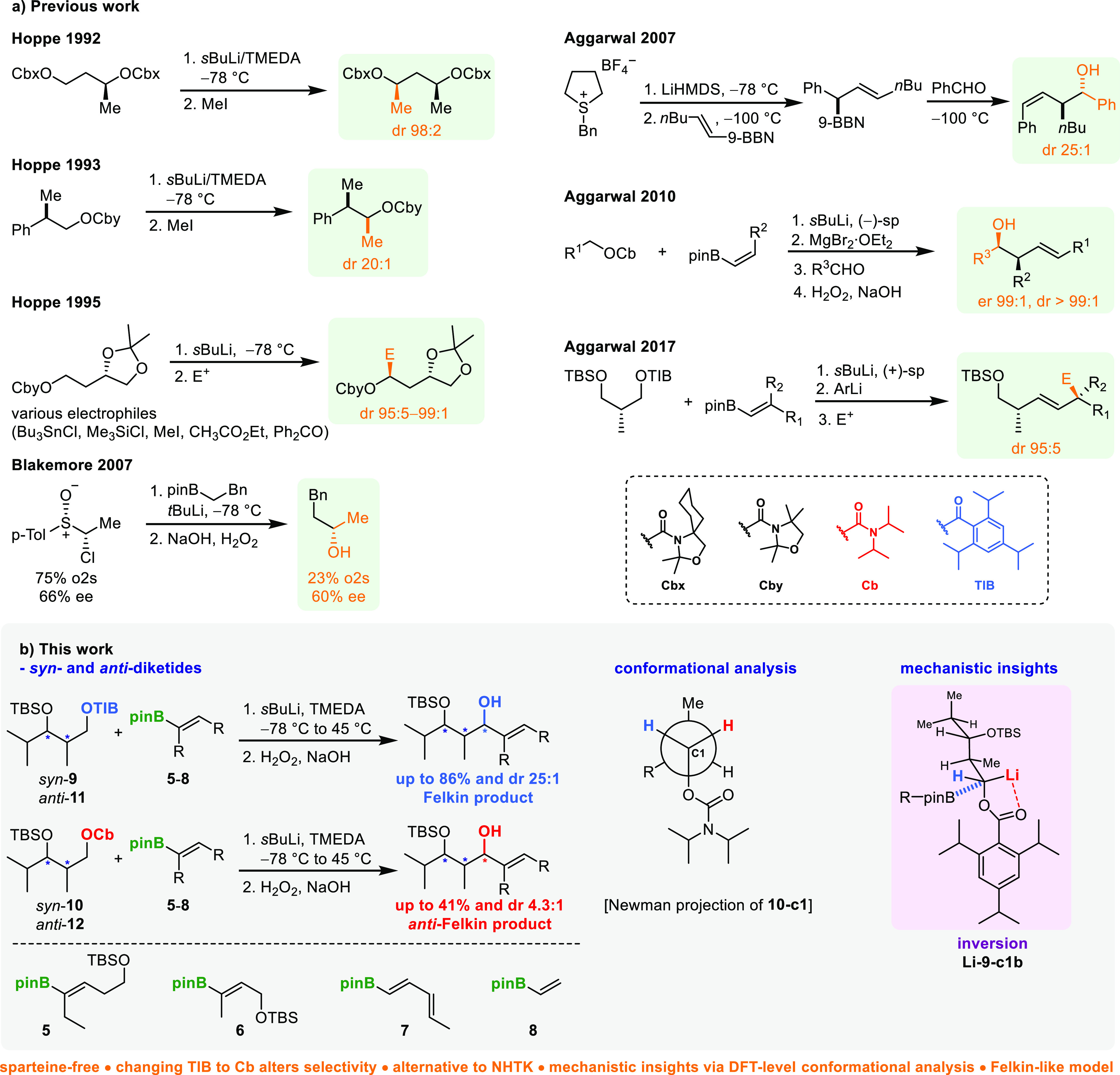
(a) Previous Work on Substrate-Induced Diastereoselective Alkylations, Sparteine-Free 1,2-Metallate Rearrangements and 1,2-Metallate Rearrangements Using Vinyl Boronic Esters; (b) Substrate- and Reagent-Controlled Synthesis of Stereotriads

Already in the early 1970s, Hoppe and co-workers reported substrate-induced diastereoselective alkylations of their carbamate-derived anions, which were later used by Aggarwal (and herein). In 1992, Hoppe published the diastereoselective methylation of double Cbx-protected diols, generating methyl-branched chains in a 1,3-distance (Scheme 2a).^7^ They proposed that the observed selectivity arises from chelation effects of both (3,3-dimethyl-1-oxa-4-azaspiro[4.5]decan-4-yl)-carbonyl (Cbx) groups. Even without the second carbamate moiety, they observed diastereoselective alkylation controlled by the chiral center in β-position (1993).^8^ This was followed in 1995 by an extension of their 1992 observation, namely, the use of a chiral five-ring acetonide, which also provided stereocontrol with various electrophiles (Scheme 2a).^9,10^ Blakemore was even the first to describe a sparteine-free “lithiation–borylation” reaction around 2007. However, his α-sulfinyl chlorides require a two-step sequence of enantioselective Jackson–Ellman–Bolm oxidation and Yamakawa chlorination. Using the methyl branch common in polyketides, only 66% ee were obtained in the preparation of the *α*-sulfinyl chlorides. Homologation with benzyl boronic ester also provided only a moderate yield of 23%, indicating the need for further sparteine-free lithiation–borylation methods.^11−19^

In 2007, Aggarwal and co-workers also showed the utility of chiral sulfonium benzylides in 1,2-metallate rearrangements with vinyl 9-borabicyclo-[3.3.1]nonanes (9-BBNs) (Scheme 2a).^20^ The obtained chiral allylic boranes were further employed in nucleophilic additions to aldehydes, generating homoallylic alcohols in high selectivities. A further enhancement of this method for the synthesis of chiral 1,2,4-substitued homoallylic alcohols was achieved by Aggarwal in 2010 via the addition of Hoppe anions to vinyl boronic esters and subsequent addition to aldehydes.^21^ Later, in 2017, the same group used Hoppe anions obtained from TIB esters with sparteine and vinyl boronic esters.^22^ The so-obtained allyl boronate was then activated by the addition of aryllithiums to increase its nucleophilicity, allowing stereospecific reactions with a wide variety of electrophiles.

Here, we report the addition of Hoppe anions to vinyl boronic esters, albeit with an oxidative work-up instead of subsequent C–C bond formation (Scheme 2b). The aim of this work is to show that it is possible to obtain stereoselectivity without the need for sparteine using polyketide fragments having at least α- and β-substituents. Remarkably, the yields and selectivities often increase when sparteine is omitted. Furthermore, we demonstrate that alternating between the Cb and TIB groups can control whether the formal Felkin or *anti*-Felkin product is obtained, thus allowing for stereodivergent strategies in total syntheses.

## Results and Discussion

**Scheme 3:**
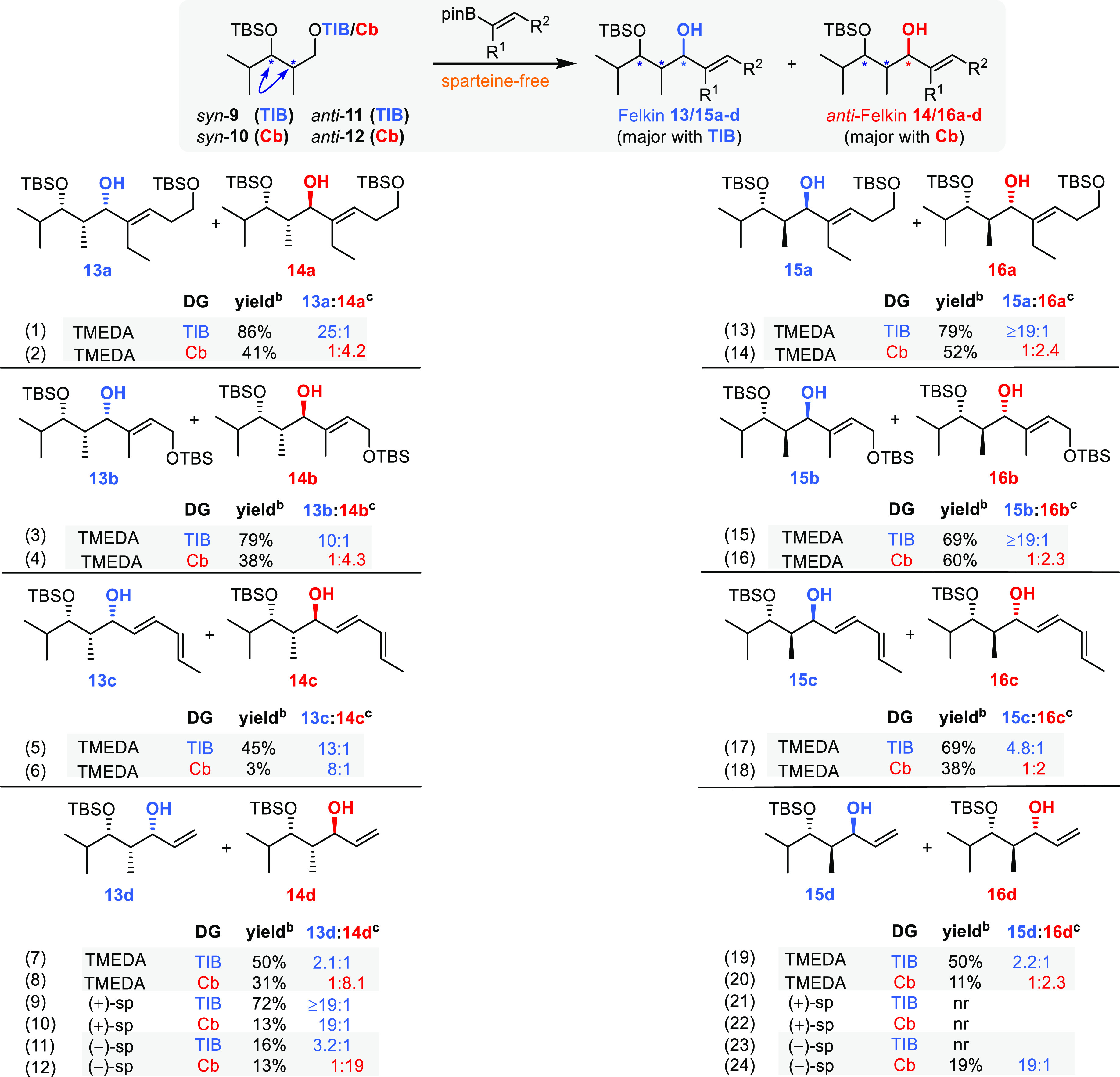
Substrate- and Reagent-Induced 1,2-Metallate Rearrangement with *syn-* and *anti*-Configured Diketides Blue indicates the formal Felkin product and red the formal *anti*-Felkin product. Yields are isolated products. General conditions: vinyl boronic ester (1.0 equiv, 0.29 mmol), TIB/Cb diketide (1.5 equiv, 0.44 mmol); for detailed information, see the SI. dr determined by ^1^H NMR. Absolute stereochemistry of selected examples determined via Mosher analysis (see the SI) .

We started our investigation with *syn-* and *anti*-diketides (Scheme 3) derived from isobutyraldehyde as its isopropyl branch resembles polyketidal extensions and therefore provides an admissible illustration for complex natural product syntheses. Throughout this work, we used two branched (**5**, **6**) and two unbranched vinyl boronic esters (**7**, **8**) (Scheme 2b). The branched vinyl boronic esters resemble the situation given in polyketidal syntheses more closely, whereas the unbranched ones represent a more conservative assessment of the lowest yields and selectivities one could expect.

### *syn*-Configured Diketides

Using the branched vinyl boronic esters **5** and **6** and the anion derived from TIB ester **9** generated Felkin products **13a**,**b** in very good yields and selectivities (25:1, 10:1, entries 1 and 3, Scheme 3). Remarkably, using the Cb group for coordinating the anion led to the opposite diastereoisomers **14a**,**b**, albeit with lower yields and selectivities. When using dienyl boronic ester **7**, the Felkin product was observed in an acceptable yield and good selectivity using TIB ester **9** (entry 5, 45%, 13:1). The same product was observed in 3% yield when carbamate **10** was employed (entry 6), and it should be pointed out that this is a rare example where we did not observe the *anti*-Felkin product with the Cb directing group. The unsubstituted vinyl boronic ester **8** gave only modest yields and selectivities (50%, 2.1:1, Scheme 3, entry 7) with TIB-derivatized diketide **9**. Here, the Cb group produced in good selectivity (8.1:1) the *anti*-Felkin product **14d** but only in 31% yield (entry 8). The use of (+)-sparteine in combination with TIB ester **9** restored the good selectivities and yields observed for the branched vinyl boronic esters. Remarkably, the use of (−)-sparteine should by theory in this case lead to deprotonation of the *anti*-Felkin proton. However, the Felkin product was observed here as well, albeit in low yields and selectivities, reflecting the mismatched situation and providing a first indication that inversion processes during borylation might be involved (entry 11). On the other hand, the *anti*-Felkin selectivity of the Cb analogue was improved to 19:1 and which is in accordance with the anticipated facial deprotonation of (−)-sparteine and reflects the matched situation (entry 12). This also means that in the case of the Cb directing group, both sparteine enantiomers gave their respective diastereoisomers in excellent selectivities, indicating the preference for a retention process in the borylation step.

### *anti*-Configured Diketides

Reactions employing the corresponding *anti*-configured diketides parallel the results obtained for *syn*-diketides. The boronic esters **5** and **6** and the anion derived from TIB ester **11** provided the formal Felkin products **15a**,**b** in very good yields (79 and 69%) and excellent selectivities (19:1, Scheme 3, entries 13 and 15). The opposite diastereoisomers **16a**,**b** were here as well observed when Cb was used instead of TIB, albeit in lower yields (52 and 60%) and selectivities (2.4:1 and 2.3:1, entries 14 and 16). When using dienyl boronic ester **7** in combination with TIB ester **11**, the Felkin product was observed in good yield and selectivity (69%, 4.8:1, entry 17). Using carbamate 12 and vinyl boronic ester 8 led to the anti-Felkin product in only 11% (2.3:1, entry 20, Scheme 3). In contrast to the *syn*-diketides, only the combination of (−)-sparteine with Cb analogue **12** led to the formation of Felkin product **15d**, however in only 19% yield (entry 24). This difference between the *syn*- and *anti*-diketides indicates a conformational origin of selectivity.

### Stannane Formation

**Scheme 4:**
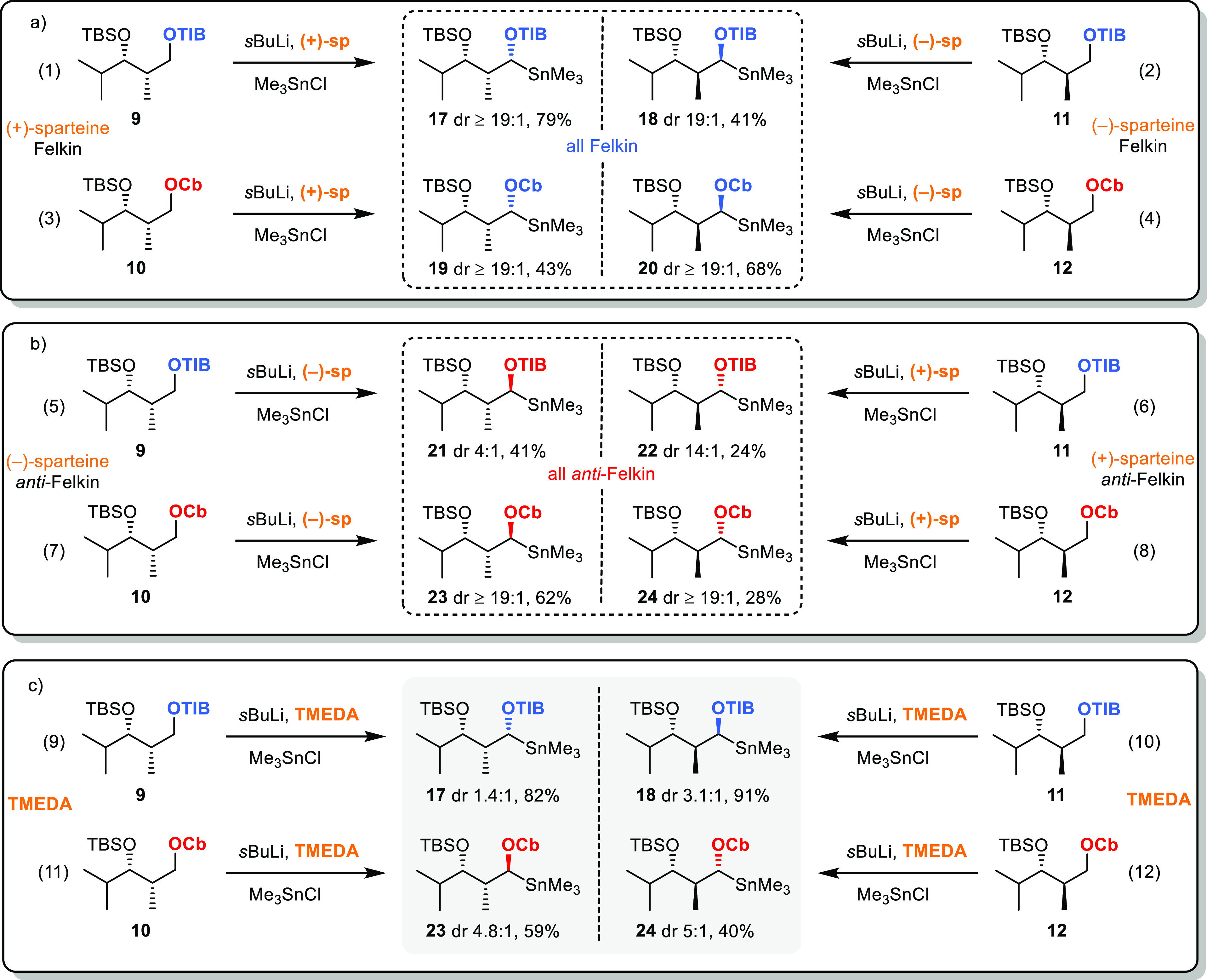
(a–c) Stannane Trapping of *syn*- and *anti*-Configured Diketides Using Reagent and Substrate Induction

At this stage, we initiated additional experiments for disclosing the origin of the observed selectivities. Specifically, the chirality of the generated lithium species in dependence of the directing groups (TIB vs Cb) and concomitant retention vs inversion processes in the subsequent borylations were considered as pivotal processes that control the stereochemistry of the rearranged products. For this, the anions generated from the TIB- and Cb-protected diketides **9**, **10**, **11**, and **12** were trapped as their trimethylstannanes (Scheme 4, entries 1–12).

The diastereomeric ratio of the generated stannanes should reflect the ratio of the formed anions since these transmetallation processes usually proceed under complete retention.^23^ In general, non-mesomeric stabilized Hoppe anions prefer to react with electrophilic reagents (e.g., TMSCl, CO_2_, Me_3_SnCl, MeI, or boronic esters) with retention.^4,24−26^ The first set of transformation was made with (+)-sparteine for the *syn*-diketides and (−)-sparteine for the *anti*-diketides as both combinations should lead to the respective Felkin stannanes (Scheme 4a). In all four cases, the Felkin stannanes sp-**17–20** were obtained in yields ranging from 41 to 79% and selectivities of at least 19:1. These results demonstrated that in all these cases, sparteine controls the stereochemical outcome of the deprotonation process and is capable of overwriting the inherent substrate selectivities in favor of the Felkin product. The generation of the *anti*-Felkin stannanes sp-**21–24**, on the other hand, required in each case the opposite enantiomer of sparteine. For Cb analogues **10** and **12**, substrate induction observed in the 1,2-metallate rearrangement was enhanced in a matched situation to the *anti*-Felkin product (Scheme 4b, entries 7 and 8). TIB esters **9** and **11** on the contrary provided the corresponding stannanes **21** and **22** in selectivities of only 4:1 and 14:1 (entry 5 and 6), respectively. This significant reduction compared to the generally observed stereoinduction of sparteine (95:5–98:2)^27−30^ highlighted the mismatched situation for TIB esters **9** and **11**. The observations on the matched and mismatched situations clearly show that the TIB group favors the formation of the Felkin product, while the inherent selectivity of the Cb group can be overruled by sparteine.

The stannane formation in the absence of the sparteines should now reveal the inherent selectivity of the deprotonation process. Unexpectedly, we observed the formation of the TIB-derived Felkin stannanes TMEDA-**17** and **18** in poor to very poor selectivities (3:1 and 1.4:1, Scheme 4c, entries 9 and 10). However, these results do not parallel the excellent yields (69–86%) and selectivities (10:1–25:1) obtained with the branched vinyl boronic esters **5** and **6**. This indicated that the stereoselectivities are not controlled by the lithiation step but by the ate-complex formation of the vinyl boronic ester. In this case, the TIB-derived lithiates would not react specifically under retention or inversion with the vinyl boronic esters, but both processes would take place in a complementary fashion during the borylation. The preference for the Felkin hemisphere clearly depends on the steric demand of the vinyl boronic ester used (Scheme 3, entries 1, 3, 13, 15 vs 5, 7, 17, 19).

The situation is different for both Cb analogues **10** and **12**. In both cases, deprotonation and stannane trapping under sparteine-free conditions generated the *anti*-Felkin stannane in a ratio of approximately 5:1 (Scheme 4c, entries 11 and 12). In contrast to the abovementioned TIB esters, this ratio reflects the stereochemical outcome of the lithiation–borylation step for branched vinyl boronic esters **5** and **6** (Scheme 3, entries 2 and 4, dr 4.2:1 and 4.3:1 for *syn*-carbamates) but also for simple vinyl boronic ester **8** (Scheme 3, entry 8, dr 8:1 for *syn*-carbamates). In any case, the carbamate-derived anions seem to react under retention of configuration in the borylation step. The same rationale holds true for the *anti*-carbamates. However, the selectivities observed in the entire rearrangement process including deprotonation and borylation are generally lower for the *anti*-Felkin product and might reflect a more pronounced steric hindrance of the *anti*-Felkin hemisphere.

To confirm the stated hypotheses, all four TMEDA-derived stannanes (**17**, **18**, **23**, and **24**) were subjected to borylation and concomitant metallate rearrangement after being treated with *n*-butyllithium for Sn–Li exchange and subsequent addition of TMEDA.

### Carbamates

**Scheme 5:**
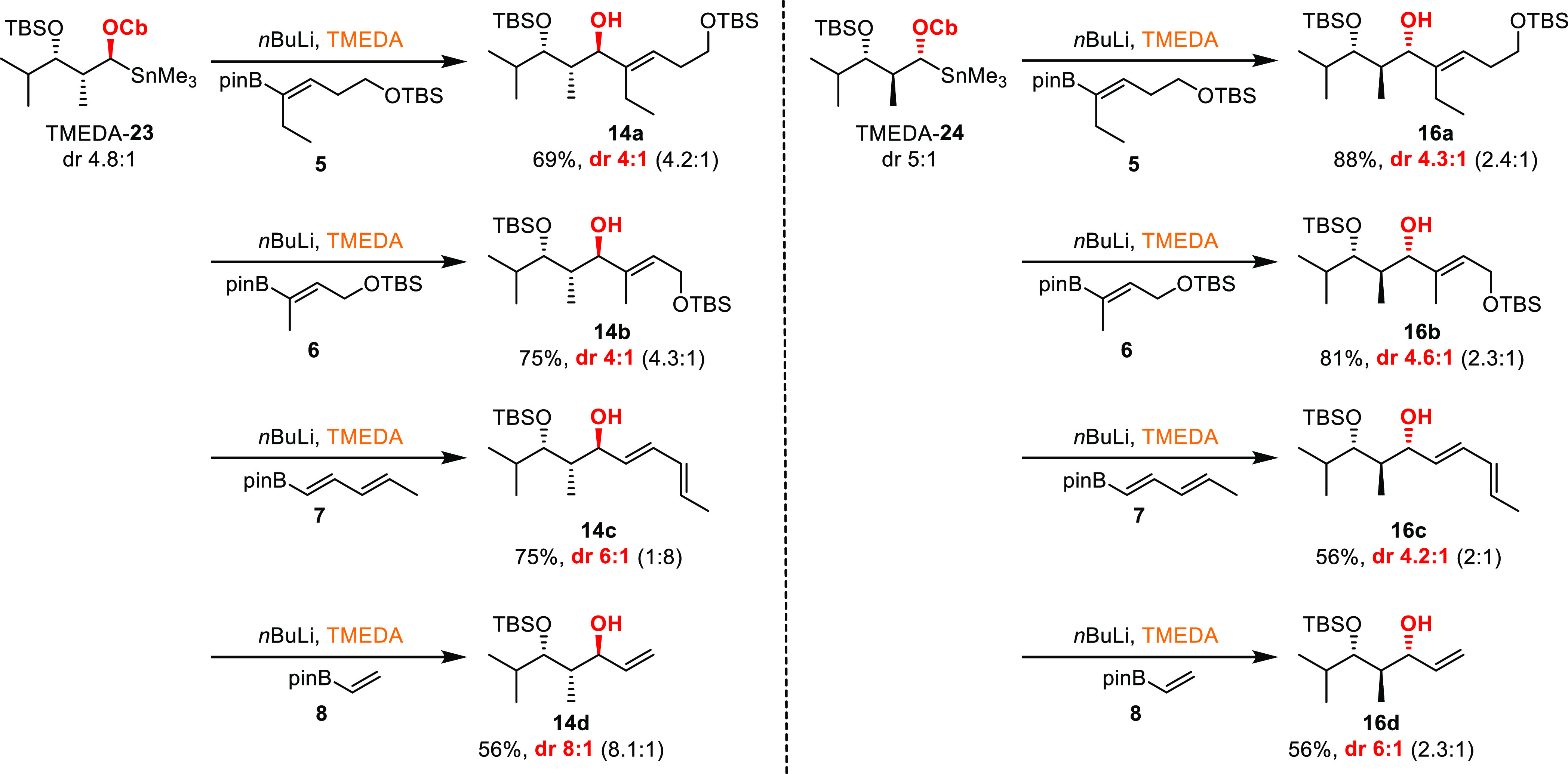
1,2-Metallate Rearrangements on the TMEDA-Derived Stannanes of the *syn*- and *anti*-Configured Cb Diketides The ratios in parentheses are the selectivities observed in the one-pot process (Scheme 3).

The re-liberated anion from stannane TMEDA-**23** (dr 4.8:1) was treated with the four vinyl boronic esters **5** to **8** (Scheme 5), and the observed selectivities were almost identical to the ones observed in the one-pot process (Scheme 3, entries 2, 4, 6, and 8). Only dienyl boronic ester **7** provided an exception, enabling the expected *anti*-Felkin product **14c** to be formed in this case. These results further support the rationale that the configuration obtained from the primary deprotonation process is retained during the borylation step, which, after stereospecific 1,2-metallate rearrangement (and additional oxidation), ultimately leads to the isolated allylic alcohols. The same tendency can be observed for the *anti*-carbamate. However, the two-step process via stannane TMEDA-**24** (dr 5:1) provides slightly higher selectivities. Nevertheless, also here borylation takes place mainly under retention of configuration, supporting our hypothesis that the Cb directing group favors ate-complex formation under retention of configuration.

### TIB Esters

The corresponding liberation and boronate trapping of *syn*-and *anti*-configured TIB-derived stannanes TMEDA-**17** (dr 1.4:1) and TMEDA-**18** (dr 3.1:1, Scheme 6) provided similar selectivities as observed in the one-pot process. Again, in the case of the *syn*-diketide, dienyl boronic ester **7** provided an exception with a lower selectivity compared to the one-pot process. Nevertheless, in all cases, the poor selectivity of the stannanes (1.4 and 3.1:1) was transformed to 19:1 for the sterically demanding vinyl boronic esters and 3:1 for the unbranched substrate. Accordingly, these results support the hypothesis that when the TIB directing group is used, inversion processes must occur during ate-complex formation in addition to retention.

**Scheme 6:**
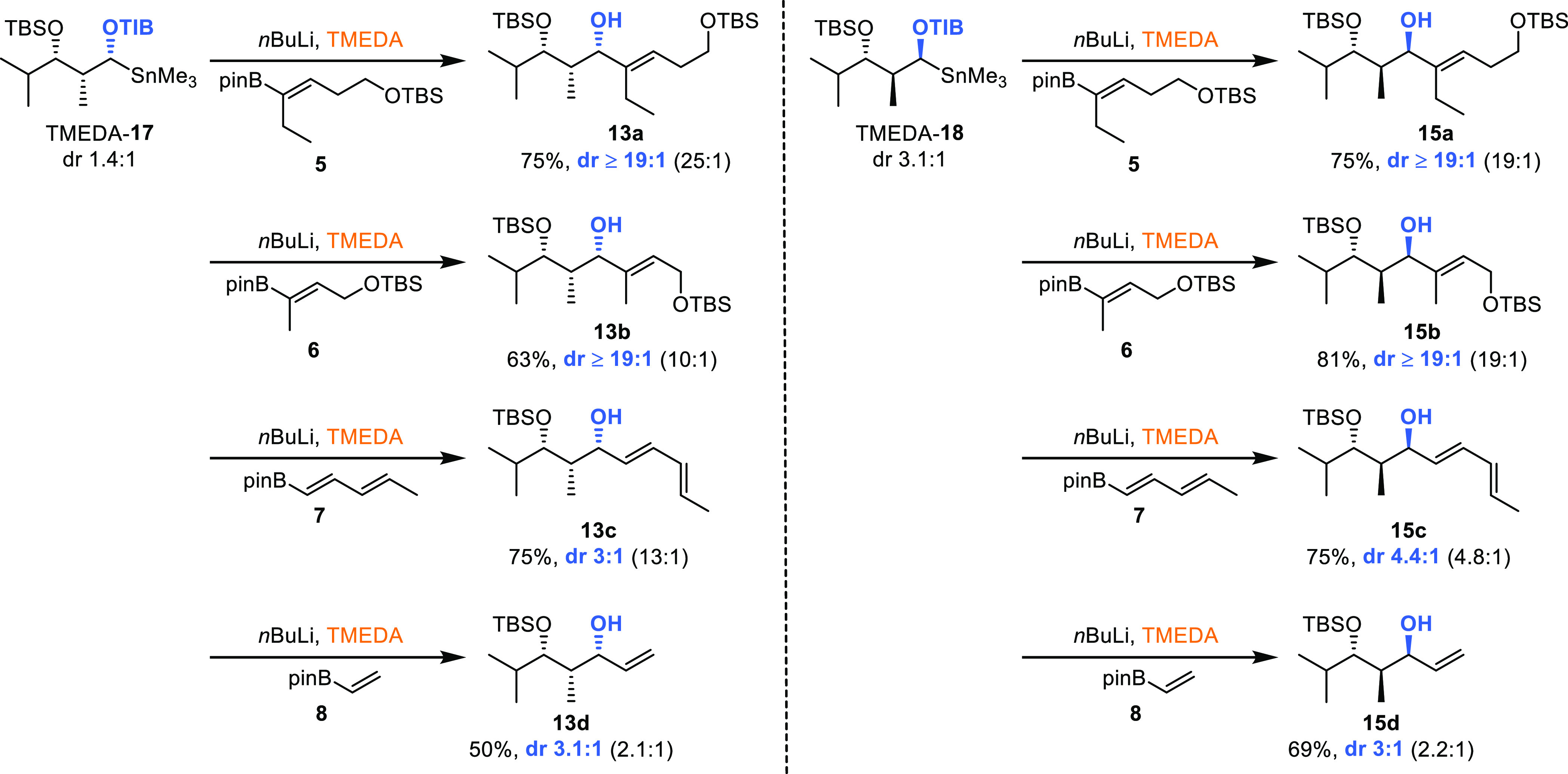
1,2-Metallate Rearrangements on the TMEDA-Derived Stannanes of the *syn*- and *anti*-Configured TIB Diketides The ratios in parentheses are the selectivities observed in the one-pot process (Scheme 3).

In order to provide a basis for discussions on the origin of the observed selectivities, we initiated a quantum chemical conformation search for the four diketides (**9**, **10**, **11**, and **12**). In a preliminary step, conformations were generated for all diketides with Grimme’s CREST module based on the semi-empirical tight binding method xTB.^31,32^ This resulted in up to 5700 different conformers for each diketide with an energy difference of up to 24 kJ/mol between the conformers. A subset of these geometries (e.g., the first 150 and then every 10th) were subsequently used as starting points for density functional theory (DFT) optimizations on the B3LYP/6-31G(d,p) level of theory in Gaussian 16.^33,34^ Corrections for dispersion and solvent interaction were included.^35−37^ Conformers with significant Boltzmann factors (>0.01) at −78 °C and thus an energy difference of less than 8 kJ/mol compared to the conformer with the lowest energy were then used for structural discussions.^38^

**Scheme 7:**
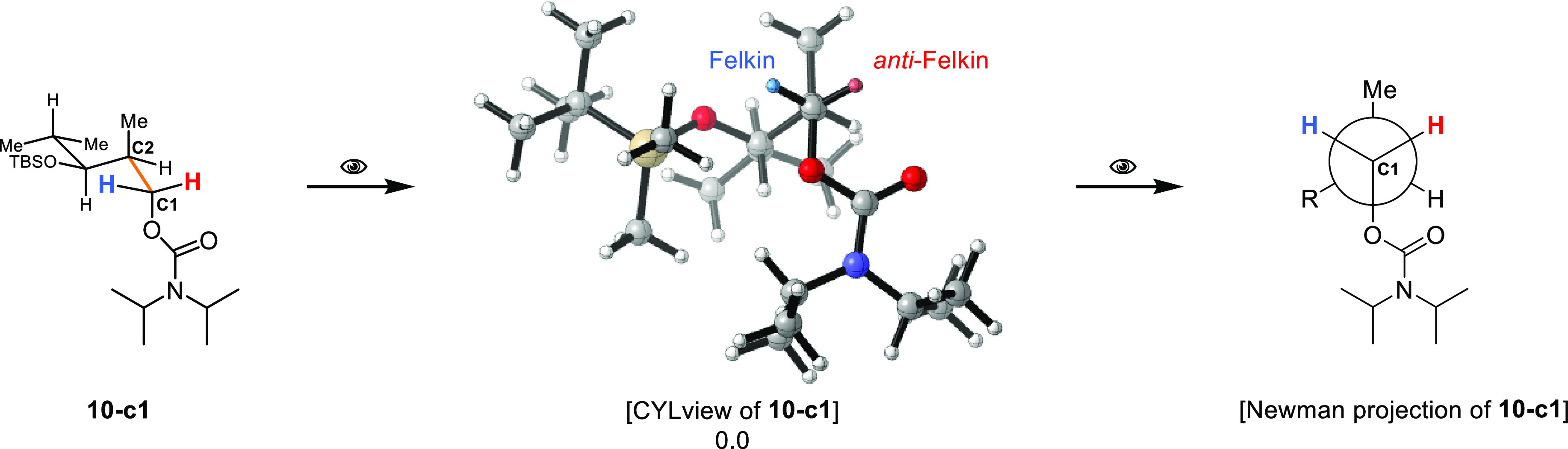
Illustration for the Representation of the Preferred Conformations Found Using *syn*-Carbamate **10** as an Example R = TBS-protected isobutanol residue.

For clarity, the preferred conformations found are presented in a Newman projection-like plot. In Scheme 7, this is exemplified by conformation **10-c1** of *syn*-carbamate **10**. The Newman-like representation is always chosen along the C1–C2 bond so that the Felkin and *anti*-Felkin hemisphere can be clearly identified, and the orientation of the directing group is evident. This type of representation also allows the different structures and conformations to be quickly and easily compared with each other.

### Substrate-Induced Lithiation

**Scheme 8:**
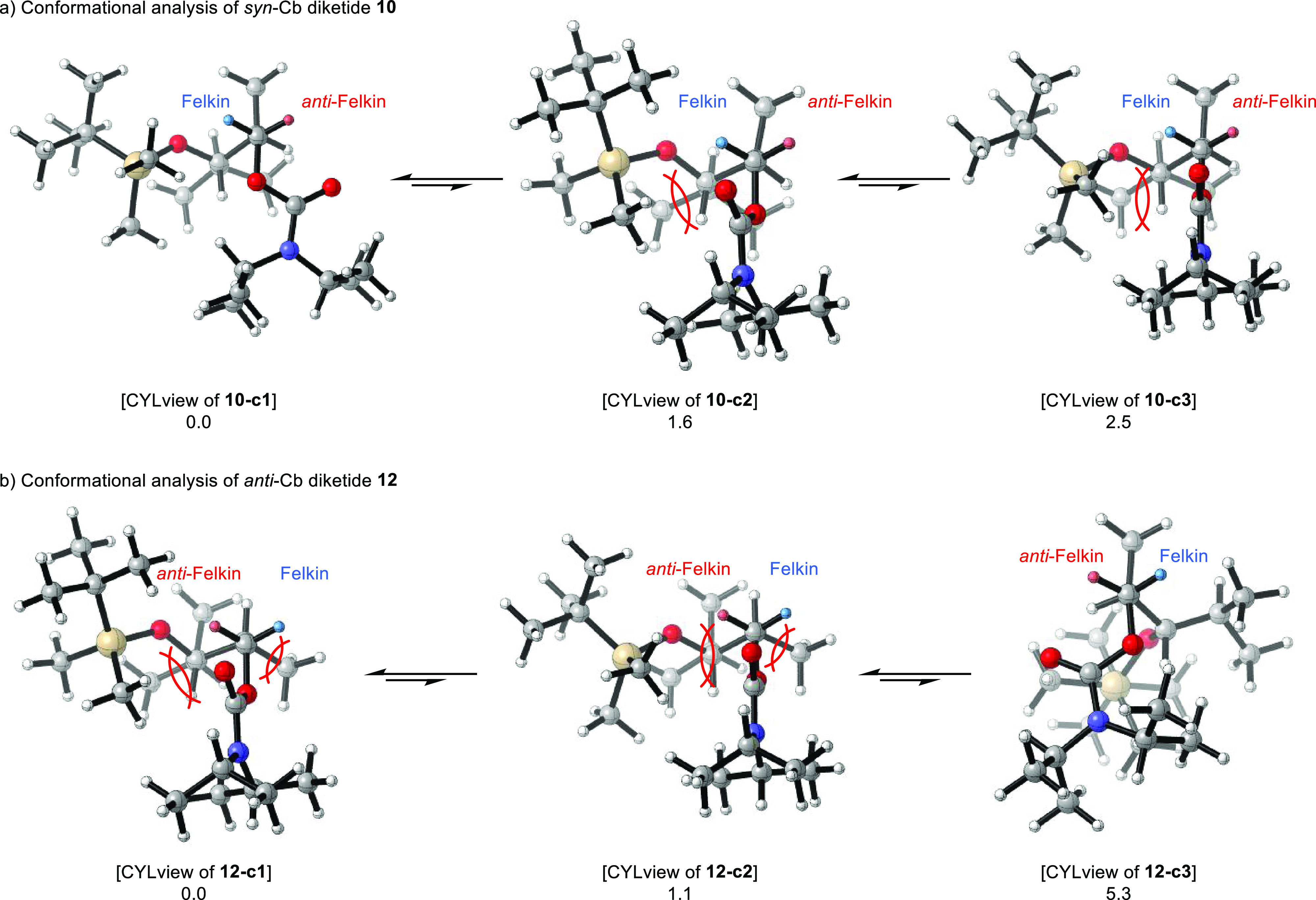
(a,b) Conformational Analysis of DFT-Optimized Cb Diketides **10** and **12** in the Course of the Lithiation Step Values below the structures are relative energies in kJ/mol.

For the carbamates, the observed *anti*-Felkin selectivity can be rationalized by the structures of the three lowest-energy conformers, respectively (Scheme 8). In the found global minimum **10-c1**, *syn-*carbamate **10** adopts a conformation in which the carbamate carbonyl oxygen is orientated toward the *anti*-Felkin proton (red). Since the concept of Hoppe’s anions implies that the carbonyl oxygen of the directing group directs the base to the proton to be deprotonated, its orientation explains the *anti*-Felkin selectivity in the lithiation step of *syn*-carbamate **10**. Taking this concept into account, the Felkin proton would be deprotonated in the energetically also favorable conformation **10-c2** (+1.6 kJ/mol). However, in this case, the Felkin hemisphere is sterically shielded by the TBS ether, making this deprotonation less favorable. In conformation **10-c3** (+2.5 kJ/mol), which is energetically slightly less favorable, the Cb group is placed like a bisector between the two hemispheres. In this conformation, however, the *anti*-Felkin hemisphere is sterically considerably less hindered. The remaining 15 conformers are structurally comparable to one of the three cases mentioned above except for slight rotations in the TBS-protected isobutanol residue. At the given temperature, the population of conformers similar to **10-c1** is 50%, to **10-c2** 30%, and to **10-c3** 20% (Boltzmann factors are given in the SI). The strongly respectively moderately preferred abstraction of the *anti*-Felkin proton in the first and third case together with the sterically hindered Felkin hemisphere in the second case explains the *anti*-Felkin selectivity in the lithiation step of *syn*-carbamate **10**.

The conformation of *anti*-configured Cb diketide **12** in the found global minimum **12-c1** (60% population) shows only a slight orientation of the directing group’s carbonyl oxygen atom toward the *anti*-Felkin hemisphere, which here exhibits steric clashes with the large residue containing the TBS ether. The Felkin hemisphere, which is sterically less hindered in this case, also shows a medium-sized residue in terms of a methyl group in contrast to the *anti*-Felkin hemispheres in **10-c2** and **10-c3** (Me vs H). Conformation **12-c2** (+1.1 kJ/mol, 30%) placed the Cb group again as a bisector, although here both hemispheres reveal steric clashes with the *anti*-Felkin hemisphere being sterically more hindered (isobutyl residue vs Me). In this case, the Felkin anion would be formed preferentially based on a pure steric analysis, which would contradict our experimental findings in which the *anti*-Felkin anion is formed predominantly. Therefore, the conformation that is most relevant for the lithiation step is **12-c3** (+5.3 kJ/mol, 2%), the energetically considered third best choice. Here, the Cb group is again orientated into the *anti*-Felkin hemisphere, enabling the coordination of the base toward the *anti*-Felkin hemisphere. Furthermore, **12-c3** shows a strongly crowded Felkin hemisphere, which makes the abstraction of the Felkin proton (blue) unfavorable. The six remaining conformers with higher energies are either similar to **12-c3** or have low populations of 2% or less. In total, conformers similar to **12-c3** have a population of 5%, which together with the high accessibility by the base due to negligible steric hindrance in the *anti*-Felkin hemisphere explains the observed *anti*-Felkin selectivity of carbamate **12** in the lithiation step.

**Scheme 9:**
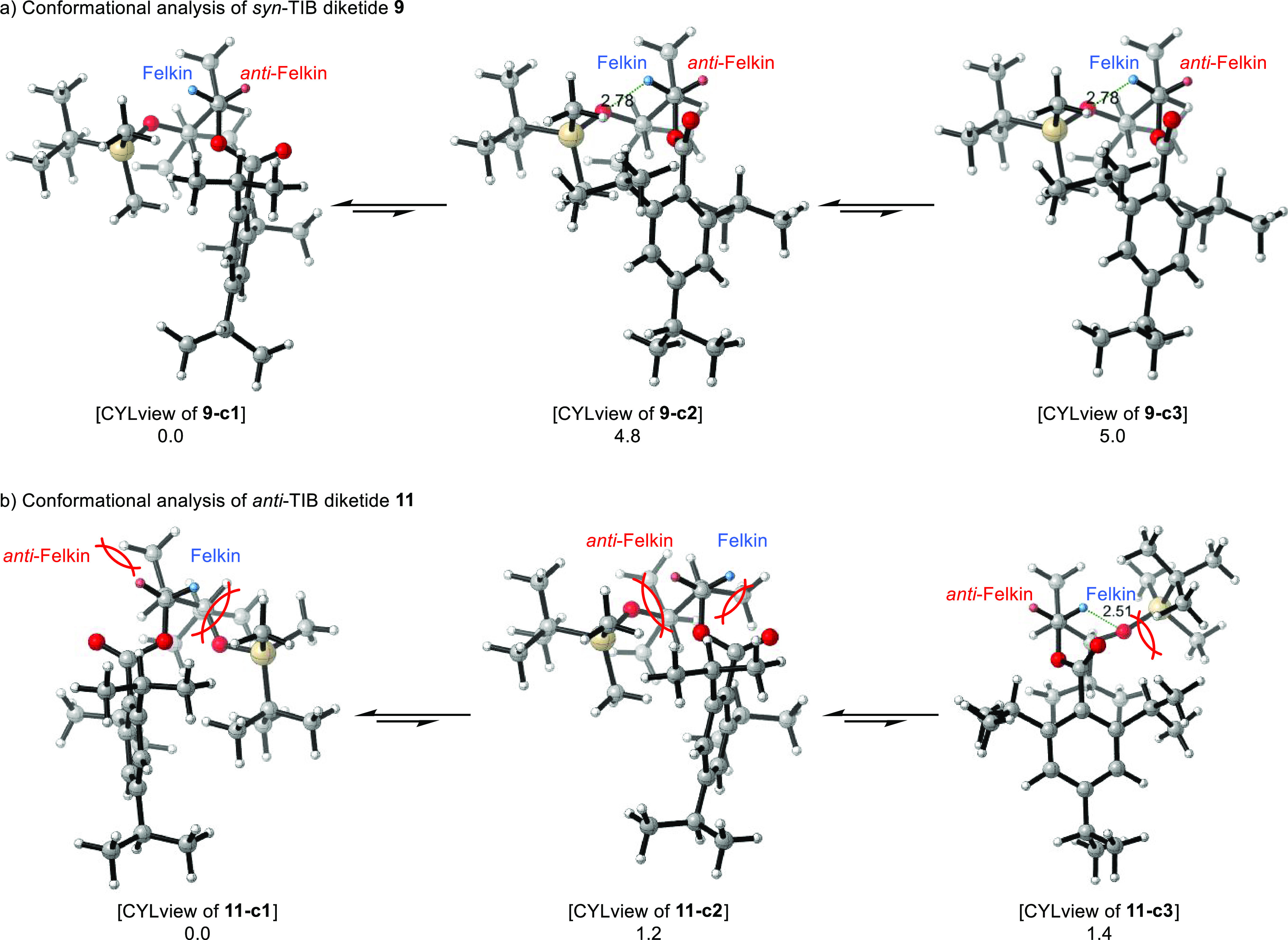
(a,b) Conformational Analysis of DFT-Optimized TIB Diketides **9** and **11** in the Course of the Lithiation Step Values below the structures are relative energies in kJ/mol.

Even though the situation for the TIB esters is more complex since retention as well as inversion can take place and the stannanes are obtained with low selectivities, the experimentally observed selectivities can be explained here as well on the basis of the low-energy conformers (Scheme 9). In the global minimum, *syn*-TIB ester **9** adopts conformation **9-c1** like the Cb derivatives with the carbonyl oxygen orientated toward the *anti*-Felkin proton. However, this would contradict our experimental findings in which the Felkin anion, respectively, stannane is formed in slight excess. Most structures above the global minimum **9-c1** show a similar conformation, leading to a summed population of 91.5% for these geometries. Exceptions are **9-c2** and **9-c3** at +4.8 and +5.0 kJ/mol, respectively, with a combined population of 8.5%, where both the Felkin and the *anti*-Felkin proton have a similar distance to the carbonyl oxygen atom (between 2.5 and 2.8 Å). Assuming that the reactivity would only depend on the population of the ground-state isomers, this should lead to a high selectivity for the *anti*-Felkin anion, contradicting the experimental results. However, the reactivity and thus the product distribution (anion/stannane) do not (only) depend on the ratio of the two ground-state isomers according to the extended Curtin–Hammett principle.^39−45^ Conformations **9-c2** and **9-c3** position the oxygen atom of the TBS ether in close proximity to the Felkin proton, rendering it more reactive as demonstrated by Knochel’s famous chemistry.^46−50^ Thus, the TBS ether could act as a directing group here and contribute to the slight Felkin selectivity.

In the case of *anti*-TIB ester **11**, the carbonyl oxygen atom in the global minimum **11-c1** (45%) is orientated toward the *anti*-Felkin hemisphere, which is hindered by the orientation of the methyl group at C2. The opposite is true for conformation **11-c2** (1.2 kJ/mol, 22%) obtained via a low rotational barrier, where the carbonyl oxygen atom and the methyl group occupy the Felkin hemisphere. Arguing only with the difference in the population of these conformers, one would suggest a moderate selectivity for the *anti*-Felkin anion. However, in conformer **11-c3** (1.4 kJ/mol) and all seven conformers with higher energies, the carbonyl oxygen atom is orientated toward the Felkin hemisphere, resulting in a population of 33% for these geometries. In some of these, the oxygen atom of the TBS ether is in close proximity to the Felkin proton, as in **9-c2**, and could act as a further directing group, analogous to Knochel’s chemistry.^46−50^ In the other ones, the Felkin hemisphere is not at all or only slightly sterically hindered. In both cases, the formation of the Felkin anion is preferred. Consideration of these aspects also explains the observed slight Felkin selectivity of the TIB diketides in the lithiation step.

### Substrate- and Reagent-Controlled Borylation

Since the actual structure of the anions as well as the mechanism of boron–lithium exchange is not precisely known, we base our considerations on retention and inversion processes during the borylation step on our experimental findings rather than on computational calculations.^4,27,51−54^

**Scheme 10:**
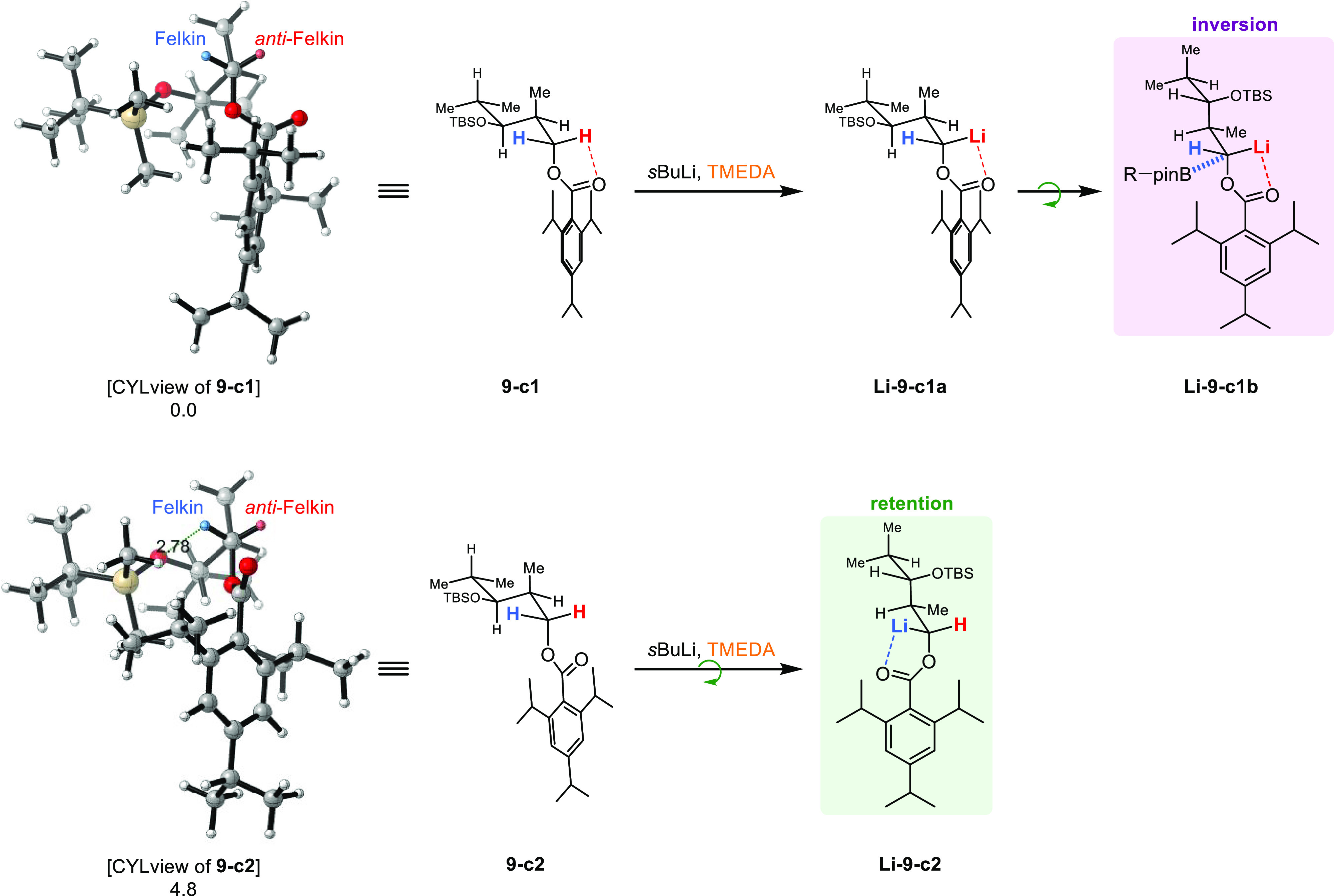
Rationalization for Inversion and Retention Processes during Borylation in the Case of *syn*-TIB Ester **9** Values below the structures are relative energies in kJ/mol.

Similar observations on inversion and retention processes, as observed by us with the TIB esters, were already reported by Hoppe and Schleyer, albeit on benzylic anions.^26,55^ Hoppe^26^ experimentally confirmed in 1990 Schleyer’s prediction on steric and electronic contributions controlling either of the two pathways.^55^ Hoppe states that “apparently the extent of the interaction of electrofugal and nucleofugal leaving groups as well as steric effects have a decisive influence thereby on the competing reaction paths.”^26^ The influential steric effect mentioned by Hoppe is also reflected in our work, with the branched vinyl boronic esters **5** and **6** giving excellent Felkin selectivities, whereas in the case of unbranched **7** and **8**, only moderate Felkin selectivities were observed. Under the assumption that the anions are formed from the conformations we determined and initially adopt a similar conformation to the non-lithiated derivatives, the occurrence of the inversion process can be explained via a small conformational change, which is presumably to some extent driven by the size of the directing group itself, thus reducing the steric hindrance in the hemisphere of the anion. In the case of *syn*-TIB ester **9** (Scheme 10), conformation **9-c1** would lead as described to the *anti*-Felkin anion with assumed conformation **Li-9-c1a**. Here, both hemispheres are sterically crowded, while the *anti*-Felkin hemisphere is shielded strongly by the 2,4,6-triisopropylphenyl (TIP) residue and slightly by one methyl group of the isobutanol residue, the Felkin hemisphere is occupied by the rest of the isobutanol substituent. A small conformational change from **Li-9-c1a** to **Li-9-c1b** would lead to reduced steric hindrance in the hemisphere of the anion (Me vs TIP) but also a completely free Felkin hemisphere (H vs Me), which would clearly favor the inversion process in the borylation step. For **9-c2**, we propose that this change already occurs during the formation of the anion since otherwise the Felkin hemisphere would be sterically too hindered to accommodate the anion with the respective ligands. **Li-9-c2** then shows a completely free Felkin hemisphere, meaning that this anion would react under retention of configuration during borylation.

**Scheme 11:**
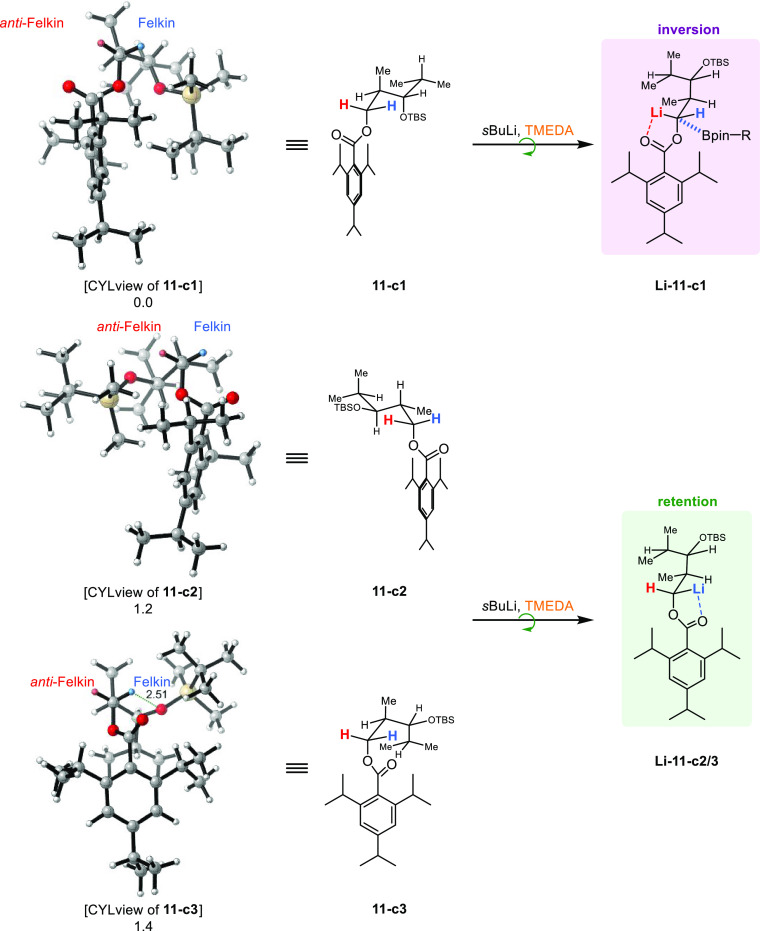
Rationalization for Inversion and Retention Processes during Borylation in the Case of *anti*-TIB Ester **11** Values below the structures are relative energies in kJ/mol.

The application of the conformational change also explains the observed selectivities in the case of *anti*-TIB ester **11** (Scheme 11). Thus, in the conformation of the anion derived by **11-c1**, the *anti*-Felkin hemisphere would again be occupied by the large TIP residue. In addition, the methyl group also points clearly into the *anti*-Felkin hemisphere, resulting in a large steric hindrance. A small conformational change while forming the anion, in which the TIP residue is rotated out of the *anti*-Felkin hemisphere and consequential rotation of the back part of the molecule (TIP vs OTBS), leads to **Li-11-c1**. Anion **Li-11-c1** then again shows a completely free Felkin hemisphere (H vs Me), which explains the borylation of this anion under inversion of configuration. The conformational change during anion formation of **11-c2** (TIP and back part) and **11-c3** (back part) would lead to anion **Li-11-c2/3** exhibiting a completely free Felkin hemisphere, allowing the borylation under retention of configuration.

The degree of retention and inversion depends thereby strongly on the size of the electrophile used. The branched vinyl boronic esters **5** and **6** appear to react after the conformational change, whereas unbranched **7** and **8** are less sensitive to steric hindrance. Accordingly, **7** and **8** may react predominantly out of the conformations close to the non-lithiated compounds, giving poorer selectivities.

**Scheme 12:**
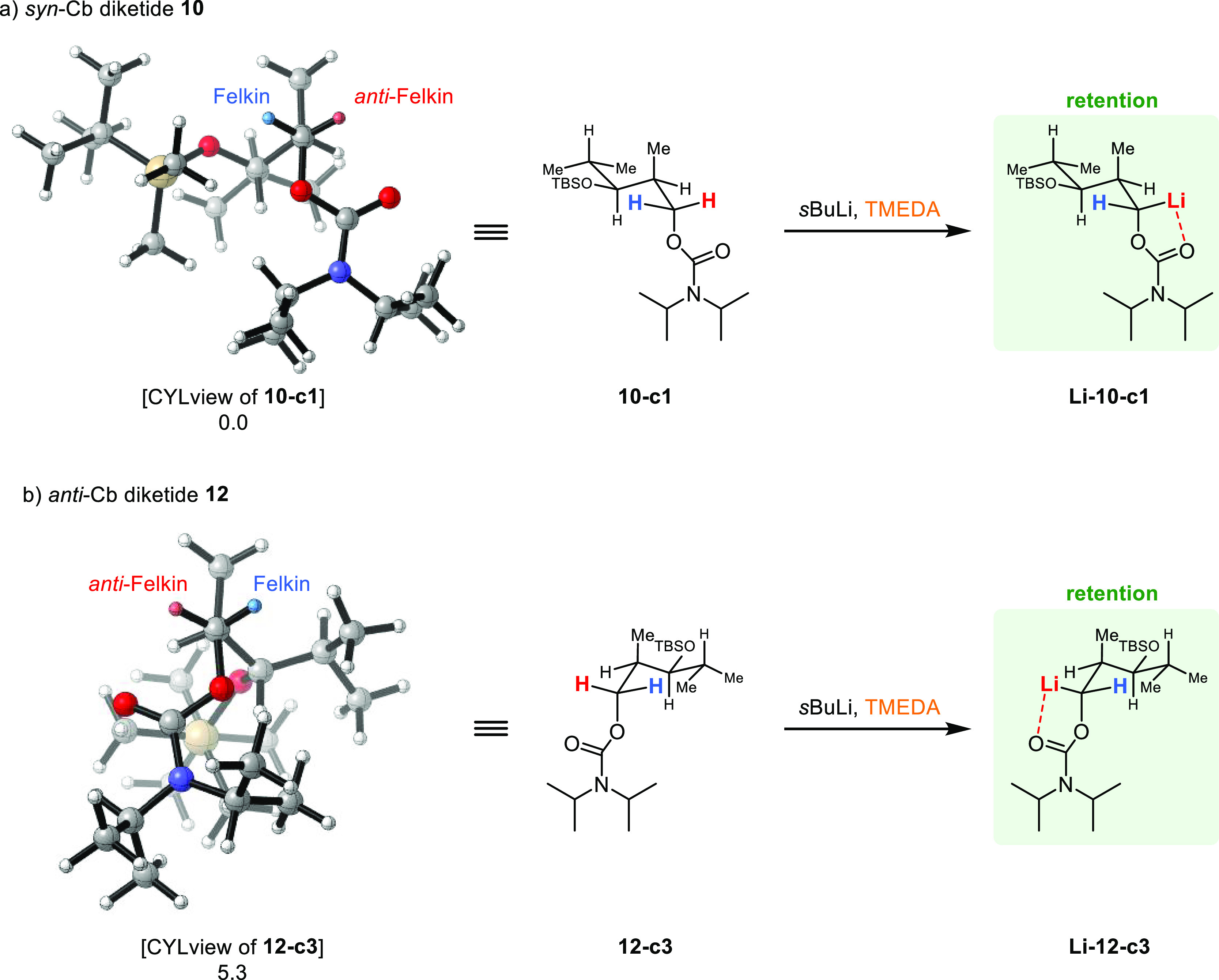
(a,b) Rationalization for Retention during Borylation in the Case of Cb Diketides **10** and **12** Values below the structures are relative energies in kJ/mol.

In the case of the carbamates, which in the borylation step predominantly react under the usual retention of configuration, our findings confirm a hypothesis made by Beak and co-workers.^56^ Beak reported that “highly reactive or non-lithium coordinating electrophiles proceed with inversion, while less reactive and lithium coordinating electrophiles give retention.”^56^ For the chemistry we discovered, this hypothesis only needs to be slightly adapted, namely, by replacing electrophiles by nucleophiles. However, the different reactivity of the TIB and Cb directing groups can be recognized by the fact that the Cb group requires MgBr_2_·OEt_2_ as an additional Lewis acid to initiate the 1,2-metallate rearrangement, thus marking the carbamates as less reactive than the TIB esters. Furthermore, due to the amide resonance enabled in the carbamates, the Cb group can coordinate the lithium much more efficiently, which also fulfills the second (lithium coordinating) of Beak’s criteria.^56^ We can also rationalize the predominant reaction under retention of configuration in the borylation step via the size of the Cb group (Scheme 12). In contrast to the TIB group, the Cb group does not occupy as much space in the hemisphere of the anion (to be formed), so the anions are likely to adopt a conformation very similar to the starting compounds, and no conformational change is necessary. Thus, the Cb-derived anions would adopt the conformations **Li-10-c1** and **Li-12-c3**, which both have a less hindered *anti*-Felkin hemisphere and thus react preferentially under retention of configuration.

## Summary

**Scheme 13:**
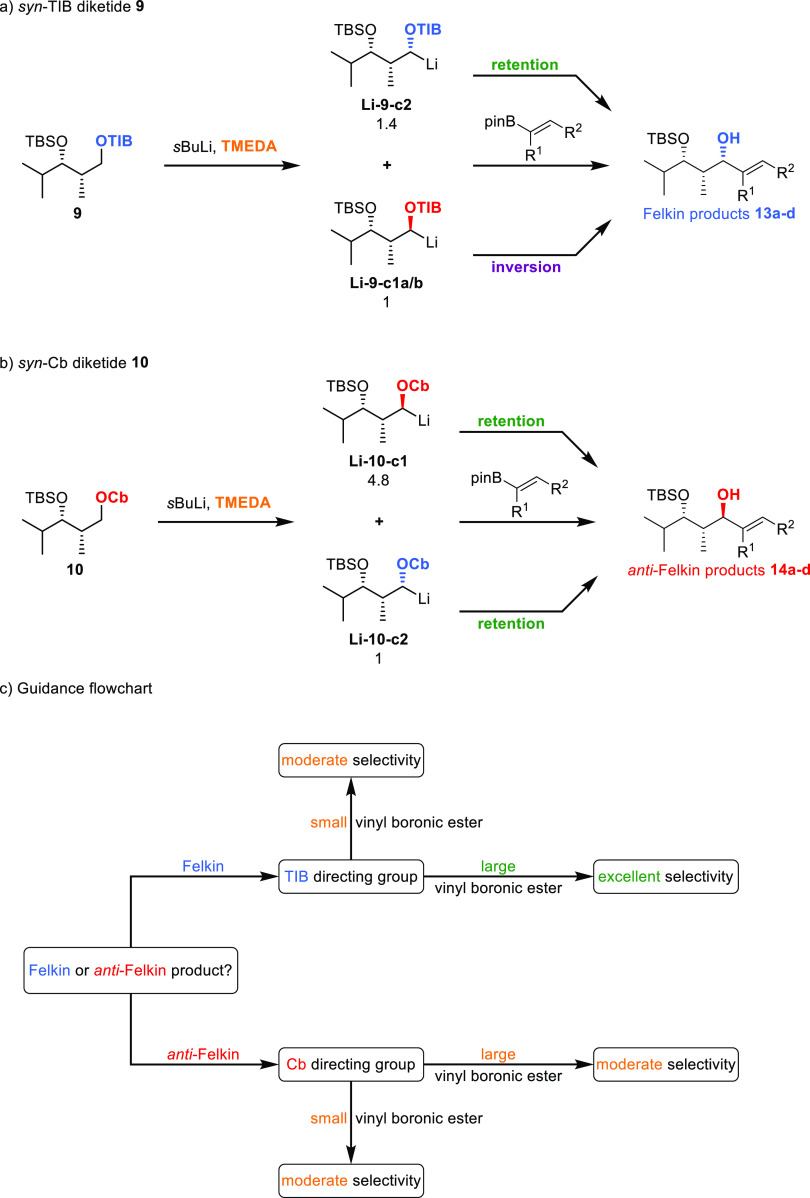
(a,b) Summary of the substrate- and reagent-controlled lithiation–borylation chemistry of diketides and vinyl boronic estersreasons of clarity, only the *syn*-diketide is shown; however, the same analysis is also valid for *anti*-diketides; (c) guidance flowchart for the substrate- and reagent-controlled lithiation–borylation chemistry of diketides and vinyl boronic estersvalues below the structures are relative energies in kj/mol.

In summary, we have shown that deprotonation of TIB-derived diketides with the achiral diamine TMEDA occurs with low diastereomeric ratios (1.4:1, 3.1:1, Scheme 4c). The so-obtained organolithiums react with vinyl boronic esters under both retention and inversion depending on the size of the electrophile used. With large electrophiles, the *anti*-Felkin Li-isomer reacts under inversion, while the Felkin anion reacts under retention giving high diastereomeric ratios. The same is true for small electrophiles, but in this case, the differentiation is less pronounced, resulting in lower diastereomeric ratios. In contrast, lithiation of the Cb-derived diketides with TMEDA occurs with higher diastereomeric ratios (4.8:1, 5:1, Scheme 4c), and the subsequent borylation takes place predominantly under retention of configuration (Scheme 13a,b).

Considering the experimentally observed selectivities and conformational analysis, we can establish a first guidance flowchart for the substrate- and reagent-controlled lithiation–borylation chemistry of diketides and vinyl boronic esters (Scheme 13c). In general, the TIB directing group favors the formation of the formal Felkin products, whereas the selectivity using large (branched) vinyl boronic esters is significantly higher through better differentiation between the Felkin and the *anti*-Felkin hemisphere in the borylation step.

The Cb directing group on the other hand favors the formation of the formal *anti*-Felkin products in moderate selectivities. Due to the nature of this directing group (small, no conformational change during anion formation), there is almost no effect of the steric demand exerted by the used vinyl boronic esters on the observed selectivities.

## Conclusions

In conclusion, we have developed a novel and high-yielding procedure for the stereocontrolled synthesis of allylic alcohols in the absence of chiral ligands. We have shown that the Hoppe–Matteson–Aggarwal protocol can be used as a powerful alternative to the NHTK reaction for the stereoselective synthesis of allylic alcohols. We observed outstanding selectivities even in the absence of sparteine and noticed that altering the directing group from TIB to Cb not only affects the yields and selectivities but also in most cases provides the opposite diastereoisomer. Accordingly, we were able to show that, in addition to Aggarwal’s findings on the influence of the diamine ligand,^57^ the directing group used and the electrophile also have a decisive influence on the stereochemical outcome of the borylation. In particular, for branched vinyl boronic esters—a quite common situation during a total synthesis—this switch in selectivity can be performed in practical yields and selectivities. As already described for aldol reactions by Paterson,^58^ the stereochemical outcome depends very much on the overall conformation, and even remote chiral centers can have substantial impact on the yields and selectivities, which is also shown by our calculations and the resulting mechanistic model. In the cases where this directing group alteration did not produce the desired yields and/or selectivities, the use of either (+)- or (−)-sparteine often gave improved yields and selectivities. Considering the abovementioned points, the presented protocol serves as a highly valuable alternative to the NHTK reaction and can contribute to total syntheses of natural products. Additional applications of this highly stereoselective methodology in natural product synthesis will be reported in due course.

## Methods

### General Procedure for the Synthesis of TIB Esters

The required primary alcohol (1.1 equiv) was dissolved in anhydrous tetrahydrofuran (THF) (0.3 M); PPh_3_ (1.0 equiv) and TIBOH (1.0 equiv) were added successively. After cooling to 0 °C, DIAD (1.1 equiv, 0.12 mL/min) was added, and the reaction mixture was slowly warmed to rt and stirred overnight at that temperature. MTBE and sat. aq. NaHCO_3_ were added, and the phases were separated. The aqueous phase was extracted with MTBE (3×), and the organic layers were combined and dried over Na_2_SO_4_. The crude material was loaded on silica and purified by flash column chromatography.

### General Procedure for the Synthesis of Carbamates

The required primary alcohol (1.0 equiv) was dissolved in 1,2-dichloroethane (0.3 M), and Et_3_N (3.0 equiv) and *N*,*N*-diisopropylcarbamoyl chloride (3.0 equiv) were added successively. After heating to 70 °C overnight, H_2_O was added. The phases were separated, and the aqueous phase was extracted with CH_2_Cl_2_ (3×); then, the organic layers were combined and dried over Na_2_SO_4_. The solvent was removed in vacuo, and the crude material was purified by flash column chromatography to afford the corresponding carbamate.

### General Procedure of Substrate-Controlled 1,2-Metallate Rearrangement of Vinyl Boronates with TIB Esters

To a stirred solution of TIB ester (1.5 equiv) and diamine (1.5 equiv) in Et_2_O (0.2 M) at −78 °C was added *s*BuLi (1.3 M in hexanes, 1.4 equiv). The reaction mixture was stirred for 5 h at that temperature before a solution of vinyl boronic ester (1.0 equiv) in Et_2_O (0.5 M) was added. After stirring for further 3 h at −78 °C, the reaction mixture was warmed to 45 °C and stirred overnight. The reaction mixture was cooled to rt, sat. aq. NH_4_Cl was added, and the biphasic mixture was stirred for 15 min. The phases were separated, the organic layer was washed with sat. aq. NH_4_Cl (3×), and the combined aqueous phases were extracted with MTBE (3×). The combined organic phases were dried over Na_2_SO_4_ and concentrated in vacuo, and the crude material was purified by short flash column chromatography (to remove TIBOH). The residue was dissolved in THF (0.2 M) and cooled to −20 °C. A premixed, ice-cooled solution of NaOH (2.0 M)/H_2_O_2_ (35%, 2/1 v/v, 0.12 M) was added dropwise. The reaction mixture was stirred at rt before being diluted with MTBE and quenched by the slow addition of sat. aq. Na_2_S_2_O_3_ at 0 °C after thin-layer chromatography (TLC) showed full conversion. The solution was diluted with MTBE, the phases were separated, and the aqueous phase was extracted with MTBE (3x). The combined organic layers were dried over Na_2_SO_4_ and concentrated in vacuo. The crude product was purified by flash column chromatography to afford allylic alcohol.

### General Procedure of Substrate-Controlled 1,2-Metallate Rearrangement of Vinyl Boronates with Carbamates

To a stirred solution of carbamate (1.5 equiv) and diamine (1.5 equiv) in Et_2_O (0.2 M) at −78 °C was added *s*BuLi (1.3 M in hexanes, 1.4 equiv). The reaction mixture was stirred for 5 h at that temperature before a solution of vinyl boronic ester (1.0 equiv) in Et_2_O (0.5 M) was added. The reaction mixture was stirred for 3 h at −78 °C. In parallel, magnesium turnings were activated (2× 1.0 M HCl, 2× H_2_O, 2× acetone, drying under high vacuum). The required amount (2.0 equiv) was dissolved in Et_2_O (0.8 M), and 1,2-dibromoethane (2.0 equiv) was added under water bath cooling. The reaction mixture was stirred for 2 h at this temperature. The biphasic MgBr_2_·OEt_2_ solution was added dropwise to the main reaction mixture, which was then stirred for another 30 min at −78 °C before being warmed to 45 °C and stirred overnight. The reaction mixture was cooled to rt, sat. aq. NH_4_Cl was added, and the biphasic mixture was stirred for 15 min. The phases were separated, the organic layer was washed with sat. aq. NH_4_Cl (3×), and the combined aqueous phases were extracted with MTBE (3×). The combined organic phases were dried over Na_2_SO_4_ and concentrated in vacuo, and the crude material was purified by short flash column chromatography (to remove excess of the carbamate). The residue was dissolved in THF (0.2 M) and cooled to −20 °C. A premixed, ice-cooled solution of NaOH (2.0 M)/H_2_O_2_ (35%, 2/1 v/v, 0.12 M) was added dropwise. The reaction mixture was stirred at rt before being diluted with MTBE and quenched by the slow addition of sat. aq. Na_2_S_2_O_3_ at 0 °C after TLC showed full conversion. The solution was diluted with MTBE, the phases were separated, and the aqueous phase was extracted with MTBE (3×). The combined organic layers were dried over Na_2_SO_4_ and concentrated in vacuo. The crude product was purified by flash column chromatography to afford allylic alcohol.

## Supporting Information Available

Experimental procedures, computational details, and compound characterization (PDF)

## Abbreviations

Bn: benzyl
Bu: butyl
Cb: *N,N*-diisopropyl carbamoyl
Cbx: (3,3-dimethyl-1-oxa-4-azaspiro[4.5]decan-4-yl)carbonyl
Cby: (2,2,4,4-tetramethyloxazolidin-3-yl)carbonyl
Me: methyl
Ph: phenyl
pin: pinacolato
sp: sparteine
TBS: *tert*-butyldimethylsilyl
TMEDA: *N,N,N′,N′*-tetramethylethylenediamine
TMS: trimethylsilyl
TIB: 2,4,6-triisopropylbenzoyl
TIP: 2,4,6-triisopropylphenyl
p-Tol: p-tolyl

## References

[ref1] Gil A.; Albericio F.; Álvarez M. Role of the Nozaki–Hiyama–Takai–Kishi Reaction in the Synthesis of Natural Products. Chem. Rev. 2017, 117, 8420–8446. 10.1021/acs.chemrev.7b00144. 28627170

[ref2] Takao K.-I.; Ogura A.; Yoshida K.; Simizu S. Total Synthesis of Natural Products Using Intramolecular Nozaki-Hiyama-Takai-Kishi Reactions. Synlett 2020, 31, 421–433. 10.1055/s-0039-1691580.

[ref3] Linne Y.; Bonandi E.; Tabet C.; Geldsetzer J.; Kalesse M. The Total Synthesis of Chondrochloren A. Angew. Chem., Int. Ed. 2021, 60, 6938–6942. 10.1002/anie.202016072. PMC8048958 33450788

[ref4] Stymiest J. L.; Bagutski V.; French R. M.; Aggarwal V. K. Enantiodivergent conversion of chiral secondary alcohols into tertiary alcohols. Nature 2008, 456, 778–782. 10.1038/nature07592. 19079057

[ref5] Burns M.; Essafi S.; Bame J. R.; Bull S. P.; Webster M. P.; Balieu S.; Dale J. W.; Butts C. P.; Harvey J. N.; Aggarwal V. K. Assembly-line synthesis of organic molecules with tailored shapes. Nature 2014, 513, 183–188. 10.1038/nature13711. 25209797 PMC4167605

[ref6] Balieu S.; Hallett G. E.; Burns M.; Bootwicha T.; Studley J.; Aggarwal V. K. Toward Ideality: The Synthesis of (+)-Kalkitoxin and (+)-Hydroxyphthioceranic Acid by Assembly-Line Synthesis. J. Am. Chem. Soc. 2015, 137, 4398–4403. 10.1021/ja512875g. 25625684

[ref7] Ahrens H.; Paetow M.; Hoppe D. Stereoselective generation of 1,3- and 1,4-dioxy-substituted carbanions by sparteine-assisted deprotonation of chiral precursors: Substrate or reagent control in the synthesis of α,γ- and α,δ-diols. Tetrahedron Lett. 1992, 33, 5327–5330. 10.1016/S0040-4039(00)79084-8.

[ref8] Haller J.; Hense T.; Hoppe D. Kinetic Resolution of β-Stereogenic O-Alkyl Carbamates by (−)-Sparteine-Assisted Deprotonation. External versus Internal Chiral Induction. Synlett 1993, 726–728. 10.1055/s-1993-22585.

[ref9] Helmke H.; Hoppe D. Chelation-Directed Asymmetric Lithiation and C-Substitution of 1,2,4-Butanetriol Acetonide. Synlett 1995, 978–980. 10.1055/s-1995-5115.

[ref10] Fiorito D.; Keskin S.; Bateman J. M.; George M.; Noble A.; Aggarwal V. K. Stereocontrolled Total Synthesis of Bastimolide B Using Iterative Homologation of Boronic Esters. J. Am. Chem. Soc. 2022, 144, 7995–8001. 10.1021/jacs.2c03192. 35499478 PMC9100475

[ref11] Hoyt A. L.; Blakemore P. R. On the nature of the chain-extending species in organolithium initiated stereospecific reagent-controlled homologation reactions using α-chloroalkyl aryl sulfoxides. Tetrahedron Lett. 2015, 56, 2980–2982. 10.1016/j.tetlet.2014.08.123.

[ref12] Emerson C. R.; Zakharov L. N.; Blakemore P. R. Investigation of Functionalized α-Chloroalkyllithiums for a Stereospecific Reagent-Controlled Homologation Approach to the Analgesic Alkaloid (−)-Epibatidine. Chem. – Eur. J. 2013, 19, 16342–16356. 10.1002/chem.201302511. 24127119

[ref13] Sun X.; Blakemore P. R. Programmed Synthesis of a Contiguous Stereotriad Motif by Triple Stereospecific Reagent-Controlled Homologation. Org. Lett. 2013, 15, 4500–4503. 10.1021/ol402049y. 23947788

[ref14] Emerson C. R.; Zakharov L. N.; Blakemore P. R. Iterative Stereospecific Reagent-Controlled Homologation Using a Functionalized α-Chloroalkyllithium: Synthesis of Cyclic Targets Related to Epibatidine. Org. Lett. 2011, 13, 1318–1321. 10.1021/ol103170y. 21338072

[ref15] Blakemore P. R.; Burge M. S.; Stephton M. A. Competing reaction pathways from α-halo-α-protioalkyl aryl sulfoxides initiated by organometallic reagents. Tetrahedron Lett. 2007, 48, 3999–4002. 10.1016/j.tetlet.2007.04.031.

[ref16] Blakemore P. R.; Burge M. S. Iterative Stereospecific Reagent-Controlled Homologation of Pinacol Boronates by Enantioenriched α-Chloroalkyllithium Reagents. J. Am. Chem. Soc. 2007, 129, 3068–3069. 10.1021/ja068808s. 17326640

[ref17] Blakemore P. R.; Marsden S. P.; Vater H. D. Reagent-Controlled Asymmetric Homologation of Boronic Esters by Enantioenriched Main-Group Chiral Carbenoids. Org. Lett. 2006, 8, 773–776. 10.1021/ol053055k. 16468764

[ref18] Rayner P. J.; O’Brien P.; Horan R. A. J. Preparation and Reactions of Enantiomerically Pure α-Functionalized Grignard Reagents. J. Am. Chem. Soc. 2013, 135, 8071–8077. 10.1021/ja4033956. 23647498

[ref19] Casoni G.; Kucukdisli M.; Fordham J. M.; Burns M.; Myers E. L.; Aggarwal V. K. α-Sulfinyl Benzoates as Precursors to Li and Mg Carbenoids for the Stereoselective Iterative Homologation of Boronic Esters. J. Am. Chem. Soc. 2017, 139, 11877–11886. 10.1021/jacs.7b05457. 28812893

[ref20] Fang G. Y.; Aggarwal V. K. Asymmetric Synthesis of α-Substituted Allyl Boranes and Their Application in the Synthesis of Iso-agatharesinol. Angew. Chem., Int. Ed. 2007, 46, 359–362. 10.1002/anie.200603659. 17146825

[ref21] Althaus M.; Mahmood A.; Suárez J. R.; Thomas S. P.; Aggarwal V. K. Application of the Lithiation–Borylation Reaction to the Preparation of Enantioenriched Allylic Boron Reagents and Subsequent In Situ Conversion into 1,2,4-Trisubstituted Homoallylic Alcohols with Complete Control over All Elements of Stereochemistry. J. Am. Chem. Soc. 2010, 132, 4025–4028. 10.1021/ja910593w. 20192266

[ref22] García-Ruiz C.; Chen J. L. Y.; Sandford C.; Feeney K.; Lorenzo P.; Berionni G.; Mayr H.; Aggarwal V. K. Stereospecific Allylic Functionalization: The Reactions of Allylboronate Complexes with Electrophiles. J. Am. Chem. Soc. 2017, 139, 15324–15327. 10.1021/jacs.7b10240. 29028321 PMC5682599

[ref23] Still W. C.; Sreekumar C. alpha.-Alkoxyorganolithium reagents. A new class of configurationally stable carbanions for organic synthesis. J. Am. Chem. Soc. 1980, 102, 1201–1202. 10.1021/ja00523a066.

[ref24] Hoppe D.; Hense T. Enantioselective Synthesis with Lithium/(−)-Sparteine Carbanion Pairs. Angew. Chem., Int. Ed. 1997, 36, 2282–2316. 10.1002/anie.199722821.

[ref25] Dutheuil G.; Webster M. P.; Worthington P. A.; Aggarwal V. K. Stereocontrolled Synthesis of Carbon Chains Bearing Contiguous Methyl Groups by Iterative Boronic Ester Homologations: Application to the Total Synthesis of (+)-Faranal. Angew. Chem., Int. Ed. 2009, 48, 6317–6319. 10.1002/anie.200901194. 19437526

[ref26] Hoppe D.; Carstens A.; Kramev T. Generation of a Configurationally Stable Chiral Benzyllithium Derivative, and the Capricious Stereochemistry of Its Electrophilic Substitution. Angew. Chem., Int. Ed. 1990, 12, 1424–1425. 10.1002/anie.199014241.

[ref27] Gallagher D. J.; Kerrick S. T.; Beak P. Enantioselective deprotonation: the structure and reactivity of an unsymmetrically complexed isopropyllithium/sparteine/Et_2_O dimer. J. Am. Chem. Soc. 1992, 114, 5872–5873. 10.1021/ja00040a066.

[ref28] Hoppe D.; Hintze F.; Tebben P.; Paetow M.; Ahrens H.; Schwerdtfeger J.; Sommerfeld P.; Haller J.; Guarnieri W.; Kolczewski S.; Hense T.; Hoppe I. Enantioselective synthesis via sparteine-induced asymmetric deprotonation. Pure Appl. Chem. 1994, 66, 1479–1486. 10.1351/pac199466071479.

[ref29] Würthwein E.-U.; Hoppe D. Enantioselective Lithiation of O-Alkyl and O-Alk-2-enyl Carbamates in the Presence of (−)-Sparteine and (−)-α-Isosparteine. A Theoretical Study. J. Org. Chem. 2005, 70, 4443–4451. 10.1021/jo050253g. 15903323

[ref30] Stymiest J. L.; Dutheuil G.; Mahmood A.; Aggarwal V. K. Lithiated Carbamates: Chiral Carbenoids for Iterative Homologation of Boranes and Boronic Esters. Angew. Chem., Int. Ed. 2007, 46, 7491–7494. 10.1002/anie.200702146. 17659521

[ref31] Grimme S. Exploration of Chemical Compound, Conformer, and Reaction Space with Meta-Dynamics Simulations Based on Tight-Binding Quantum Chemical Calculations. J. Chem. Theory Comput. 2019, 15, 2847–2862. 10.1021/acs.jctc.9b00143. 30943025

[ref32] Pracht P.; Bohle F.; Grimme S. Automated Exploration of the Low-Energy Chemical Space with Fast Quantum Chemical Methods. Phys. Chem. Chem. Phys. 2020, 22, 7169–7192. 10.1039/C9CP06869D. 32073075

[ref33] Frisch M. J.; Trucks G. W.; Schlegel H. B.; Scuseria G. E.; Robb M. A.; Cheeseman J. R.; Scalmani G.; Barone V.; Petersson G. A.; Nakatsuji H.; Li X.; Caricato M.; Marenich A. V.; Bloino J.; Janesko B. G.; Gomperts R.; Mennucci B.; Hratchian H. P.; Ortiz J. V.; Izmaylov A. F.; Sonnenberg J. L.; Williams-Young D.; Ding F.; Lipparini F.; Egidi F.; Goings J.; Peng B.; Petrone A.; Henderson T.; Ranasinghe D.; Zakrzewski V. G.; Gao J.; Rega N.; Zheng G.; Liang W.; Hada M.; Ehara M.; Toyota K.; Fukuda R.; Hasegawa J.; Ishida M.; Nakajima T.; Honda Y.; Kitao O.; Nakai H.; Vreven T.; Throssell K.; Montgomery J. A. Jr.; Peralta J. E.; Ogliaro F.; Bearpark M. J.; Heyd J. J.; Brothers E. N.; Kudin K. N.; Staroverov V. N.; Keith T. A.; Kobayashi R.; Normand J.; Raghavachari K.; Rendell A. P.; Burant J. C.; Iyengar S. S.; Tomasi J.; Cossi M.; Millam J. M.; Klene M.; Adamo C.; Cammi R.; Ochterski J. W.; Martin R. L.; Morokuma K.; Farkas O.; Foresman J. B.; Fox D. J. Gaussian 16 Revision B.01, 2016.

[ref34] Becke A. D. Density-functional Thermochemistry. III. The Role of Exact Exchange. J. Chem. Phys. 1993, 98, 5648–5652. 10.1063/1.464913.

[ref35] Grimme S.; Ehrlich S.; Goerigk L. Effect of the Damping Function in Dispersion Corrected Density Functional Theory. J. Comput. Chem. 2011, 32, 1456–1465. 10.1002/jcc.21759. 21370243

[ref36] Grimme S.; Antony J.; Ehrlich S.; Krieg H. A Consistent and Accurate Ab Initio Parametrization of Density Functional Dispersion Correction (DFT-D) for the 94 Elements H-Pu. J. Chem. Phys. 2010, 132, 154104 10.1063/1.3382344. 20423165

[ref37] Tomasi J.; Mennucci B.; Cammi R. Quantum Mechanical Continuum Solvation Models. Chem. Rev. 2005, 105, 2999–3094. 10.1021/cr9904009. 16092826

[ref38] In the case of syn-TIB ester **9**, the relative energies were calculated for the corresponding enantiomer. However, since this has no effect on the relative energy difference, the experimentally used enantiomer is shown in the figures for reasons of comprehensibility.

[ref39] Eliel E. L. Stereochemistry of Carbon Compounds; McGraw-Hill: New York, 1962; pp 149–156.

[ref40] Zefirov N. S. Stereochemical studies—XXI: General equation of relationship between products ratio and conformational equilibrium. Tetrahedron 1977, 33, 2719–2722. 10.1016/0040-4020(77)80296-2.

[ref41] Seeman J. I.; Farone W. A. Analytical solution to the Curtin-Hammett/Winstein-Holness kinetic system. J. Org. Chem. 1978, 43, 1854–1864. 10.1021/jo00404a002.

[ref42] Dauben W. G.; Pitzer K. S. Steric Effects. In Organic Chemistry; Newman M. S., Ed.; Wiley: New York, 1956; Chapter 1.

[ref43] Eliel E. L.; Allinger N.; Angyal S. J.; Morrison G. A. Conformational Analysis; Wiley: New York, 1966; pp 25–35.

[ref44] Gold V. Glossary of terms used in physical organic chemistry. Pure Appl. Chem. 1979, 51, 1725–1801. 10.1515/pac-2018-1010.

[ref45] Seeman J. I. Effect of conformational change on reactivity in organic chemistry. Evaluations, applications, and extensions of Curtin-Hammett Winstein-Holness kinetics. Chem. Rev. 1983, 83, 83–134. 10.1021/cr00054a001.

[ref46] Morozova V.; Skotnitzki J.; Moriya K.; Karaghiosoff K.; Knochel P. Preparation of Optically Enriched Secondary Alkyllithium and Alkylcopper Reagents-Synthesis of (−)-Lardolure and Siphonarienal. Angew. Chem., Int. Ed. 2018, 57, 5516–5519. 10.1002/anie.201800792. 29512307

[ref47] Dagousset G.; Moriya K.; Mose R.; Berionni G.; Karaghiosoff K.; Knochel P. Diastereoselective Synthesis of Open-Chain Secondary Alkyllithium Compounds and Trapping Reactions with Electrophiles. Angew. Chem., Int. Ed. 2014, 53, 1425–1429. 10.1002/anie.201308679. 24459059

[ref48] Moriya K.; Didier D.; Simon M.; Hammann J. M.; Berionni G.; Karaghiosoff K.; Zipse H.; Mayr H.; Knochel P. Stereoselective Synthesis and Reactions of Secondary Alkyllithium Reagents Functionalized at the 3-Position. Angew. Chem., Int. Ed. 2015, 54, 2754–2757. 10.1002/anie.201409165. 25640227

[ref49] Morozova V.; Moriya K.; Mayer P.; Knochel P. Stereoselective Synthesis and Retentive Trapping of α-Chiral Secondary Alkyllithiums Leading to Stereodefined α,β-Dimethyl Carboxylic Esters. Chem. – Eur. J. 2016, 22, 9962–9965. 10.1002/chem.201601911. 27140953

[ref50] Skotnitzki J.; Kremsmair A.; Knochel P. Stereoselective Preparation and Reactions of Chiral Secondary Alkyllithiums. Synthesis 2020, 52, 189–196. 10.1055/s-0039-1690713.

[ref51] Rutherford J. L.; Hoffmann D.; Collum D. B. Consequences of Correlated Solvation on the Structures and Reactivities of RLi-Diamine Complexes: 1,2-Addition and α-Lithiation Reactions of Imines by TMEDA-Solvated *n*-Butyllithium and Phenyllithium. J. Am. Chem. Soc. 2002, 124, 264–271. 10.1021/ja002979u. 11782178

[ref52] Bryantsev V. S.; Diallo M. S.; Goddard W. A. pKa Calculations of Aliphatic Amines, Diamines, and Aminoamides via Density Functional Theory with a Poisson–Boltzmann Continuum Solvent Model. J. Phys. Chem. A 2007, 111, 4422–4430. 10.1021/jp071040t. 17469810

[ref53] Carbone G.; O’Brien P.; Hilmersson G. Asymmetric Deprotonation using *s*-BuLi or *i*-PrLi and Chiral Diamines in THF: The Diamine Matters. J. Am. Chem. Soc. 2010, 132, 15445–15450. 10.1021/ja107672h. 20936816

[ref54] Mykura R. C.; Veth S.; Varela A.; Dewis L.; Farndon J. J.; Myers E. L.; Aggarwal V. K. Investigation of the Deprotonative Generation and Borylation of Diamine-Ligated α-Lithiated Carbamates and Benzoates by in situ IR spectroscopy. J. Am. Chem. Soc. 2018, 140, 14677–14686. 10.1021/jacs.8b06871. 30260635

[ref55] Jemmis E. D.; Chandrasekhar J.; Schleyer P. v. R. Stabilization of D_3h_ Pentacoordinate Carbonium Ions. Linear Three-Center-Two-Electron Bonds. Implications for Aliphatic Electrophilic Substitution Reactions. J. Am. Chem. Soc. 1979, 101, 527–533. 10.1021/ja00497a004.

[ref56] Park Y. S.; Beak P. Enantioselective Syntheses of α-, β-, and γ-Aryl Amino Acids and Esters. J. Org. Chem. 1997, 62, 1574–1575. 10.1021/jo9700080.

[ref57] Binanzer M.; Fang G. Y.; Aggarwal V. K. Asymmetric Synthesis of Allylsilanes by the Borylation of Lithiated Carbamates: Formal Total Synthesis of (−)-Decarestrictine D. Angew. Chem., Int. Ed. 2010, 49, 4264–4268. 10.1002/anie.201001223. 20446329

[ref58] Paterson I.; Ward R. A.; Smith J. D.; Cumming J. G. The total synthesis of swinholide A. Part 2: A stereocontrolled synthesis of a C1–C15 segment. Tetrahedron 1995, 51, 9431–9466. 10.1021/jo00038a002.

