# Supplementary material for: Sparteine-Free, Highly Stereoselective Construction of Complex Allylic Alcohols Using 1,2-Metallate Rearrangements
Source: JACS Au. 2023 May 22;3(6):1695–710. doi: 10.1021/jacsau.3c00114 (PMC10301690; doi:10.1021/jacsau.3c00114)
Supplement: Supplementary file 1 — au3c00114_si_001.pdf [file au3c00114_si_001.pdf]

Supporting Information

Sparteine-Free, Highly Stereoselective Construction of Complex Allylic Alcohols Using 1,2-Metallate Rearrangements

Yannick Linne, Maike Birkner, Jan Flormann, Daniel Lücke, Jörg August Becker, and Markus Kalesse*

Institute of Organic Chemistry, Gottfried Wilhelm Leibniz Universität Hannover, Schneiderberg 1B, 30167 Hannover (Germany)

Corresponding Author

*markus.kalesse@oci.uni-hannover.de

Table of Contents

1. General Considerations……………………………………………………S1

2. Experimental Procedures and Characterization Data…………………..S3

2-1. Synthesis of boronic esters……………………………………………………….S3

2-2. Synthesis of the syn-motif…………………………………………………….S4

2-3. Synthesis of the anti-motif ……………………………………………………S6

2-4. Synthesis of TIB esters…………………………………………………………S8

2-5. Synthesis of carbamates……………………………………………………….S10

2-6. Substrate-controlled 1,2-metallate rearrangement of vinyl boronates………….S12

2-7. Analysis of stereochemistry……………………………………………………S23

2-8. Rationalization of substrate control…………………………………………S26

3. Conformational analysis………………………………………………S43

3-1. Conformational search with CREST…………………………………………..S43

3-2. DFT-optimization…………………………………………………………….S47

3-3. Calculation of Boltzmann factors……………………………………………S63

4. References………………………………………………………………S65

5. Spectra………………………………………………………………….S67

6. Appendix……………………………………………………………….S135

1. General Considerations

Unless otherwise noted all reactions were carried out under an argon atmosphere using a Drierite™ gas-drying unit. The used glassware was flame dried under high vacuum. Air- and moisture-sensitive liquids and solutions were transferred via syringe flushed with argon prior to use. All reagents were purchased from commercial suppliers and used without further purification unless otherwise noted. Vinylboronic acid pinacol ester (**8**) was bought from Sigma Aldrich and Alfa Aesar and was distilled prior to use. Vinyl boronic ester **7**[1] and vinyl boronic ester **6**[2] were prepared according to literature. **(+)-Sparteine** was purchased from Chem-Impex and **(−)-sparteine** was bought from TCI. Both were distilled over CaH_2 under high vacuum and stored under argon at −25 °C. Stated temperatures, except room temperature, refer to bath temperatures.

Dry solvents Dichloromethane and all amine bases were distilled under an inert atmosphere over calcium hydride. Tetrahydrofuran, diethyl ether and methanol were purchased from Acros Organics over molecular sieves and under inert atmosphere.

Thin layer chromatography All reactions were stirred magnetically and monitored using pre-coated TLC sheets ALUGRAM® Xtra SIL G/UV_{254} (0.2 mm, silica gel, F_{254}, aluminum-backed, MACHEREY-NAGEL) with detection by UV light (λ = 254 nm) and/or by staining with either basic potassium permanganate, acidic ceric ammonium molybdate, acidic anisaldehyde or acidic vanillin stain.

Flash column chromatography was performed using silica gel (0.04-0.063 mm, 240-400 mesh) obtained from MACHEREY-NAGEL. The applied petroleum ether fraction had a bp of 40-60 °C. The eluent is given in volume ratios (v/v).

^{1}H-NMR experiments were recorded in $CDCl_3$ or C_6D_6 using either a DPX 400 (Bruker), an AMX 400 (Bruker) or an Ascend 400 Avance III HD (Bruker). The spectra were calibrated using the residual solvent peak: $\delta(CDCl_3)$ = 7.26 ppm, $\delta(C_6D_6)$ = 7.16 ppm. Chemical shift δ is given in parts per million (ppm), coupling constant J in hertz (Hz) and multiplicity as follows: s, singlet; d, doublet; t, triplet; q, quadruplet; p, pentet; sex, sextet; sep, septet; m, multiplet; m_c, centered multiplet; br, broad; or combination of these acronyms. NMR spectra were processed using TopSpin (Bruker). Field strength and NMR solvent are given with every single procedure. Signals not attributed to the product are either diastereoisomers, unreacted starting material or TIB acid. Specifications can be found under the respective synthetic procedure.

^{13}C-NMR experiments were recorded in $CDCl_3$ or C_6D_6 using either a DPX 400 (Bruker), an AMX 400 (Bruker) or an Ascend 400 Avance III HD (Bruker). The spectra were calibrated using the residual solvent peak: $\delta(CDCl_3)$ = 77.16 ppm, $\delta(C_6D_6)$ = 128.06 ppm. Chemical

shift δ is given in parts per million (ppm). NMR spectra were processed using TopSpin (Bruker).

Diastereomeric ratios were determined via integration of the respective signals in the ^{1}H-NMR spectra and are shown (in small sections) in the corresponding spectra.

High Resolution Mass Spectra (HRMS) were obtained either using a Q-Tof Premier (Waters), a LCT Premier (Waters) or a GC-system Agilent 6890 coupled with an Agilent 5973. Both the masses found and the masses calculated are given.

Optical rotation $[\boldsymbol{\alpha}]_{\boldsymbol{D}}^{\boldsymbol{T}}$ were measured either on a P3000 polarimeter (A. Krüss Optronic, λ = 589 nm) or a Perkin-Elmer 341 (λ = 589 nm). The sample concentration (in g/100 mL) is given with every single experiment.

2. Experimental Procedures and Characterization Data

2-1. Synthesis of boronic esters

Vinyl boronic ester 5

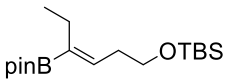

5

$C_{18}H_{37}BO_3Si$

340.39

According to Aggarwal's procedure[2]; in the glovebox, CuCl (0.23 g, 2.29 mmol, 0.5 equiv) and KO*t*Bu (1.03 g, 9.15 mmol, 2.0 equiv) were transferred to a Schlenk-flask. Then recrystallized PPh_3 (0.72 g, 2.74 mmol, 0.6 equiv) was added. The flask was then evacuated and purged with argon (3x). After the addition of THF (23.0 mL) the catalyst suspension was stirred for 30 min at rt.

In a separate Schlenk-flask B_2pin_2 (1.28 g, 5.03 mmol, 1.1 equiv) was dissolved in THF (12.5 mL). After the addition of 1/10 (2.3 mL) of the catalyst suspension the reaction mixture was stirred for 15 min at rt. The reaction mixture was then cooled to 0 °C and a solution of 3-hexyn-1-ol (0.50 mL, 4.57 mmol, 1.0 equiv) in THF (14.0 mL) and MeOH (0.37 mL, 9.14 mmol, 2.0 equiv) were added successively. After the reaction mixture was slowly warmed to rt, it was stirred overnight. Sat. aq. NH_4Cl was added and the reaction mixture was stirred for further 30 min before being diluted with MTBE. The organic layer was separated and the aqueous layer was extracted with MTBE (3x). The combined organic layers were dried over Na_2SO_4 and concentrated *in vacuo*. The crude product was purified by flash column chromatography (PE:MTBE 2:1) to afford the corresponding vinyl boronic ester which was used in the next step without detailed characterization.

To a stirred solution of the obtained vinyl boronic ester in CH_2Cl_2 (23.0 mL) at 0 °C were added imidazole (0.44 g, 6.40 mmol, 1.4 equiv) and TBSCl (0.90 g, 5.94 mmol, 1.3 equiv) successively. The reaction mixture was stirred for 3 h at rt before being quenched by the addition of sat. aq. $NaHCO_3$. The organic layer was separated and the aqueous layer was extracted with CH_2Cl_2 (3x). The combined organic layers were dried over Na_2SO_4 and concentrated *in vacuo*. The crude product was purified by flash column chromatography (PE:MTBE 98:2) to afford vinyl boronic ester **5** (0.79 g, 2.32 mmol, 51% o2s%) as a colorless oil.

^{1}H-NMR (400 MHz, C_6D_6): δ = 6.71 (t, *J* = 7.2 Hz, 1H), 3.58 (t, *J* = 6.9 Hz, 2H), 2.46-2.38 (m, 4H), 1.18 (t, *J* = 7.5 Hz, 3H), 1.09 (s, 12H), 0.97 (s, 9H), 0.05 (s, 6H) ppm;

^{13}C-NMR (101 MHz, C_6D_6): δ = 142.2, 83.0, 62.9, 32.6, 26.2, 24.9, 22.4, 18.6, 15.3, −5.1 ppm (carbon attached to boron not observed);

HRMS (ESI): $C_{18}H_{37}BO_3SiNa$ $[M+Na]^+$ calculated: 363.2503, found: 363.2510;

R*f* = 0.3 (PE:MTBE 98:2, vanillin).

2-2. Synthesis of the *syn*-motif

General Procedure 1 (GP1): Evans-Aldol Reaction, TBS-Protection, Reduction

A solution of the required propionated Evans auxiliary (1.0 equiv) in CH_2Cl_2 (0.1 M) was cooled to −78 °C and treated with Et_3N (1.3 equiv). Subsequently, *n*Bu_2BOTf (1.0 M in CH_2Cl_2, 1.15 equiv) was added. The reaction mixture was stirred at −78 °C for 1 h, was then allowed to warm to 0 °C and stirred at this temperature for 1 h until it was re-cooled to −78 °C and the corresponding aldehyde (1.3 equiv) was added. The reaction mixture was stirred at −78 °C for 2 h, until it was warmed to 0 °C and stirred for further 2 h. After the addition of pH 7 buffer, H_2O_2 (35%) and MeOH and further stirring for 30 min at 0 °C the organic layer was separated. The aqueous layer was extracted with CH_2Cl_2 (3x), the combined organic layers were washed with sat. aq. NaCl, dried over Na_2SO_4 and concentrated *in vacuo*.[3]

To a solution of the obtained *syn*-aldol product in CH_2Cl_2 (1.0 M) at −78 °C were added 2,6-lutidine (4.0 equiv) and TBSOTf (2.0 equiv) successively. The reaction mixture was stirred for 20 min at −78 °C and at 0 °C until TLC showed full conversion. After the addition of sat. aq. NH_4Cl the organic layer was separated and the aqueous layer was extracted with CH_2Cl_2 (3x). The combined organic layers were washed with aq. $KHSO_4$ (1.0 M) and sat. aq. NaCl, dried over Na_2SO_4 and concentrated *in vacuo*. The crude material was purified by flash column chromatography to afford the TBS-protected *syn*-aldol product.

To a solution of the TBS-protected *syn*-aldol product (1.0 equiv) in THF (0.1 M) and MeOH (8.0 equiv) at 0 °C was added $LiBH_4$ (4.0 M in THF, 8.0 equiv) dropwise. The reaction mixture was stirred for 30 min at 0 °C and overnight at rt. The reaction mixture was then cooled to 0 °C and sat. aq. NaCl and MTBE were added. The organic layer was separated and the aqueous layer was extracted with MTBE (3x). The combined organic layers were dried over Na_2SO_4 and concentrated *in vacuo*. The crude product was purified by flash column chromatography to afford the corresponding primary alcohol.[4]

1,2-*syn* alcohol S1

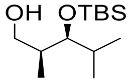

S1
$C_{13}H_{30}O_2Si$
246.47

Using GP1, (−)-4-benzyl-3-propionyl-2-oxazolidinone (3.00 g, 12.8 mmol, 1.0 equiv) and freshly distilled isobutyraldehyde (1.5 mL, 16.7 mmol, 1.3 equiv) gave primary alcohol **S1** (1.30 g, 5.27 mmol, 41% o3s, dr ≥ 19:1) after purification by flash column chromatography (PE:MTBE 7:1) as a colorless oil.

^{1}H-NMR (400 MHz, $CDCl_3$): δ = 3.62 (dd, J = 10.4, 8.1 Hz, 1H), 3.52 (dd, J = 5.7, 2.7 Hz, 1H), 3.47 (dd, J = 10.1, 5.9 Hz, 1H), 1.92 (m_c, 1H), 1.81 (m_c, 2H), 0.94-0.88 (m, 15H), 0.86 (d, J = 7.0 Hz, 3H), 0.08 (s, 3H), 0.06 (s, 3H) ppm;

^{13}C-NMR (101 MHz, $CDCl_3$): δ = 78.5, 66.6, 39.4, 31.6, 26.2, 20.5, 19.3, 18.5, 12.2, −3.8, −4.0 ppm;

HRMS (ESI): $C_{13}H_{30}O_2SiNa$ $[M+Na]^+$ calculated: 269.1913, found: 269.1920;

R$_f$ = 0.3 (PE:MTBE 4:1, vanillin);

$[\alpha]_D^{20}$ = +5.7 (*c* 0.9, $CHCl_3$).

Analytical data are in accordance with the literature.[5]

2-3. Synthesis of the *anti*-motif

General Procedure 2 (GP2): Oppolzer-Aldol Reaction, TBS-Protection, Reduction

The required propionated Oppolzer auxiliary (1.0 equiv) was dissolved in CH_2Cl_2 (1.0 M) and Et_3N (1.5 equiv) and TMSOTf (1.7 equiv) were added successively over a period of 15 min. The reaction mixture was then stirred overnight at rt. A solution of the required aldehyde (1.5 equiv) in CH_2Cl_2 (0.8 M) at −78 °C was treated with $TiCl_4$ (1.0 M in CH_2Cl_2, 1.5 equiv) over a period of 15 min. The solution containing the (*Z*)-ketene acetal was cooled to −78 °C and then transferred to the cooled aldehyde containing solution via cannula. After complete addition, the reaction mixture was stirred at −78 °C until TLC showed full conversion before being quenched by the addition of sat. aq. NH_4Cl. After warming to 0 °C and stirring for further 30 min at this temperature the organic layer was separated. The aqueous layer was extracted with CH_2Cl_2 (3x), the combined organic layers were dried over Na_2SO_4 and the solvent was removed *in vacuo*.
To a solution of the obtained *anti*-aldol product in CH_2Cl_2 (1.0 M) at −78 °C were added 2,6-lutidine (4.0 equiv) and TBSOTf (2.0 equiv) successively. The reaction mixture was stirred for 20 min at −78 °C and at 0 °C until TLC showed full conversion. After the addition of sat. aq. NH_4Cl the organic layer was separated and the aqueous layer was extracted with CH_2Cl_2 (3x). The combined organic layers were washed with aq. $KHSO_4$ (1.0 M) and sat. aq. NaCl, dried over Na_2SO_4 and concentrated *in vacuo*. The crude material was purified by flash column chromatography to afford the TBS-protected *anti*-aldol product.
To a solution of the TBS-protected *anti*-aldol product (1.0 equiv) in THF (0.1 M) and MeOH (8.0 equiv) at 0 °C was added $LiBH_4$ (4.0 M in THF, 8.0 equiv) dropwise. The reaction mixture was stirred for 30 min at 0 °C and overnight at rt. The reaction mixture was then cooled to 0 °C and sat. aq. NaCl and MTBE were added. The organic layer was separated and the aqueous layer was extracted with MTBE (3x). The combined organic layers were dried over Na_2SO_4 and concentrated *in vacuo*. The crude product was purified by flash column chromatography to afford the corresponding primary alcohol.[4]

1,2-*anti* alcohol S2

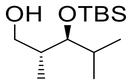

S2
$C_{13}H_{30}O_2Si$
246.47

Using GP2, (−)-*N*-propionyl sultam (1.50 g, 5.53 mmol, 1.0 equiv) and freshly distilled isobutyraldehyde (0.76 mL, 8.29 mmol, 1.5 equiv) gave primary alcohol **S2** (0.91 g, 3.69 mmol, 67% o3s, dr ≥ 19:1) after purification by flash column chromatography (PE:MTBE 4:1) as a colorless oil.

^{1}H-NMR (400 MHz, $CDCl_3$): δ = 3.67 (dd, J = 11.0, 4.2 Hz, 1H), 3.58 (dd, J = 11.0, 6.0 Hz, 1H), 3.43 (t, J = 4.9 Hz, 1H), 1.91-1.76 (m, 2H), 0.98 (d, J = 6.8 Hz, 3H), 0.95-0.88 (m, 15H), 0.11 (s, 3H), 0.08 (s, 3H) ppm;

^{13}C-NMR (101 MHz, $CDCl_3$): δ = 82.6, 66.2, 37.0, 33.3, 26.2, 19.1, 18.6, 18.4, 16.7, −3.8, −3.9 ppm;

HRMS (ESI): $C_{13}H_{30}O_2SiNa$ $[M+Na]^+$ calculated: 269.1913, found: 269.1907;

R*f* = 0.3 (PE:MTBE 4:1, $KMnO_4$);

$[\alpha]_D^{20}$ = +3.5 (*c* 0.6, $CHCl_3$).

Analytical data are in accordance with the literature.[6]

2-4. Synthesis of TIB esters

General Procedure 3 (GP3): Mitsunobu Conditions

The required primary alcohol (1.1 equiv) was dissolved in anhydrous THF (0.3 M), PPh_3 (1.0 equiv) and TIBOH (1.0 equiv) were added successively. After cooling to 0 °C, DIAD (1.1 equiv, 0.12 mL/min) was added, the reaction mixture was slowly warmed to rt and stirred overnight at that temperature. MTBE and sat. aq. $NaHCO_3$ were added and the phases separated. The aqueous phase was extracted with MTBE (3x), the organic layers combined and dried over Na_2SO_4. The crude material was loaded on silica and purified by flash column chromatography.

TIB ester 9

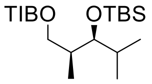

9
$C_{29}H_{52}O_3Si$
476.82

Using GP3 and primary alcohol **S1** (1.00 g, 4.06 mmol, 1.1 equiv) gave TIB ester **9** (1.49 g, 3.12 mmol, 85%) after purification by flash column chromatography (PE:MTBE 98:2) as a colorless oil.

^{1}H-NMR (400 MHz, $CDCl_3$): δ = 7.01 (s, 2H), 4.21 (m_c, 2H), 3.49 (dd, J = 5.6, 3.1 Hz, 1H), 2.95- 2.77 (m, 3H), 2.08 (m_c, 1H), 1.78 (m_c, 1H), 1.24 (m_c, 18H), 0.86 (d, J = 7.2 Hz, 3H), 0.93-0.85 (m, 15H), 0.06 (s, 3H), 0.04 (s, 3H) ppm;

^{13}C-NMR (101 MHz, $CDCl_3$): δ = 171.2, 150.1, 144.9, 130.8, 121.0, 77.2, 68.4, 36.5, 34.5, 32.1, 31.7, 26.2, 24.5, 24.3, 24.1, 20.1, 19.0, 18.6, 12.3, −3.7, −3.8 ppm;

HRMS (ESI): $C_{29}H_{52}O_3SiNa$ $[M+Na]^+$ calculated: 499.3583, found: 499.3589;

R_f = 0.3 (PE:MTBE 98:2, uv, vanillin);

$[\alpha]_D^{20}$ = +9.4 (c 0.7, $CHCl_3$).

TIB ester 11

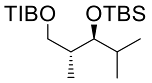

11
$C_{29}H_{52}O_3Si$
476.82

Using GP3 and primary alcohol **S2** (500 mg, 2.03 mmol, 1.1 equiv) gave TIB ester **11** (815 mg, 1.71 mmol, 92%) after purification by flash column chromatography (PE:MTBE 100:1) as a colorless oil.

^{1}H-NMR (400 MHz, $CDCl_3$): δ = 7.00 (s, 2H), 4.51 (dd, J = 10.8, 4.0 Hz, 1H), 4.85 (dd, J = 10.7, 8.3 Hz, 1H), 3.39 (t, J = 4.7 Hz, 1H), 2.96-2.78 (m_c, 3H), 2.11-2.00 (m, 1H), 1.88-

1.77 (m, 1H), 1.28-1.20 (m, 18H), 1.02 (d, *J* = 7.0 Hz, 3H), 0.95-0.86 (m, 15H), 0.09 (s, 3H), 0.06 (s, 3H) ppm;

^{13}C-NMR (101 MHz, $CDCl_3$): δ = 171.3, 150.1, 144.9, 131.0, 121.0, 79.4, 68.2, 36.9, 36.8, 34.5, 31.8, 31.7, 26.3, 24.4, 24.3, 24.1, 20.3, 20.3, 18.6, 17.9, 17.9, 15.8, −3.6, −3.8 ppm;

HRMS (ESI): $C_{29}H_{52}O_3SiNa$ $[M+Na]^+$ calculated: 499.3583, found: 499.3582;

R_f = 0.3 (PE:MTBE 98:2, uv, vanillin);

$[\alpha]_D^{20}$ = +7.4 (*c* 0.5, $CHCl_3$).

2-5. Synthesis of carbamates

General Procedure 4 (GP4): CbCl

The required primary alcohol (1.0 equiv) was dissolved in 1,2-dichloroethane (0.3 M) and Et_3N (3.0 equiv) and *N,N*-di*iso*propylcarbamoyl chloride (3.0 equiv) were added successively. After heating to 70 °C overnight H_2O was added. The phases were separated and the aqueous phase was extracted with CH_2Cl_2 (3x), the organic layers were combined and dried over Na_2SO_4. The solvent was removed *in vacuo* and the crude material was purified by flash column chromatography to afford the corresponding carbamate.

Carbamate 10

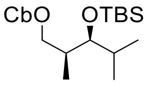

10
$C_{20}H_{43}NO_3Si$
373.65

Using GP4 and primary alcohol **S1** (1.06 g, 4.30 mmol, 1.0 equiv) gave carbamate **10** (1.55 g, 4.15 mmol, ≥ 95%) after purification by flash column chromatography (PE:MTBE 95:5) as a colorless oil.

^{1}H-NMR (400 MHz, $CDCl_3$): δ = 4.20-3.75 (m, 4H), 3.48 (dd, *J* = 5.9, 2.7 Hz, 1H), 2.01 (m_c, 1H), 1.77 (m_c, 1H), 1.24-1.18 (m, 12H), 0.89 (m, 18H), 0.05 (s, 3H), 0.04 (s, 3H) ppm;

^{13}C-NMR (101 MHz, $CDCl_3$): δ = 155.8, 77.1, 68.2, 45.9 (brs), 36.0, 32.5, 26.3, 21.4 (brs), 19.8, 19.3, 18.6, 11.9, −3.6, −3.8 ppm;

HRMS (ESI): $C_{20}H_{43}NO_3SiNa$ $[M+Na]^+$ calculated: 396.2910, found: 396.2899;

R*f* = 0.2 (PE:MTBE 95:5, vanillin);

$[\alpha]_D^{20}$ = +10.3 (*c* 0.5, $CHCl_3$).

Carbamate 12

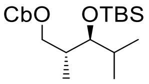

12
$C_{20}H_{43}NO_3Si$
373.65

Using GP4 and primary alcohol **S2** (400 mg, 1.62 mmol, 1.0 equiv) gave carbamate **12** (600 mg, 1.61 mmol, ≥ 95%) after purification by flash column chromatography (PE:MTBE 95:5) as a colorless oil.

^{1}H-NMR (400 MHz, $CDCl_3$): δ = 4.20 (dd, *J* = 10.7, 4.9 Hz, 1H), 4.15-3.61 (m, 3H), 3.41 (dd, *J* = 5.2, 4.3 Hz, 1H), 2.06-1.95 (m, 1H), 1.87-1.76 (m, 1H), 1.24-1.18 (m, 12H), 0.98 (t, *J* = 7.0 Hz, 3H), 0.93 (t, *J* = 6.9 Hz, 3H), 0.91 (s, 9H), 0.88 (t, *J* = 6.8 Hz, 3H), 0.05 (s, 3H), 0.04 (s, 3H) ppm;

^{13}C-NMR (101 MHz, $CDCl_3$): δ = 156.0, 79.0, 67.5, 45.8 (brs), 37.3, 31.4, 26.3, 21.2 (brs), 20.7, 18.6, 17.7, 15.5, −3.8, −3.8 ppm;

HRMS (ESI): $C_{20}H_{43}NO_3SiNa$ $[M+Na]^+$ calculated: 396.2910, found: 396.2907;

R_f = 0.3 (PE:MTBE 95:5, $KMnO_4$);

$[\alpha]_D^{20}$ = +10.7 (*c* 0.5, $CHCl_3$).

2-6. Substrate-Controlled 1,2-Metallate rearrangement of Vinyl Boronates

General Procedure 5 (GP5): TIB esters

To a stirred solution of TIB ester (1.5 equiv) and diamine (1.5 equiv) in Et_2O (0.2 M) at −78 °C was added *s*BuLi (1.3 M in hexanes, 1.4 equiv). The reaction mixture was stirred for 5 h at that temperature before a solution of vinyl boronic ester (1.0 equiv) in Et_2O (0.5 M) was added. After stirring for further 3 h at −78 °C, the reaction mixture was warmed to 45 °C and stirred overnight. The reaction mixture was cooled to rt, sat. aq. NH_4Cl was added and the biphasic mixture was stirred for 15 min. The phases were separated, the organic layer was washed with sat. aq. NH_4Cl (3x) and the combined aqueous phases were extracted with MTBE (3x). The combined organic phases were dried over Na_2SO_4, concentrated *in vacuo* and the crude material was purified by a short flash column chromatography (to remove TIBOH).

The residue was dissolved in THF (0.2 M) and cooled to −20 °C. A premixed, ice-cooled solution of NaOH (2.0 M)/H_2O_2 (35%, 2/1 v/v, 0.12 M) was added dropwise. The reaction mixture was stirred at rt before being diluted with MTBE and quenched by the slow addition of sat. aq. $Na_2S_2O_3$ at 0 °C after TLC showed full conversion. The solution was diluted with MTBE, the phases were separated and the aqueous phase was extracted with MTBE (3x). The combined organic layers were dried over Na_2SO_4 and concentrated *in vacuo*. The crude product was purified by flash column chromatography to afford allylic alcohol.

General Procedure 6 (GP6): Carbamates

To a stirred solution of carbamate (1.5 equiv) and diamine (1.5 equiv) in Et_2O (0.2 M) at −78 °C was added *s*BuLi (1.3 M in hexanes, 1.4 equiv). The reaction mixture was stirred for 5 h at that temperature before a solution of vinyl boronic ester (1.0 equiv) in Et_2O (0.5 M) was added. The reaction mixture was stirred for 3 h at −78 °C.

In parallel, magnesium turnings were activated (2x 1.0 M HCl, 2x H_2O, 2x acetone, drying under high vacuum). The required amount (2.0 equiv) was dissolved in Et_2O (0.8 M) and 1,2-dibromoethane (2.0 equiv) was added under water bath cooling. The reaction mixture was stirred for 2 h at this temperature.

The biphasic $MgBr_2 \cdot OEt_2$ solution was added dropwise to the main reaction mixture, which was then stirred for another 30 min at −78 °C before being warmed to 45 °C and stirred overnight. The reaction mixture was cooled to rt, sat. aq. NH_4Cl was added and the biphasic mixture was stirred for 15 min. The phases were separated, the organic layer was washed with sat. aq. NH_4Cl (3x) and the combined aqueous phases were extracted with MTBE (3x). The combined organic phases were dried over Na_2SO_4 and concentrated *in vacuo* and the crude material was purified by a short flash column chromatography (to remove excess of the carbamate).

The residue was dissolved in THF (0.2 M) and cooled to −20 °C. A premixed, ice-cooled solution of NaOH (2.0 M)/H_2O_2 (35%, 2/1 v/v, 0.12 M) was added dropwise. The reaction mixture was stirred at rt before being diluted with MTBE and quenched by the slow addition of sat. aq. $Na_2S_2O_3$ at 0 °C after TLC showed full conversion. The solution was diluted with MTBE, the phases were separated and the aqueous phase was extracted with MTBE (3x). The combined organic layers were dried over Na_2SO_4 and concentrated *in vacuo*. The crude product was purified by flash column chromatography to afford allylic alcohol.

(*R*)-Allylic alcohol 4

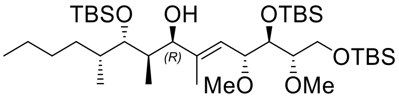

4

$C_{37}H_{80}O_6Si_3$

705.30

According to GP5, TIB ester **2a** (1.43 g, 2.75 mmol, 1.5 equiv),[4] vinyl boronic ester **3** (1.00 g, 1.84 mmol, 1.0 equiv)[4] and TMEDA gave (*R*)-allylic alcohol **4** (1.10 g, 1.56 mmol, 85% o2s, dr ≥ 19:1) after purification by flash column chromatography (PE:MTBE 95:5) as a colorless oil.

^{1}H-NMR (400 MHz, C_6D_6): δ = 5.96 (m_c, 1H), 4.58 (brs, 1H), 4.43 (dd, *J* = 9.8, 2.5 Hz, 1H), 4.26 (dd, *J* = 7.7, 2.5 Hz, 1H), 4.03 (dd, *J* = 11.2, 2.3 Hz, 1H), 3.90 (dd, *J* = 11.2, 5.0 Hz, 1H), 3.68 (m_c, 1H), 3.34 (s, 3H), 3.24 (m_c, 1H), 3.19 (s, 3H), 2.58 (d, *J* = 2.3 Hz, 1H), 1.92 (m_c, 1H), 1.78-1.70 (m, 4H), 1.58-1.49 (m, 1H), 1.36-1.17 (m, 5H), 1.10 (s, 9H), 1.05-0.97 (m, 24H), 0.93 (t, *J* = 7.0 Hz, 3H), 0.37 (s, 3H), 0.28 (s, 3H), 0.16 (s, 3H), 0.15 (s, 3H), 0.12 (s, 3H), 0.10 (s, 3H) ppm;

^{13}C-NMR (101 MHz, C_6D_6): δ = 142.0, 121.9, 83.4, 80.6, 78.3, 74.7, 74.6, 62.7, 57.9, 55.6, 39.0, 37.6, 34.4, 30.4, 26.52, 26.45, 26.2, 23.5, 18.8, 18.7, 18.6, 15.3, 14.8, 14.4, 11.6, −3.4, −3.6, −3.8, −4.6, −4.97, −5.04 ppm;

HRMS (ESI): $C_{37}H_{80}O_6Si_3Na$ $[M+Na]^+$ calculated: 727.5160, found: 727.5166;

R*f* = 0.3 (PE:MTBE 95:5, vanillin);

$[\boldsymbol{\alpha}]_{\mathbf{D}}^{\mathbf{20}}$ = −10.9 (*c* 0.7, $CHCl_3$).

Analytical data are in accordance with the literature.[4]

(*R*)-Allylic alcohol 13d

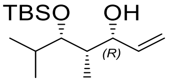

(*R*)-13d

$C_{15}H_{32}O_2Si$

272.50

According to GP5, TIB ester **9** (232 mg, 0.48 mmol, 1.5 equiv), vinyl boronic ester **8** (50 mg, 0.32 mmol, 1.0 equiv) and TMEDA gave allylic alcohol **(*R*)-13d** (43 mg, 0.16 mmol, 50% o2s, dr 2.1:1) after purification by flash column chromatography (PE:MTBE 95:5) as a colorless oil.
According to GP5, TIB ester **9** (232 mg, 0.48 mmol, 1.5 equiv), vinyl boronic ester **8** (50 mg, 0.32 mmol, 1.0 equiv) and (+)-sparteine gave allylic alcohol **(*R*)-13d** (63 mg, 0.23 mmol, 72% o2s, dr ≥ 19:1) after purification by flash column chromatography (PE:MTBE 95:5) as a colorless oil.
According to GP5, TIB ester **9** (232 mg, 0.48 mmol, 1.5 equiv), vinyl boronic ester **8** (50 mg, 0.32 mmol, 1.0 equiv) and (−)-sparteine gave allylic alcohol **(*R*)-13d** (13 mg, 0.05 mmol,

16% o2s, dr 3.2:1) after purification by flash column chromatography (PE:MTBE 95:5) as a colorless oil.
According to GP6, carbamate **10** (182 mg, 0.48 mmol, 1.5 equiv), vinyl boronic ester **8** (50 mg, 0.32 mmol, 1.0 equiv) and (+)-sparteine gave allylic alcohol **(*R*)-13d** (11 mg, 0.04 mmol, 13% o2s, dr 19:1) after purification by flash column chromatography (PE:MTBE 95:5) as a colorless oil.

Analytical data are given for allylic alcohol **(*R*)-13d** obtained by the reaction of TIB ester **9** and (+)-sparteine (dr ≥ 19:1).[1]

^{1}H-NMR (400 MHz, C_6D_6): δ = 5.74 (ddd, *J* = 17.4, 10.5, 5.7 Hz, 1H), 5.22 (dt, *J* = 17.2, 1.6 Hz, 1H), 5.03 (dt, *J* = 10.6, 1.6 Hz, 1H), 4.01 (brs, 1H), 3.59 (t, *J* = 4.1 Hz, 1H), 1.85-1.75 (m, 2H), 1.70-1.60 (m, 1H), 1.05 (d, *J* = 7.0 Hz, 3H), 1.01 (s, 9H), 0.91 (d, *J* = 6.9 Hz, 3H), 0.87 (d, *J* = 7.1 Hz, 3H), 0.10 (s, 3H), 0.07 (s, 3H) ppm;

^{13}C-NMR (101 MHz, C_6D_6): δ = 141.2, 114.6, 78.5, 75.0, 41.3, 33.3, 26.4, 19.4, 18.7, 18.2, 10.2, −3.4, −3.7 ppm;

HRMS (ESI): $C_{15}H_{32}O_2SiNa$ [M+Na]$^+$ calculated: 295.2069, found: 295.2077;

R*f* = 0.2 (PE:MTBE 9:1, vanillin);

$[\alpha]_D^{20}$ = +17.4 (*c* 0.8, $CHCl_3$).

(*S*)-Allylic alcohol 14d

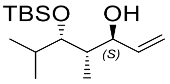

(*S*)-14d
$C_{15}H_{32}O_2Si$
272.50

According to GP6, carbamate **10** (182 mg, 0.48 mmol, 1.5 equiv), vinyl boronic ester **8** (50 mg, 0.32 mmol, 1.0 equiv) and TMEDA gave allylic alcohol **(*S*)-14d** (26 mg, 0.10 mmol, 31% o2s, dr 8.1:1) after purification by flash column chromatography (PE:MTBE 95:5) as a colorless oil.
According to GP6, carbamate **10** (182 mg, 0.48 mmol, 1.5 equiv), vinyl boronic ester **8** (50 mg, 0.32 mmol, 1.0 equiv) and (−)-sparteine gave allylic alcohol **(*S*)-14d** (10 mg, 0.04 mmol, 13% o2s, dr 19:1) after purification by flash column chromatography (PE:MTBE 95:5) as a colorless oil.

Analytical data are given for allylic alcohol **(*S*)-14d** obtained by the reaction of carbamate **10** and (−)-sparteine (dr 19:1).[2]

^{1}H-NMR (400 MHz, C_6D_6): δ = 5.71 (m_c, 1H), 5.15 (m_c, 1H), 4.99 (m_c, 1H), 3.91-3.85 (m, 2H), 1.78 (m_c, 1H), 1.69-1.61 (m, 2H), 1.02 (s, 9H), 0.99 (d, *J* = 6.7 Hz, 3H), 0.87-0.84 (m, 6H), 0.15 (s, 3H), 0.11 (s, 3H) ppm;

---

[1] Stereochemistry was assigned by usual induction of (+)-sparteine (matched-case TIB ester).[7, 8]
[2] Stereochemistry was assigned by usual induction of (−)-sparteine (matched-case carbamate).[7, 8]

^{13}**C-NMR** (101 MHz, C_6D_6): δ = 141.4, 115.5, 77.2, 75.5, 42.1, 32.6, 26.5, 20.1, 19.9, 18.7, 11.6, −3.75, −3.79 ppm;

HRMS (ESI): $C_{15}H_{32}O_2SiNa$ $[M+Na]^+$ calculated: 295.2066, found: 295.2069;

R*f* = 0.2 (PE:MTBE 95:5, vanillin);

$[\alpha]_D^{20}$ = −10.2 (*c* 0.9, $CHCl_3$).

(*R*)-Allylic alcohol 13c

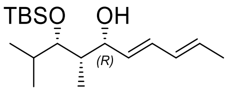

(*R*)-13c
$C_{18}H_{36}O_2Si$
312.57

According to GP5, TIB ester **9** (210 mg, 0.44 mmol, 1.5 equiv), vinyl boronic ester **7** (57 mg, 0.29 mmol, 1.0 equiv) and TMEDA gave allylic alcohol **(*R*)-13c** (41 mg, 0.13 mmol, 45% o2s, dr 13:1) after purification by flash column chromatography (PE:MTBE 95:5) as a colorless oil. According to GP6, carbamate **10** (165 mg, 0.44 mmol, 1.5 equiv), vinyl boronic ester **7** (57 mg, 0.29 mmol, 1.0 equiv) and TMEDA gave allylic alcohol **(*R*)-13c** (4 mg, 0.01 mmol, 3% o2s, dr 8:1) after purification by flash column chromatography (PE:MTBE 95:5) as a colorless oil.

Analytical data are given for allylic alcohol **(*R*)-13c** obtained by the reaction of TIB ester **9** and TMEDA (dr 13:1).

^{1}**H-NMR** (400 MHz, C_6D_6): δ = 6.30-6.23 (m, 1H), 6.08-6.01 (m, 1H), 5.59-5.50 (m, 2H), 4.08 (brs, 1H), 3.63 (t, *J* = 4.1 Hz, 1H), 1.86-1.78 (m, 1H), 1.74-1.68 (m, 1H), 1.59 (dd, *J* = 6.6, 1.3 Hz, 3H), 1.22-1.20 (m, 1H), 1.10 (d, *J* = 6.9 Hz, 3H), 1.02 (s, 9H), 0.92 (d, *J* = 6.9 Hz, 3H), 0.88 (d, *J* = 6.8 Hz, 3H), 0.13 (s, 3H), −0.08 (s, 3H) ppm;

^{13}**C-NMR** (101 MHz, C_6D_6): δ = 133.7, 131.7, 131.3, 129.0, 78.4, 74.9, 41.8, 33.5, 26.4, 19.3, 18.7, 18.3, 18.2, 10.5, −3.4, −3.7 ppm;

HRMS (ESI): $C_{18}H_{36}O_2SiNa$ $[M+Na]^+$ calculated: 335.2382, found: 335.2375;

R*f* = 0.3 (PE:MTBE 95:5, uv, vanillin);

$[\alpha]_D^{20}$ = +21.8 (*c* 1.0, $CHCl_3$).

(*S*)-Allylic alcohol 13b

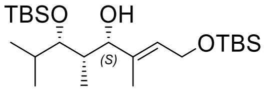

(*S*)-13b
$C_{23}H_{50}O_3Si_2$
430.82

According to GP5, TIB ester **9** (802 mg, 1.68 mmol, 1.5 equiv), vinyl boronic ester **6** (350 mg, 1.12 mmol, 1.0 equiv) and TMEDA gave allylic alcohol **(*S*)-13b** (379 mg, 0.88 mmol, 79% o2s, dr 10:1) after purification by flash column chromatography (PE:MTBE 9:1) as a colorless oil.

^{1}H-NMR (400 MHz, C_6D_6): δ = 5.80 (m_c, 1H), 4.26 (m_c, 2H), 3.87 (brs, 1H), 3.59 (t, J = 4.0 Hz, 1H), 1.85-1.77 (m, 2H), 1.46 (s, 3H), 1.18-1.16 (m, 1H), 1.08 (d, J = 6.9 Hz, 3H), 1.02 (s, 9H), 1.01 (s, 9H), 0.95 (d, J = 6.9 Hz, 3H), 0.89 (d, J = 6.9 Hz, 3H), 0.15 (s, 3H), 0.12 (s, 6H), 0.10 (s, 3H) ppm;

^{13}C-NMR (101 MHz, C_6D_6): δ = 138.0, 126.5, 78.6, 78.5, 60.1, 38.3, 33.4, 26.5, 26.2, 19.7, 18.8, 18.5, 18.1, 13.0, 10.1, −3.4, −3.5, −4.9, −5.0 ppm;

HRMS (ESI): $C_{23}H_{50}O_3Si_2Na$ $[M+Na]^+$ calculated: 453.3196, found: 453.3200;

R*f* = 0.3 (PE:MTBE 9:1, vanillin);

$[\boldsymbol{\alpha}]_{\mathbf{D}}^{\mathbf{20}}$ = +4.77 (*c* 1.0, $CHCl_3$).

(*R*)-Allylic alcohol 14b

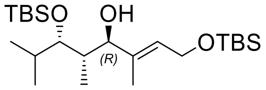

(*R*)-14b
$C_{23}H_{50}O_3Si_2$
430.82

According to GP6, carbamate **10** (165 mg, 0.44 mmol, 1.5 equiv), vinyl boronic ester **6** (91 mg, 0.29 mmol, 1.0 equiv) and TMEDA gave allylic alcohol **(*R*)-14b** (48 mg, 0.11 mmol, 38% o2s, dr 4.3:1) after purification by flash column chromatography (PE:MTBE 95:5) as a colorless oil.

^{1}H-NMR (400 MHz, C_6D_6): δ = 5.68 (m_c, 1H), 4.28-4.21 (m, 2H), 3.94-3.87 (m, 2H), 1.89-1.77 (m, 2H), 1.70 (brs, 1H), 1.57 (d, J = 1.2 Hz, 3H), 1.02-1.01 (m, 12H), 1.00 (s, 9H), 0.89 (d, J = 6.8 Hz, 3H), 0.79 (d, J = 7.1 Hz, 3H), 0.15 (s, 3H), 0.12-0.10 (m, 9H) ppm;

^{13}C-NMR (101 MHz, C_6D_6): δ = 138.0, 128.6, 79.8, 77.5, 60.1, 39.2, 32.6, 26.4, 26.2, 20.3, 20.2, 18.7, 18.5, 11.7, 10.8, −3.7, −3.8, −4.941, −4.945 ppm;

HRMS (ESI): $C_{23}H_{50}O_3Si_2Na$ $[M+Na]^+$ calculated: 453.3196, found: 453.3199;

R*f* = 0.3 (PE:MTBE 9:1, vanillin);

$[\alpha]_D^{20}$ = −8.63 (*c* 1.0, $CHCl_3$).

(*S*)-Allylic alcohol 13a

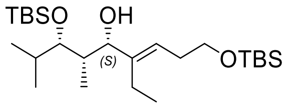

(*S*)-13a
$C_{25}H_{54}O_3Si_2$
458.87

According to GP5, TIB ester **9** (210 mg, 0.44 mmol, 1.5 equiv), vinyl boronic ester **5** (100 mg, 0.29 mmol, 1.0 equiv) and TMEDA gave allylic alcohol **(*S*)-13a** (119 mg, contaminated with 3% ketone, 0.25 mmol, 86% o2s, dr 25:1) after purification by flash column chromatography (PE:MTBE 95:5) as a colorless oil.

^{1}H-NMR (400 MHz, C_6D_6): δ = 5.58 (m_c, 1H), 4.09 (brs, 1H), 3.68-3.62 (m, 3H), 2.40-2.30 (m, 2H), 2.15-2.02 (m, 1H), 1.94-1.81 (m, 3H), 1.26 (d, *J* = 3.2 Hz, 1H), 1.07 (d, *J* = 7.0 Hz, 3H), 1.03 (s, 9H), 1.00-0.96 (m, 15H), 0.93 (d, *J* = 6.8 Hz, 3H), 0.16 (s, 3H), 0.10 (s, 3H), 0.09 (s, 6H) ppm;

^{13}C-NMR (101 MHz, C_6D_6): δ = 144.5, 121.7, 79.6, 76.9, 63.5, 38.8, 33.1, 31.8, 26.5, 26.2, 21.8, 19.9, 18.8, 18.6, 17.8, 14.4, 9.8, −3.3, −3.6, −5.097, −5.105 ppm;

HRMS (ESI): $C_{25}H_{54}O_3Si_2Na$ $[M+Na]^+$ calculated: 481.3509, found: 481.3514;

R_f = 0.3 (PE:MTBE 95:5, vanillin);

$[\alpha]_D^{20}$ = +9.66 (*c* 1.1, $CHCl_3$).

(*R*)-Allylic alcohol 14a

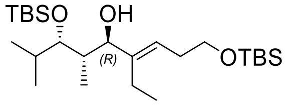

(*R*)-14a
$C_{25}H_{54}O_3Si_2$
458.87

According to GP6, carbamate **10** (165 mg, 0.44 mmol, 1.5 equiv), vinyl boronic ester **5** (100 mg, 0.29 mmol, 1.0 equiv) and TMEDA gave allylic alcohol **(*R*)-14a** (57 mg, contaminated with 5% ketone, 0.12 mmol, 41% o2s, dr 4.2:1) after purification by flash column chromatography (PE:MTBE 95:5) as a colorless oil.

^{1}H-NMR (400 MHz, C_6D_6): δ = 5.45 (m_c, 1H), 3.99-3.95 (m, 2H), 3.59 (t, *J* = 6.7 Hz, 2H), 2.31 (q, *J* = 6.8 Hz, 2H), 2.16-2.05 (m, 2H), 1.94-1.82 (m, 2H), 1.66 (brs, 1H), 1.10 (t, *J* = 7.6 Hz, 3H), 1.05-1.03 (m, 12H), 1.00 (s, 9H), 0.92 (d, *J* = 6.9 Hz, 3H), 0.85 (d, *J* = 7.0 Hz, 3H), 0.21 (s, 3H), 0.14 (s, 3H), 0.08 (s, 6H) ppm;

^{13}C-NMR (101 MHz, C_6D_6): δ = 144.8, 125.3, 80.1, 77.5, 63.2, 39.9, 32.7, 31.7, 26.5, 26.2, 20.34, 20.32, 20.29, 18.8, 18.5, 15.3, 12.0, −3.71, −3.74, −5.13, −5.14 ppm;

HRMS (ESI): $C_{25}H_{54}O_3Si_2Na$ [M+Na]$^+$ calculated: 481.3509, found: 481.3506;

R*f* = 0.3 (PE:MTBE 95:5, vanillin);

$[\alpha]_D^{20}$ = −3.80 (*c* 1.0, $CHCl_3$).

(*S*)-Allylic alcohol 15d

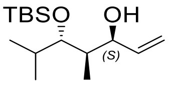

(*S*)-15d
$C_{15}H_{32}O_2Si$
272.50

According to GP5, TIB ester **11** (143 mg, 0.30 mmol, 1.5 equiv), vinyl boronic ester **8** (31 mg, 0.20 mmol, 1.0 equiv) and TMEDA gave allylic alcohol **(*S*)-15d** (27 mg, 0.10 mmol, 50% o2s, dr 2.2:1) after purification by flash column chromatography (PE:MTBE 95:5) as a colorless oil. According to GP6, carbamate **12** (182 mg, 0.48 mmol, 1.5 equiv), vinyl boronic ester **8** (50 mg, 0.32 mmol, 1.0 equiv) and (−)-sparteine gave allylic alcohol **(*S*)-15d** (15 mg, 0.06 mmol, 19% o2s, dr 19:1) after purification by flash column chromatography (PE:MTBE 95:5) as a colorless oil.

Analytical data are given for allylic alcohol **(*S*)-15d** obtained by the reaction of carbamate **12** and (−)-sparteine (dr ≥ 19:1).[3]

^{1}H-NMR (400 MHz, C_6D_6): δ = 5.77 (m_c, 1H), 5.44 (dt, *J* = 17.3, 1.9 Hz, 1H), 5.12 (dt, *J* = 10.6, 1.9 Hz, 1H), 4.68-4.64 (m, 1H), 3.40 (t, *J* = 4.9 Hz, 1H), 1.84-1.73 (m, 1H), 1.65-1.57 (m, 1H), 0.97 (s, 9H), 0.94 (d, *J* = 7.1 Hz, 3H), 0.87 (d, *J* = 7.1 Hz, 3H), 0.82 (d, *J* = 6.9 Hz, 3H), 0.09 (s, 3H), 0.04 (s, 3H) ppm;

^{13}C-NMR (101 MHz, C_6D_6): δ = 141.2, 113.7, 81.9, 71.4, 40.4, 32.1, 26.4, 20.2, 18.7, 18.5, 11.5, −3.56, −3.64 ppm;

HRMS (ESI): $C_{15}H_{32}O_2SiNa$ [M+Na]$^+$ calculated: 295.2069, found: 295.2077;

R*f* = 0.2 (PE:MTBE 95:5, vanillin);

$[\alpha]_D^{20}$ = −3.0 (*c* 0.8, $CHCl_3$).

---

[3] Stereochemistry was assigned by usual induction of (−)-sparteine (matched-case carbamate).[7, 8]

(*R*)-Allylic alcohol 16d

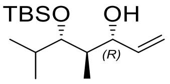

(*R*)-16d
$C_{15}H_{32}O_2Si$
272.50

According to GP6, carbamate **12** (109 mg, 0.29 mmol, 1.5 equiv), vinyl boronic ester **8** (30 mg, 0.19 mmol, 1.0 equiv) and TMEDA gave allylic alcohol **(*S*)-15d** (1.5 mg) and allylic alcohol **(*R*)-16d** (3.5 mg, 5.0 mg in total, 0.02 mmol, 11% o2s, dr 2.3:1) after purification by flash column chromatography (PE:MTBE 95:5) as colorless oils.

Analytical data are given for the pure allylic alcohol **(*R*)-16d**.

^{1}H-NMR (400 MHz, C_6D_6): δ = 5.84 (m_c, 1H), 5.27 (ddd, *J* = 17.1, 10.5, 4.1 Hz, 1H), 5.06 (m_c, 1H), 4.04 (t, *J* = 7.2 Hz, 1H), 3.57 (dd, *J* = 5.3, 4.2 Hz, 1H), 2.22 (brs, 1H), 1.88-1.78 (m, 2H), 0.98 (s, 9H), 0.95 (d, *J* = 6.7 Hz, 3H), 0.93 (d, *J* = 6.5 Hz, 3H), 0.83 (d, *J* = 5.9 Hz, 3H), 0.09 (s, 3H), 0.05 (s, 3H) ppm;

^{13}C-NMR (101 MHz, C_6D_6): δ = 140.5, 115.1, 80.4, 75.3, 43.5, 32.4, 26.3, 20.5, 18.5, 18.2, 14.4, −3.8, −4.1 ppm;

HRMS (ESI): $C_{15}H_{32}O_2SiNa$ $[M+Na]^+$ calculated: 295.2069, found: 295.2066;

R*f* = 0.2 (PE:MTBE 95:5, vanillin);

$[\alpha]_D^{20}$ = +8.3 (*c* 0.8, $CHCl_3$).

(*S*)-Allylic alcohol 15c

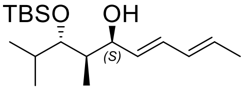

(*S*)-15c
$C_{18}H_{36}O_2Si$
312.57

According to GP5, TIB ester **11** (210 mg, 0.44 mmol, 1.5 equiv), vinyl boronic ester **7** (57 mg, 0.29 mmol, 1.0 equiv) and TMEDA gave allylic alcohol **(*S*)-15c** (62 mg, 0.20 mmol, 69% o2s, dr 4.8:1) after purification by flash column chromatography (PE:MTBE 98:2 → 10:1) as a colorless oil.

^{1}H-NMR (400 MHz, C_6D_6): δ = 6.48 (m_c, 1H), 6.14-6.07 (m, 1H), 5.59-5.54 (m, 2H), 4.73 (brs, 1H), 3.43 (t, *J* = 4.9 Hz, 1H), 2.56 (d, *J* = 2.3 Hz, 1H), 1.86-1.77 (m, 1H), 1.69-1.58 (m, 4H), 0.99-0.97 (m, 12H), 0.89 (d, *J* = 6.8 Hz, 3H), 0.85 (d, *J* = 6.9 Hz, 3H), 0.11 (s, 3H), 0.05 (s, 3H) ppm;

^{13}C-NMR (101 MHz, C_6D_6): δ = 134.0, 132.0, 130.1, 128.3, 81.9, 71.1, 41.1, 32.1, 26.4, 20.2, 18.7, 18.5, 18.2, 11.8, −3.55, −3.62 ppm;

HRMS (ESI): $C_{18}H_{36}O_2SiNa$ $[M+Na]^+$ calculated: 335.2382, found: 335.2387;

R$_f$ = 0.3 (PE:MTBE 95:5, uv, vanillin);

$[\alpha]_D^{20}$ = −0.92 (c 0.8, $CHCl_3$).

(*R*)-Allylic alcohol 16c

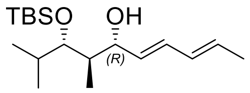

(*R*)-16c
$C_{18}H_{36}O_2Si$
312.57

According to GP6, carbamate **12** (165 mg, 0.44 mmol, 1.5 equiv), vinyl boronic ester **7** (57 mg, 0.29 mmol, 1.0 equiv) and TMEDA gave allylic alcohol **(*R*)-16c** (35 mg, 0.11 mmol, 38% o2s, dr 2:1) after purification by flash column chromatogra-phy (PE:MTBE 95:5) as a colorless oil.

^{1}H-NMR (400 MHz, C_6D_6): δ = 6.30 (dd, J = 15.1, 10.4 Hz, 1H), 6.10-6.03 (m, 1H), 5.63-5.52 (m, 2H), 4.09 (t, J = 7.6 Hz, 1H), 3.64 (dd, J = 5.0, 4.3 Hz, 1H), 2.21 (s, 1H), 1.92-1.82 (m, 2H) 1.59 (dd, J = 6.6, 1.4 Hz, 3H), 1.00 (s, 9H), 0.99-0.97 (m, 3H), 0.95 (d, J = 6.8 Hz, 3H), 0.86 (d, J = 7.1 Hz, 3H), 0.13 (s, 3H), 0.07 (s, 3H) ppm;

^{13}C-NMR (101 MHz, C_6D_6): δ = 133.1, 131.9, 131.6, 129.0, 80.2, 75.0, 44.1, 32.3, 26.3, 20.6, 18.6, 18.3, 18.2, 14.4, −3.8, −4.1 ppm;

HRMS (ESI): $C_{18}H_{36}O_2SiNa$ $[M+Na]^+$ calculated: 335.2382, found: 335.2387;

R$_f$ = 0.3 (PE:MTBE 95:5, uv, vanillin);

$[\alpha]_D^{31}$ = +8.7 (c 1.0, $CHCl_3$).

(*R*)-Allylic alcohol 15b

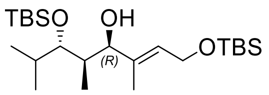

(*R*)-15b
$C_{23}H_{50}O_3Si_2$
430.82

According to GP5, TIB ester **11** (802 mg, 1.68 mmol, 1.5 equiv), vinyl boronic ester **6** (350 mg, 1.12 mmol, 1.0 equiv) and TMEDA gave allylic alcohol **(*R*)-15b** (333 mg, 0.77 mmol, 69% o2s, dr ≥ 19:1) after purification by flash column chromatogra-phy (PE:MTBE 98:2 → 9:1) as a colorless oil.

^{1}H-NMR (400 MHz, C_6D_6): δ = 6.08 (m_c, 1H), 4.43 (brs, 1H), 4.34 (m_c, 2H), 3.43 (m_c, 1H), 2.75 (d, J = 2.0 Hz, 1H), 1.87-1.75 (m, 2H), 1.51 (s, 3H), 1.01 (s, 9H), 0.98 (s, 9H), 0.94 (d, J = 7.1 Hz, 3H), 0.91 (d, J = 6.8 Hz, 3H), 0.86 (d, J = 6.9 Hz, 3H), 0.13 (s, 6H), 0.07 (s, 3H), 0.04 (s, 3H) ppm;

^{13}C-NMR (101 MHz, C_6D_6): δ = 137.3, 124.8, 82.1, 74.1, 60.4, 37.9, 32.1, 26.4, 26.2, 20.3, 18.69, 18.65, 18.6, 14.1, 11.4, −3.5, −3.6, −4.85, −4.88 ppm;

HRMS (ESI): $C_{23}H_{50}O_3Si_2Na$ $[M+Na]^+$ calculated: 453.3196, found: 453.3198;

$\mathbf{R}_f$ = 0.3 (PE:MTBE 9:1, vanillin);

$[\alpha]_D^{20}$ = +10.1 (*c* 1.3, $CHCl_3$).

(*S*)-Allylic alcohol 16b

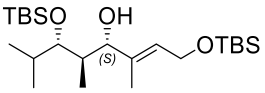

(*S*)-16b
$C_{23}H_{50}O_3Si_2$
430.82

According to GP6, carbamate **12** (165 mg, 0.44 mmol, 1.5 equiv), vinyl boronic ester **6** (91 mg, 0.29 mmol, 1.0 equiv) and TMEDA gave allylic alcohol **(*S*)-16b** (76 mg, 0.18 mmol, 60% o2s, dr 2.3:1) after purification by flash column chromatography (PE:MTBE 20:1) as a colorless oil.

^{1}H-NMR (400 MHz, C_6D_6): δ = 5.68-5.66 (m, 1H), 4.26-4.24 (m, 2H), 3.90 (d, *J* = 9.6 Hz, 1H), 3.69 (t, *J* = 4.4 Hz, 1H), 2.36 (d, *J* = 1.5 Hz, 1H), 2.01-1.92 (m, 1H), 1.90-1.82 (m 1H), 1.65 (d, *J* = 1.1 Hz, 3H), 1.01 (s, 9H), 0.99-0.96 (m, 6H), 0.98 (s, 9H), 0.80 (d, *J* = 7.2 Hz, 3H), 0.13 (s, 3H), 0.11 (s, 3H), 0.10 (s, 3H), 0.06 (s, 3H) ppm;

^{13}C-NMR (101 MHz, C_6D_6): δ = 137.6, 128.5, 81.0, 80.3, 60.1, 40.7, 32.4, 26.2, 26.2, 20.7, 18.6, 18.5, 18.5, 15.0, 11.0, −3.9, −4.2, −4.9, −5.0 ppm;

HRMS (ESI): $C_{23}H_{50}O_3Si_2Na$ $[M+Na]^+$ calculated: 453.3196, found: 453.3198;

$\mathbf{R}_f$ = 0.3 (PE:MTBE 9:1, vanillin);

$[\alpha]_D^{31}$ = +3.0 (*c* 1.0, $CHCl_3$).

(*R*)-Allylic alcohol 15a

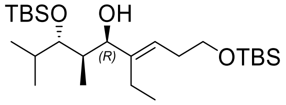

(*R*)-15a
$C_{25}H_{54}O_3Si_2$
458.87

According to GP5, TIB ester **11** (210 mg, 0.44 mmol, 1.5 equiv), vinyl boronic ester **5** (100 mg, 0.29 mmol, 1.0 equiv) and TMEDA gave allylic alcohol **(*R*)-15a** (107 mg, 0.23 mmol, 79% o2s, dr ≥ 19:1) after purification by flash column chromatography (PE:MTBE 98:2 → 9:1) as a colorless oil.

^{1}H-NMR (400 MHz, C_6D_6): δ = 5.76 (m_c, 1H), 4.62 (brs, 1H), 3.66 (t, *J* = 7.0 Hz, 2H), 3.51 (t, *J* = 5.1 Hz, 1H), 2.54 (d, *J* = 2.0 Hz, 1H), 2.48-2.35 (m, 2H), 2.16 (m_c, 1H), 1.91-1.77 (m, 3H), 1.06-0.99 (m, 21H), 0.96 (d, *J* = 7.1 Hz, 3H), 0.93 (d, *J* = 6.8 Hz, 3H), 0.90 (d, *J* = 7.0 Hz, 3H), 0.12 (s, 3H), 0.09 (s, 6H), 0.07 (s, 3H) ppm;

^{13}**C-NMR** (101 MHz, C_6D_6): δ = 143.8, 120.6, 81.9, 72.2, 63.6, 38.1, 32.1, 31.8, 26.5, 26.2, 22.1, 20.4, 18.7, 18.6, 18.3, 14.2, 11.3, −3.46, −3.55, −5.1 ppm;

HRMS (ESI): $C_{25}H_{54}O_3Si_2Na$ $[M+Na]^+$ calculated: 481.3509, found: 481.3511;

$\mathbf{R}_f$ = 0.3 (PE:MTBE 95:5, vanillin);

$[\alpha]_D^{20}$ = +3.19 (*c* 1.2, $CHCl_3$).

(*S*)-Allylic alcohol 16a

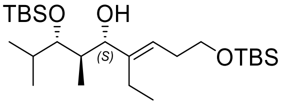

(*S*)-16a
$C_{25}H_{54}O_3Si_2$
458.87

According to GP6, carbamate **12** (165 mg, 0.44 mmol, 1.5 equiv), vinyl boronic ester **5** (100 mg, 0.29 mmol, 1.0 equiv) and TMEDA gave allylic alcohol **(*S*)-16a** (71 mg, 0.15 mmol, 52% o2s, dr 2.4:1) after purification by flash column chromatography (PE:MTBE 20:1) as a colorless oil.

^{1}**H-NMR** (400 MHz, C_6D_6): δ = 5.43 (t, *J* = 7.2 Hz, 1H), 3.98 (d, *J* = 9.8 Hz, 1H), 3.71 (t, *J* = 4.4 Hz, 1H), 3.61 (t, *J* = 6.7 Hz, 2H), 2.49 (s, 1H), 2.34 (q, *J* = 6.7 Hz, 2H), 2.29-2.11 (m, 2H), 2.07-1.98 (m, 1H), 1.94-1.84 (m, 1H), 1.19 (t, *J* = 7.6 Hz, 3H), 1.01-0.98 (m, 6H), 1.00 (s, 9H), 0.99 (s, 9H), 0.85 (d, *J* = 7.1 Hz, 3H), 0.16 (s, 3H), 0.09 (s, 6H), 0.07 (s, 3H) ppm;

^{13}**C-NMR** (101 MHz, C_6D_6): δ = 144.3, 125.1, 81.7, 80.8, 63.3, 41.3, 32.8, 31.7, 26.3, 26.2, 20.6, 20.4, 18.7, 18.6, 18.5, 15.8, 15.3, −3.9, −4.2, −5.1 ppm;

HRMS (ESI): $C_{25}H_{54}O_3Si_2Na$ $[M+Na]^+$ calculated: 481.3509, found: 481.3511;

$\mathbf{R}_f$ = 0.3 (PE:MTBE 95:5, vanillin);

$[\alpha]_D^{31}$ = ±0 (*c* 1.0, $CHCl_3$).

2-7. Analysis of Stereochemistry

General Procedure 7 (GP7): Mosher ester[4]

To a stirred solution of allylic alcohol (1.0 equiv) in CH_2Cl_2 (0.02 M) were added Et_3N (10.0 equiv), DMAP (6.0 equiv) and MTPACl (6.0 equiv) successively. The reaction mixture was stirred at rt until TLC showed full conversion. After the addition of MTBE and aq. NaOH (2.0 M) the organic layer was separated. The organic layer was washed with aq. NaOH (2.0 M, 3x), sat. aq. $NaHCO_3$ (3x), aq. $CuSO_4$ (1.0 M) and sat. aq. NaCl, dried over Na_2SO_4 and concentrated *in vacuo*.

(*S*)-Mosher ester S3

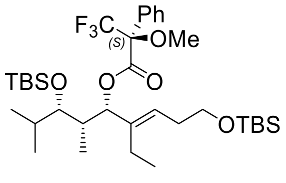

S3
$C_{35}H_{61}F_3O_5Si_2$
675.03

Following GP7, allylic alcohol **(*S*)-13a** (12 mg, 26.2 µmol, 1.0 equiv) and (*R*)-MTPACl gave (*S*)-mosher ester **S3** (16 mg, 23.7 µmol, 90%) as a yellow oil.

^{1}H-NMR (400 MHz, C_6D_6): δ = 7.72-7.70 (m, 2H), 7.14-7.06 (m, 3H), 5.76 (d, J = 6.2 Hz, 1H), 5.68 (m_c, 1H), 3.57 (m_c, 2H), 3.49 (d, J = 0.9 Hz, 3H), 3.44 (m_c, 1H), 2.27 (m_c, 2H), 2.11-2.05 (m, 1H), 2.00 (m_c, 1H), 1.91 (m_c, 1H), 1.86-1.80 (m, 1H), 1.06 (m_c, 6H), 1.03 (s, 9H), 0.98 (s, 9H), 0.88 (d, J = 6.9 Hz, 6H), 0.17 (s, 3H), 0.08 (s, 3H), 0.07 (s, 6H) ppm.

(*R*)-Mosher ester S4

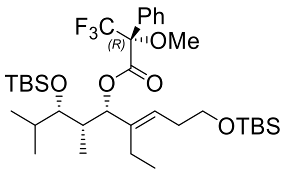

S4
$C_{35}H_{61}F_3O_5Si_2$
675.03

Following GP7, allylic alcohol **(*S*)-13a** (12 mg, 26.2 µmol, 1.0 equiv) and (*S*)-MTPACl gave (*R*)-mosher ester **S4** (17 mg, 25.2 µmol, ≥ 95%) as a yellow oil.

^{1}H-NMR (400 MHz, C_6D_6): δ = 7.70-7.68 (m, 2H), 7.15-7.10 (m, 2H), 7.08-7.04 (m, 1H), 5.70 (d, J = 5.8 Hz, 1H), 5.55 (m_c, 1H), 3.56-3.52 (m, 3H), 3.48 (d, J = 1.0 Hz, 3H), 2.24 (m_c, 2H), 2.02 (m_c, 2H), 1.89-1.77 (m, 2H), 1.16 (d, J = 6.8 Hz, 3H), 1.03 (s, 9H), 0.98 (s, 9H), 0.96 (t, J = 7.5 Hz, 3H), 0.92 (d, J = 7.0 Hz, 3H), 0.90 (d, J = 6.8 Hz, 3H), 0.18 (s, 3H), 0.10 (s, 3H), 0.071 (s, 3H), 0.066 (s, 3H) ppm.

[4] Unless otherwise noted, the stereochemistry of at least one result (per stereo-motif) of the branched vinyl boronic ester(s) was determined via Mosher-analysis. The stereochemistry of the other results of the same motif were assigned in analogy to the one determined via Mosher-analysis.

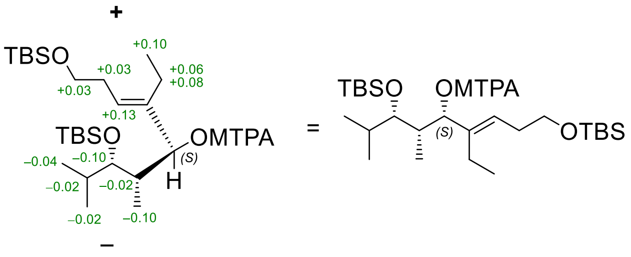

Figure 1. Mosher-analysis of allylic alcohol **(*S*)-13a**.

(*S*)-Mosher ester S5

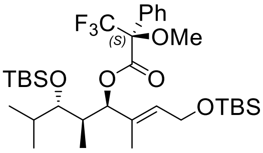

S5

$C_{33}H_{57}F_3O_5Si_2$

646.98

Following GP7, allylic alcohol **(*R*)-15b** (10 mg, 23.2 µmol, 1.0 equiv) and (*R*)-MTPACl gave (*S*)-mosher ester **S5** (14 mg, 21.6 µmol, 93%) as a yellow oil.

^{1}H-NMR (400 MHz, C_6D_6): δ = 7.74-7.72 (m, 2H), 7.14-7.12 (m, 2H), 7.09-7.05 (m, 1H), 5.75 (m_c, 1H), 5.59 (d, J = 6.7 Hz, 1H), 4.12 (m_c, 2H), 3.49 (m_c, 4H), 2.06 (m_c, 1H), 1.75 (m_c, 1H), 1.50 (d, J = 0.9 Hz, 3H), 1.06 (d, J = 7.0 Hz, 3H), 0.99 (s, 9H), 0.97 (s, 9H), 0.94 (d, J = 7.0 Hz, 3H), 0.92 (d, J = 6.7 Hz, 3H), 0.08 (s, 3H), 0.07 (s, 3H), 0.06 (s, 6H) ppm.

(*R*)-Mosher ester S6

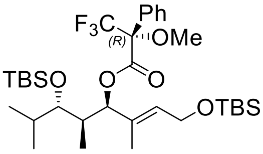

S6

$C_{33}H_{57}F_3O_5Si_2$

646.98

Following GP7, allylic alcohol **(*R*)-15b** (10 mg, 23.2 µmol, 1.0 equiv) and (*S*)-MTPACl gave (*R*)-mosher ester **S6** (13 mg, 20.1 µmol, 87%) as a yellow oil.

^{1}H-NMR (400 MHz, C_6D_6): δ = 7.74-7.72 (m, 2H), 7.13-7.06 (m, 3H), 5.83 (m_c, 1H), 5.65 (d, J = 7.2 Hz, 1H), 4.12 (m_c, 2H), 3.49 (d, J = 1.0 Hz, 3H), 3.45 (m_c, 1H), 2.06 (m_c, 1H), 1.73 (m_c, 1H), 1.63 (s, 3H), 0.99 (s, 9H), 0.97 (s, 9H), 0.95 (d, J = 7.1 Hz, 3H), 0.92 (d, J = 6.4 Hz, 3H), 0.91 (d, J = 6.5 Hz, 3H), 0.11 (s, 3H), 0.08 (s, 3H), 0.06 (s, 6H) ppm.

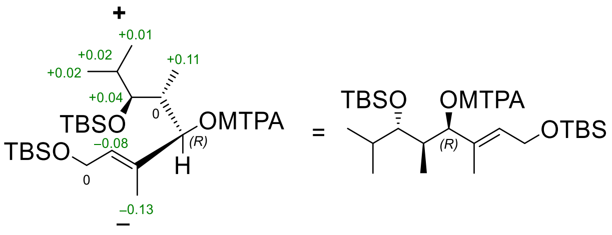

Figure 2. Mosher-analysis of allylic alcohol ***(R)*-15b**.

2-8. Rationalization of substrate control

General Procedure 8 (GP8): Stannanes

The required TIB ester or carbamate (1.0 equiv) and diamine (1.5 equiv) were dissolved in Et_2O (0.2 M). The solution was cooled to −78 °C, *s*BuLi (1.3 M in hexanes, 1.5 equiv) was added and the reaction mixture was stirred for 5 h at this temperature. Then a freshly prepared solution of Me_3SnCl (1.7 equiv) in Et_2O (1.0 M) was added dropwise and the reaction mixture was stirred for 1 h at −78 °C before being warmed to rt. After stirring overnight at rt sat. aq. NH_4Cl was added and the biphasic mixture was stirred for further 15 min. The phases were separated, the organic layer was washed with sat. aq. NH_4Cl (3x) and the combined aqueous phases were extracted with MTBE (3x). The combined organic phases were dried over Na_2SO_4 and concentrated *in vacuo* and the crude material was purified by flash column chromatography.

General Procedure 9 (GP9): Transmetalation of TIB-derived stannanes

To a stirred solution of stannane (1.5 equiv) in Et_2O (0.2 M) at −78 °C was added *n*BuLi (2.5 M in hexanes, 1.65 equiv). The reaction mixture was stirred for 1 h at that temperature before TMEDA (1.5 equiv) was added. Stirring was continued at −78 °C for 20 min before a solution of vinyl boronic ester (1.0 equiv) in Et_2O (0.5 M) was added. After stirring for further 3 h at −78 °C, the reaction mixture was warmed to 45 °C and stirred overnight. The reaction mixture was cooled to rt, sat. aq. NH_4Cl was added and the biphasic mixture was stirred for 15 min. The phases were separated, the organic layer was washed with sat. aq. NH_4Cl (3x) and the combined aqueous phases were extracted with MTBE (3x). The combined organic phases were dried over Na_2SO_4, concentrated *in vacuo* and the crude material was purified by a short flash column chromatography (to remove TIBOH).

The residue was dissolved in THF (0.2 M) and cooled to −20 °C. A premixed, ice-cooled solution of NaOH (2.0 M)/H_2O_2 (35%, 2/1 v/v, 0.12 M) was added dropwise. The reaction mixture was stirred at rt before being diluted with MTBE and quenched by the slow addition of sat. aq. $Na_2S_2O_3$ at 0 °C after TLC showed full conversion. The solution was diluted with MTBE, the phases were separated and the aqueous phase was extracted with MTBE (3x). The combined organic layers were dried over Na_2SO_4 and concentrated *in vacuo*. The crude product was purified by flash column chromatography to afford allylic alcohol.

General Procedure 10 (GP10): Transmetalation of Cb-derived stannanes

To a stirred solution of stannane (1.5 equiv) in Et_2O (0.2 M) at −78 °C was added *n*BuLi (2.5 M in hexanes, 1.65 equiv). The reaction mixture was stirred for 1 h at that temperature before TMEDA (1.5 equiv) was added. Stirring was continued at −78 °C for 20 min before a solution of vinyl boronic ester (1.0 equiv) in Et_2O (0.5 M) was added. The reaction mixture was stirred for 3 h at −78 °C.

In parallel, magnesium turnings were activated (2x 1.0 M HCl, 2x H_2O, 2x acetone, drying under high vacuum). The required amount (2.0 equiv) was dissolved in Et_2O (0.8 M) and 1,2-dibromoethane (2.0 equiv) was added under water bath cooling. The reaction mixture was stirred for 2 h at this temperature.

The biphasic $MgBr_2{\cdot}OEt_2$ solution was added dropwise to the main reaction mixture, which was then stirred for another 30 min at −78 °C before being warmed to 45 °C and stirred overnight. The reaction mixture was cooled to rt, sat. aq. NH_4Cl was added and the biphasic mixture was stirred for 15 min. The phases were separated, the organic layer was washed with sat. aq. NH_4Cl (3x) and the combined aqueous phases were extracted with MTBE (3x). The combined organic phases were dried over Na_2SO_4 and concentrated *in vacuo* and the crude material was purified by a short flash column chromatography (to remove excess of the carbamate).

The residue was dissolved in THF (0.2 M) and cooled to −20 °C. A premixed, ice-cooled solution of NaOH (2.0 M)/H_2O_2 (35%, 2/1 v/v, 0.12 M) was added dropwise. The reaction mixture was stirred at rt before being diluted with MTBE and quenched by the slow addition of sat. aq. $Na_2S_2O_3$ at 0 °C after TLC showed full conversion. The solution was diluted with MTBE, the phases were separated and the aqueous phase was extracted with MTBE (3x). The combined organic layers were dried over Na_2SO_4 and concentrated *in vacuo*. The crude product was purified by flash column chromatography to afford allylic alcohol.

Stannane 17

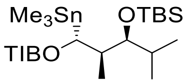

17
$C_{32}H_{60}O_3SiSn$
639.62

According to GP8, TIB ester **9** (250 mg, 0.52 mmol, 1.0 equiv) and (+)-sparteine (0.18 mL, 0.79 mmol, 1.5 equiv) gave stannane (+)-sp-**17** (260 mg, 0.41 mmol, 79%, dr ≥ 19:1) after purification by flash column chromatography (PE:MTBE 200:1 → 150:1) as a colorless oil. According to GP8, TIB ester **9** (158 mg, 0.33 mmol, 1.0 equiv) and TMEDA (0.08 mL, 0.50 mmol, 1.5 equiv) gave stannane TMEDA-**17** (173 mg, 0.27 mmol, 82%, dr 1.4:1) after purification by flash column chromatogra-phy (PE:MTBE 200:1 → 150:1) as a colorless oil.

Analytical data are given for stannane (+)-sp-**17** obtained by the reaction of TIB ester **9** and (+)-sparteine (dr ≥ 19:1).

^{1}H-NMR (400 MHz, C_6D_6): δ = 7.08 (s, 2H), 5.16 (m_c, 1H), 3.71 (dd, J = 8.2, 2.2 Hz, 1H), 3.12 (sep, J = 6.8 Hz, 2H), 2.72 (sep, J = 6.9 Hz, 1H), 2.20 (m_c, 1H), 2.10 (m_c, 1H), 1.34-1.29 (m, 15H), 1.16 (d, J = 6.9 Hz, 6H), 1.01-0.98 (m, 12H), 0.94 (d, J = 6.7 Hz, 3H), 0.36 (m_c, 9H), 0.02 (s, 3H), 0.01 (s, 3H) ppm;

^{13}C-NMR (101 MHz, C_6D_6): δ = 171.8, 150.4, 145.7, 131.4, 121.2, 79.4, 75.6, 41.3, 34.8, 32.1, 31.2, 26.5, 24.7, 24.6, 24.1, 21.5, 18.8, 16.3, 15.7, −3.1, −3.3, −7.5 ppm;

HRMS (ESI): $C_{32}H_{60}O_3SiSnNa$ $[M+Na]^+$ calculated: 663.3231, found: 663.3231;

R_f = 0.4 (PE:MTBE 98:2, uv, vanillin);

$[\alpha]_D^{20}$ = −39.9 (c 0.7, $CHCl_3$).

Stannane 21

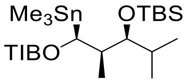

21
$C_{32}H_{60}O_3SiSn$
639.62

According to GP8, TIB ester **9** (175 mg, 0.37 mmol, 1.0 equiv) and (−)-sparteine (0.13 mL, 0.55 mmol, 1.5 equiv) gave stannane (−)-sp-**21** (198 mg, contaminated with 51% TIB ester, 0.15 mmol, 41%, dr 4:1) after purification by flash column chromatography (PE:MTBE 200:1 → 150:1) as a colorless oil.

^{1}H-NMR (400 MHz, C_6D_6): δ = 7.10 (s, 2H), 5.15 (m_c, 1H), 3.64 (m_c, 1H), 3.24-3.19 (m, 2H), 2.77-2.70 (m, 1H), 2.61 (m_c, 1H), 1.80 (m_c, 1H), 1.36-1.33 (m, 12H), 1.17 (d, J = 6.9 Hz, 6H), 1.14 (d, J = 6.9 Hz, 3H), 1.04 (d, J = 6.7 Hz, 3H), 1.01 (s, 9H), 0.92 (d, J = 6.9 Hz, 3H), 0.35 (m_c, 9H), 0.19 (s, 3H), 0.05 (s, 3H) ppm;

^{13}C-NMR (101 MHz, C_6D_6): δ = 171.2, 150.5, 146.2, 131.0, 121.4, 77.8, 75.3, 41.1, 34.8, 33.1, 31.9, 26.6, 24.9, 24.7, 24.1, 20.1, 18.9, 18.4, 14.5, −3.1, −3.5, −7.0 ppm;

HRMS (ESI): $C_{32}H_{60}O_3SiSnNa$ $[M+Na]^+$ calculated: 663.3231, found: 663.3233;

$\mathbf{R}_{\boldsymbol{f}}$ = 0.3 (PE:MTBE 98:2, uv, vanillin).

Stannane 19

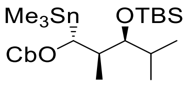

19
$C_{23}H_{51}NO_3SiSn$
536.46

According to GP8, carbamate **10** (200 mg, 0.54 mmol, 1.0 equiv) and (+)-sparteine (0.18 mL, 0.80 mmol, 1.5 equiv) gave stannane (+)-sp-**19** (125 mg, 0.23 mmol, 43%, dr ≥ 19:1) after purification by flash column chromatography (PE:MTBE 100:1) as a colorless oil.

^{1}H-NMR (400 MHz, C_6D_6): δ = 4.69 (m_c, 1H), 3.85-3.68 (m, 3H), 2.25 (m_c, 1H), 1.97 (m_c, 1H), 1.27 (d, J = 7.0 Hz, 3H), 1.10-1.06 (m, 12H), 1.03 (s, 9H), 0.99 (d, J = 7.0 Hz, 3H), 0.93 (d, J = 6.8 Hz, 3H), 0.34 (m_c, 9H), 0.18 (s, 3H), 0.14 (s, 3H) ppm;

^{13}C-NMR (101 MHz, C_6D_6): δ = 156.5, 79.9, 74.7, 46.1 (brs), 41.4, 31.5, 26.5, 21.5, 21.3 (brs), 20.7 (brs), 18.9, 16.3, 16.1, −3.1, −3.3, −7.3 ppm;

HRMS (ESI): $C_{23}H_{51}NO_3SiSnNa$ $[M+Na]^+$ calculated: 560.2558, found: 560.2560;

$\mathbf{R}_{\boldsymbol{f}}$ = 0.5 (PE:MTBE 95:5, vanillin);

$[\boldsymbol{\alpha}]_{\mathbf{D}}^{\mathbf{20}}$ = −40.4 (c 0.9, $CHCl_3$).

Stannane 23

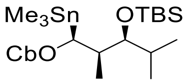

23
$C_{23}H_{51}NO_3SiSn$
536.46

According to GP8, carbamate **10** (175 mg, 0.47 mmol, 1.0 equiv) and (−)-sparteine (0.16 mL, 0.70 mmol, 1.5 equiv) gave stannane (−)-sp-**23** (154 mg, 0.29 mmol, 62%, dr ≥ 19:1) after purification by flash column chromatography (PE:MTBE 100:1) as a colorless oil.
According to GP8, carbamate **10** (200 mg, 0.54 mmol, 1.0 equiv) and TMEDA (0.12 mL, 0.80 mmol, 1.5 equiv) gave stannane TMEDA-**23** (174 mg, 0.32 mmol, 59%, dr 4.8:1) after purification by flash column chromatography (PE:MTBE 100:1) as a colorless oil.

Analytical data are given for stannane (−)-sp-**23** obtained by the reaction of carbamate **10** and (−)-sparteine (dr ≥ 19:1).

^{1}H-NMR (400 MHz, C_6D_6): δ = 4.32 (m_c, 1H), 3.91 (brs, 1H), 3.77 (dd, J = 6.8, 1.2 Hz, 1H), 3.61 (brs, 1H), 2.63 (m_c, 1H), 1.77 (m_c, 1H), 1.16 (d, J = 6.7 Hz, 6H), 1.08-1.03 (m, 15H), 1.01 (d, J = 6.9 Hz, 3H), 0.98 (d, J = 6.8 Hz, 3H), 0.90 (d, J = 6.8 Hz, 3H), 0.33 (m_c, 9H), 0.25 (s, 3H), 0.16 (s, 3H) ppm;

^{13}C-NMR (101 MHz, C_6D_6): δ = 155.8, 76.5, 75.0, 46.0, 45.9, 38.4, 33.8, 26.5, 21.3 (brs), 20.7 (brs), 20.2, 19.6, 18.9, 12.1, −3.4, −3.6, −7.3 ppm;

HRMS (ESI): $C_{23}H_{51}NO_3SiSnNa$ $[M+Na]^+$ calculated: 560.2558, found: 560.2552;

R*f* = 0.5 (PE:MTBE 95:5, vanillin);

$[\alpha]_D^{20}$ = −9.98 (*c* 1.4, $CHCl_3$).

Stannane 22

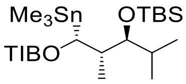

22
$C_{32}H_{60}O_3SiSn$
639.62

According to GP8, TIB ester **11** (175 mg, 0.37 mmol, 1.0 equiv) and (+)-sparteine (0.13 mL, 0.55 mmol, 1.5 equiv) gave stannane (+)-sp-**22** (166 mg, contaminated with 66% TIB ester, 0.09 mmol, 24%, dr 17:1) after purification by flash column chromatography (PE:MTBE 200:1 → 150:1) as a colorless oil.

^{1}H-NMR (400 MHz, C_6D_6): δ = 7.09 (s, 2H), 5.67 (m_c, 1H), 3.57 (dd, *J* = 7.8, 2.3 Hz, 1H), 3.19 (covered by TIB ester, sep, *J* = 6.8 Hz, 2H), 2.82-2.71 (covered by TIB ester, m, 1H), 2.52 (m_c, 1H), 1.85 (m_c, 1H), 1.33 (d, *J* = 6.9 Hz, 12H), 1.18 (partly covered by TIB ester, d, *J* = 6.8 Hz, 6H), 1.06-1.04 (m, 12H), 0.98 (d, *J* = 6.9 Hz, 3H), 0.94 (d, *J* = 6.8 Hz, 3H), 0.38 (m_c, 9H), 0.25 (s, 3H), 0.10 (s, 3H) ppm;

^{13}C-NMR (101 MHz, C_6D_6): δ = 171.0, 150.4, 145.9, 131.6, 121.3, 77.6, 77.1, 44.5, 34.8, 32.0, 30.6, 26.4, 24.7, 24.5, 24.2, 21.7, 18.7, 16.1, 15.7, −3.1, −3.2, −6.9 ppm;

HRMS (ESI): $C_{32}H_{60}O_3SiSnNa$ $[M+Na]^+$ calculated: 663.3231, found: 663.3231;

R*f* = 0.3 (PE:MTBE 98:2, uv, vanillin).

Stannane 18

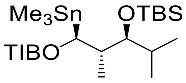

18
$C_{32}H_{60}O_3SiSn$
639.62

According to GP8, TIB ester **11** (175 mg, 0.37 mmol, 1.0 equiv) and (−)-sparteine (0.13 mL, 0.55 mmol, 1.5 equiv) gave stannane (−)-sp-**18** (201 mg, contaminated with 53% TIB ester, 0.15 mmol, 41%, dr 19:1) after purification by flash column chromatography (PE:MTBE 200:1 → 150:1) as a colorless oil.
According to GP8, TIB ester **11** (210 mg, 0.44 mmol, 1.0 equiv) and TMEDA (0.10 mL, 0.66 mmol, 1.5 equiv) gave stannane TMEDA-**18** (258 mg, 0.40 mmol, 91%, dr 3.1:1) after purification by flash column chromatography (PE:MTBE 200:1 → 150:1) as a colorless oil.

Analytical data are given for stannane (−)-sp-**18** obtained by the reaction of TIB ester **11** and (−)-sparteine (dr 19:1).

^{1}H-NMR (400 MHz, C_6D_6): δ = 7.11 (s, 2H), 5.21 (m_c, 1H), 3.68 (dd, J = 8.4, 1.8 Hz, 1H), 3.23-3.15 (covered by TIB ester, m, 2H), 2.82-2.69 (covered by TIB ester, m, 1H), 2.20-2.10 (covered by TIB ester, m, 1H), 1.78-1.70 (covered by TIB ester, m, 1H), 1.35-1.31 (partly covered by TIB ester, m, 12H), 1.18 (dd, J = 6.9, 1.0 Hz, 6H), 1.10 (d, J = 7.2 Hz, 3H), 1.03-1.01 (m, 12H), 0.93 (d, J = 7.0 Hz, 3H), 0.39 (m_c, 9H), 0.23 (s, 3H), 0.09 (s, 3H) ppm;

^{13}C-NMR (101 MHz, C_6D_6): δ = 171.9, 150.5, 146.1, 131.1, 121.4, 78.8, 75.3, 42.4, 34.8, 32.0, 31.0, 26.5, 25.0, 24.7, 24.1, 21.5, 18.9, 14.8, 14.4, −3.1, −3.4, −7.3 ppm;

HRMS (ESI): $C_{32}H_{60}O_3SiSnNa$ $[M+Na]^+$ calculated: 663.3231, found: 663.3231;

R$_f$ = 0.3 (PE:MTBE 98:2, uv, vanillin).

Stannane 24

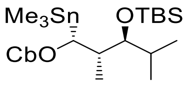

24
$C_{23}H_{51}NO_3SiSn$
536.46

According to GP8, carbamate **12** (175 mg, 0.47 mmol, 1.0 equiv) and (+)-sparteine (0.16 mL, 0.70 mmol, 1.5 equiv) gave stannane (+)-sp-**24** (72 mg, 0.13 mmol, 28%, dr ≥ 19:1) after purification by flash column chromatography (PE:MTBE 100:1) as a colorless oil.
According to GP8, carbamate **12** (175 mg, 0.47 mmol, 1.0 equiv) and TMEDA (0.11 mL, 0.70 mmol, 1.5 equiv) gave stannane TMEDA-**24** (101 mg, 0.19 mmol, 40%, dr 5:1) after purification by flash column chromatography (PE:MTBE 100:1) as a colorless oil.

Analytical data are given for stannane (+)-sp-**24** obtained by the reaction of carbamate **12** and (+)-sparteine (dr ≥ 19:1).

^{1}H-NMR (400 MHz, C_6D_6): δ = 4.22 (m_c, 1H), 3.86-3.60 (m, 3H), 2.77 (m_c, 1H), 1.99 (m_c, 1H), 1.17 (brs, 6H), 1.07 (d, J = 6.9 Hz, 3H), 1.05-1.01 (m, 18H), 0.99 (d, J = 7.2 Hz, 3H), 0.30 (m_c, 9H), 0.18 (s, 3H), 0.14 (s, 3H) ppm;

^{13}C-NMR (101 MHz, C_6D_6): δ = 155.9, 76.7, 75.1, 45.9, 42.8, 29.1, 26.3, 22.9, 21.3 (brs), 20.7 (brs), 18.8, 18.6, 12.6, −3.8, −4.4, −7.5 ppm;

HRMS (ESI): $C_{23}H_{51}NO_3SiSnNa$ $[M+Na]^+$ calculated: 560.2558, found: 560.2564;

R$_f$ = 0.5 (PE:MTBE 95:5, vanillin);

$[\alpha]_D^{20}$ = +4.83 (c 1.0, $CHCl_3$).

Stannane 20

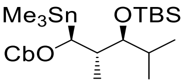

20
$C_{23}H_{51}NO_3SiSn$
536.46

According to GP8, carbamate **12** (175 mg, 0.47 mmol, 1.0 equiv) and (−)-sparteine (0.16 mL, 0.70 mmol, 1.5 equiv) gave stannane (−)-sp-**20** (170 mg, 0.32 mmol, 68%, dr ≥ 19:1) after purification by flash column chromatography (PE:MTBE 100:1) as a colorless oil.

^{1}H-NMR (400 MHz, C_6D_6): δ = 4.99 (m_c, 1H), 3.88-3.68 (m, 3H), 2.06 (m_c, 1H), 1.85 (m_c, 1H), 1.17-1.01 (m, 27H), 0.95 (d, J = 6.8 Hz, 3H), 0.32 (m_c, 9H), 0.19 (s, 3H), 0.14 (s, 3H) ppm;

^{13}C-NMR (101 MHz, C_6D_6): δ = 156.4, 78.5, 74.4, 45.9 (brs), 41.7, 30.8, 26.6, 22.0, 21.4 (brs), 20.7 (brs), 18.9, 15.4, 14.7, −2.8, −4.3, −7.9 ppm;

HRMS (ESI): $C_{23}H_{51}NO_3SiSnNa$ $[M+Na]^+$ calculated: 560.2558, found: 560.2562;

R*f* = 0.5 (PE:MTBE 95:5, vanillin);

$[\boldsymbol{\alpha}]_{\mathbf{D}}^{\mathbf{20}}$ = +50.2 (c 1.3, $CHCl_3$).

(*R*)-Allylic alcohol 13d

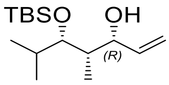

(*R*)-13d
$C_{15}H_{32}O_2Si$
272.50

According to GP9, stannane TMEDA-**17** (156 mg, 0.24 mmol, 1.5 equiv) and vinyl boronic ester **8** (25 mg, 0.16 mmol, 1.0 equiv) gave allylic alcohol **(*R*)-13d** (23 mg, 0.08 mmol, 50% o2s, dr 3.1:1) after purification by flash column chromatography (PE:MTBE 95:5) as a colorless oil.
Analytical data are in accordance with those given for allylic alcohol **(*R*)-13d** obtained by the reaction of TIB ester **9** and (+)-sparteine (dr ≥ 19:1).[4]

^{1}H-NMR (400 MHz, C_6D_6): δ = 5.74 (ddd, J = 17.4, 10.5, 5.7 Hz, 1H), 5.22 (dt, J = 17.2, 1.6 Hz, 1H), 5.03 (dt, J = 10.6, 1.6 Hz, 1H), 4.01 (brs, 1H), 3.59 (t, J = 4.1 Hz, 1H), 1.85-1.75 (m, 2H), 1.70-1.60 (m, 1H), 1.05 (d, J = 7.0 Hz, 3H), 1.01 (s, 9H), 0.91 (d, J = 6.9 Hz, 3H), 0.87 (d, J = 7.1 Hz, 3H), 0.10 (s, 3H), 0.07 (s, 3H) ppm;

^{13}C-NMR (101 MHz, C_6D_6): δ = 141.2, 114.6, 78.5, 75.0, 41.3, 33.3, 26.4, 19.4, 18.7, 18.2, 10.2, −3.4, −3.7 ppm;

HRMS (ESI): $C_{15}H_{32}O_2SiNa$ $[M+Na]^+$ calculated: 295.2069, found: 295.2077;

R*f* = 0.2 (PE:MTBE 9:1, vanillin);

$[\alpha]_D^{20}$ = +17.4 (c 0.8, $CHCl_3$).

(*S*)-Allylic alcohol 14d

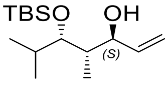

(*S*)-14d
$C_{15}H_{32}O_2Si$
272.50

According to GP10, stannane TMEDA-**23** (131 mg, 0.24 mmol, 1.5 equiv) and vinyl boronic ester **8** (25 mg, 0.16 mmol, 1.0 equiv) gave allylic alcohol **(*S*)-14d** (24 mg, 0.09 mmol, 56% o2s, dr 8:1) after purification by flash column chromatography (PE:MTBE 9:1) as a colorless oil.
Analytical data are in accordance with those given for allylic alcohol **(*S*)-14d** obtained by the reaction of carbamate **10** and (−)-sparteine (dr ≥ 19:1).

^{1}H-NMR (400 MHz, C_6D_6): δ = 5.71 (m_c, 1H), 5.15 (m_c, 1H), 4.99 (m_c, 1H), 3.91-3.85 (m, 2H), 1.78 (m_c, 1H), 1.69-1.61 (m, 2H), 1.02 (s, 9H), 0.99 (d, J = 6.7 Hz, 3H), 0.87-0.84 (m, 6H), 0.15 (s, 3H), 0.11 (s, 3H) ppm;

^{13}C-NMR (101 MHz, C_6D_6): δ = 141.4, 115.5, 77.2, 75.5, 42.1, 32.6, 26.5, 20.1, 19.9, 18.7, 11.6, −3.75, −3.79 ppm;

HRMS (ESI): $C_{15}H_{32}O_2SiNa$ $[M+Na]^+$ calculated: 295.2066, found: 295.2069;

R*f* = 0.2 (PE:MTBE 95:5, vanillin);

$[\alpha]_D^{20}$ = −10.2 (c 0.9, $CHCl_3$).

(*R*)-Allylic alcohol 13c

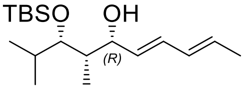

(*R*)-13c
$C_{18}H_{36}O_2Si$
312.57

According to GP9, stannane TMEDA-**17** (156 mg, 0.24 mmol, 1.5 equiv) and vinyl boronic ester **7** (32 mg, 0.16 mmol, 1.0 equiv) gave allylic alcohol **(*R*)-13c** (37 mg, 0.12 mmol, 75% o2s, dr 3:1) after purification by flash column chromatography (PE:MTBE 95:5) as a colorless oil.
Analytical data are in accordance with those given for allylic alcohol allylic alcohol **(*R*)-13c** obtained by the reaction of TIB ester **9** and TMEDA (dr 13:1).

^{1}H-NMR (400 MHz, C_6D_6): δ = 6.30-6.23 (m, 1H), 6.08-6.01 (m, 1H), 5.59-5.50 (m, 2H), 4.08 (brs, 1H), 3.63 (t, J = 4.1 Hz, 1H), 1.86-1.78 (m, 1H), 1.74-1.68 (m, 1H), 1.59 (dd, J = 6.6, 1.3 Hz, 3H), 1.22-1.20 (m, 1H), 1.10 (d, J = 6.9 Hz, 3H), 1.02 (s, 9H), 0.92 (d, J = 6.9 Hz, 3H), 0.88 (d, J = 6.8 Hz, 3H), 0.13 (s, 3H), −0.08 (s, 3H) ppm;

^{13}C-NMR (101 MHz, C_6D_6): δ = 133.7, 131.7, 131.3, 129.0, 78.4, 74.9, 41.8, 33.5, 26.4, 19.3, 18.7, 18.3, 18.2, 10.5, −3.4, −3.7 ppm;

HRMS (ESI): $C_{18}H_{36}O_2SiNa$ [M+Na]$^+$ calculated: 335.2382, found: 335.2375;

R$_f$ = 0.3 (PE:MTBE 95:5, uv, vanillin);

$[\alpha]_D^{20}$ = +21.8 (*c* 1.0, $CHCl_3$).

(*S*)-Allylic alcohol 14c

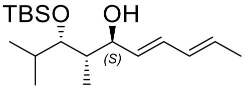

(*S*)-14c
$C_{18}H_{36}O_2Si$
312.57

According to GP10, stannane TMEDA-**23** (131 mg, 0.24 mmol, 1.5 equiv) and vinyl boronic ester **7** (32 mg, 0.16 mmol, 1.0 equiv) gave allylic alcohol **(*S*)-14c** (38 mg, 0.12 mmol, 75% o2s, dr 6:1) after purification by flash column chromatography (PE:MTBE 95:5) as a colorless oil.

^{1}H-NMR (400 MHz, C_6D_6): δ = 6.23-6.17 (m, 1H), 6.05-5.98 (m, 1H), 5.56-5.43 (m, 2H), 3.97-3.90 (m, 2H), 1.84-1.76 (m, 1H), 1.74-1.66 (m, 1H), 1.60-1.58 (m, 4H), 1.04 (s, 9H), 1.01 (d, *J* = 6.8 Hz, 3H), 0.90-0.87 (m, 6H), 0.19 (s, 3H), 0.13 (s, 3H) ppm;

^{13}C-NMR (101 MHz, C_6D_6): δ = 133.9, 132.2, 131.7, 129.3, 77.1, 74.8, 42.4, 32.8, 26.5, 20.04, 20.03, 18.8, 18.2, 11.6, −3.72, −3.73 ppm;

HRMS (ESI): $C_{18}H_{36}O_2SiNa$ [M+Na]$^+$ calculated: 335.2382, found: 335.2375;

R$_f$ = 0.3 (PE:MTBE 95:5, uv, vanillin);

$[\alpha]_D^{20}$ = −18.9 (*c* 0.9, $CHCl_3$).

(*S*)-Allylic alcohol 13b

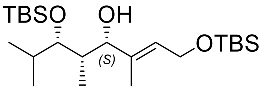

(*S*)-13b
$C_{23}H_{50}O_3Si_2$
430.82

According to GP9, stannane TMEDA-**17** (156 mg, 0.24 mmol, 1.5 equiv) and vinyl boronic ester **6** (50 mg, 0.16 mmol, 1.0 equiv) gave allylic alcohol **(*S*)-13b** (41 mg, 0.10 mmol, 63% o2s, dr ≥ 19:1) after purification by flash column chromatography (PE:MTBE 95:5) as a colorless oil.
Analytical data are in accordance with those given for allylic alcohol **(*S*)-13b** obtained by the reaction of TIB ester **9** and TMEDA (dr 10:1).

^{1}H-NMR (400 MHz, C_6D_6): δ = 5.80 (m_c, 1H), 4.26 (m_c, 2H), 3.87 (d, *J* = 4.9 Hz, 1H), 3.59 (t, *J* = 4.0 Hz, 1H), 1.85-1.77 (m, 2H), 1.46 (s, 3H), 1.18-1.17 (m, 1H), 1.08 (d, *J* = 6.9 Hz, 3H), 1.02 (s, 9H), 1.01 (s, 9H), 0.95 (d, *J* = 6.9 Hz, 3H), 0.89 (d, *J* = 6.9 Hz, 3H), 0.15 (s, 3H), 0.12 (s, 6H), 0.10 (s, 3H) ppm;

^{13}C-NMR (101 MHz, C_6D_6): δ = 138.0, 126.5, 78.6, 78.5, 60.1, 38.3, 33.4, 26.5, 26.2, 19.7, 18.8, 18.5, 18.1, 13.0, 10.1, −3.4, −3.5, −4.9, −5.0 ppm;

HRMS (ESI): $C_{23}H_{50}O_3Si_2Na$ $[M+Na]^+$ calculated: 453.3196, found: 453.3200;

R_f = 0.3 (PE:MTBE 9:1, vanillin);

$[\alpha]_D^{20}$ = +6.52 (*c* 1.0, $CHCl_3$).

(*R*)-Allylic alcohol 14b

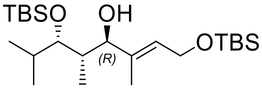

(*R*)-14b
$C_{23}H_{50}O_3Si_2$
430.82

According to GP10, stannane TMEDA-**23** (131 mg, 0.24 mmol, 1.5 equiv) and vinyl boronic ester **6** (50 mg, 0.16 mmol, 1.0 equiv) gave allylic alcohol **(*R*)-14b** (52 mg, 0.12 mmol, 75% o2s, dr 4:1) after purification by flash column chromatography (PE:MTBE 95:5) as a colorless oil.
Analytical data are in accordance with those given for allylic alcohol **(*R*)-14b** obtained by the reaction of carbamate **10** and TMEDA (dr 4.3:1).

^{1}H-NMR (400 MHz, C_6D_6): δ = 5.68 (m_c, 1H), 4.28-4.21 (m, 2H), 3.94-3.87 (m, 2H), 1.89-1.77 (m, 2H), 1.70 (brs, 1H), 1.57 (d, *J* = 1.2 Hz, 3H), 1.02-1.01 (m, 12H), 1.00 (s, 9H), 0.89 (d, *J* = 6.8 Hz, 3H), 0.79 (d, *J* = 7.1 Hz, 3H), 0.15 (s, 3H), 0.12-0.10 (m, 9H) ppm;

^{13}C-NMR (101 MHz, C_6D_6): δ = 138.0, 128.6, 79.8, 77.5, 60.1, 39.2, 32.6, 26.4, 26.2, 20.3, 20.2, 18.7, 18.5, 11.7, 10.8, −3.7, −3.8, −4.941, −4.945 ppm;

HRMS (ESI): $C_{23}H_{50}O_3Si_2Na$ $[M+Na]^+$ calculated: 453.3196, found: 453.3199;

$\mathbf{R}_f$ = 0.3 (PE:MTBE 9:1, vanillin);

$[\alpha]_D^{20}$ = −8.63 (*c* 1.0, $CHCl_3$).

(*S*)-Allylic alcohol 13a

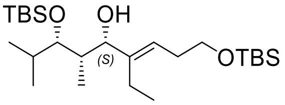

(*S*)-13a
$C_{25}H_{54}O_3Si_2$
458.87

According to GP9, stannane TMEDA-**17** (156 mg, 0.24 mmol, 1.5 equiv) and vinyl boronic ester **5** (55 mg, 0.16 mmol, 1.0 equiv) gave allylic alcohol **(*S*)-13a** (53 mg, 0.12 mmol, 75% o2s, dr ≥ 19:1) after purification by flash column chromatography (PE:MTBE 95:5) as a colorless oil.
Analytical data are in accordance with those given for allylic alcohol **(*S*)-13a** obtained by the reaction of TIB ester **9** and TMEDA (dr 25:1).

^{1}H-NMR (400 MHz, C_6D_6): δ = 5.58 (m_c, 1H), 4.09 (brs, 1H), 3.68-3.62 (m, 3H), 2.40-2.30 (m, 2H), 2.15-2.02 (m, 1H), 1.94-1.81 (m, 3H), 1.26 (d, *J* = 3.2 Hz, 1H), 1.07 (d, *J* = 7.0 Hz, 3H), 1.03 (s, 9H), 1.00-0.96 (m, 15H), 0.93 (d, *J* = 6.8 Hz, 3H), 0.16 (s, 3H), 0.10 (s, 3H), 0.09 (s, 6H) ppm;

^{13}C-NMR (101 MHz, C_6D_6): δ = 144.5, 121.7, 79.6, 76.9, 63.5, 38.8, 33.1, 31.8, 26.5, 26.2, 21.8, 19.9, 18.8, 18.6, 17.8, 14.4, 9.8, −3.3, −3.6, −5.097, −5.105 ppm;

HRMS (ESI): $C_{25}H_{54}O_3Si_2Na$ $[M+Na]^+$ calculated: 481.3509, found: 481.3514;

$\mathbf{R}_f$ = 0.3 (PE:MTBE 95:5, vanillin);

$[\alpha]_D^{20}$ = +10.3 (*c* 0.9, $CHCl_3$).

(*R*)-Allylic alcohol 14a

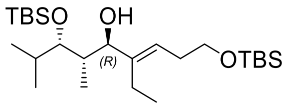

(*R*)-14a
$C_{25}H_{54}O_3Si_2$
458.87

According to GP10, stannane TMEDA-**23** (131 mg, 0.24 mmol, 1.5 equiv) and vinyl boronic ester **5** (55 mg, 0.16 mmol, 1.0 equiv) gave allylic alcohol **(*R*)-14a** (51 mg, contaminated with 5% ketone, 0.11 mmol, 69% o2s, dr 4:1) after purification by flash column chromatography (PE:MTBE 95:5) as a colorless oil.
Analytical data are in accordance with those given for allylic alcohol **(*R*)-14a** obtained by the reaction of carbamate **10** and TMEDA (dr 4.2:1).

^{1}H-NMR (400 MHz, C_6D_6): δ = 5.45 (m_c, 1H), 3.99-3.95 (m, 2H), 3.59 (t, *J* = 6.7 Hz, 2H), 2.31 (q, *J* = 6.8 Hz, 2H), 2.16-2.05 (m, 2H), 1.94-1.82 (m, 2H), 1.66 (brs, 1H), 1.10 (t, *J* = 7.6 Hz, 3H), 1.05-1.03 (m, 12H), 1.00 (s, 9H), 0.92 (d, *J* = 6.9 Hz, 3H), 0.85 (d, *J* = 7.0 Hz, 3H), 0.21 (s, 3H), 0.14 (s, 3H), 0.08 (s, 6H) ppm;

^{13}C-NMR (101 MHz, C_6D_6): δ = 144.8, 125.3, 80.1, 77.5, 63.2, 39.9, 32.7, 31.7, 26.5, 26.2, 20.34, 20.32, 20.29, 18.8, 18.5, 15.3, 12.0, −3.71, −3.74, −5.13, −5.14 ppm;

HRMS (ESI): $C_{25}H_{54}O_3Si_2Na$ $[M+Na]^+$ calculated: 481.3509, found: 481.3506;

R_f = 0.3 (PE:MTBE 95:5, vanillin);

$[\alpha]_D^{20}$ = −3.80 (*c* 1.0, $CHCl_3$).

(*S*)-Allylic alcohol 15d

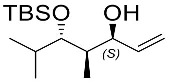

(*S*)-15d
$C_{15}H_{32}O_2Si$
272.50

According to GP9, stannane TMEDA-**18** (156 mg, 0.24 mmol, 1.5 equiv) and vinyl boronic ester **8** (25 mg, 0.16 mmol, 1.0 equiv) gave allylic alcohol **(*S*)-15d** (29 mg, 0.11 mmol, 69% o2s, dr 3:1) after purification by flash column chromatography (PE:MTBE 95:5) as a colorless oil.
Analytical data are in accordance with those given for allylic alcohol **(*S*)-15d** obtained by the reaction of carbamate **12** and (−)-sparteine (dr 19:1).

^{1}H-NMR (400 MHz, C_6D_6): δ = 5.77 (m_c, 1H), 5.44 (dt, *J* = 17.3, 1.9 Hz, 1H), 5.12 (dt, *J* = 10.6, 1.9 Hz, 1H), 4.68-4.64 (m, 1H), 3.40 (t, *J* = 4.9 Hz, 1H), 1.84-1.73 (m, 1H), 1.65-1.57 (m, 1H), 0.97 (s, 9H), 0.94 (d, *J* = 7.1 Hz, 3H), 0.87 (d, *J* = 7.1 Hz, 3H), 0.82 (d, *J* = 6.9 Hz, 3H), 0.09 (s, 3H), 0.04 (s, 3H) ppm;

^{13}C-NMR (101 MHz, C_6D_6): δ = 141.2, 113.7, 81.9, 71.4, 40.4, 32.1, 26.4, 20.2, 18.7, 18.5, 11.5, −3.56, −3.64 ppm;

HRMS (ESI): $C_{15}H_{32}O_2SiNa$ $[M+Na]^+$ calculated: 295.2069, found: 295.2077;

R$_f$ = 0.2 (PE:MTBE 95:5, vanillin);

$[\alpha]_D^{20}$ = −3.0 (*c* 0.8, $CHCl_3$).

(*R*)-Allylic alcohol 16d

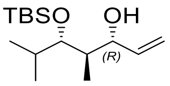

(*R*)-16d
$C_{15}H_{32}O_2Si$
272.50

According to GP10, stannane TMEDA-**24** (131 mg, 0.24 mmol, 1.5 equiv) and vinyl boronic ester **8** (25 mg, 0.16 mmol, 1.0 equiv) gave allylic alcohol **(*R*)-16d** (24 mg, 0.09 mmol, 56% o2s, dr 6:1) after purification by flash column chromatography (PE:MTBE 9:1) as a colorless oil.
Analytical data are in accordance with those given for the pure allylic alcohol **(*R*)-16d** obtained by the reaction of carbamate **12** and TMEDA.

^{1}H-NMR (400 MHz, C_6D_6): δ = 5.84 (m_c, 1H), 5.27 (ddd, *J* = 17.1, 10.5, 4.1 Hz, 1H), 5.06 (m_c, 1H), 4.04 (t, *J* = 7.2 Hz, 1H), 3.57 (dd, *J* = 5.3, 4.2 Hz, 1H), 2.22 (brs, 1H), 1.88-1.78 (m, 2H), 0.98 (s, 9H), 0.95 (d, *J* = 6.7 Hz, 3H), 0.93 (d, *J* = 6.5 Hz, 3H), 0.83 (d, *J* = 5.9 Hz, 3H), 0.09 (s, 3H), 0.05 (s, 3H) ppm;

^{13}C-NMR (101 MHz, C_6D_6): δ = 140.5, 115.1, 80.4, 75.3, 43.5, 32.4, 26.3, 20.5, 18.5, 18.2, 14.4, −3.8, −4.1 ppm;

HRMS (ESI): $C_{15}H_{32}O_2SiNa$ $[M+Na]^+$ calculated: 295.2069, found: 295.2066;

R$_f$ = 0.2 (PE:MTBE 95:5, vanillin);

$[\alpha]_D^{20}$ = +8.3 (*c* 0.8, $CHCl_3$).

(*S*)-Allylic alcohol 15c

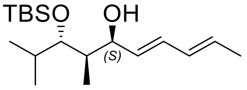

(*S*)-15c
$C_{18}H_{36}O_2Si$
312.57

According to GP9, stannane TMEDA-**18** (156 mg, 0.24 mmol, 1.5 equiv) and vinyl boronic ester **7** (32 mg, 0.16 mmol, 1.0 equiv) gave allylic alcohol **(*S*)-15c** (38 mg, 0.12 mmol, 75% o2s, dr 4.4:1) after purification by flash column chromatography (PE:MTBE 98:2 → 10:1) as a colorless oil.

Analytical data are in accordance with those given for allylic alcohol ***(S)*-15c** obtained by the reaction of TIB ester **11** and TMEDA (dr 4.8:1).

^{1}H-NMR (400 MHz, C_6D_6): δ = 6.48 (m_c, 1H), 6.14-6.07 (m, 1H), 5.59-5.54 (m, 2H), 4.73 (brs, 1H), 3.43 (t, J = 4.9 Hz, 1H), 2.56 (d, J = 2.3 Hz, 1H), 1.86-1.77 (m, 1H), 1.69-1.58 (m, 4H), 0.99-0.97 (m, 12H), 0.89 (d, J = 6.8 Hz, 3H), 0.85 (d, J = 6.9 Hz, 3H), 0.11 (s, 3H), 0.05 (s, 3H) ppm;

^{13}C-NMR (101 MHz, C_6D_6): δ = 134.0, 132.0, 130.1, 128.3, 81.9, 71.1, 41.1, 32.1, 26.4, 20.2, 18.7, 18.5, 18.2, 11.8, −3.55, −3.62 ppm;

HRMS (ESI): $C_{18}H_{36}O_2SiNa$ $[M+Na]^+$ calculated: 335.2382, found: 335.2387;

R*f* = 0.3 (PE:MTBE 95:5, uv, vanillin);

$[\alpha]_D^{20}$ = −0.92 (*c* 0.8, $CHCl_3$).

(*R*)-Allylic alcohol 16c

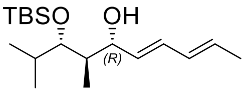

(*R*)-16c
$C_{18}H_{36}O_2Si$
312.57

According to GP10, stannane TMEDA-**24** (131 mg, 0.24 mmol, 1.5 equiv) and vinyl boronic ester **7** (32 mg, 0.16 mmol, 1.0 equiv) gave allylic alcohol ***(R)*-16c** (29 mg, 0.09 mmol, 56% o2s, dr 4.2:1) after purification by flash column chromatography (PE:MTBE 95:5) as a colorless oil.
Analytical data are in accordance with those given for allylic alcohol ***(R)*-16c** obtained by the reaction of carbamate **12** and TMEDA (dr 2:1).

^{1}H-NMR (400 MHz, C_6D_6): δ = 6.30 (dd, J = 15.1, 10.4 Hz, 1H), 6.10-6.03 (m, 1H), 5.63-5.52 (m, 2H), 4.09 (t, J = 7.6 Hz, 1H), 3.64 (dd, J = 5.0, 4.3 Hz, 1H), 2.22 (d, J = 2.4 Hz, 1H), 1.92-1.82 (m, 2H) 1.59 (dd, J = 6.6, 1.4 Hz, 3H), 1.00-0.98 (m, 12H), 0.95 (d, J = 6.8 Hz, 3H), 0.86 (d, J = 7.1 Hz, 3H), 0.13 (s, 3H), 0.07 (s, 3H) ppm;

^{13}C-NMR (101 MHz, C_6D_6): δ = 133.1, 131.9, 131.6, 129.0, 80.2, 75.0, 44.1, 32.3, 26.3, 20.6, 18.6, 18.3, 18.2, 14.4, −3.8, −4.1 ppm;

HRMS (ESI): $C_{18}H_{36}O_2SiNa$ $[M+Na]^+$ calculated: 335.2382, found: 335.2387;

R*f* = 0.3 (PE:MTBE 95:5, uv, vanillin);

$[\alpha]_D^{20}$ = +13.7 (*c* 0.9, $CHCl_3$).

(*R*)-Allylic alcohol 15b

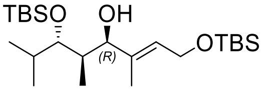

(*R*)-15b
$C_{23}H_{50}O_3Si_2$
430.82

According to GP9, stannane TMEDA-**18** (156 mg, 0.24 mmol, 1.5 equiv) and vinyl boronic ester **6** (50 mg, 0.16 mmol, 1.0 equiv) gave allylic alcohol **(*R*)-15b** (54 mg, 0.13 mmol, 81% o2s, dr ≥ 19:1) after purification by flash column chromatography (PE:MTBE 95:5) as a colorless oil.
Analytical data are in accordance with those given for allylic alcohol **(*R*)-15b** obtained by the reaction of TIB ester **11** and TMEDA (dr ≥ 19:1).

^{1}H-NMR (400 MHz, C_6D_6): δ = 6.08 (m_c, 1H), 4.43 (brs, 1H), 4.34 (m_c, 2H), 3.43 (m_c, 1H), 2.75 (d, *J* = 2.0 Hz, 1H), 1.87-1.75 (m, 2H), 1.51 (s, 3H), 1.01 (s, 9H), 0.98 (s, 9H), 0.94 (d, *J* = 7.1 Hz, 3H), 0.91 (d, *J* = 6.8 Hz, 3H), 0.86 (d, *J* = 6.9 Hz, 3H), 0.13 (s, 6H), 0.07 (s, 3H), 0.04 (s, 3H) ppm;

^{13}C-NMR (101 MHz, C_6D_6): δ = 137.3, 124.8, 82.1, 74.1, 60.4, 37.9, 32.1, 26.4, 26.2, 20.3, 18.69, 18.65, 18.6, 14.1, 11.4, −3.5, −3.6, −4.85, −4.88 ppm;

HRMS (ESI): $C_{23}H_{50}O_3Si_2Na$ $[M+Na]^+$ calculated: 453.3196, found: 453.3198;

R*f* = 0.3 (PE:MTBE 9:1, vanillin);

$[\alpha]_D^{20}$ = +10.1 (*c* 1.3, $CHCl_3$).

(*S*)-Allylic alcohol 16b

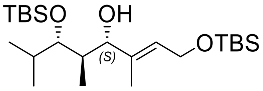

(*S*)-16b
$C_{23}H_{50}O_3Si_2$
430.82

According to GP10, stannane TMEDA-**24** (131 mg, 0.24 mmol, 1.5 equiv) and vinyl boronic ester **6** (50 mg, 0.16 mmol, 1.0 equiv) gave allylic alcohol **(*S*)-16b** (57 mg, 0.13 mmol, 81% o2s, dr 4.6:1) after purification by flash column chromatography (PE:MTBE 95:5) as a colorless oil.
Analytical data are in accordance with those given for allylic alcohol **(*S*)-16b** obtained by the reaction of carbamate **12** and TMEDA (dr 2.3:1).

^{1}H-NMR (400 MHz, C_6D_6): δ = 5.67 (m_c, 1H), 4.25 (m_c, 2H), 3.90 (dd, *J* = 9.8, 1.4 Hz, 1H), 3.69 (t, *J* = 4.4 Hz, 1H), 2.37 (d, *J* = 1.7 Hz, 1H), 2.01-1.92 (m, 1H), 1.90-1.83 (m 1H), 1.65 (m_c, 3H), 1.00 (s, 9H), 0.99-0.96 (m, 15H), 0.80 (d, *J* = 7.2 Hz, 3H), 0.13 (s, 3H), 0.105 (s, 3H), 0.103 (s, 3H), 0.06 (s, 3H) ppm;

^{13}C-NMR (101 MHz, C_6D_6): δ = 137.6, 128.5, 81.0, 80.3, 60.1, 40.7, 32.4, 26.24, 26.18, 20.7, 18.6, 18.53, 18.46, 15.0, 11.0, −3.9, −4.2, −4.9, −5.0 ppm;

HRMS (ESI): $C_{23}H_{50}O_3Si_2Na$ [M+Na]$^+$ calculated: 453.3196, found: 453.3198;

R$_f$ = 0.3 (PE:MTBE 9:1, vanillin);

$[\alpha]_D^{20}$ = +2.30 (c 1.0, $CHCl_3$).

(*R*)-Allylic alcohol 15a

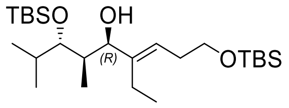

(*R*)-15a
$C_{25}H_{54}O_3Si_2$
458.87

According to GP9, stannane TMEDA-**18** (156 mg, 0.24 mmol, 1.5 equiv) and vinyl boronic ester **5** (55 mg, 0.16 mmol, 1.0 equiv) gave allylic alcohol **(*R*)-15a** (55 mg, 012 mmol, 75% o2s, dr ≥ 19:1) after purification by flash column chromatography (PE:MTBE 98:2 → 9:1) as a colorless oil.
Analytical data are in accordance with those given for allylic alcohol **(*R*)-15a** obtained by the reaction of TIB ester **11** and TMEDA (dr ≥ 19:1).

^{1}H-NMR (400 MHz, C_6D_6): δ = 5.76 (m_c, 1H), 4.62 (brs, 1H), 3.66 (t, J = 7.0 Hz, 2H), 3.51 (t, J = 5.1 Hz, 1H), 2.54 (d, J = 2.0 Hz, 1H), 2.48-2.35 (m, 2H), 2.16 (m_c, 1H), 1.91-1.77 (m, 3H), 1.06-0.99 (m, 21H), 0.96 (d, J = 7.1 Hz, 3H), 0.93 (d, J = 6.8 Hz, 3H), 0.90 (d, J = 7.0 Hz, 3H), 0.12 (s, 3H), 0.09 (s, 6H), 0.07 (s, 3H) ppm;

^{13}C-NMR (101 MHz, C_6D_6): δ = 143.8, 120.6, 81.9, 72.2, 63.6, 38.1, 32.1, 31.8, 26.5, 26.2, 22.1, 20.4, 18.7, 18.6, 18.3, 14.2, 11.3, −3.46, −3.55, −5.1 ppm;

HRMS (ESI): $C_{25}H_{54}O_3Si_2Na$ [M+Na]$^+$ calculated: 481.3509, found: 481.3511;

R$_f$ = 0.3 (PE:MTBE 95:5, vanillin);

$[\alpha]_D^{20}$ = +3.19 (c 1.2, $CHCl_3$).

(*S*)-Allylic alcohol 16a

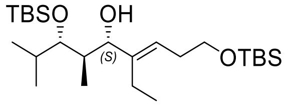

(*S*)-16a
$C_{25}H_{54}O_3Si_2$
458.87

According to GP10, stannane TMEDA-**24** (131 mg, 0.24 mmol, 1.5 equiv) and vinyl boronic ester **5** (50 mg, 0.16 mmol, 1.0 equiv) gave allylic alcohol **(*S*)-16a** (63 mg, 0.14 mmol,

88% o2s, dr 4.3:1) after purification by flash column chromatography (PE:MTBE 95:5) as a colorless oil.
Analytical data are in accordance with those given for allylic alcohol **(*S*)-16a** obtained by the reaction of carbamate **12** and TMEDA (dr 2.4:1).

^{1}H-NMR (400 MHz, C_6D_6): δ = 5.43 (t, J = 7.2 Hz, 1H), 3.98 (dd, J = 9.8, 1.5 Hz, 1H), 3.71 (t, J = 4.4 Hz, 1H), 3.61 (t, J = 6.7 Hz, 2H), 2.49 (d, J = 1.9 Hz, 1H), 2.34 (q, J = 6.7 Hz, 2H), 2.26-2.13 (m, 2H), 2.07-1.99 (m, 1H), 1.94-1.86 (m, 1H), 1.19 (t, J = 7.6 Hz, 3H), 1.01-0.98 (m, 24H), 0.85 (d, J = 7.1 Hz, 3H), 0.16 (s, 3H), 0.09 (s, 6H), 0.07 (s, 3H) ppm;

^{13}C-NMR (101 MHz, C_6D_6): δ = 144.3, 125.1, 81.7, 80.8, 63.3, 41.3, 32.8, 31.7, 26.3, 26.2, 20.6, 20.4, 18.7, 18.6, 18.5, 15.8, 15.3, −3.9, −4.2, −5.1 ppm;

HRMS (ESI): $C_{25}H_{54}O_3Si_2Na$ $[M+Na]^+$ calculated: 481.3509, found: 481.3511;

R*f* = 0.3 (PE:MTBE 95:5, vanillin);

$[\boldsymbol{\alpha}]_{\mathbf{D}}^{\mathbf{20}}$ = +0.81 (*c* 1.0, $CHCl_3$).

3. Conformational analysis

3-1. Conformational search with CREST

Calculations were performed on xtb-6.4.1 and crest 2.11.2.[9, 10] For the conformational searches the options –alpb ether and –nocross were used to simulate the solvent diethyl ether and to skip the structural crossing near the end of the process. Due to the size of the investigated systems (diketides **9**, **10**, **11** and **12**) this would otherwise crash the program. The starting structures for the conformational searches are given below. The calculated structure of *syn*-TIB ester **9** is the enantiomer of the empirically used one. This has however no influence on the relative energies of the observed conformers. Mirrored pictures of these structures are shown in the main part of this work. For *syn*-carbamate **10**, 4817 geometries were found, for *anti*-carbamate **12** 3175, for *syn*-TIB ester **9** 5717 and for *anti*-TIB ester **11** 5359 geometries.

Coordinates of starting structure *syn*-carbamate **10**.

	Coordinates		
Atom	x / A	y / A	z / A
H	6.4415	0.3723	0.0054
H	5.4713	0.1122	1.4585
H	5.3953	-2.4243	0.9416
H	6.3904	-2.1168	-0.484
H	-0.8926	1.4901	2.3526
H	-0.9575	2.9991	1.4317
H	4.8624	-2.9862	-0.6512
H	-2.1174	-1.9696	0.2719
H	-1.0735	0.8002	-2.6067
H	3.2236	-2.168	2.4071
H	5.6801	-0.4395	-2.247
C	-2.7466	0.1944	-0.8398
O	-1.4824	-0.3261	-0.9164
C	-0.5488	0.4203	-1.728
C	0.1171	1.5777	-0.9711
C	0.8767	1.0636	0.2783
C	1.0265	2.0595	1.4575
C	1.8182	3.3343	1.1367
C	-0.3302	2.3953	2.0961
O	-3.0622	1.2353	-1.4021
H	-5.0056	-2.9971	0.2407
H	0.812	-2.2393	-0.3117
H	4.1618	0.4584	-2.0849
H	4.1368	-1.2731	-2.4549
H	2.2358	-3.2817	-0.2356
H	1.9632	-2.2548	-1.65
H	-3.8423	-3.0971	-1.0944

Coordinates of starting structure *anti*-carbamate **12**.

	Coordinates		
Atom	x / A	y / A	z / A
H	2.6416	-1.8195	-1.8586
H	3.4275	-3.0594	-0.868
H	5.4287	0.7419	-1.6138
H	3.8347	0.4189	-2.316
H	2.9902	3.5495	0.4323
H	2.6506	3.9595	-1.2528
H	5.2545	-0.4755	-2.8813
H	-5.5103	-1.7984	0.0267
H	-0.7021	0.7597	-1.2495
H	4.1952	1.4128	2.2544
H	6.3602	-2.2118	-1.4244
C	-2.9916	0.3712	-0.2883
O	-1.9666	-0.5243	-0.2333
C	-0.6423	0.0176	-0.4527
C	-0.0383	0.6156	0.8237
C	1.346	1.2525	0.5429
C	1.3104	2.456	-0.4348
C	0.2395	3.4936	-0.0556
C	2.6847	3.1328	-0.5356
O	-2.8158	1.5732	-0.4522
H	-2.7545	-2.2884	1.2367
H	2.869	-1.3501	2.7891
H	5.7459	-2.5917	0.1863
H	6.5591	-1.0472	-0.1123
H	4.2931	-2.212	2.2053
H	2.7055	-2.5539	1.5063
H	-4.1901	-1.7476	2.1283

C	0.9845	2.3571	-1.9732	C	0.0152	-0.4182	1.9552
O	2.1704	0.5834	-0.1151	O	2.2356	0.2547	0.022
Si	2.8434	-0.9042	0.2801	Si	3.6658	-0.401	0.6089
C	1.8776	-2.2982	-0.5593	C	3.3472	-1.7555	1.8917
C	2.7864	-1.1968	2.1494	C	4.7419	0.9095	1.4491
C	4.6437	-0.8122	-0.3612	C	4.5178	-1.146	-0.9382
C	4.6487	-0.4985	-1.8726	C	5.8681	-1.7818	-0.5415
C	5.4114	0.3025	0.381	C	3.6115	-2.2304	-1.5605
C	5.3543	-2.1622	-0.122	C	4.77	-0.0481	-1.9925
H	1.7542	-1.2007	2.5171	H	5.0948	1.6806	0.7593
H	3.3322	-0.4271	2.7037	H	5.6199	0.4379	1.9047
H	4.9389	1.2795	0.2384	H	4.0835	-2.6547	-2.4575
H	0.1948	-0.3184	-2.0373	H	-0.0508	-0.8343	-0.7929
H	-3.5449	-3.9711	0.4189	H	-4.1775	-3.3387	1.3488
H	0.2826	0.2276	0.677	H	1.7273	1.6248	1.5084
H	1.6063	1.5029	2.2063	H	1.0685	2.055	-1.4281
H	1.2402	4.0255	0.5142	H	0.3299	4.3743	-0.7
H	2.7518	3.1002	0.6183	H	0.3716	3.836	0.979
H	2.0686	3.8662	2.0612	H	-0.7771	3.1083	-0.1634
H	-0.1868	2.9688	3.0179	H	3.4572	2.4369	-0.8669
N	-3.5867	-0.5708	-0.0813	N	-4.2137	-0.2282	-0.1402
C	-3.1618	-1.8404	0.5495	C	-4.4236	-1.6924	-0.0378
C	-3.9398	-3.0426	-0.0064	C	-3.8453	-2.2998	1.2489
H	-5.365	0.6533	-1.8989	H	-5.5506	0.7879	2.0124
H	-5.7343	-1.0734	-1.7125	H	-7.0038	1.3113	1.1443
H	-6.8243	0.1422	-1.024	H	-6.6501	-0.4096	1.3014
C	-5.7737	-0.0968	-1.2204	C	-6.1933	0.5753	1.1533
C	-3.2307	-1.765	2.0821	C	-3.9798	-2.454	-1.2961
H	-2.6363	-0.9261	2.4559	H	-4.2885	-3.5023	-1.2221
H	-4.2568	-1.6446	2.4451	H	-4.4405	-2.0271	-2.1918
H	-2.8347	-2.687	2.5203	H	-2.8956	-2.4231	-1.4156
C	-4.989	-0.1171	0.0995	C	-5.3978	0.6607	-0.1564
H	-5.444	-0.8805	0.7372	H	-4.9804	1.6652	-0.2337
C	-5.0785	1.218	0.8526	C	-6.2798	0.4204	-1.3896
H	-4.6415	2.0237	0.261	H	-7.0956	1.1503	-1.4131
H	-4.5528	1.1608	1.8107	H	-6.7342	-0.5767	-1.3882
H	-6.1272	1.4603	1.0545	H	-5.6978	0.5318	-2.3089
H	-0.6957	2.2284	-0.6302	H	-0.6978	1.4272	1.1455
H	1.7724	1.7205	-2.3842	H	0.4616	0.0134	2.8582
H	1.467	3.2212	-1.5159	H	0.6044	-1.294	1.6714
H	0.3665	2.7198	-2.8014	H	-0.9904	-0.7598	2.2136

Coordinates of starting structure *syn*-TIB ester **9**.

	Coordinates		
Atom	x / A	y / A	z / A
C	1.0768	0.4717	2.1661
H	0.6959	0.7366	3.1546
H	1.6528	-0.4518	2.2274
C	1.9269	1.615	1.6083
H	1.2754	2.4949	1.5787
C	3.0814	1.8878	2.5851
H	3.63	2.794	2.3152
H	2.7055	2.0252	3.6044
H	3.7905	1.0552	2.5885
C	2.4072	1.3428	0.161
H	1.5139	1.1481	-0.4514
C	3.1604	2.5394	-0.4737
H	4.0993	2.6564	0.083
C	3.5213	2.2641	-1.9402
H	4.1364	1.3691	-2.0435
H	2.6189	2.1323	-2.5499
H	4.0823	3.1056	-2.3602
C	2.3671	3.8536	-0.3763
H	1.3724	3.75	-0.8279
H	2.233	4.1951	0.6534
H	2.8879	4.6494	-0.9186
O	3.2613	0.1932	0.1522
Si	3.1067	-1.3066	-0.594
C	2.3431	-1.1478	-2.3173
H	1.3617	-0.6632	-2.2675
H	2.9632	-0.5728	-3.0101
H	2.1866	-2.1416	-2.7514
C	1.9971	-2.4725	0.3994
H	1.9242	-3.4452	-0.1002
H	2.3757	-2.6506	1.4113
H	0.9837	-2.0681	0.4837
C	4.8971	-1.9902	-0.6586
C	4.9017	-3.3848	-1.322
H	4.5308	-3.3521	-2.3529
H	4.2909	-4.1073	-0.7692
H	5.9239	-3.7857	-1.3582
C	5.8074	-1.0451	-1.47
H	5.4655	-0.933	-2.5054
H	5.853	-0.0489	-1.0193
H	6.832	-1.4401	-1.5081
C	5.4614	-2.11	0.7737
H	6.4928	-2.4881	0.7482
H	5.4753	-1.1403	1.2815

Coordinates of starting structure *anti*-TIB ester **11**.

	Coordinates		
Atom	x / A	y / A	z / A
C	-2.5835	-0.1523	0.2505
C	-2.8749	-0.5774	-1.0606
C	-4.2143	-0.5957	-1.4672
C	-5.2552	-0.2155	-0.6196
C	-4.9314	0.1909	0.6797
C	-3.6131	0.2311	1.1398
C	-1.7893	-1.0296	-2.0367
H	-4.456	-0.9205	-2.4758
C	-6.6997	-0.2487	-1.1018
H	-5.7296	0.487	1.3531
C	-3.318	0.6604	2.5771
C	-1.1639	-0.1339	0.7415
O	-0.4058	0.765	0.0713
C	0.9826	0.8325	0.4808
C	1.7205	1.7887	-0.4517
C	3.2381	1.7674	-0.1265
C	4.0674	2.7348	-1.0071
C	5.5282	2.8109	-0.5437
C	3.9922	2.391	-2.5022
O	-0.7283	-0.8196	1.6464
C	-7.3366	1.1535	-1.1014
C	-7.552	-1.2443	-0.2927
C	-4.1398	1.8811	3.0264
C	-3.5072	-0.5251	3.5444
C	-1.5791	0.0109	-3.1528
C	-2.0755	-2.4253	-2.6214
C	1.1135	3.1983	-0.3711
H	1.5967	1.401	-1.4702
O	3.7298	0.4323	-0.2755
Si	4.3908	-0.6461	0.8359
C	3.4568	-0.6045	2.4815
C	6.1987	-0.2374	1.2138
C	4.2511	-2.3518	-0.0246
C	4.7517	-3.4598	0.9278
C	2.7832	-2.6403	-0.405
C	5.1074	-2.3639	-1.3095
H	-0.8464	-1.1065	-1.4877
H	-6.682	-0.6011	-2.1415
H	-2.2642	0.9505	2.634
H	1.0212	1.1729	1.5223
H	1.4018	-0.1705	0.436
H	3.358	2.0951	0.9203
H	3.6334	3.7316	-0.8589

H	4.8773	-2.8038	1.3888
O	-0.0499	0.1466	1.3085
C	-1.2074	0.8186	1.5269
O	-1.3253	1.6948	2.3597
C	-4.4278	-0.4579	-1.0378
C	-4.02	-1.2475	0.0436
C	-2.9672	-0.8711	0.881
C	-2.3087	0.3489	0.6155
C	-2.6979	1.1723	-0.4575
C	-3.7556	0.7434	-1.2679
H	-4.5381	-2.1808	0.2387
H	-4.0671	1.3619	-2.1047
C	-2.5988	-1.7105	2.1038
H	-1.5358	-1.544	2.3079
C	-2.0198	2.5165	-0.7152
H	-1.0351	2.4926	-0.234
C	-5.5746	-0.889	-1.9432
H	-5.6968	-0.1034	-2.7004
C	-5.2563	-2.1998	-2.6867
H	-4.3308	-2.1131	-3.2641
H	-5.136	-3.0345	-1.9876
H	-6.0665	-2.4589	-3.3769
C	-6.9044	-0.9976	-1.174
H	-6.8593	-1.7805	-0.4092
H	-7.1504	-0.0564	-0.673
H	-7.7252	-1.2467	-1.8553
C	-3.3896	-1.2285	3.339
H	-3.2135	-0.1666	3.532
H	-4.4655	-1.371	3.1886
H	-3.0946	-1.7927	4.2308
C	-2.7744	-3.2233	1.8972
H	-3.8285	-3.5072	1.8088
H	-2.2532	-3.5703	0.9996
H	-2.3679	-3.7659	2.7567
C	-2.817	3.6584	-0.0532
H	-2.9093	3.4897	1.0225
H	-2.3165	4.6203	-0.2106
H	-3.8238	3.727	-0.4801
C	-1.775	2.801	-2.2068
H	-2.7102	2.9564	-2.7545
H	-1.1811	3.714	-2.3213
H	-1.235	1.9806	-2.6901
H	6.063	3.5936	-1.0916
H	6.0491	1.8664	-0.7191
H	5.6009	3.0456	0.5245
H	4.3393	1.3687	-2.6783
H	4.6264	3.0693	-3.0823
H	2.9754	2.4748	-2.898
H	-8.3528	1.1162	-1.5086
H	-6.7524	1.8543	-1.7057
H	-7.4004	1.5613	-0.0867
H	-8.5693	-1.2977	-0.6953
H	-7.6263	-0.9428	0.7577
H	-7.1204	-2.2494	-0.3208
H	-4.0352	2.7164	2.3268
H	-3.7986	2.2175	4.0111
H	-5.2063	1.6506	3.1182
H	-4.5424	-0.8841	3.5244
H	-3.2734	-0.2246	4.5719
H	-2.8477	-1.3515	3.2695
H	-2.4886	0.1398	-3.7499
H	-0.7767	-0.3047	-3.829
H	-1.3106	0.9833	-2.731
H	-2.9717	-2.4301	-3.2504
H	-2.2182	-3.1661	-1.8288
H	-1.2364	-2.7518	-3.245
H	1.4805	3.8514	-1.1666
H	1.3458	3.674	0.5899
H	0.026	3.1434	-0.4609
H	3.4488	0.4059	2.9067
H	3.9634	-1.2485	3.2092
H	2.4184	-0.9399	2.4063
H	6.8198	-0.2184	0.3133
H	6.6225	-0.9841	1.8951
H	6.2931	0.738	1.7021
H	5.7982	-3.3135	1.219
H	4.6871	-4.4404	0.4369
H	4.1531	-3.5166	1.8438
H	2.7003	-3.6257	-0.884
H	2.402	-1.896	-1.1115
H	2.1211	-2.6482	0.4683
H	4.7899	-1.5863	-2.0119
H	5.0146	-3.332	-1.8208
H	6.1711	-2.2107	-1.0961

3-2. DFT-optimization

DFT optimizations on the B3LYP/6-31G(d,p) level[11-16] were performed on Gaussian G16.B01 and Gaussview 6.0.16 was used to edit the job-files.[17, 18] Due to limitation of computational resources, not all structures found by CREST could be optimized on DFT level. The structures found by CREST were sorted by energy and for all four diketides the first 150 structures were optimized on DFT level since structures with low DFT energies would most likely be found in this subset. Additionally, every 20th structure for *syn*-carbamate **10**, every 10th for *anti*-carbamate **12** and every 25th for both TIB ester (**9** and **11**), depending on the total amount of structures found by CREST for each diketide, were also optimized to check the higher energies for structures with low DFT energy. Default convergence criteria were used for optimizations and frequency calculations at the end of the optimization. Structures were only considered if both optimization and frequency analysis met the convergence criteria. To model dispersion interactions gd3bj-corrections were used and to account for the solvent environment a polarizable continuum model (PCM) was used.[19-21] The route section used for all DFT optimizations is # opt=(calcfc,recalc=20) freq b3lyp/6-31g(d,p) empiricaldispersion=gd3bj scfr=(solvent=diethylether)". If the first optimization and frequency calculation for a specific structure including PCM did not converge, the optimization was restarted up to two times. If the convergence criteria of optimization and frequency calculation were still not met, the optimization was started without the PCM, which usually leads to convergence. Corrections due to the solvent were then added in an additional frequency calculation including PCM without optimization. Additional information on the empirical dispersion and the solvent background can be found on the gaussian website (https://gaussian.com/dft/ and https://gaussian.com/scrf/).

Coordinates of **10-c1** (0.00 kJ/mol).

	Coordinates		
Atom	x / A	y / A	z / A
H	4.4214	-2.7572	0.9622
H	5.8001	-2.3454	-0.0646
H	4.2606	-0.9473	-2.3625
H	5.7229	-0.3109	-1.5997
H	1.9003	2.6024	3.2209
H	2.5295	1.266	2.2493
H	4.2629	0.6892	-1.686
H	-1.2961	-1.6999	0.1661
H	-1.1051	1.2536	-2.8062
H	0.5998	-1.4134	1.7059

Coordinates of **10-c2** (1.59 kJ/mol).

	Coordinates		
Atom	x / A	y / A	z / A
H	-4.9608	-1.8632	-0.3057
H	-4.4967	-0.844	1.0657
H	-1.7658	-3.8942	-0.4012
H	-3.3118	-3.6279	-1.2233
H	-1.7597	2.3801	-2.5719
H	-3.0541	3.5462	-2.2635
H	-3.2803	-4.3437	0.3915
H	4.463	0.5613	-1.1641
H	0.5968	0.8003	2.8857
H	-4.1195	-0.3944	-2.1755

H	4.4352	1.0318	0.8523	H	-1.1122	-2.4286	1.653
C	-2.5244	0.1772	-0.9653	C	2.072	-0.4789	0.9054
O	-1.2391	-0.1548	-1.2873	O	1.3669	0.679	0.9676
C	-0.4903	0.8514	-1.999	C	0.2327	0.6853	1.8594
C	-0.0303	1.9731	-1.0688	C	-0.6635	1.8526	1.4694
C	0.6757	1.4127	0.1807	C	-1.269	1.656	0.0616
C	1.0275	2.4818	1.2332	C	-2.0101	2.8989	-0.4705
C	-0.1955	3.3274	1.6156	C	-1.1038	4.1386	-0.4759
C	1.6313	1.8372	2.4858	C	-2.565	2.6469	-1.8758
O	-3.096	1.1437	-1.4606	O	1.7476	-1.4862	1.5313
H	-3.8821	-3.294	0.5463	H	4.4199	2.7933	-0.1807
H	0.4685	-1.9071	-1.4547	H	0.2178	-1.7481	-0.3506
H	5.8701	-0.0069	0.8938	H	-2.608	-3.0108	2.3973
H	4.493	-0.3626	1.9431	H	-2.2943	-1.2736	2.2857
H	1.8796	-2.9703	-1.3501	H	0.1956	-0.5697	-1.6708
H	1.8975	-1.5825	-2.4447	H	-0.388	-2.2109	-1.9478
H	-2.8807	-3.2508	-0.9172	H	3.1258	2.3192	0.935
C	0.8401	2.9593	-1.8538	C	-1.7377	2.0575	2.5438
O	1.8704	0.7366	-0.2204	O	-2.1582	0.5373	0.0993
Si	2.2797	-0.8816	-0.0681	Si	-2.1109	-0.9064	-0.7563
C	1.5613	-1.928	-1.4621	C	-0.3526	-1.3985	-1.2137
C	1.6695	-1.5937	1.5673	C	-3.1134	-0.7874	-2.3499
C	4.1813	-0.8878	-0.1675	C	-2.9067	-2.1959	0.3989
C	4.7729	-0.0045	0.9461	C	-2.1834	-2.2251	1.7576
C	4.7036	-2.328	-0.0056	C	-4.3883	-1.8418	0.6276
C	4.6266	-0.332	-1.5338	C	-2.8072	-3.5896	-0.2517
H	2.1905	-1.1668	2.428	H	-3.2162	-1.78	-2.8022
H	1.8221	-2.6782	1.5808	H	-2.6215	-0.1407	-3.0824
H	4.3242	-2.9913	-0.7904	H	-4.8532	-2.5612	1.3154
H	0.36	0.3146	-2.4186	H	-0.2894	-0.2665	1.789
H	-2.2381	-3.9387	0.5873	H	4.731	1.5747	1.0698
H	-0.012	0.7002	0.6579	H	-0.442	1.4456	-0.6299
H	1.7877	3.1382	0.7907	H	-2.861	3.0879	0.1959
H	0.0462	3.988	2.454	H	-0.8326	4.466	0.531
H	-1.0301	2.6881	1.9292	H	-1.6087	4.9752	-0.9684
H	-0.5465	3.9542	0.7921	H	-0.1761	3.9424	-1.0274
H	0.9107	1.1592	2.959	H	-3.2953	1.8383	-1.8758
N	-3.0704	-0.6773	-0.0599	N	3.1421	-0.3872	0.0688
C	-2.3042	-1.7747	0.5665	C	3.5646	0.8544	-0.616
C	-2.8649	-3.1442	0.1708	C	3.9827	1.953	0.3677
H	-5.2847	-1.6383	-1.3208	H	5.3934	-1.1856	1.3705
H	-6.4785	-0.6262	-0.4866	H	5.8897	-2.4332	0.2114
H	-5.2949	0.1181	-1.5816	H	5.9261	-0.7332	-0.2605
C	-5.4438	-0.6684	-0.8407	C	5.3677	-1.4774	0.3169
C	-2.2158	-1.5937	2.0863	C	2.5555	1.3494	-1.6583
H	-1.7872	-0.6181	2.3345	H	2.2754	0.5413	-2.3395
H	-3.1945	-1.67	2.5695	H	1.6532	1.7341	-1.1825

H	-1.5753	-2.37	2.5153	H	3.0037	2.1545	-2.2494
C	-4.4838	-0.4896	0.3396	C	3.9216	-1.6191	-0.1675
H	-4.6824	-1.301	1.0436	H	3.4318	-2.3756	0.4452
C	-4.6997	0.8309	1.0868	C	3.8307	-2.056	-1.6331
H	-5.7284	0.8854	1.4568	H	2.7855	-2.1766	-1.9316
H	-4.5186	1.6789	0.4254	H	4.3426	-3.0133	-1.7703
H	-4.0234	0.9035	1.9436	H	4.3003	-1.3315	-2.3068
H	-0.9373	2.4843	-0.7331	H	-0.023	2.7402	1.4335
H	1.0598	3.8537	-1.2649	H	-1.2854	2.1461	3.5363
H	0.3338	3.2826	-2.7689	H	-2.4341	1.2152	2.5547
H	1.7907	2.495	-2.1307	H	-2.3144	2.9675	2.3602

Coordinates of **10-c3** (2.50 kJ/mol).

	Coordinates		
Atom	x / A	y / A	z / A
H	-2.4092	-2.7812	2.0843
H	-4.1766	-2.7185	2.1232
H	-4.5819	-0.2754	0.2616
H	-5.4909	-1.796	0.1722
H	-3.7074	2.8422	-2.1674
H	-3.5068	1.1723	-1.6226
H	-4.6724	-1.1692	-1.2639
H	4.1503	0.1637	-1.7338
H	0.9668	1.4453	2.6913
H	-0.9407	-0.3449	-2.5943
H	-4.2725	-4.0092	-0.0731
C	2.3662	-0.0869	1.0684
O	1.4032	0.8225	0.7624
C	0.4424	1.0697	1.8083
C	-0.5664	2.0912	1.3051
C	-1.2902	1.6072	0.0291
C	-2.311	2.6247	-0.5196
C	-1.6634	3.9928	-0.7757
C	-2.9809	2.1114	-1.798
O	2.3988	-0.6696	2.1475
H	3.6452	2.5332	-1.9044
H	0.6696	-1.6199	-0.302
H	-2.5088	-4.0741	-0.1487
H	-3.4305	-3.4158	-1.5098
H	-0.2802	-3.0881	-0.087
H	-0.0494	-1.9792	1.2703
H	2.6175	2.4686	-0.4606
C	-1.5403	2.4159	2.4453
O	-1.9548	0.377	0.317
Si	-1.7756	-1.1404	-0.3754

C	-0.2144	-2.0301	0.1915
C	-1.7003	-1.0573	-2.258
C	-3.3267	-2.067	0.2211
C	-3.3809	-3.4678	-0.4166
C	-3.2792	-2.2029	1.755
C	-4.5874	-1.2775	-0.1766
H	-2.6527	-0.7685	-2.7092
H	-1.4216	-2.0404	-2.6531
H	-3.2341	-1.2226	2.2401
H	-0.0489	0.1339	2.0786
H	4.3625	2.1804	-0.3217
H	-0.5295	1.4491	-0.7475
H	-3.0936	2.7415	0.2404
H	-2.3777	4.6671	-1.2581
H	-0.7985	3.896	-1.4433
H	-1.3243	4.4758	0.1442
H	-2.2415	1.9468	-2.5905
N	3.2504	-0.2707	0.0466
C	3.2667	0.5236	-1.2015
C	3.4825	2.0206	-0.9511
H	5.7255	0.0962	1.1161
H	6.4396	-1.426	0.5508
H	5.9864	-0.1818	-0.6168
C	5.6937	-0.6579	0.3249
C	2.0611	0.2426	-2.1043
H	2.1891	0.7519	-3.0647
H	1.9611	-0.8291	-2.2959
H	1.1391	0.5998	-1.646
C	4.301	-1.2903	0.2411
H	4.0732	-1.7384	1.208
C	4.2152	-2.3855	-0.8265
H	4.4332	-2.0025	-1.8289
H	3.2171	-2.8323	-0.8381
H	4.9433	-3.1731	-0.6105
H	-0.0063	2.9956	1.0414
H	-2.1893	1.5591	2.6467
H	-2.1743	3.2713	2.2008
H	-0.9979	2.6591	3.3643

Coordinates of **12-c1** (0.00 kJ/mol).

Atom	Coordinates x / A	y / A	z / A
H	1.6109	-3.9777	0.1163
H	3.1664	-3.8172	0.9481
H	2.2173	-1.1987	-2.3882
H	1.005	-2.3641	-1.8364
H	4.0002	2.2448	-0.5867
H	4.0162	3.9059	0.0303
H	2.4811	-2.9327	-2.6288
H	-4.4572	0.5791	1.2963
H	-0.6227	1.004	-2.774
H	4.0935	-0.6682	2.083
H	4.4442	-0.9367	-1.1633
C	-2.128	-0.3466	-0.8951
O	-1.3812	0.7852	-0.8589
C	-0.2525	0.8244	-1.759
C	0.6727	1.9503	-1.3178
C	1.3538	1.6279	0.0306
C	2.2385	2.7731	0.5629
C	2.7873	2.4531	1.9564
C	3.3795	3.1257	-0.3986
O	-1.8461	-1.3021	-1.6156
H	-4.3354	2.894	0.5203
H	0.2997	-2.343	1.7904
H	4.7393	-2.6455	-1.5245
H	4.8752	-2.058	0.1377
H	-0.2952	-1.779	0.221
H	-0.2304	-0.6712	1.5985
H	-3.0881	2.4669	-0.6657
C	-0.0626	3.2977	-1.316
O	2.1374	0.4452	-0.1405
Si	2.0587	-1.0301	0.6557
C	0.2889	-1.4937	1.0986
C	3.0707	-1.0196	2.247
C	2.8089	-2.2723	-0.5777
C	4.3016	-1.9561	-0.7897
C	2.662	-3.7003	-0.0175
C	2.0811	-2.1836	-1.9318
H	3.1267	-2.0333	2.6593
H	2.6124	-0.3795	3.0066
H	3.1079	-4.428	-0.7088
H	0.2521	-0.138	-1.7447
H	-4.7258	1.8037	-0.8226
H	0.5641	1.4461	0.7732
H	1.5829	3.6464	0.6653

Coordinates of **12-c2** (1.13 kJ/mol).

Atom	Coordinates x / A	y / A	z / A
H	4.1101	-2.639	-2.2765
H	3.2046	-1.1154	-2.3026
H	4.6559	-1.2956	1.1908
H	4.5973	-0.3249	-0.2882
H	3.9147	2.0165	-0.8861
H	4.264	3.542	-0.0558
H	5.4545	-1.876	-0.2757
H	-4.1705	0.0336	1.7439
H	-0.9479	1.6136	-2.5928
H	2.6639	-0.8266	2.6468
H	3.3512	-3.5222	1.316
C	-2.3687	-0.0133	-1.0589
O	-1.4399	0.9122	-0.7026
C	-0.4481	1.2018	-1.7111
C	0.546	2.2017	-1.1409
C	1.331	1.6253	0.0566
C	2.4216	2.5834	0.5844
C	3.1017	2.022	1.8372
C	3.465	2.9295	-0.4849
O	-2.3699	-0.5509	-2.1618
H	-3.7841	2.4371	1.9931
H	-0.6941	-1.5856	0.2665
H	4.181	-4.0539	-0.1516
H	2.4161	-4.08	-0.0805
H	0.2248	-3.0648	-0.0009
H	-0.0064	-1.9154	-1.3251
H	-2.8101	2.4657	0.5112
C	-0.1302	3.5373	-0.8023
O	1.929	0.3897	-0.3345
Si	1.7563	-1.1469	0.3167
C	0.1747	-1.999	-0.2509
C	1.7109	-1.1212	2.2014
C	3.2836	-2.0758	-0.3362
C	3.3021	-3.5114	0.2215
C	3.2274	-2.1228	-1.875
C	4.5679	-1.3472	0.0997
H	1.4576	-2.1213	2.57
H	0.9408	-0.4358	2.5697
H	2.3418	-2.6596	-2.2322
H	0.0498	0.2773	-2.0006
H	-4.5385	2.0798	0.4286
H	0.6185	1.453	0.8754
H	1.907	3.5053	0.8814

H	1.9884	2.1451	2.6403	H	3.679	1.1248	1.6007
H	3.2783	3.3315	2.3873	H	2.3699	1.7616	2.6082
H	3.5229	1.6473	1.9117	H	3.7899	2.7601	2.2613
H	3.0115	3.4915	-1.3618	H	3.0338	3.4894	-1.32
N	-3.1868	-0.296	-0.0404	N	-3.2565	-0.2722	-0.0562
C	-3.5556	0.89	0.7631	C	-3.3153	0.4696	1.2221
C	-3.9466	2.0878	-0.1098	C	-3.6283	1.9567	1.0221
H	-5.4784	-0.8829	-1.3834	H	-5.9945	-0.3375	0.6147
H	-6.0116	-2.2154	-0.3411	H	-5.7508	0.046	-1.1005
H	-5.9781	-0.5677	0.2896	H	-6.3953	-1.5347	-0.619
C	-5.4541	-1.2741	-0.3626	C	-5.6834	-0.748	-0.3516
C	-2.5159	1.2483	1.8309	C	-2.0909	0.2353	2.1135
H	-2.9202	2.0208	2.4927	H	-1.9143	-0.8345	2.2551
H	-2.2702	0.3731	2.4384	H	-1.1967	0.6797	1.6765
H	-1.5992	1.6275	1.3789	H	-2.2575	0.6873	3.0963
C	-4.0102	-1.515	0.0874	C	-4.2643	-1.3228	-0.3035
H	-3.5551	-2.2268	-0.6009	H	-4.0212	-1.7091	-1.2931
C	-3.9217	-2.0933	1.5034	C	-4.1265	-2.4676	0.705
H	-4.3564	-1.4196	2.2494	H	-3.1105	-2.8717	0.6884
H	-2.879	-2.2806	1.7748	H	-4.8228	-3.273	0.4524
H	-4.4685	-3.0397	1.5559	H	-4.3528	-2.1457	1.7269
H	1.4668	1.9794	-2.0746	H	1.2649	2.3651	-1.9533
H	-0.7584	3.3681	-0.4746	H	0.6002	4.3363	-0.6556
H	-0.6443	3.4139	-2.236	H	-0.7292	3.4564	0.1101
H	0.6303	4.1397	-1.2543	H	-0.7986	3.8446	-1.6126

Coordinates of **12-c3** (5.34 kJ/mol).

Atom	Coordinates x / A	y / A	z / A
H	-4.0276	-0.9186	-2.2833
H	-4.4132	0.475	-1.261
H	-3.6659	-3.2418	-1.1908
H	-5.2284	-3.0864	-0.3794
H	-0.8289	1.768	-2.7254
H	-1.9627	3.1248	-2.797
H	-3.7732	-3.4159	0.5682
H	4.0758	-0.9267	-1.6297
H	1.4308	3.0894	1.2385
H	-0.0623	-1.2889	-1.3698
H	-4.3313	-1.3704	2.0339
C	2.2382	-0.0219	0.9943
O	2.0207	1.2702	0.6318
C	1.2217	2.0632	1.5377
C	-0.2725	1.7251	1.4747
C	-0.8273	1.4898	0.0507
C	-1.1873	2.7212	-0.8016
C	-0.0408	3.7317	-0.9404
C	-1.6484	2.2672	-2.1934
O	1.8268	-0.4888	2.0543
H	5.3126	0.6032	-0.1314
H	-1.4419	-2.9093	1.441
H	-5.7623	-1.0438	1.0477
H	-4.5447	0.2214	1.2891
H	-1.8761	-1.5292	2.4601
H	-0.2978	-1.5768	1.6656
H	5.0817	1.2838	-1.7519
C	-1.0856	2.7368	2.2873
O	-2.0236	0.7087	0.1801
Si	-2.0905	-0.9623	0.0354
C	-1.3575	-1.8213	1.5413
C	-1.1278	-1.5062	-1.4919
C	-3.9483	-1.3389	-0.1284
C	-4.6839	-0.8533	1.1351
C	-4.5233	-0.6094	-1.3564
C	-4.1581	-2.8561	-0.2911
H	-1.4712	-0.9987	-2.398
H	-1.2251	-2.586	-1.6466
H	-5.5939	-0.8297	-1.4668
H	1.5833	1.9111	2.5574
H	4.091	1.8615	-0.3996
H	-0.0756	0.9166	-0.5061
H	-2.0327	3.2163	-0.3066

H	0.8777	3.248	-1.2906
H	0.1837	4.2437	-0.0024
H	-0.3067	4.4999	-1.6733
H	-2.4834	1.5684	-2.1265
N	2.9395	-0.7124	0.0532
C	3.5327	-0.1036	-1.1598
C	4.566	0.9815	-0.8351
H	4.9033	-3.4017	0.8732
H	5.3252	-2.1671	-0.316
H	5.0916	-1.7138	1.3837
C	4.7286	-2.3618	0.5812
C	2.4809	0.3647	-2.1705
H	1.911	1.2103	-1.7847
H	2.9735	0.6751	-3.0972
H	1.7859	-0.4446	-2.4096
C	3.2333	-2.133	0.3387
H	2.7039	-2.3425	1.2678
C	2.6692	-3.0488	-0.7512
H	1.5939	-2.893	-0.8699
H	2.8367	-4.0949	-0.4776
H	3.15	-2.8823	-1.7208
H	-0.3717	0.7544	1.9657
H	-2.1443	2.4662	2.2641
H	-0.9862	3.7553	1.8988
H	-0.7623	2.7464	3.3332

Coordinates of **9-c1** (0.00 kJ/mol).

Atom	x / A	y / A	z / A
C	1.3172	1.0703	2.3857
H	0.9909	1.6188	3.272
H	1.9075	0.2053	2.686
C	2.1144	1.9826	1.4519
H	1.5333	2.9016	1.3348
C	3.4546	2.3112	2.1185
H	3.991	3.0918	1.5736
H	3.3058	2.6667	3.1433
H	4.0903	1.422	2.1511
C	2.2752	1.364	0.0501
H	1.2699	1.1765	-0.3465
C	2.9968	2.2805	-0.9583
H	4.0511	2.3241	-0.6567
C	2.9244	1.6905	-2.3713
H	3.4985	2.3015	-3.0752
H	3.3202	0.6764	-2.4049
H	1.8871	1.6639	-2.7239
C	2.4248	3.7057	-0.9674
H	1.3439	3.694	-1.1517
H	2.5982	4.2377	-0.0289
H	2.8861	4.2907	-1.769
O	2.9824	0.1243	0.15
Si	2.4035	-1.425	-0.1522
C	0.9859	-1.3733	-1.3918
H	0.6499	-2.3906	-1.6189
H	0.1276	-0.8334	-0.9794
H	1.2721	-0.8946	-2.3325
C	1.7934	-2.2548	1.4264
H	0.8801	-1.7744	1.7822
H	1.5679	-3.31	1.2372
H	2.5403	-2.214	2.2253
C	3.9057	-2.4031	-0.8072
C	4.9874	-2.463	0.2892
H	5.3058	-1.4595	0.5898
H	5.8735	-3	-0.076
H	4.6329	-2.9862	1.1834
C	3.4679	-3.8356	-1.1698
H	3.0489	-4.3685	-0.3097
H	2.7166	-3.8421	-1.9671
H	4.3286	-4.4171	-1.5262
C	4.5046	-1.7331	-2.0557
H	4.8824	-0.7326	-1.8271
H	5.345	-2.3266	-2.4411

Coordinates of **9-c2** (4.82 kJ/mol).

Atom	x / A	y / A	z / A
C	-1.2946	1.2708	-2.1984
H	-0.9642	1.7859	-3.1035
H	-1.7842	0.3405	-2.4938
C	-2.2171	2.1501	-1.3608
H	-1.7093	3.1132	-1.2388
C	-3.5307	2.3613	-2.1202
H	-4.1616	3.1038	-1.6254
H	-3.3438	2.7106	-3.1408
H	-4.0911	1.4241	-2.1743
C	-2.4248	1.5762	0.0549
H	-1.432	1.4499	0.5007
C	-3.2372	2.4869	0.9973
H	-4.2862	2.4266	0.6798
C	-3.1346	1.9753	2.439
H	-3.4266	0.9287	2.5164
H	-2.1041	2.0682	2.8036
H	-3.7772	2.5575	3.1072
C	-2.7857	3.9535	0.9429
H	-3.3092	4.5359	1.7074
H	-1.7112	4.0398	1.1429
H	-2.9872	4.4226	-0.0234
O	-3.0732	0.3019	-0.0298
Si	-2.4282	-1.2164	0.289
C	-0.9584	-1.077	1.4575
H	-0.588	-2.0735	1.72
H	-0.134	-0.5352	0.9845
H	-1.2183	-0.5608	2.3865
C	-1.8894	-2.0599	-1.3091
H	-1.6384	-3.1095	-1.1253
H	-2.6841	-2.0346	-2.0609
H	-1.0056	-1.5868	-1.7389
C	-3.8498	-2.2452	1.0407
C	-4.406	-1.5826	2.3126
H	-5.1874	-2.2121	2.7598
H	-4.8523	-0.6093	2.0909
H	-3.6298	-1.436	3.072
C	-4.9887	-2.3706	0.01
H	-4.6645	-2.8952	-0.8947
H	-5.8285	-2.9373	0.4347
H	-5.3667	-1.3871	-0.2877
C	-3.3291	-3.6514	1.3982
H	-2.9355	-4.1805	0.5241
H	-2.5356	-3.6124	2.1524

H	3.7703	-1.6432	-2.864	H	-4.1426	-4.2632	1.8107
O	0.1595	0.4952	1.7318	O	-0.1419	0.9457	-1.3879
C	-0.9247	1.2792	1.5573	C	0.9208	0.3771	-1.9937
O	-1.0033	2.4201	1.9704	O	0.9454	0.1107	-3.1807
C	-3.9046	-0.7659	-0.7722	C	4.0019	-0.4702	0.8905
C	-3.6299	-1.166	0.5347	C	3.4565	-1.4813	0.1004
C	-2.6658	-0.524	1.3194	C	2.4643	-1.2301	-0.8522
C	-1.9733	0.5635	0.7616	C	2.0091	0.0958	-1.0061
C	-2.2317	1.0005	-0.5548	C	2.5517	1.1444	-0.2309
C	-3.1962	0.32	-1.298	C	3.5394	0.8352	0.7049
H	-4.1828	-2.0022	0.9529	H	3.8144	-2.4975	0.2352
H	-3.3992	0.6404	-2.314	H	3.9618	1.636	1.3033
C	-2.4407	-0.9784	2.7551	C	1.9225	-2.3894	-1.6787
H	-1.6021	-0.4117	3.1687	H	1.0364	-2.049	-2.215
C	-1.5031	2.1985	-1.1498	C	2.1231	2.5988	-0.3934
H	-0.5713	2.3375	-0.5937	H	1.4522	2.6702	-1.2517
C	-4.9428	-1.4967	-1.6056	C	5.0695	-0.7824	1.9241
H	-5.3628	-2.2908	-0.9758	H	5.2655	-1.8606	1.8718
C	-6.0956	-0.5666	-2.0172	C	6.3863	-0.0513	1.615
H	-6.5659	-0.1103	-1.1411	H	6.7474	-0.297	0.6119
H	-5.7387	0.241	-2.6649	H	6.256	1.0347	1.6677
H	-6.861	-1.1225	-2.5684	H	7.1602	-0.3303	2.3374
C	-4.3029	-2.1634	-2.8349	C	4.5795	-0.4638	3.3465
H	-3.8774	-1.4146	-3.5113	H	4.3791	0.6065	3.4626
H	-3.4982	-2.8434	-2.5398	H	3.6561	-1.0042	3.5752
H	-5.0492	-2.7354	-3.3959	H	5.3357	-0.7448	4.0868
C	-3.6752	-0.6697	3.6201	C	2.9498	-2.8082	-2.744
H	-3.9178	0.3966	3.5902	H	3.8817	-3.1489	-2.2801
H	-4.5511	-1.2243	3.2677	H	2.5566	-3.626	-3.357
H	-3.494	-0.9525	4.6622	H	3.1817	-1.9658	-3.4013
C	-2.0637	-2.4661	2.834	C	1.496	-3.5797	-0.806
H	-2.8796	-3.1044	2.4801	H	2.3485	-4.0466	-0.303
H	-1.1798	-2.6822	2.2279	H	0.7804	-3.269	-0.0395
H	-1.847	-2.7487	3.8692	H	1.0202	-4.3462	-1.4262
C	-2.3351	3.4778	-0.9544	C	3.3221	3.5172	-0.6821
H	-2.5306	3.6463	0.1071	H	2.9771	4.5391	-0.8699
H	-1.8023	4.348	-1.3523	H	4.0183	3.5535	0.1616
H	-3.2946	3.3966	-1.4765	H	3.8767	3.1757	-1.5611
C	-1.1193	2.001	-2.6225	C	1.3398	3.0776	0.8398
H	-0.4832	2.8267	-2.956	H	0.4732	2.4376	1.0224
H	-0.5722	1.0652	-2.7693	H	1.9686	3.0585	1.7363
H	-1.9979	1.9852	-3.2749	H	0.9865	4.1045	0.6979

Coordinates of **9-c3** (5.00 kJ/mol).

Atom	Coordinates x / A	y / A	z / A
C	1.3731	1.4468	2.0651
H	1.0855	2.0677	2.9175
H	1.7637	0.5033	2.4517
C	2.3904	2.1524	1.1741
H	1.9846	3.1469	0.9567
C	3.7113	2.2961	1.9368
H	3.5511	2.7390	2.9253
H	4.1777	1.3162	2.0680
H	4.4156	2.9346	1.3977
C	2.5575	1.4338	-0.1800
H	1.5620	1.3602	-0.6317
C	3.4639	2.1728	-1.1850
H	4.4980	2.0410	-0.8422
C	3.3285	1.5417	-2.5758
H	2.3163	1.6978	-2.9689
H	4.0316	1.9949	-3.2819
H	3.5196	0.4697	-2.5510
C	3.1553	3.6743	-1.2733
H	3.3920	4.2101	-0.3506
H	3.7389	4.1319	-2.0782
H	2.0960	3.8439	-1.4998
O	3.0821	0.1206	0.0339
Si	2.3093	-1.3583	-0.1505
C	0.8731	-1.2057	-1.3592
H	0.4133	-2.1850	-1.5294
H	0.0956	-0.5460	-0.9625
H	1.1910	-0.8129	-2.3297
C	1.6745	-1.9951	1.5069
H	1.3216	-3.0273	1.4133
H	2.4620	-1.9817	2.2666
H	0.8397	-1.4013	1.8807
C	3.6477	-2.5712	-0.7668
C	3.0142	-3.9550	-1.0101
H	3.7787	-4.6701	-1.3415
H	2.2429	-3.9193	-1.7873
H	2.5579	-4.3654	-0.1031
C	4.2848	-2.0764	-2.0762
H	3.5404	-1.9358	-2.8681
H	4.8073	-1.1275	-1.9279
H	5.0189	-2.8063	-2.4436
C	4.7509	-2.6948	0.3024
H	5.5500	-3.3624	-0.0471
H	5.2011	-1.7217	0.5237

H	4.3661	-3.1096	1.2398
O	0.2050	1.1693	1.2621
C	-0.9049	0.7252	1.8970
O	-0.9543	0.5475	3.0970
C	-3.9965	-0.1259	-0.9744
C	-3.5493	-1.1036	-0.0829
C	-2.5609	-0.8460	0.8677
C	-2.0008	0.4501	0.9157
C	-2.4451	1.4636	0.0423
C	-3.4379	1.1483	-0.8892
H	-3.9805	-2.0980	-0.1306
H	-3.7904	1.9188	-1.5688
C	-2.1374	-1.9605	1.8161
H	-1.2188	-1.6586	2.3189
C	-1.9093	2.8902	0.0918
H	-1.2585	2.9853	0.9633
C	-5.0627	-0.4398	-2.0089
H	-5.2456	0.4818	-2.5760
C	-4.5802	-1.5114	-3.0007
H	-3.6493	-1.2066	-3.4877
H	-4.3941	-2.4627	-2.4913
H	-5.3330	-1.6889	-3.7758
C	-6.3878	-0.8527	-1.3477
H	-7.1611	-1.0225	-2.1040
H	-6.2708	-1.7796	-0.7765
H	-6.7426	-0.0797	-0.6598
C	-3.1970	-2.1483	2.9150
H	-4.1649	-2.4282	2.4848
H	-2.8933	-2.9366	3.6118
H	-3.3248	-1.2211	3.4791
C	-1.8413	-3.2769	1.0820
H	-1.4375	-4.0133	1.7841
H	-2.7407	-3.7122	0.6349
H	-1.1075	-3.1273	0.2847
C	-3.0397	3.9186	0.2609
H	-2.6214	4.9241	0.3747
H	-3.7071	3.9362	-0.6064
H	-3.6444	3.6971	1.1450
C	-1.0573	3.1980	-1.1503
H	-0.2375	2.4822	-1.2448
H	-1.6596	3.1439	-2.0634
H	-0.6315	4.2053	-1.0878

Coordinates of **11-c1** (0.00 kJ/mol).

Atom	Coordinates x / A	y / A	z / A
C	1.5925	-1.226	0.0772
C	2.5602	-0.7784	0.9912
C	3.6205	-0.0072	0.5089
C	3.7329	0.3257	-0.8426
C	2.7537	-0.1366	-1.725
C	1.6712	-0.9051	-1.292
C	2.4925	-1.1238	2.4723
H	4.3722	0.3469	1.2077
C	4.8687	1.2019	-1.341
H	2.8303	0.1334	-2.7731
C	0.5963	-1.3699	-2.2665
C	0.4268	-2.0372	0.5543
O	-0.4454	-1.2777	1.2437
C	-1.6732	-1.9201	1.6591
C	-2.6852	-2.0574	0.5177
C	-3.3818	-0.7264	0.1559
C	-4.2104	-0.8163	-1.1469
C	-5.0328	0.4566	-1.3729
C	-3.3373	-1.1061	-2.3748
O	0.2602	-3.2233	0.3352
C	6.2455	0.5948	-1.0283
C	4.7486	2.627	-0.7744
C	0.3255	-0.3653	-3.3943
C	0.9439	-2.7551	-2.8398
C	2.3894	0.141	3.3397
C	3.6899	-1.9926	2.8922
C	-3.7097	-3.1329	0.9094
H	-2.1289	-2.4151	-0.3528
O	-2.4249	0.322	0.0186
Si	-2.2889	1.7252	0.9283
C	-1.6129	1.3921	2.6561
C	-3.9625	2.5703	1.1417
C	-1.0931	2.8248	-0.0609
C	0.3265	2.2329	-0.0125
C	-1.5603	2.9045	-1.5258
C	-1.0746	4.2414	0.5449
H	1.5865	-1.7109	2.6485
H	4.7726	1.2674	-2.432
H	-0.3376	-1.4654	-1.7021
H	-1.4239	-2.9065	2.0554
H	-2.0554	-1.295	2.4666
H	-4.0771	-0.4896	0.9786
H	-4.9179	-1.6442	-1.019

Coordinates of **11-c2** (1.18 kJ/mol).

Atom	Coordinates x / A	y / A	z / A
C	1.9776	0.8417	0.4647
C	2.0785	1.1211	-0.9077
C	2.9851	0.3745	-1.6661
C	3.7688	-0.6303	-1.096
C	3.635	-0.8877	0.2725
C	2.7421	-0.1709	1.0705
C	1.2443	2.2301	-1.5326
H	3.0791	0.5696	-2.7296
C	4.7307	-1.4379	-1.9504
H	4.2344	-1.6708	0.7239
C	2.6351	-0.4273	2.5675
C	1.0005	1.6186	1.2986
O	-0.1564	0.937	1.3896
C	-1.2736	1.485	2.1347
C	-2.276	2.1635	1.1963
C	-2.5065	1.2931	-0.0586
C	-3.5136	1.8893	-1.0615
C	-3.4439	1.1591	-2.4074
C	-4.9424	1.9148	-0.509
O	1.2065	2.6964	1.8203
C	4.3191	-2.9187	-2.0045
C	6.1833	-1.2842	-1.4712
C	2.7619	-1.9094	2.9425
C	3.665	0.4328	3.3228
C	0.7768	1.9167	-2.9595
C	2.0012	3.5685	-1.47
C	-1.8541	3.6003	0.8544
H	-3.2182	2.2024	1.7577
O	-2.9379	-0.0074	0.35
Si	-2.171	-1.4902	0.1511
C	-1.2495	-2.0042	1.715
C	-0.9471	-1.4249	-1.278
C	-3.573	-2.7502	-0.1563
C	-2.9661	-4.1497	-0.3775
C	-4.5015	-2.7877	1.0733
C	-4.4043	-2.3691	-1.3929
H	0.3401	2.3393	-0.9246
H	4.6717	-1.0377	-2.9703
H	1.6386	-0.1047	2.8893
H	-1.7217	0.6171	2.6154
H	-0.8945	2.1762	2.889
H	-1.5443	1.2109	-0.578
H	-3.1953	2.9218	-1.2463

H	-4.3793	1.3126	-1.5605	H	-2.4291	1.1902	-2.8186
H	-5.6591	0.6909	-0.5064	H	-4.1156	1.625	-3.1355
H	-5.6887	0.3392	-2.2413	H	-3.7357	0.1123	-2.3071
H	-3.9477	-1.1082	-3.2833	H	-5.2723	0.903	-0.2587
H	-2.8362	-2.0763	-2.3141	H	-5.6345	2.3288	-1.2489
H	-2.5704	-0.3348	-2.4823	H	-5.0193	2.5261	0.3958
H	6.3367	-0.4121	-1.4464	H	4.9838	-3.4808	-2.6688
H	6.4115	0.5257	0.0519	H	3.2936	-3.0287	-2.3697
H	7.044	1.2152	-1.448	H	4.3722	-3.3771	-1.0113
H	3.777	3.0663	-1.0202	H	6.8666	-1.8264	-2.1329
H	5.5332	3.274	-1.1804	H	6.3099	-1.6856	-0.4602
H	4.8466	2.6229	0.3164	H	6.4835	-0.2323	-1.4539
H	1.1649	-0.3031	-4.0943	H	3.7692	-2.2929	2.7518
H	0.1355	0.6373	-3.0014	H	2.0513	-2.5228	2.3807
H	-0.5519	-0.68	-3.9672	H	2.5604	-2.0428	4.0098
H	0.1504	-3.1028	-3.5103	H	4.6833	0.1583	3.0274
H	1.0667	-3.4852	-2.0376	H	3.5717	0.2882	4.4042
H	1.8762	-2.7067	-3.4133	H	3.5205	1.494	3.1024
H	2.2877	-0.1258	4.3966	H	0.0717	2.6828	-3.2966
H	1.5238	0.7448	3.0534	H	0.2779	0.9445	-3.0105
H	3.2827	0.7658	3.2376	H	1.6107	1.908	-3.6684
H	3.6066	-2.2775	3.9461	H	2.9278	3.5118	-2.0511
H	4.6341	-1.4532	2.7642	H	2.2572	3.8181	-0.4373
H	3.7406	-2.9058	2.2921	H	1.389	4.3779	-1.8816
H	-4.2793	-2.8307	1.7962	H	-0.9981	3.6229	0.1734
H	-3.2052	-4.076	1.1383	H	-1.5601	4.1363	1.7606
H	-4.4235	-3.3279	0.1066	H	-2.6694	4.157	0.3872
H	-0.7124	0.7764	2.6158	H	-1.8814	-1.9269	2.6054
H	-2.3516	0.8831	3.2846	H	-0.3663	-1.3804	1.8628
H	-1.3656	2.3379	3.1508	H	-0.9187	-3.0453	1.6343
H	-4.3549	2.9683	0.2024	H	-1.4171	-1.1255	-2.2189
H	-3.8656	3.4017	1.8483	H	-0.4917	-2.4099	-1.4258
H	-4.7091	1.8812	1.5498	H	-0.1366	-0.7237	-1.0583
H	0.723	2.2074	1.0077	H	-3.7633	-4.8879	-0.5383
H	1.0159	2.8384	-0.6169	H	-2.3804	-4.4859	0.4842
H	0.3547	1.2132	-0.3994	H	-2.3143	-4.1766	-1.2576
H	-0.8819	3.541	-2.1103	H	-3.9698	-3.1083	1.9752
H	-2.5657	3.3329	-1.6104	H	-5.3261	-3.4948	0.9093
H	-1.58	1.9146	-1.9894	H	-4.9403	-1.8046	1.273
H	-0.3682	4.8788	-0.0037	H	-3.7875	-2.3029	-2.2961
H	-0.7576	4.2337	1.5939	H	-4.9067	-1.4078	-1.2547
H	-2.0575	4.7218	0.4946	H	-5.1792	-3.1251	-1.5798

Coordinates of **11-c3** (1.39 kJ/mol).

Atom	Coordinates x / A	y / A	z / A
C	1.9285	0.3114	-0.8103
C	1.9385	-1.0521	-0.4455
C	3.1358	-1.597	0.0196
C	4.3035	-0.8349	0.1318
C	4.2576	0.5084	-0.2394
C	3.084	1.1049	-0.7118
C	0.6903	-1.9179	-0.5736
H	3.1599	-2.6418	0.309
C	5.5905	-1.4529	0.6504
H	5.1609	1.105	-0.1546
C	3.078	2.5815	-1.0868
C	0.6441	0.9147	-1.2896
O	0.1922	1.8359	-0.4148
C	-1.1333	2.3718	-0.6233
C	-1.842	2.4991	0.7201
C	-1.9598	1.1494	1.4724
C	-0.6926	0.673	2.2177
C	-0.0993	1.765	3.1172
C	-1.0085	-0.5764	3.0466
O	0.0607	0.6083	-2.3121
C	6.0794	-2.5888	-0.2632
C	5.4347	-1.9385	2.1012
C	3.039	3.4558	0.1784
C	4.2593	2.9634	-1.992
C	0.5649	-2.9647	0.5418
C	0.6554	-2.5911	-1.9574
C	-3.2206	3.1303	0.4803
H	-1.2695	3.1863	1.3498
O	-2.3658	0.1422	0.5425
Si	-3.784	-0.7501	0.468
C	-5.1879	0.1354	1.3611
C	-3.5523	-2.437	1.2773
C	-4.1585	-0.9797	-1.3862
C	-4.5186	0.366	-2.0414
C	-2.9217	-1.5668	-2.0928
C	-5.3483	-1.946	-1.5518
H	-0.1857	-1.2665	-0.4966
H	6.3544	-0.6655	0.6421
H	2.1643	2.7856	-1.6536
H	-1.0283	3.3509	-1.1008
H	-1.6756	1.7063	-1.2925
H	-2.7409	1.2845	2.237
H	0.055	0.4049	1.468

H	0.3279	2.5896	2.5416
H	0.7026	1.348	3.7342
H	-0.8559	2.1786	3.7959
H	-1.7862	-0.3677	3.7927
H	-0.1174	-0.9172	3.5835
H	-1.3528	-1.3949	2.4156
H	6.2149	-2.2375	-1.2904
H	5.3604	-3.4146	-0.2837
H	7.0351	-2.9868	0.0935
H	5.1118	-1.124	2.7565
H	6.3838	-2.3306	2.4812
H	4.6905	-2.739	2.1702
H	3.9328	3.2921	0.79
H	2.1614	3.2152	0.7834
H	2.9993	4.5179	-0.0861
H	4.1629	4.0047	-2.3154
H	4.2961	2.3296	-2.8829
H	5.2174	2.8699	-1.4713
H	1.3236	-3.7493	0.4554
H	-0.4118	-3.4544	0.4801
H	0.6559	-2.5107	1.5321
H	-0.2481	-3.1999	-2.0661
H	1.5253	-3.245	-2.0857
H	0.6631	-1.8402	-2.7497
H	-3.1281	4.1038	-0.0116
H	-3.7459	3.2815	1.4276
H	-3.8434	2.4949	-0.154
H	-6.0991	-0.47	1.3038
H	-5.4097	1.1173	0.936
H	-4.9571	0.2732	2.4227
H	-2.691	-2.9704	0.8637
H	-4.4389	-3.0605	1.122
H	-3.4011	-2.3417	2.3563
H	-4.7453	0.2221	-3.1063
H	-3.6926	1.0798	-1.9783
H	-5.399	0.825	-1.578
H	-3.1388	-1.7456	-3.1548
H	-2.6232	-2.5263	-1.656
H	-2.0652	-0.89	-2.0379
H	-6.2498	-1.579	-1.0478
H	-5.1245	-2.9421	-1.1569
H	-5.594	-2.0634	-2.6155

3-3. Calculation of Boltzmann factors

To estimate the populations of the respective conformers, Boltzmann factors $f_{\mathrm{B}} = \exp\left(-\frac{E_{\mathrm{rel}}}{RT}\right)$ were calculated using the relative energy difference E_{rel} between the respective conformer and the lowest energy conformer, the gas constant $R = 8.3145\ \frac{\mathrm{J}}{\mathrm{mol\ K}}$ and the reaction temperature $T = 195.15\ K$.[22] Constants and conversion factors $\left(1\ \mathrm{H} = 2625.4988\ \frac{\mathrm{kJ}}{\mathrm{mol}}\right)$[22] where taken from reference 22. Conformers with Boltzmann factors less than 0.01 were not included due to the negligible population. Therefore n conformers of diketide j were taken into consideration. The population p_i of a specific conformer i was then calculated using the Boltzmann distribution.

$$p_i = \frac{\exp\left(-\frac{E_{\mathrm{rel,i}}}{RT}\right)}{\sum_{k=1}^{n} \exp\left(-\frac{E_{\mathrm{rel,k}}}{RT}\right)}$$

with the sum of all Boltzmann factors of a specific diketide.[23] Relative energies, Boltzmann factors, populations and if applicable conformers with similar structures are given in the table below. Structures of all considered conformers can be found in the Appendix 6.

Boltzmann factor and population of different conformers of carbamate **10**.

Conformer	E_{rel} in kJ/mol	Boltzmann factor	Population in %	Structurally similar to
**10-c1**	0.000	1.0000	39.3%	**10-c1**
**10-c2**	1.594	0.3744	14.7%	**10-c2**
**10-c3**	2.499	0.2144	8.4%	**10-c3**
**10-c4**	2.725	0.1864	7.3%	**10-c3**
**10-c5**	2.883	0.1692	6.6%	**10-c1**
**10-c6**	3.227	0.1369	5.4%	**10-c3**
**10-c7**	3.825	0.0946	3.7%	**10-c2**
**10-c8**	4.025	0.0837	3.3%	**10-c2**
**10-c9**	4.954	0.0472	1.9%	**10-c3**
**10-c10**	5.028	0.0451	1.8%	**10-c2**
**10-c11**	5.122	0.0426	1.7%	**10-c2**
**10-c12**	5.175	0.0412	1.6%	**10-c2**
**10-c13**	5.529	0.0331	1.3%	**10-c1**
**10-c14**	5.808	0.0279	1.1%	**10-c2**
**10-c15**	6.658	0.0165	0.6%	**10-c1**
**10-c16**	7.036	0.0131	0.5%	**10-c2**
**10-c17**	7.317	0.0110	0.4%	**10-c1**
**10-c18**	7.401	0.0104	0.4%	**10-c2**

Boltzmann factor and population of different conformers of carbamate **12**.

Conformer	E_{rel} in kJ/mol	Boltzmann factor	Population in %	Structurally similar to
**12-c1**	0.000	1.0000	60.7%	**12-c1**
**12-c2**	1.134	0.4971	30.2%	**12-c2**
**12-c3**	5.340	0.0372	2.3%	**12-c3**
**12-c4**	5.519	0.0333	2.0%	**12-c4**
**12-c5**	6.031	0.0243	1.5%	**12-c5**
**12-c6**	6.419	0.0191	1.2%	**12-c3**
**12-c7**	7.162	0.0121	0.7%	**12-c3**
**12-c8**	7.239	0.0115	0.7%	**12-c8**
**12-c9**	7.244	0.0115	0.7%	**12-c3**

Boltzmann factor and population of different conformers of TIB ester **9**.

Conformer	E_{rel} in kJ/mol	Boltzmann factor	Population in %	Structurally similar to
**9-c1**	0.000	1.0000	87.4%	**9-c1**
**9-c2**	4.823	0.0512	4.5%	**9-c2**
**9-c3**	5.004	0.0458	4.0%	**9-c3**
**9-c4**	5.884	0.0266	2.3%	**9-c1**
**9-c5**	6.257	0.0212	1.8%	**9-c1**

Boltzmann factor and population of different conformers of TIB ester **11**.

Conformer	E_{rel} in kJ/mol	Boltzmann factor	Population in %	Structurally similar to
**11-c1**	0.000	1.0000	45.4%	**11-c1**
**11-c2**	1.181	0.4829	21.9%	**11-c2**
**11-c3**	1.391	0.4243	19.2%	**11-c3**
**11-c4**	3.765	0.0982	4.5%	**11-c3**
**11-c5**	4.403	0.0663	3.0%	**11-c3**
**11-c6**	4.442	0.0647	2.9%	**11-c3**
**11-c7**	5.886	0.0266	1.2%	**11-c2**
**11-c8**	6.267	0.0210	1.0%	**11-c2**
**11-c9**	7.386	0.0105	0.5%	**11-c2**
**11-c10**	7.433	0.0102	0.5%	**11-c2**

4. References

[1] Coombs, J. R.; Zhang, L.; Morken, J. P. Synthesis of Vinyl Boronates from Aldehydes by a Practical Boron–Wittig Reaction. *Org. Lett.* **2015**, *17*, 1708–1711.

[2] Hesse, M. J.; Butts, C. P.; Willis, C. L.; Aggarwal, V. K. Diastereodivergent Synthesis of Trisubstituted Alkenes through Protodeboronation of Allylic Boronic Esters: Application to the Synthesis of the Californian Red Scale Beetle Pheromone. *Angew. Chem. Int. Ed.* **2012**, *51*, 12444–12448.

[3] a) Evans, D. A.; Connell, B. T. Synthesis of the Antifungal Macrolide Antibiotic (+)-Roxaticin. *J. Am. Chem. Soc.* **2003**, *125*, 10899–10905; b) Zampella, A.; Sepe, V.; D'Orsi, R.; Bifulco, G.; Bassarello, C.; D'Auria, M. V. Stereochemical assignment of the C23–C35 portion of sphinxolide/reidispongiolide class of natural products by asymmetric synthesis. *Tetrahedron: Asymmetry* **2003**, *14*, 1787–1798.

[4] Linne, Y.; Bonandi, E.; Tabet, C.; Geldsetzer, J.; Kalesse, M. The Total Synthesis of Chondrochloren A. *Angew. Chem. Int. Ed.* **2021**, *60*, 6938–6942.

[5] Crimmins, M. T.; King, B. W.; Tabet, E. A.; Chaudhary, K. Asymmetric Aldol Additions: Use of Titanium Tetrachloride and (−)-Sparteine for the Soft Enolization of *N*-Acyl Oxazolidinones, Oxazolidinethiones, and Thiazolidinethiones. *J. Org. Chem.* **2001**, *66*, 894–902.

[6] Echeverria, P.-G.; Prévost, S.; Cornil, J.; Férard, C.; Reymond, S.; Guérinot, A.; Cossy, J.; Ratovelomanana-Vidal, V.; Phansavath, P. Synthetic Strategy toward the C44–C65 Fragment of Mirabalin. *Org. Lett.* **2014**, *16*, 2390–2393.

[7] Hoppe, D.; Hintze, F.; Tebben, P.; Paetow, M.; Ahrens, H.; Schwerdtfeger, J.; Sommerfeld, P.; Haller, J.; Guarnieri, W.; Kolczewski, S.; Hense, T.; Hoppe, I. Enantioselective synthesis via sparteine-induced asymmetric deprotonation. *Pure & Appl. Chem.* **1994**, *66*, 1479–1486.

[8] Würthwein, E.-U.; Hoppe, D. Enantioselective Lithiation of O-Alkyl and O-Alk-2-enyl Carbamates in the Presence of (−)-Sparteine and (−)-α-Isosparteine. A Theoretical Study. *J. Org. Chem.* ***2005***, *70*, 4443–4451.

[9] Grimme, S. Exploration of Chemical Compound, Conformer, and Reaction Space with Meta-Dynamics Simulations Based on Tight-Binding Quantum Chemical Calculations. *J. Chem. Theory Comput.* **2019**, *15*, 2847–2862.

[10] Pracht, P.; Bohle, F.; Grimme, S. Automated Exploration of the Low-Energy Chemical Space with Fast Quantum Chemical Methods. *Phys. Chem. Chem. Phys.* **2020**, *22*, 7169–7192.

[11] Becke, A. D. Density-functional Thermochemistry. III. The Role of Exact Exchange. *The Journal of Chemical Physics* **1993**, *98*, 5648–5652.

[12] Hehre, W. J.; Ditchfield, R.; Pople, J. A. Self—Consistent Molecular Orbital Methods. XII. Further Extensions of Gaussian—Type Basis Sets for Use in Molecular Orbital Studies of Organic Molecules. *The Journal of Chemical Physics* **1972**, *56*, 2257–2261.

[13] Hariharan, P. C.; Pople, J. A. The Influence of Polarization Functions on Molecular Orbital Hydrogenation Energies. *Theoret. Chim. Acta* **1973**, *28*, 213–222.

[14] Francl, M. M.; Pietro, W. J.; Hehre, W. J.; Binkley, J. S.; Gordon, M. S.; DeFrees, D. J.; Pople, J. A. Self-consistent Molecular Orbital Methods. XXIII. A Polarization-type Basis Set for Second-row Elements. *The Journal of Chemical Physics* **1982**, *77*, 3654–3665.

[15] Hariharan, P. C.; Pople, J. A. Accuracy of AH_n Equilibrium Geometries by Single Determinant Molecular Orbital Theory. *Molecular Physics* **1974**, *27*, 209–214.

[16] Hariharan, P. C.; Pople, J. A. The Influence of Polarization Functions on Molecular Orbital Hydrogenation Energies. *Theoret. Chim. Acta* **1973**, *28*, 213–222.

[17] Frisch, M. J.; Trucks, G. W.; Schlegel, H. B.; Scuseria, G. E.; Robb, M. A.; Cheeseman, J. R.; Scalmani, G.; Barone, V.; Petersson, G. A.; Nakatsuji, H.; Li, X.; Caricato, M.; Marenich, A. V.; Bloino, J.; Janesko, B. G.; Gomperts, R.; Mennucci, B.; Hratchian, H. P.; Ortiz, J. V.; Izmaylov, A. F.; Sonnenberg, J. L.; Williams-Young, D.; Ding, F.; Lipparini, F.; Egidi, F.; Goings, J.; Peng, B.; Petrone, A.; Henderson, T.; Ranasinghe, D.; Zakrzewski, V. G.; Gao, J.; Rega, N.; Zheng, G.; Liang, W.; Hada, M.; Ehara, M.; Toyota, K.; Fukuda, R.; Hasegawa, J.; Ishida, M.; Nakajima, T.; Honda, Y.; Kitao, O.; Nakai, H.; Vreven, T.; Throssell, K.; Montgomery, J. A., Jr.; Peralta, J. E.; Ogliaro, F.; Bearpark, M. J.; Heyd, J. J.; Brothers, E. N.; Kudin, K. N.; Staroverov, V. N.; Keith, T. A.; Kobayashi, R.; Normand, J.; Raghavachari, K.; Rendell, A. P.; Burant, J. C.; Iyengar, S. S.; Tomasi, J.; Cossi, M.; Millam, J. M.; Klene, M.; Adamo, C.; Cammi, R.; Ochterski, J. W.; Martin, R. L.; Morokuma, K.; Farkas, O.; Foresman, J. B.; Fox, D. J. Gaussian 16 Revision B.01, 2016.

[18] Dennington, R.; Keith, T. A.; Millam, J. M. GaussView Version 6-0.16, 2019.

[19] Grimme, S.; Ehrlich, S.; Goerigk, L. Effect of the Damping Function in Dispersion Corrected Density Functional Theory. *J. Comput. Chem.* **2011**, *32*, 1456–1465.

[20] Grimme, S.; Antony, J.; Ehrlich, S.; Krieg, H. A Consistent and Accurate Ab Initio Parametrization of Density Functional Dispersion Correction (DFT-D) for the 94 Elements H-Pu. *The Journal of Chemical Physics* **2010**, *132*, 154104-1–154104-18.

[21] Tomasi, J.; Mennucci, B.; Cammi, R. Quantum Mechanical Continuum Solvation Models. *Chem. Rev.* **2005**, *105*, 2999–3094.

[22] Atkins, P. W.; Friedman, R. Molecular Quantum Mechanics, 5th ed.; Oxford University Press: Oxford; New York, 2011.

[23] Atkins, P. W.; de Paula, J.; Keeler, J. J.; Hartmann, C. *Physikalische Chemie*; Wiley-VCH; Weinheim, Germany, 2022.

5. Spectra

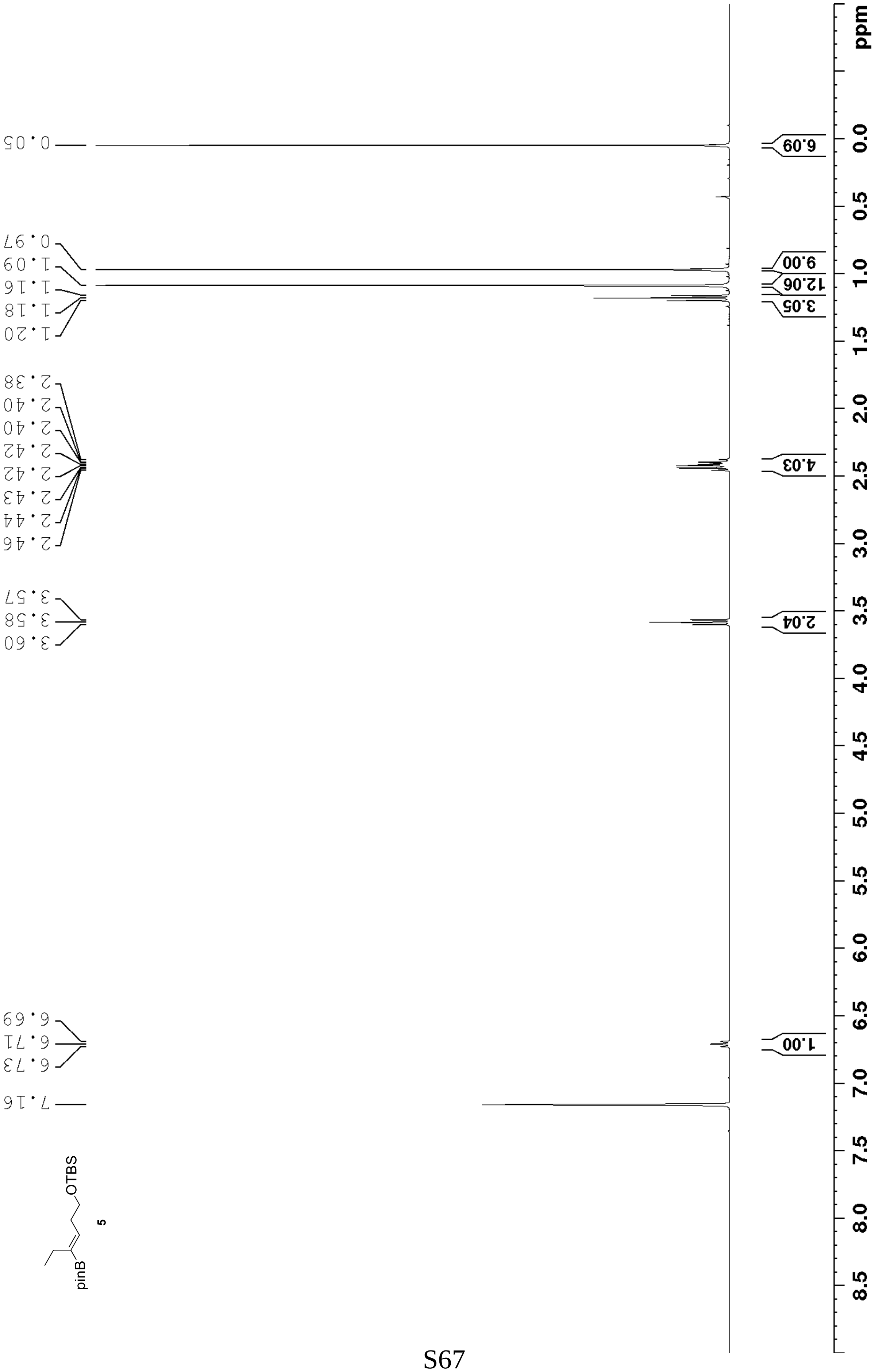
0.05
0.97
1.09
1.16
1.18
1.20
2.38
2.40
2.40
2.42
2.42
2.43
2.44
2.46
3.57
3.58
3.60
6.69
6.71
6.73
7.16
6.09
9.00
12.06
3.05
4.03
2.04
1.00
ppm
pinB
OTBS
5

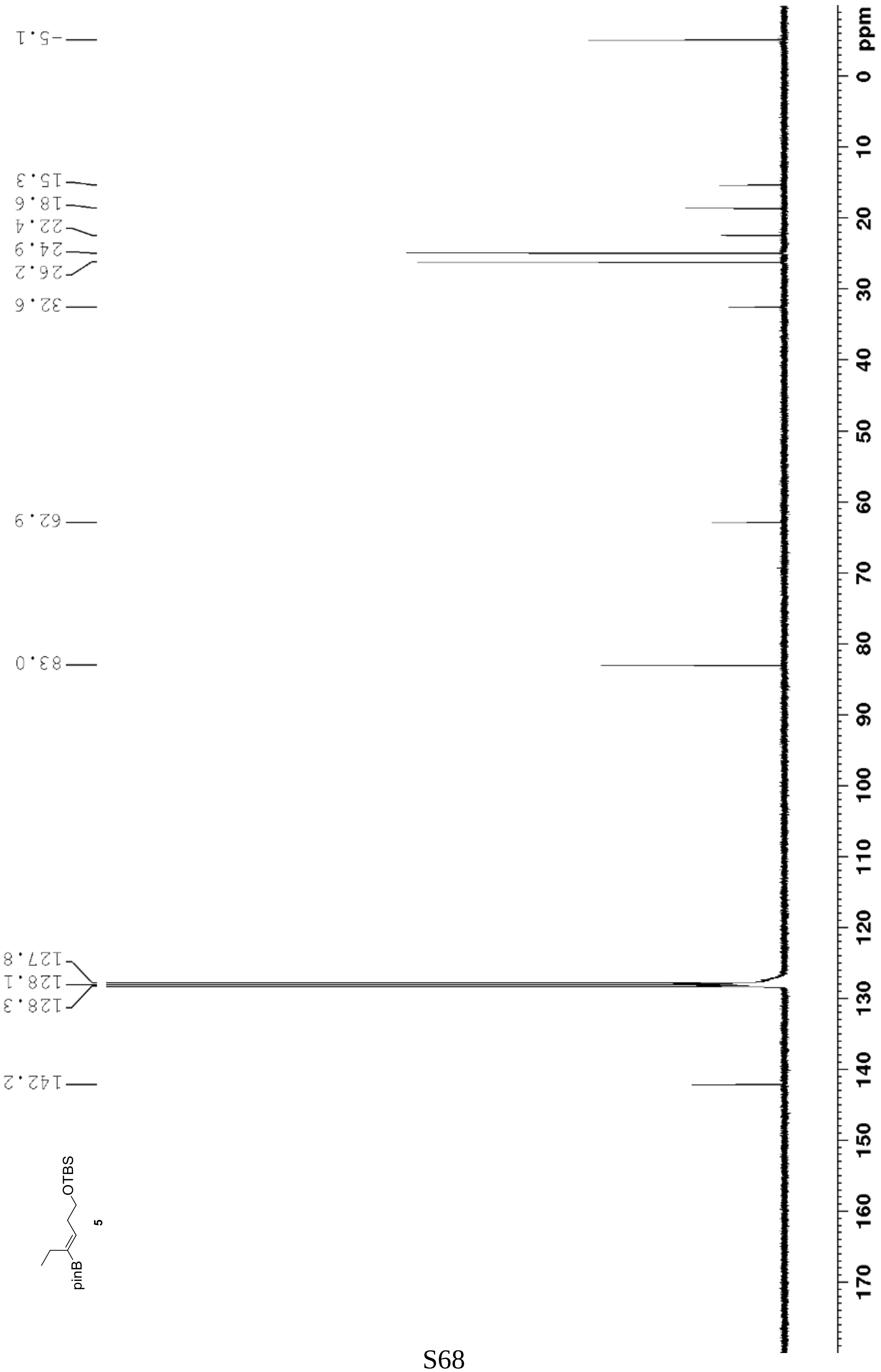

-5.1
15.3
18.6
22.4
24.9
26.2
32.6
62.9
83.0
127.8
128.1
128.3
142.2
pinB
OTBS
5
ppm
0
10
20
30
40
50
60
70
80
90
100
110
120
130
140
150
160
170

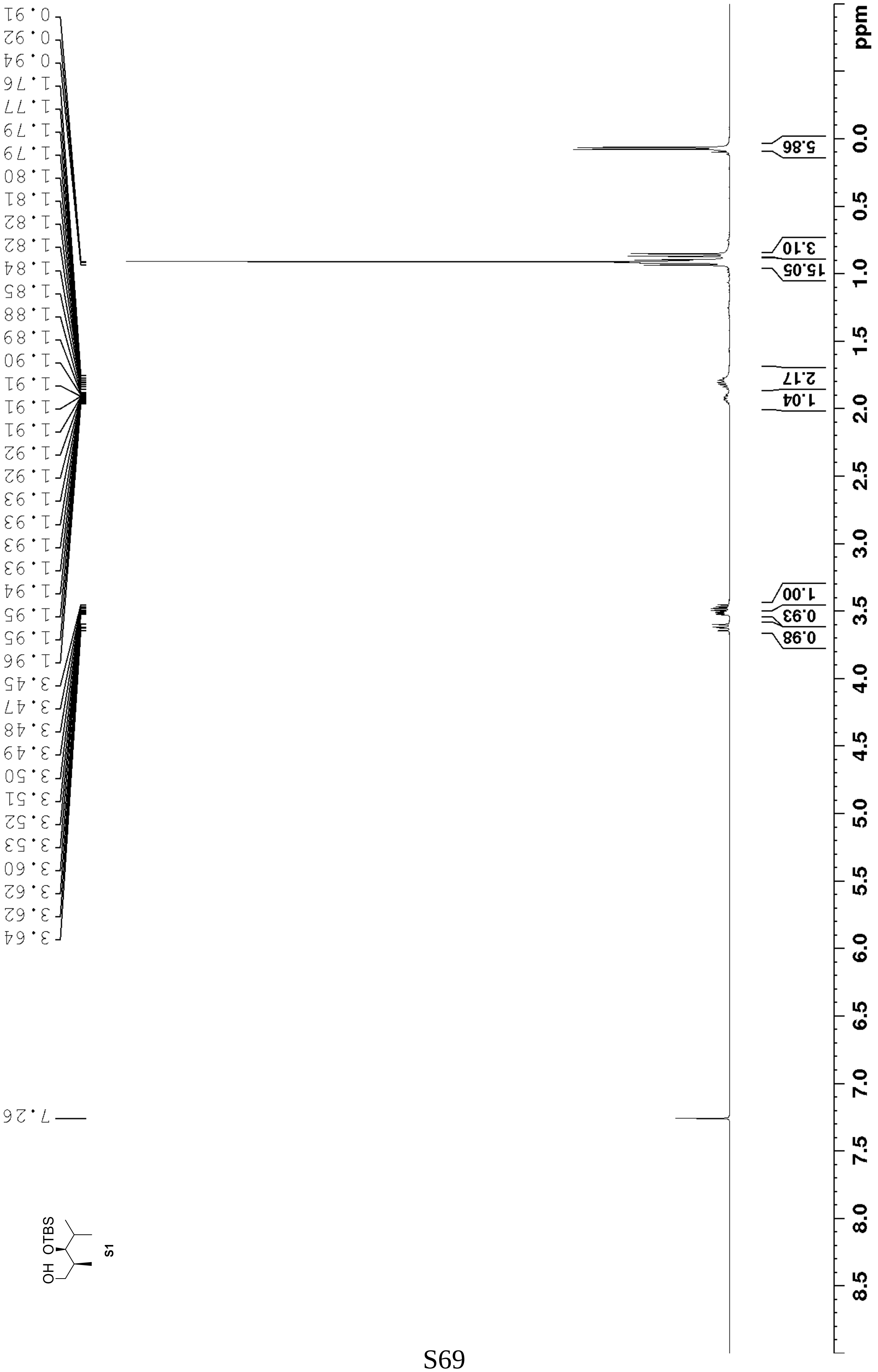

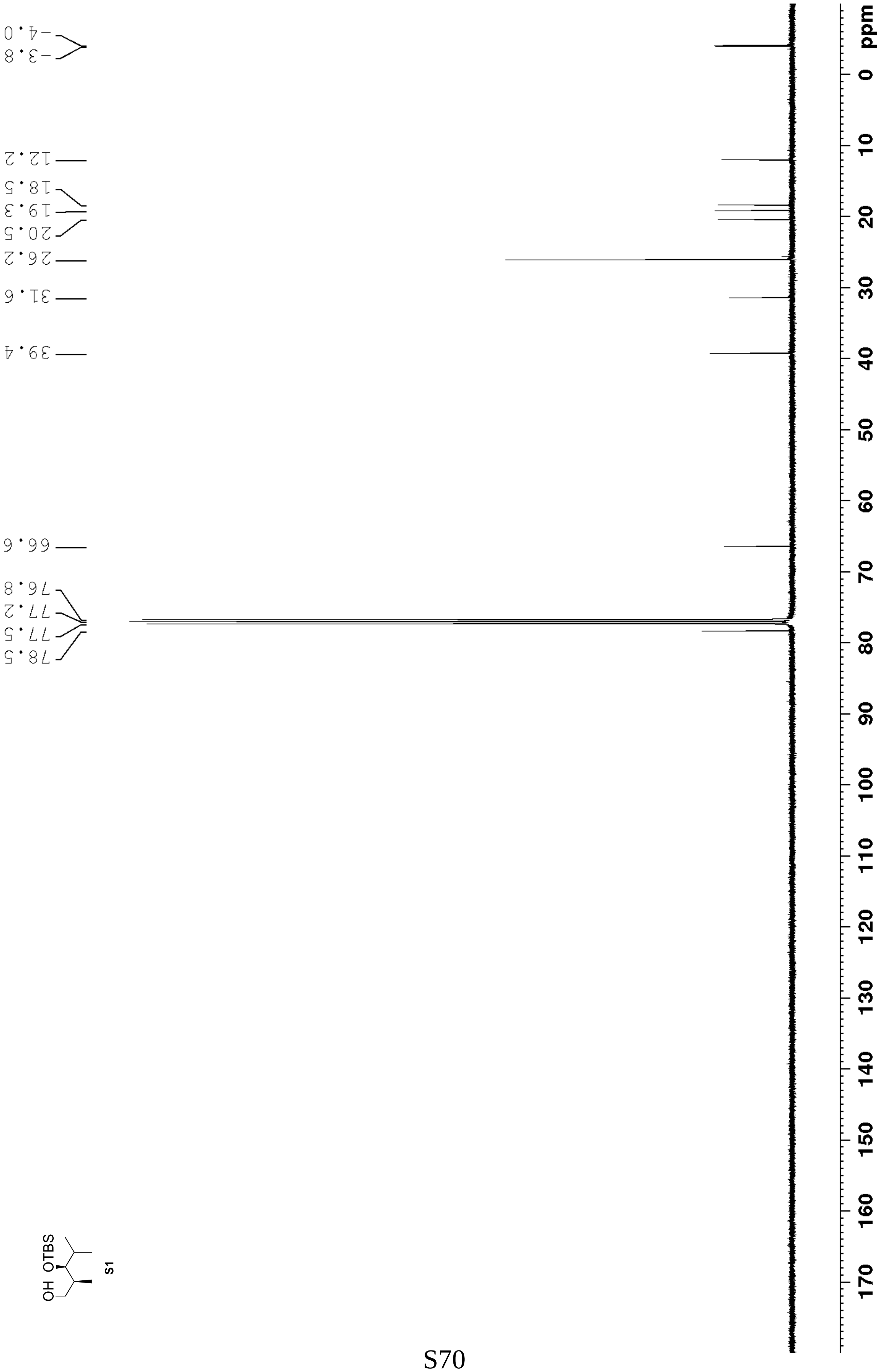
-4.0
-3.8
12.2
18.5
19.3
20.5
26.2
31.6
39.4
66.6
76.8
77.2
77.5
78.5
ppm
OH OTBS
S1

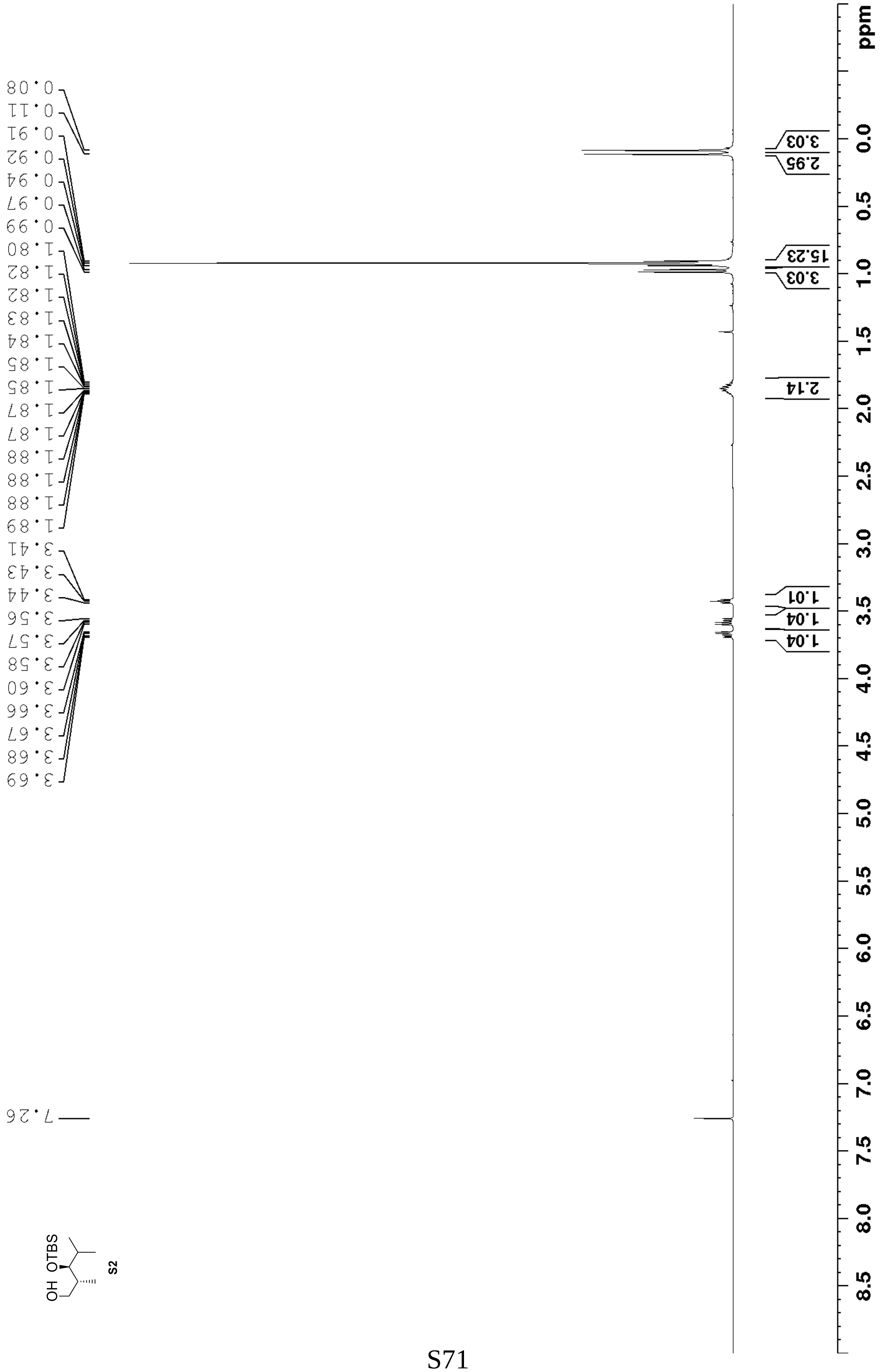
0.08
0.11
0.91
0.92
0.94
0.97
0.99
1.80
1.82
1.82
1.83
1.84
1.85
1.85
1.87
1.87
1.88
1.88
1.88
1.89
3.41
3.43
3.44
3.56
3.57
3.58
3.60
3.66
3.67
3.68
3.69
7.26
3.03
2.95
15.23
3.03
2.14
1.01
1.04
1.04
ppm
OH
OTBS
S2

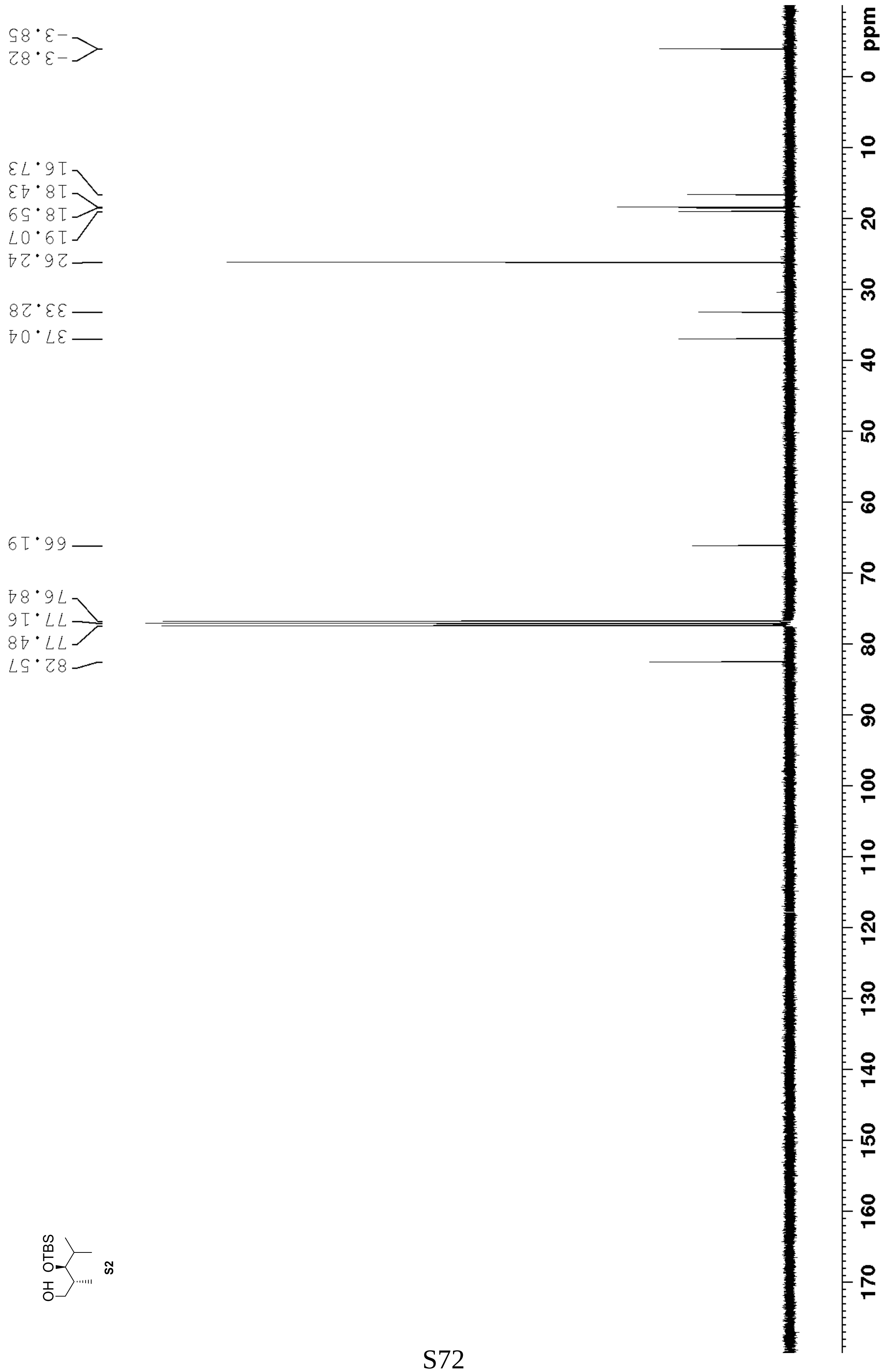
-3.85
-3.82
16.73
18.43
18.59
19.07
26.24
33.28
37.04
66.19
76.84
77.16
77.48
82.57
ppm
OH OTBS
S2

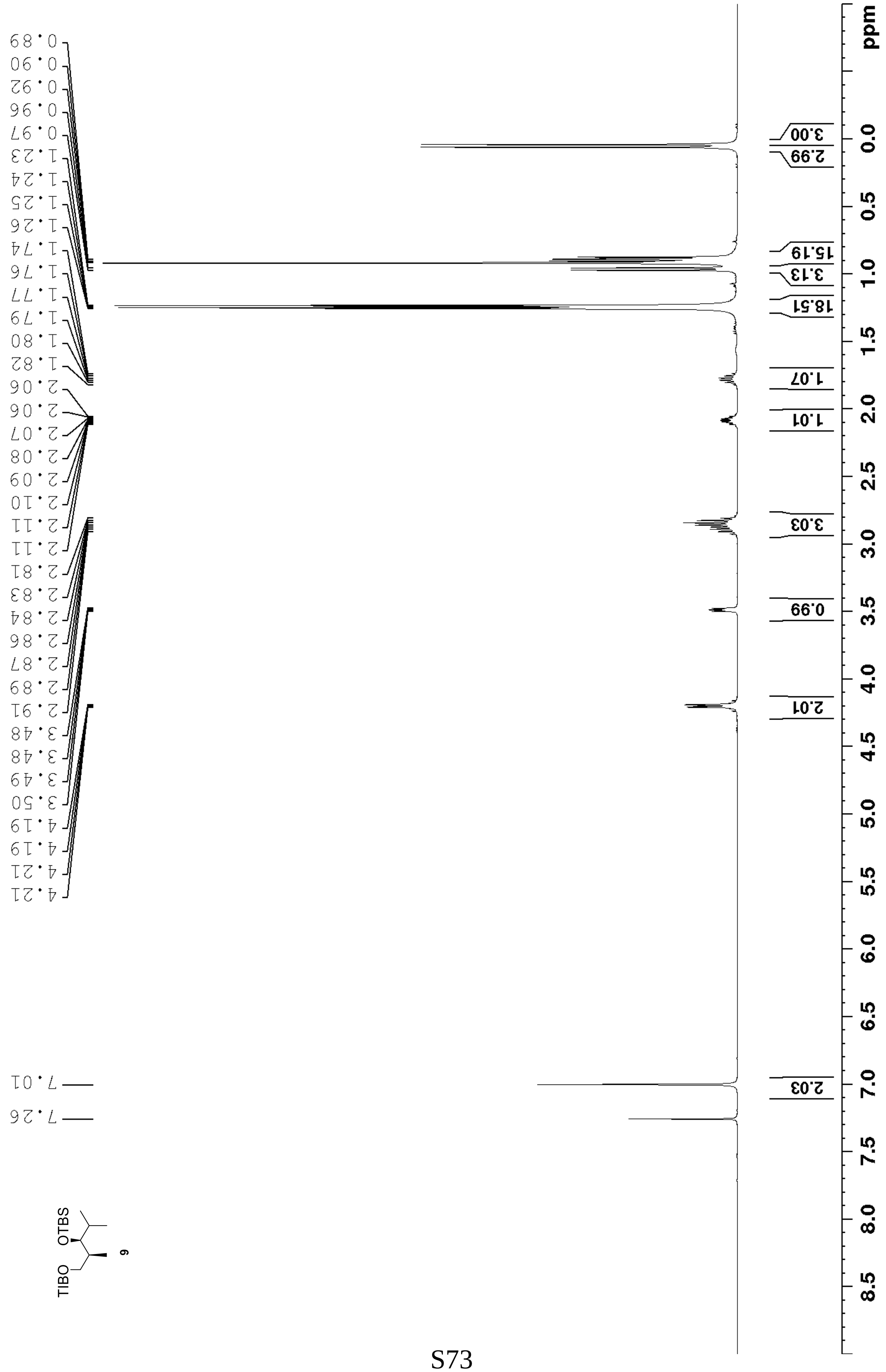

0.89
0.90
0.92
0.96
0.97
1.23
1.24
1.25
1.26
1.74
1.76
1.77
1.79
1.80
1.82
2.06
2.06
2.07
2.08
2.09
2.10
2.11
2.11
2.81
2.83
2.84
2.86
2.87
2.89
2.91
3.48
3.48
3.49
3.50
4.19
4.19
4.21
4.21
7.01
7.26
3.00
2.99
15.19
3.13
18.51
1.07
1.01
3.03
0.99
2.01
2.03
ppm
TIBO
OTBS
9

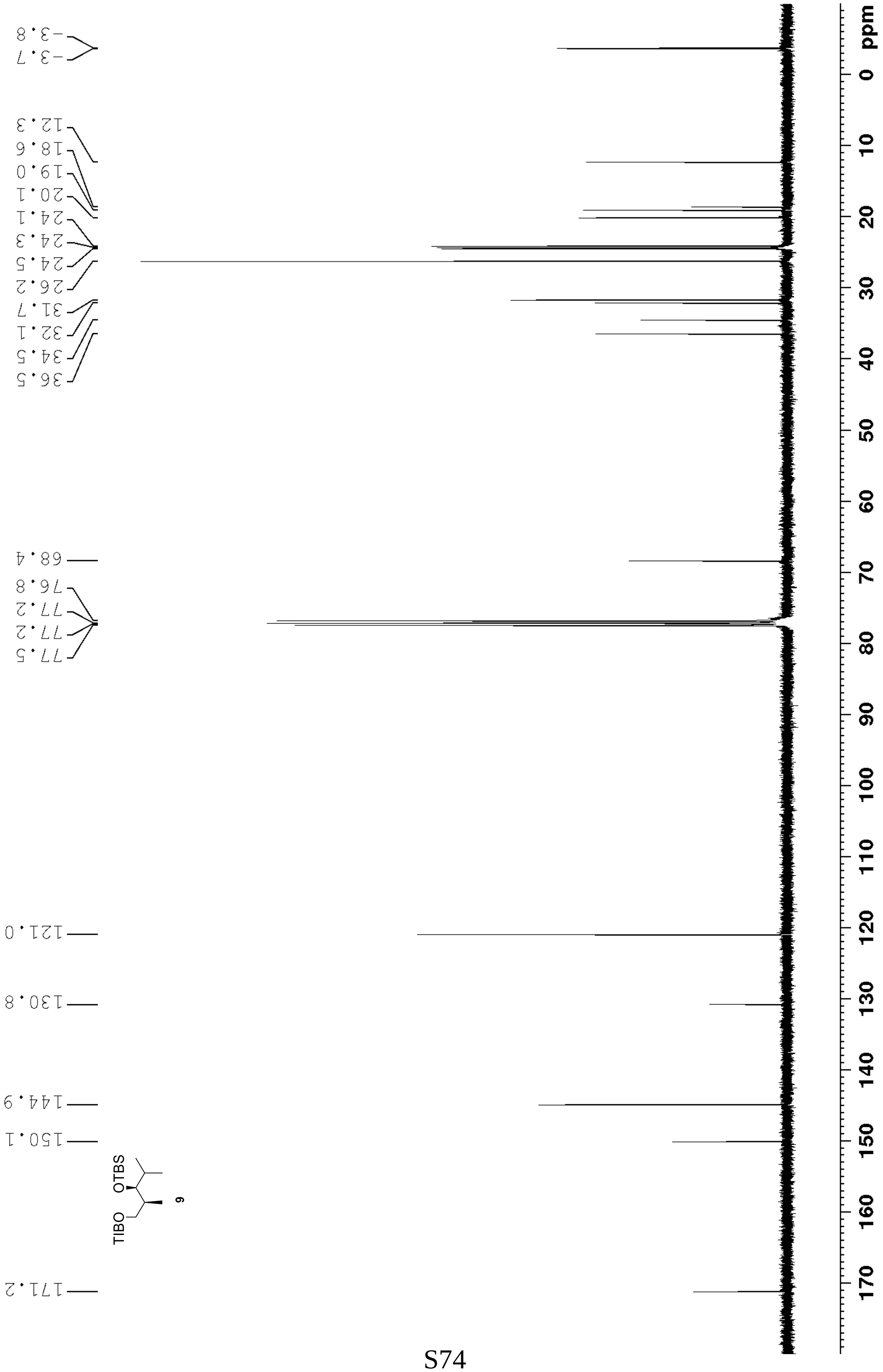
-3.8
-3.7
12.3
18.6
19.0
20.1
24.1
24.3
24.5
26.2
31.7
32.1
34.5
36.5
68.4
76.8
77.2
77.2
77.5
121.0
130.8
144.9
150.1
171.2
TIBO
OTBS
9
ppm

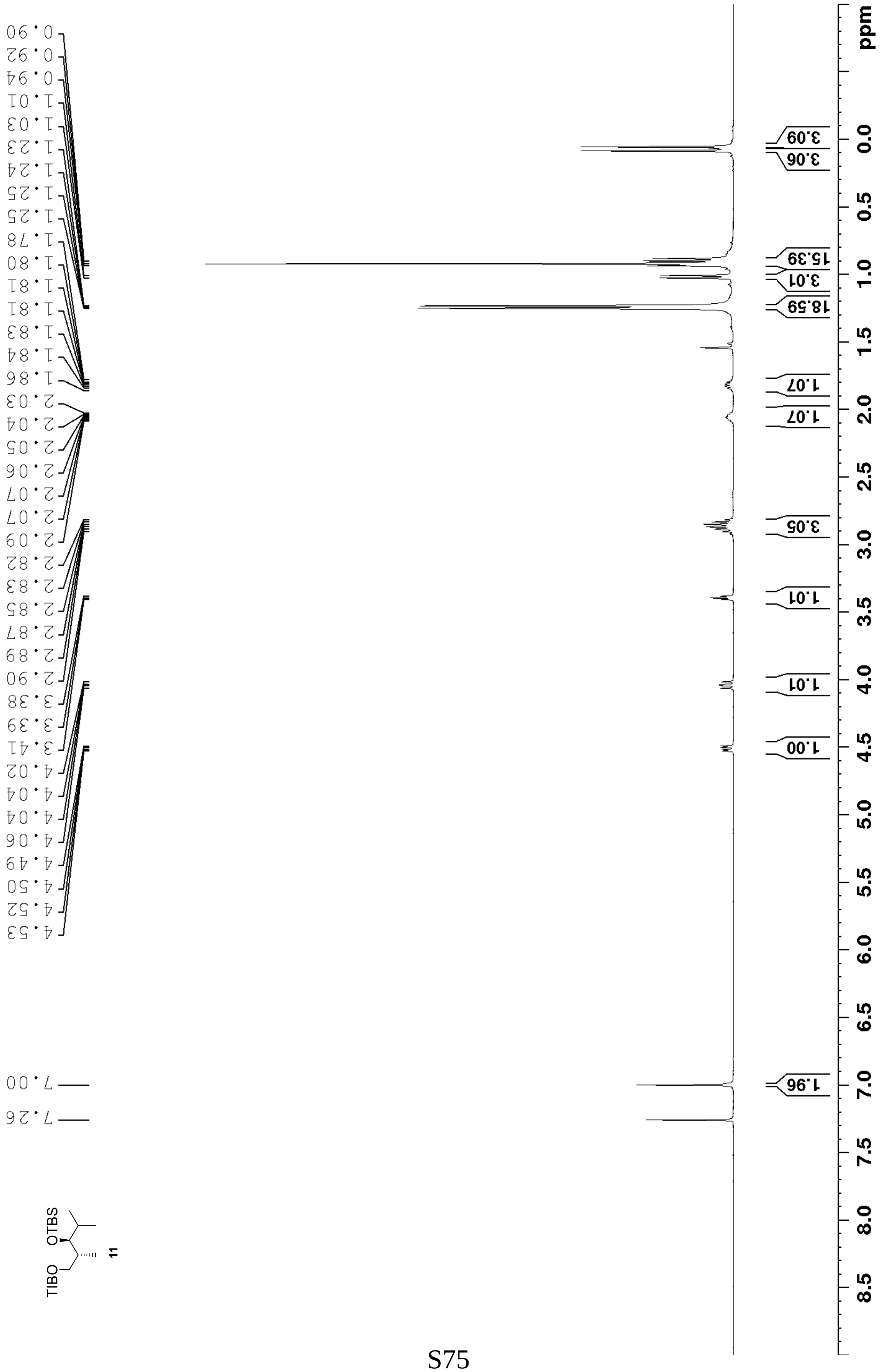
0.90
0.92
0.94
1.01
1.03
1.23
1.24
1.25
1.25
1.78
1.80
1.81
1.81
1.83
1.84
1.86
2.03
2.04
2.05
2.06
2.07
2.07
2.09
2.82
2.83
2.85
2.87
2.89
2.90
3.38
3.39
3.41
4.02
4.04
4.04
4.06
4.49
4.50
4.52
4.53
7.00
7.26
TIBO
OTBS
11
3.09
3.06
15.39
3.01
18.59
1.07
1.07
3.05
1.01
1.01
1.00
1.96
ppm

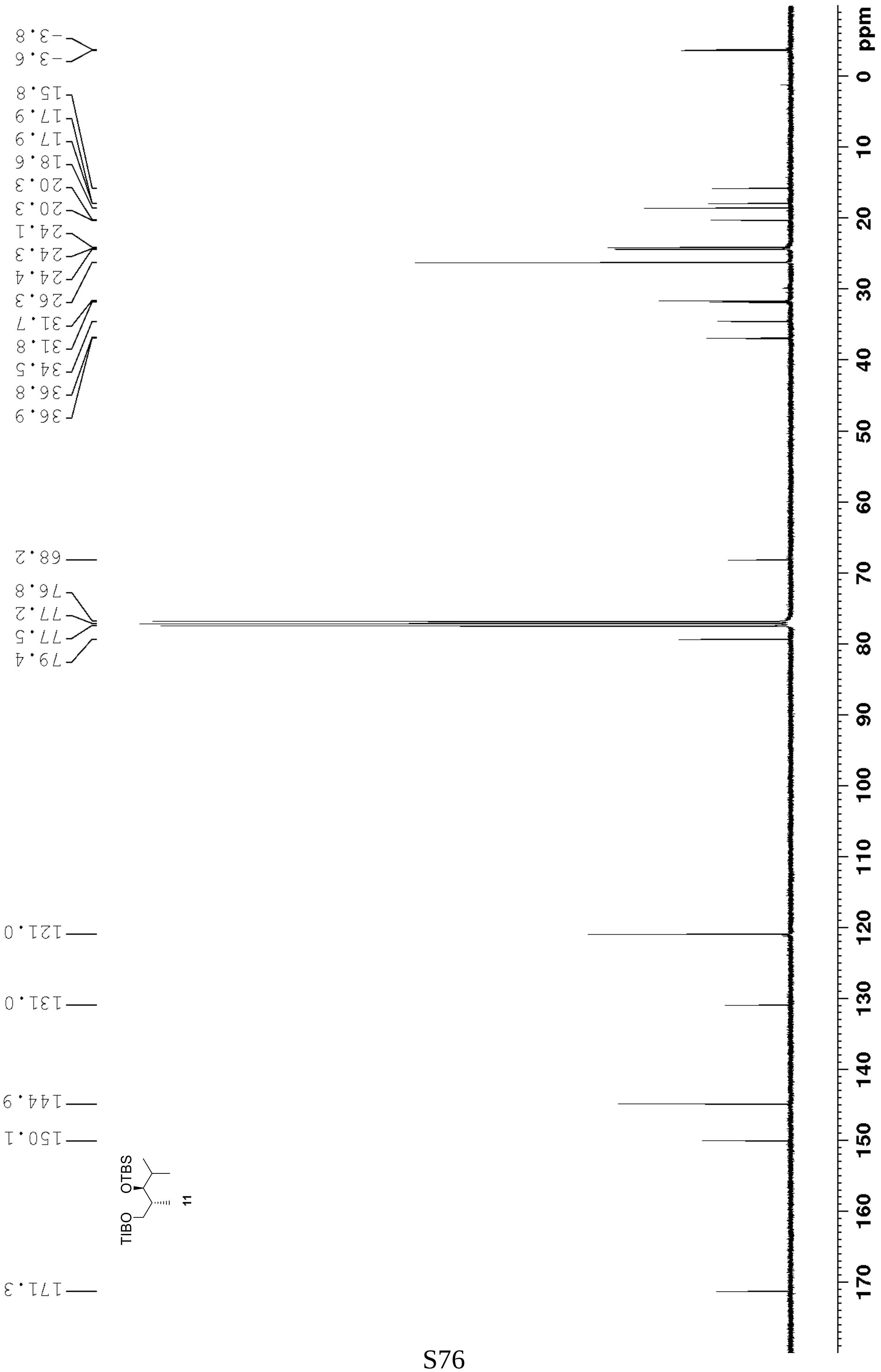
-3.8
-3.6
15.8
17.9
17.9
18.6
20.3
20.3
24.1
24.3
24.4
26.3
31.7
31.8
34.5
36.8
36.9
68.2
76.8
77.2
77.5
79.4
121.0
131.0
144.9
150.1
171.3
TIBO
OTBS
11
ppm

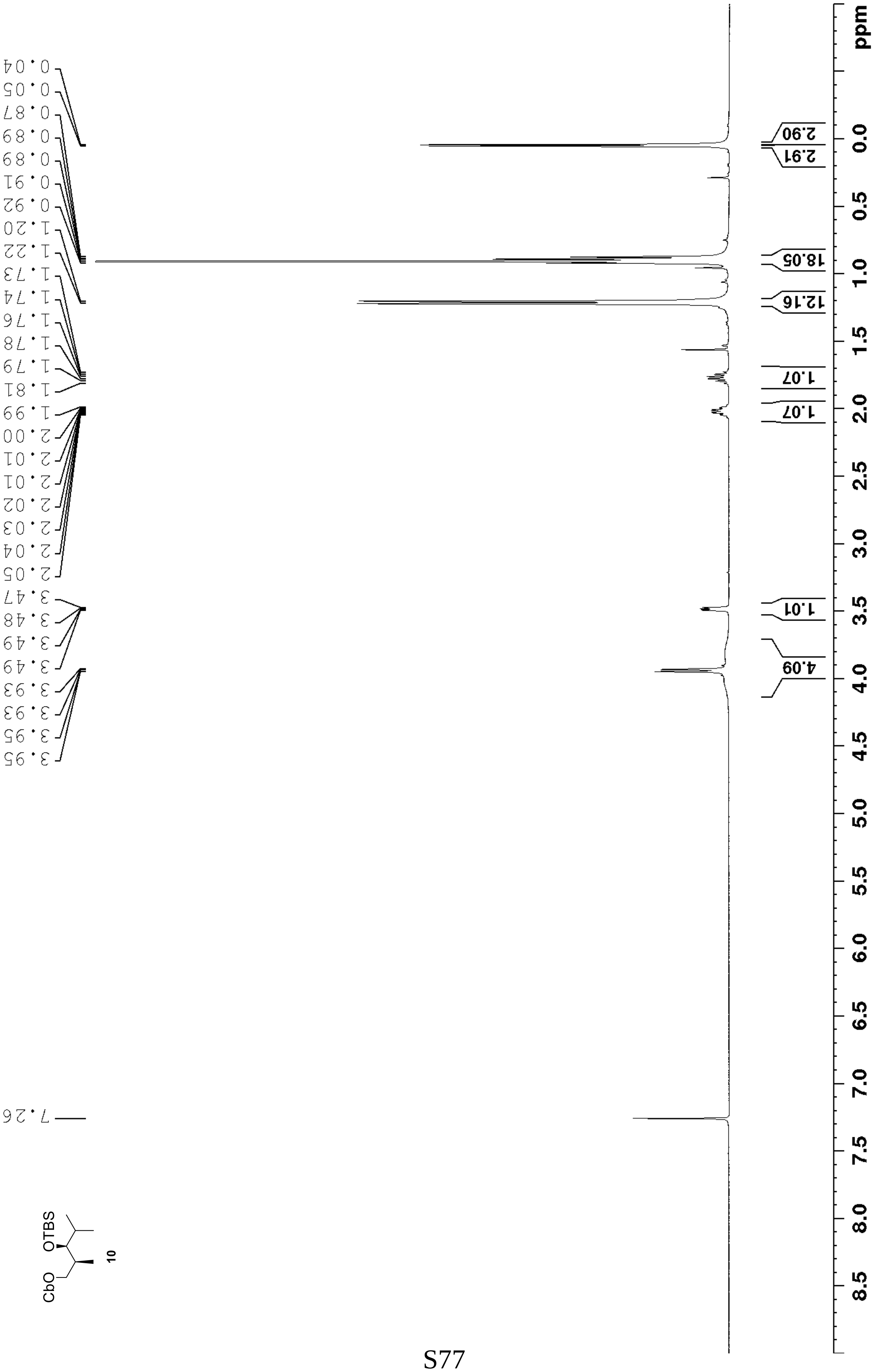
0.04
0.05
0.87
0.89
0.89
0.91
0.92
1.20
1.22
1.73
1.74
1.76
1.78
1.79
1.81
1.99
2.00
2.01
2.01
2.02
2.03
2.04
2.05
3.47
3.48
3.49
3.49
3.93
3.93
3.95
3.95
7.26
2.90
2.91
18.05
12.16
1.07
1.07
1.01
4.09
ppm
0.0
0.5
1.0
1.5
2.0
2.5
3.0
3.5
4.0
4.5
5.0
5.5
6.0
6.5
7.0
7.5
8.0
8.5
CbO
OTBS
10

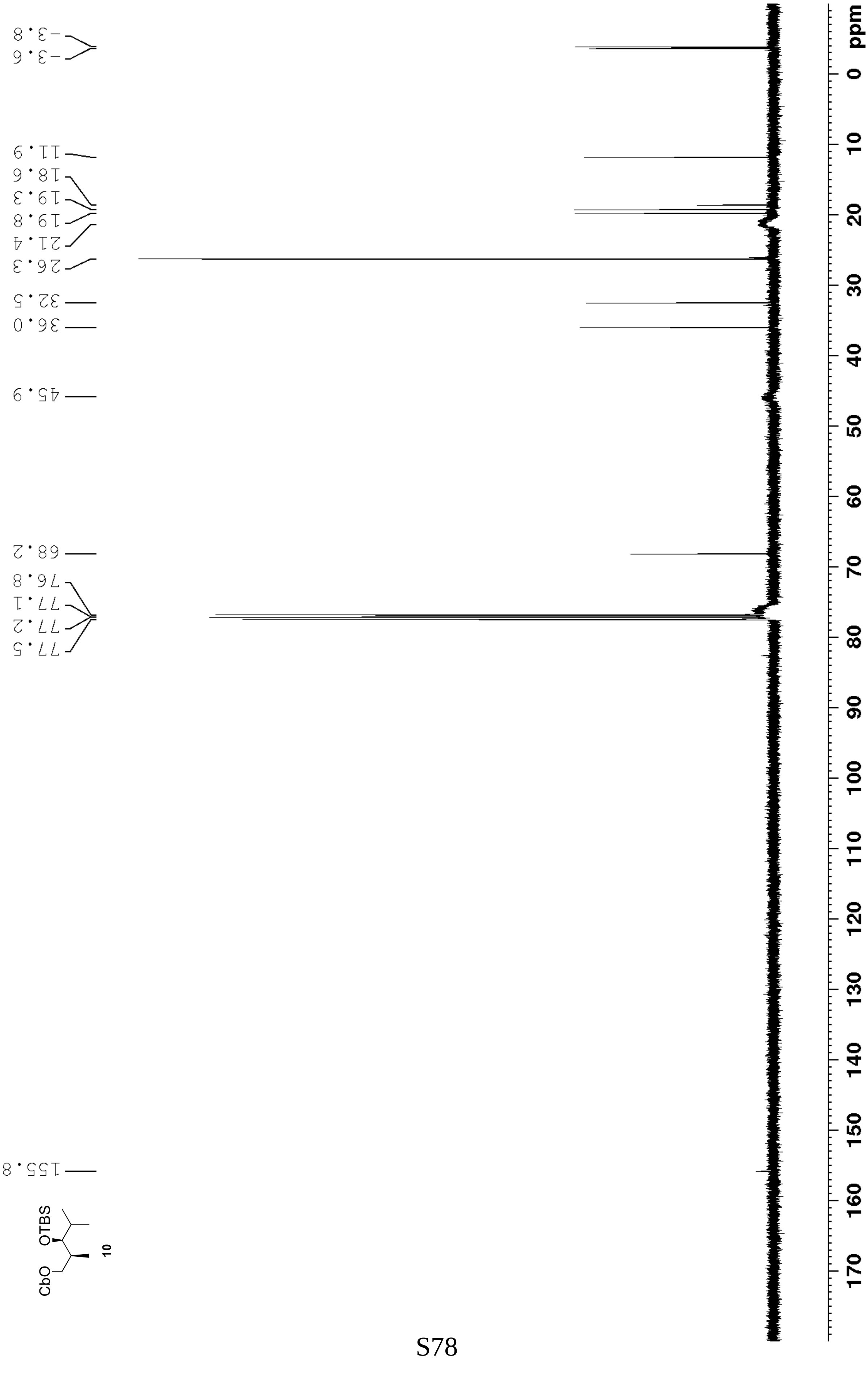

-3.8
-3.6
11.9
18.6
19.3
19.8
21.4
26.3
32.5
36.0
45.9
68.2
76.8
77.1
77.2
77.5
155.8
CbO
OTBS
10
ppm

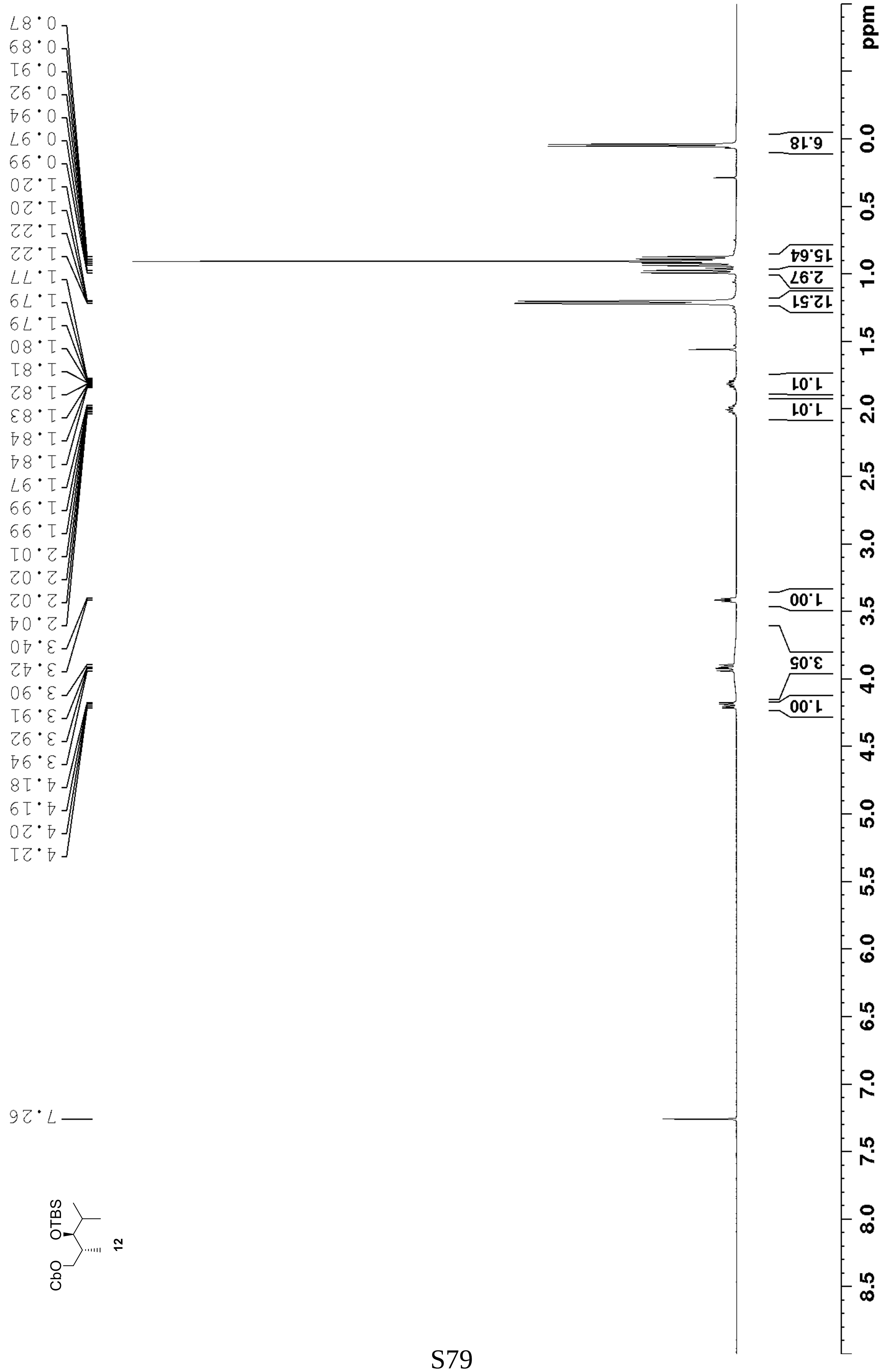
0.87
0.89
0.91
0.92
0.94
0.97
0.99
1.20
1.20
1.22
1.22
1.77
1.79
1.79
1.80
1.81
1.82
1.83
1.84
1.84
1.97
1.99
1.99
2.01
2.02
2.02
2.04
3.40
3.42
3.90
3.91
3.92
3.94
4.18
4.19
4.20
4.21
7.26
CbO
OTBS
12
6.18
15.64
2.97
12.51
1.01
1.01
1.00
3.05
1.00
ppm
0.0
0.5
1.0
1.5
2.0
2.5
3.0
3.5
4.0
4.5
5.0
5.5
6.0
6.5
7.0
7.5
8.0
8.5

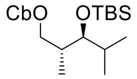

12

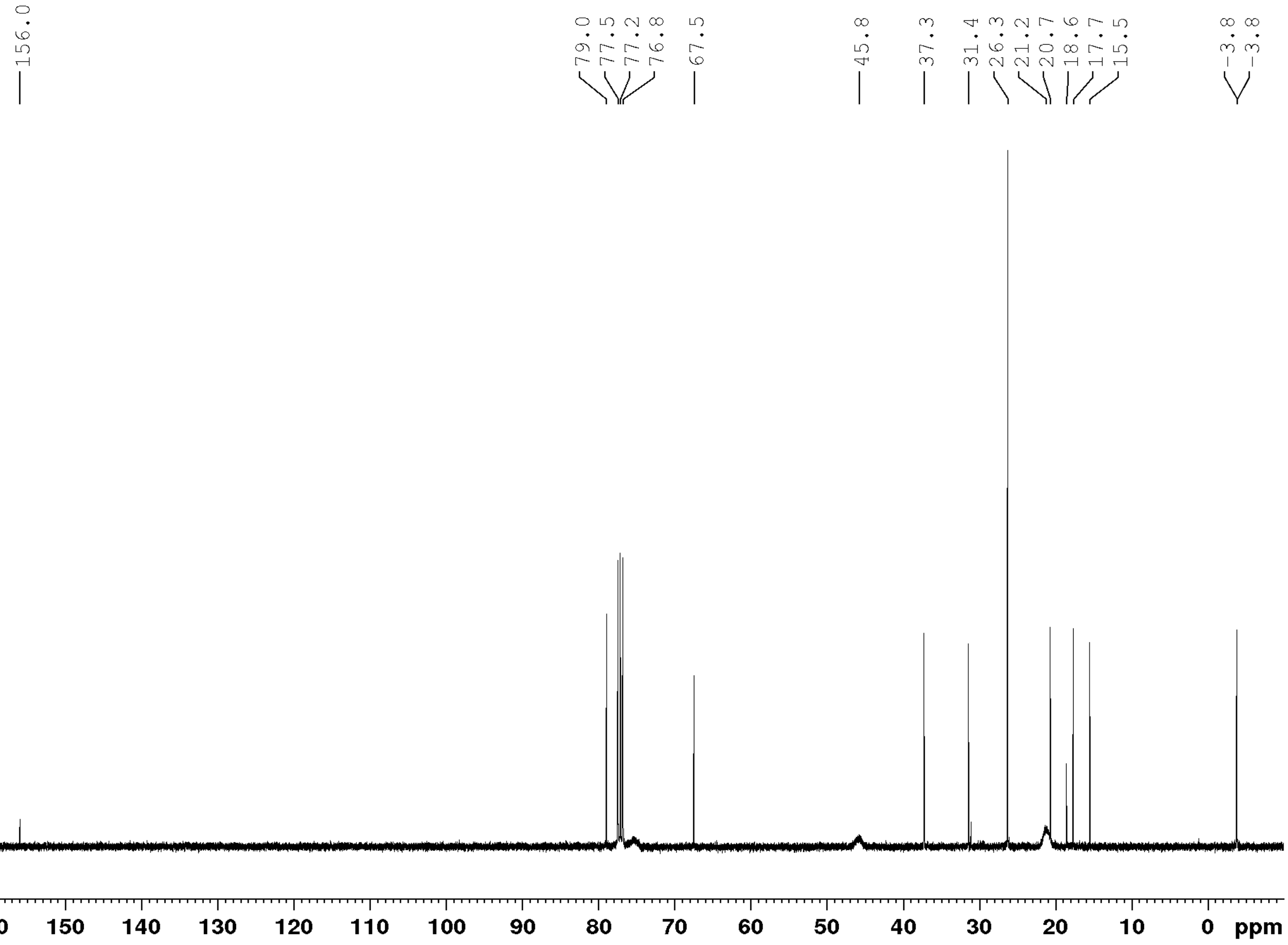
156.0
79.0
77.5
77.2
76.8
67.5
45.8
37.3
31.4
26.3
21.2
20.7
18.6
17.7
15.5
-3.8
-3.8
170
160
150
140
130
120
110
100
90
80
70
60
50
40
30
20
10
0
ppm

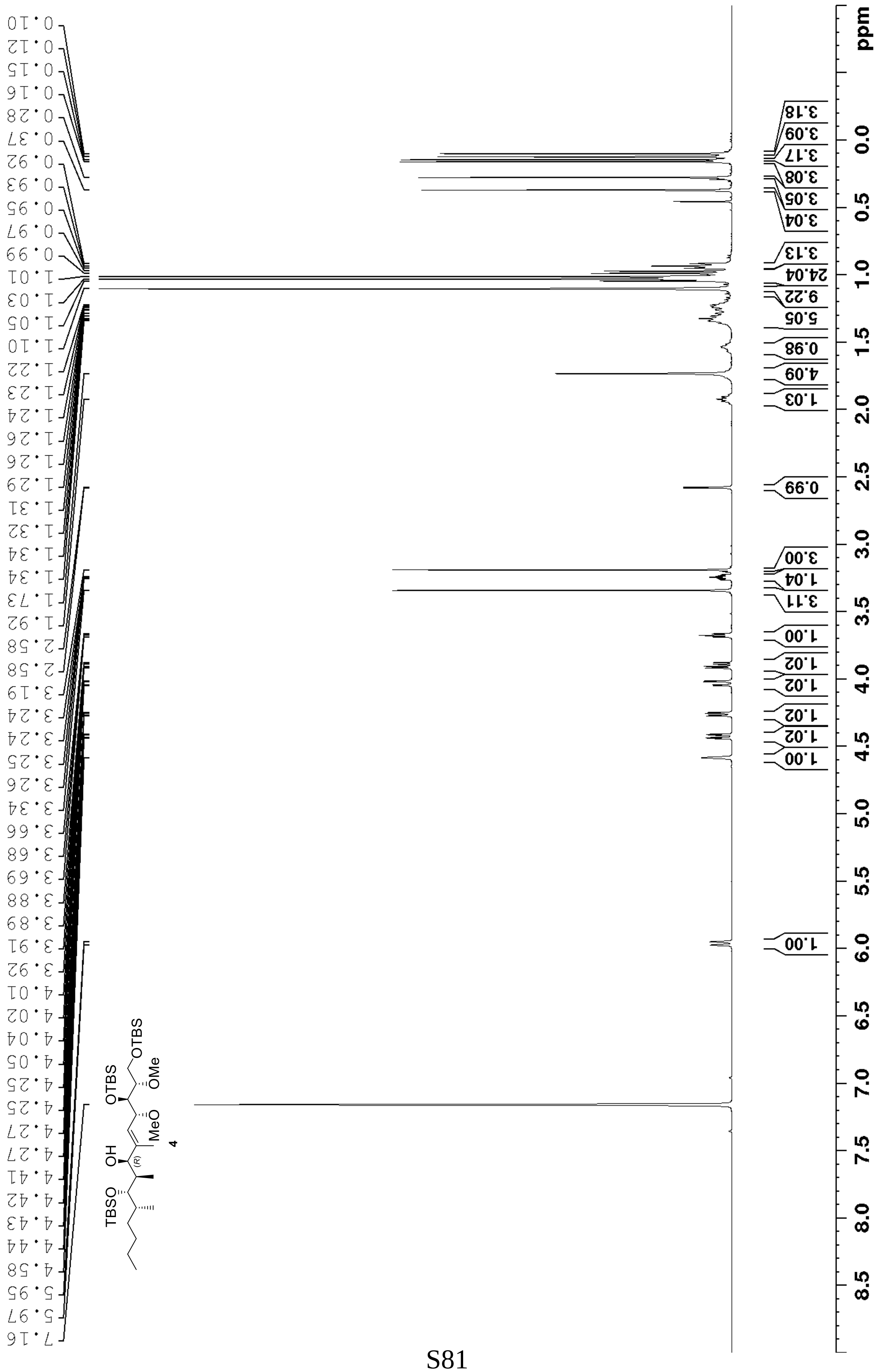
0.10
0.12
0.15
0.16
0.28
0.37
0.92
0.93
0.95
0.97
0.99
1.01
1.03
1.05
1.10
1.22
1.23
1.24
1.26
1.26
1.29
1.31
1.32
1.34
1.34
1.73
1.92
2.58
2.58
3.19
3.24
3.24
3.25
3.26
3.34
3.66
3.68
3.69
3.88
3.89
3.91
3.92
4.01
4.02
4.04
4.05
4.25
4.25
4.27
4.27
4.41
4.42
4.43
4.44
4.58
5.95
5.97
7.16
TBSO
OH
(R)
OTBS
OTBS
MeO
OMe
4
ppm

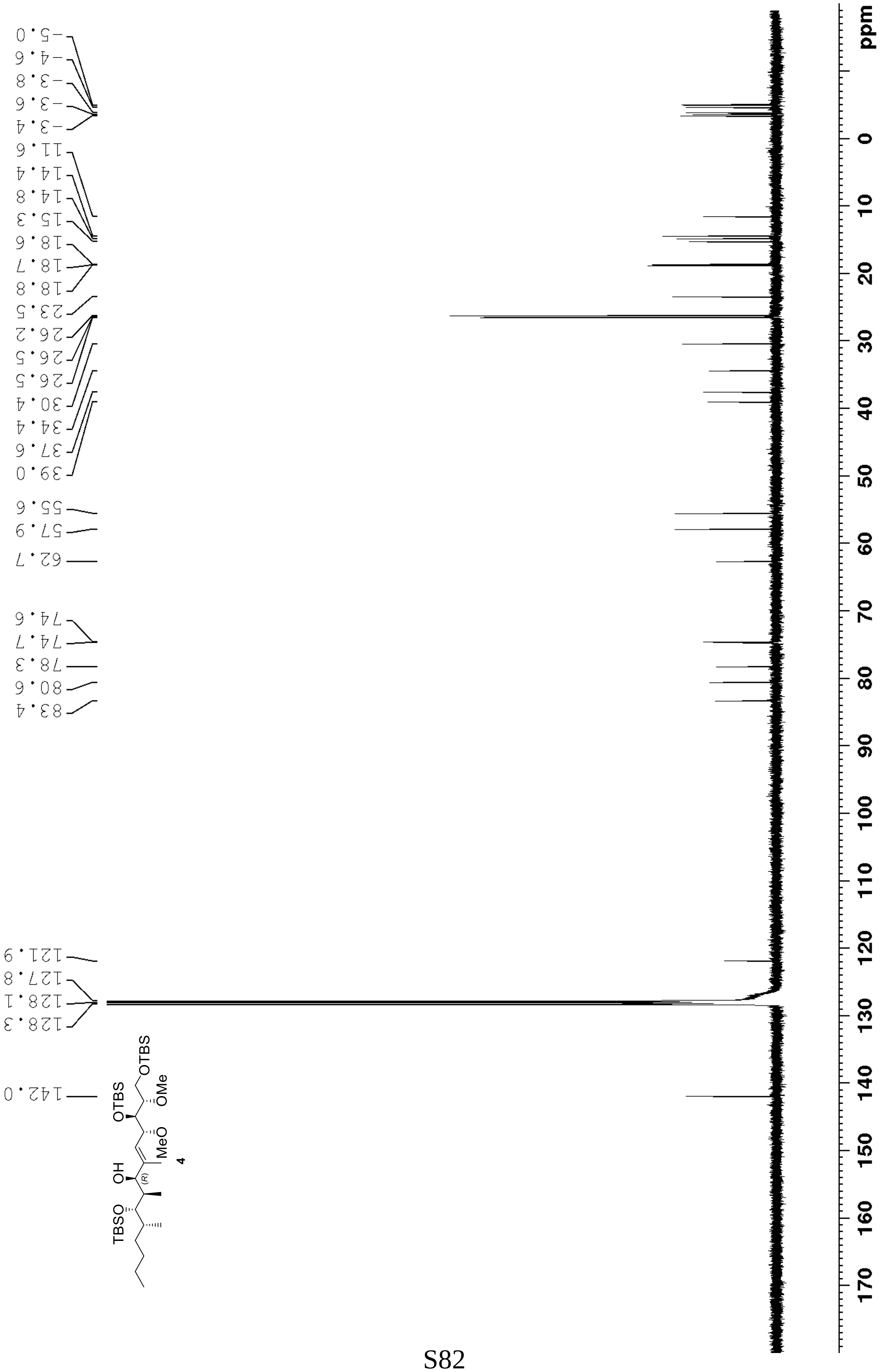
-5.0
-4.6
-3.8
-3.6
-3.4
11.6
14.4
14.8
15.3
18.6
18.7
18.8
23.5
26.2
26.5
26.5
30.4
34.4
37.6
39.0
55.6
57.9
62.7
74.6
74.7
78.3
80.6
83.4
121.9
127.8
128.1
128.3
142.0
TBSO
OH
(R)
MeO
OTBS
OMe
OTBS
4
ppm

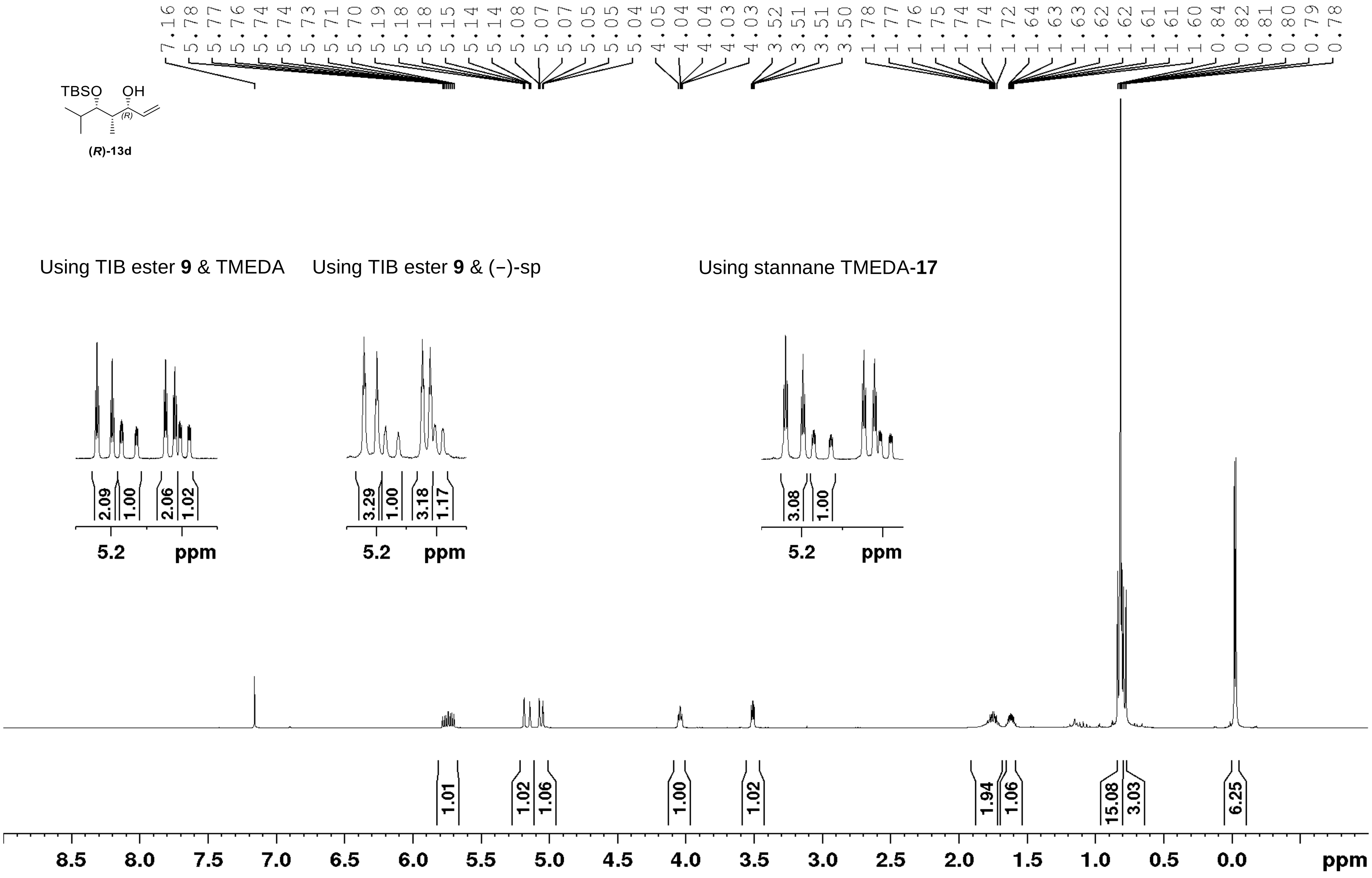

7.16 5.78 5.77 5.76 5.74 5.74 5.73 5.71 5.70 5.19 5.18 5.18 5.15 5.14 5.14 5.08 5.07 5.07 5.05 5.05 5.04 4.05 4.04 4.04 4.03 4.03 3.52 3.51 3.51 3.50 1.78 1.77 1.76 1.75 1.74 1.74 1.72 1.64 1.63 1.63 1.62 1.62 1.61 1.61 1.60 0.84 0.82 0.81 0.80 0.79 0.78
TBSO OH
(R)
(R)-13d
Using TIB ester 9 & TMEDA
Using TIB ester 9 & (−)-sp
Using stannane TMEDA-17
2.09 1.00 2.06 1.02
5.2 ppm
3.29 1.00 3.18 1.17
5.2 ppm
3.08 1.00
5.2 ppm
1.01 1.02 1.06 1.00 1.02 1.94 1.06 15.08 3.03 6.25
8.5 8.0 7.5 7.0 6.5 6.0 5.5 5.0 4.5 4.0 3.5 3.0 2.5 2.0 1.5 1.0 0.5 0.0 ppm

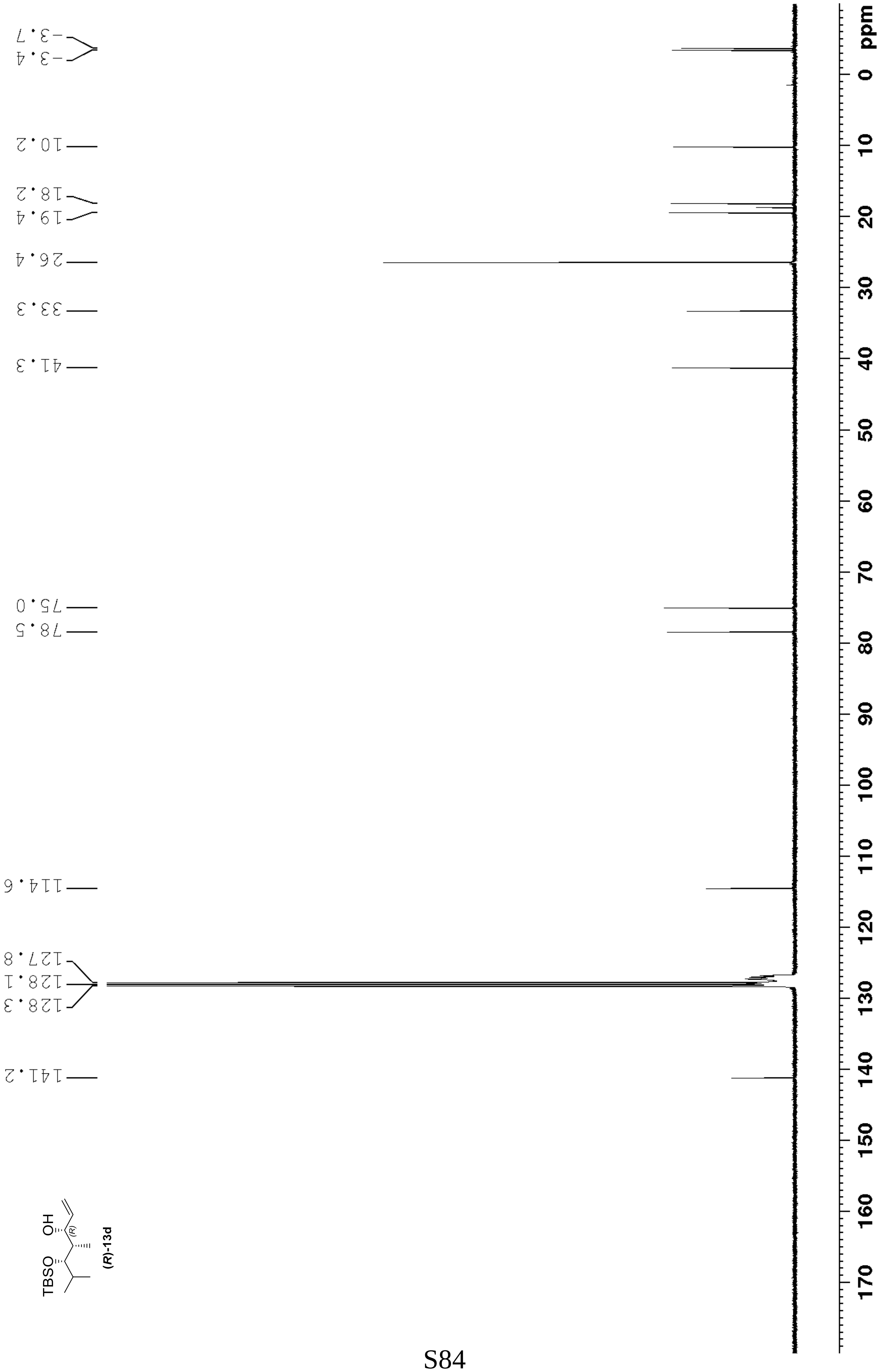
-3.7
-3.4
10.2
18.2
19.4
26.4
33.3
41.3
75.0
78.5
114.6
127.8
128.1
128.3
141.2
ppm
0
10
20
30
40
50
60
70
80
90
100
110
120
130
140
150
160
170
TBSO
OH
(R)
(R)-13d

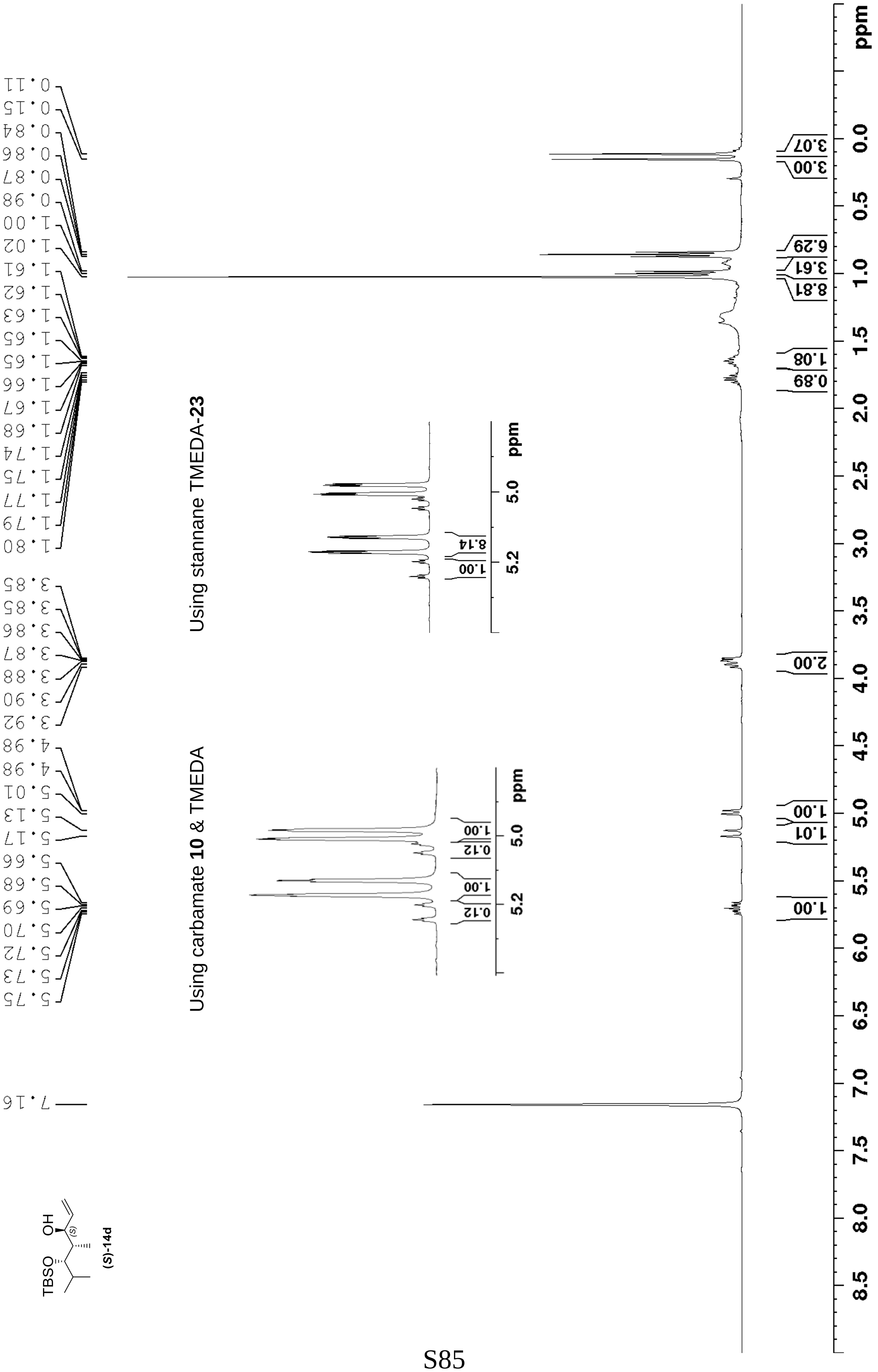

TBSO
OH
(S)
(S)-14d
Using stannane TMEDA-23
Using carbamate 10 & TMEDA
0.11
0.15
0.84
0.86
0.87
0.98
1.00
1.02
1.61
1.62
1.63
1.65
1.65
1.66
1.67
1.68
1.74
1.75
1.77
1.79
1.80
3.85
3.85
3.86
3.87
3.88
3.90
3.92
4.98
4.98
5.01
5.13
5.17
5.66
5.68
5.69
5.70
5.72
5.73
5.75
7.16
3.07
3.00
6.29
3.61
8.81
1.08
0.89
2.00
1.00
1.01
1.00
8.14
1.00
1.00
0.12
1.00
0.12
5.0
5.2
ppm
0.0
0.5
1.0
1.5
2.0
2.5
3.0
3.5
4.0
4.5
5.0
5.5
6.0
6.5
7.0
7.5
8.0
8.5
ppm

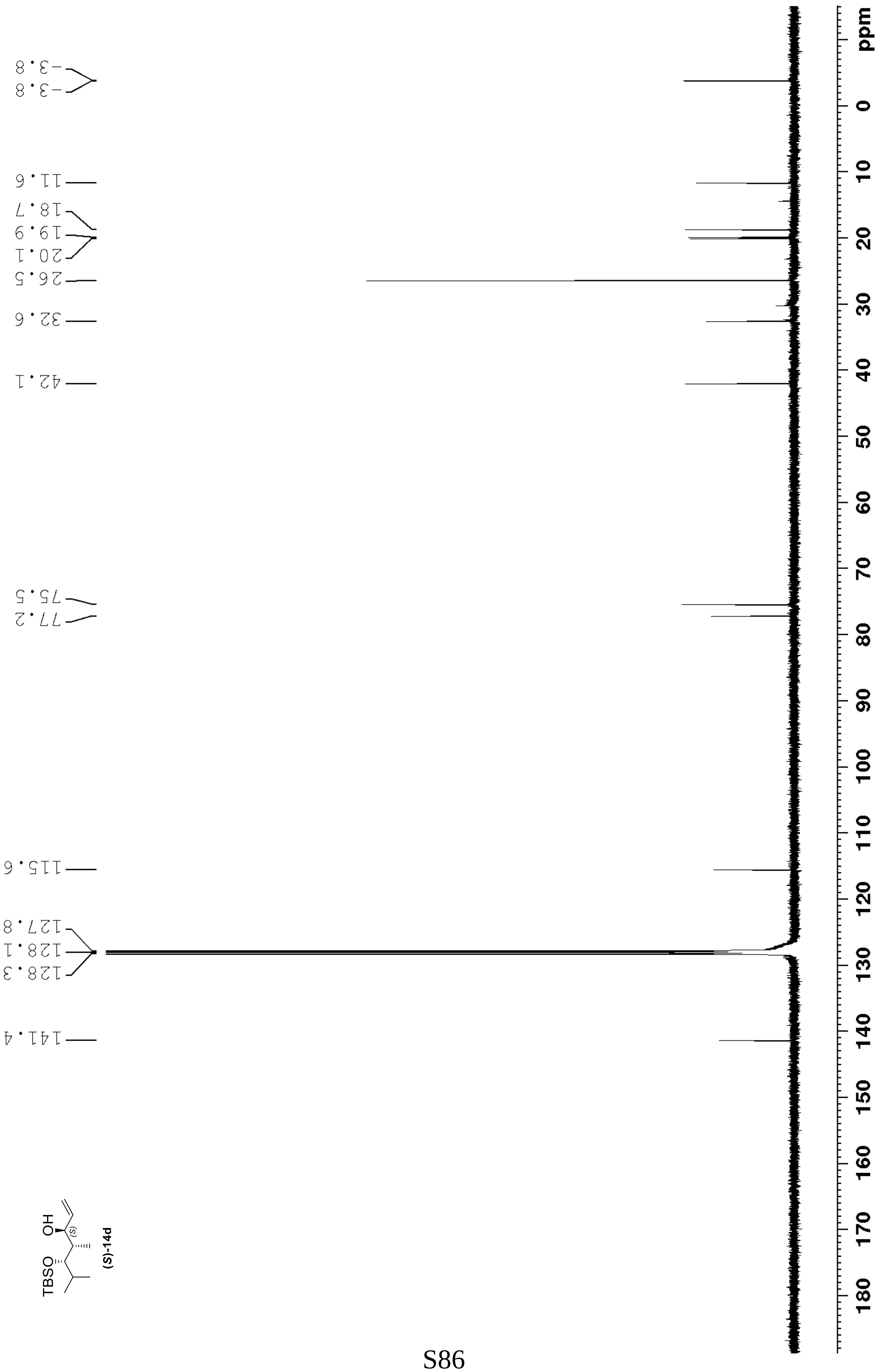

-3.8
-3.8
11.6
18.7
19.9
20.1
26.5
32.6
42.1
75.5
77.2
115.6
127.8
128.1
128.3
141.4
ppm
0
10
20
30
40
50
60
70
80
90
100
110
120
130
140
150
160
170
180
TBSO
OH
(S)
(S)-14d

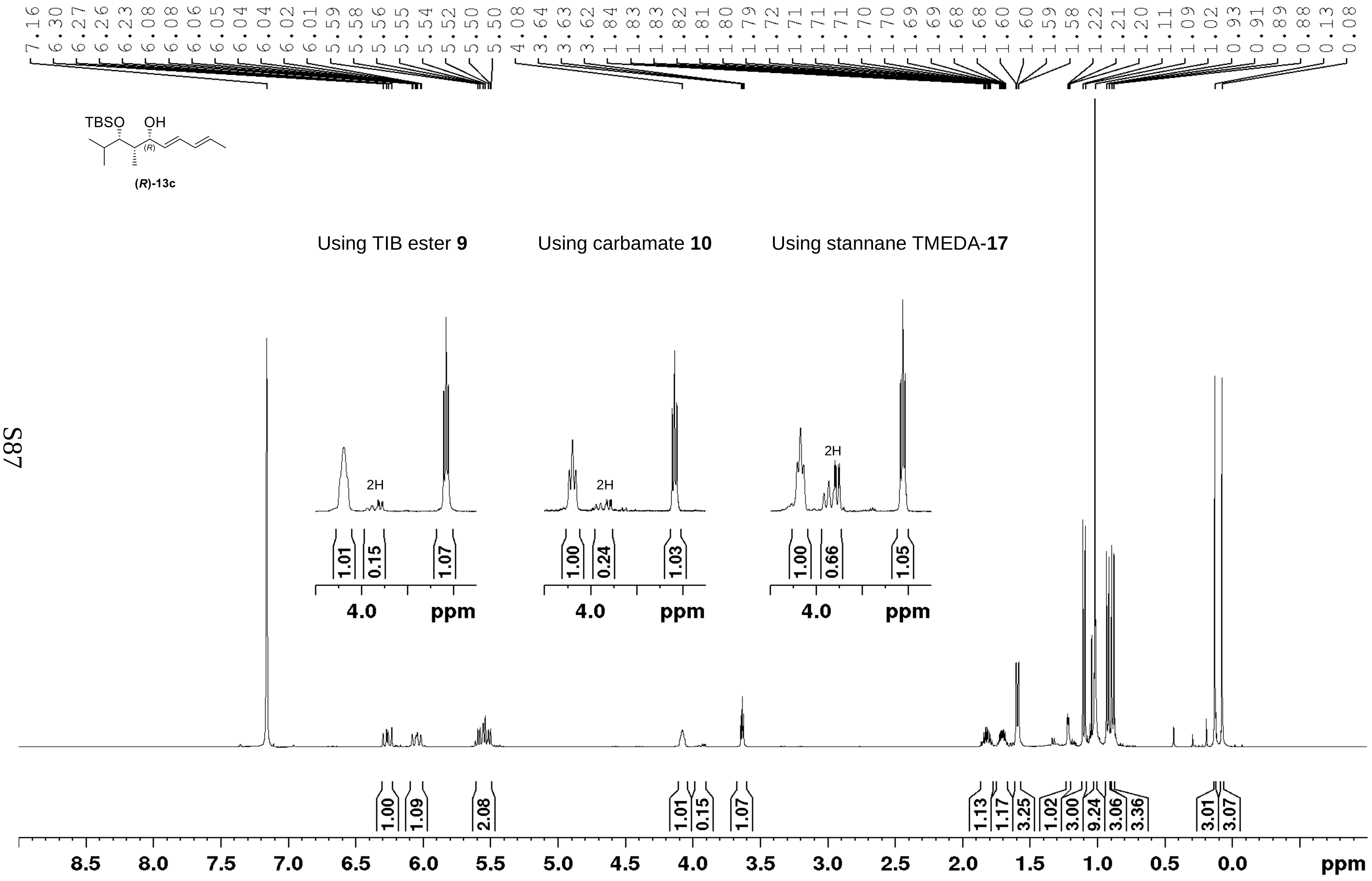
7.16
6.30
6.27
6.26
6.23
6.08
6.08
6.06
6.05
6.04
6.04
6.02
6.01
5.59
5.58
5.56
5.55
5.54
5.52
5.50
5.50
4.08
3.64
3.63
3.62
1.84
1.83
1.83
1.82
1.81
1.80
1.79
1.72
1.71
1.71
1.71
1.70
1.70
1.69
1.69
1.68
1.60
1.60
1.59
1.58
1.22
1.21
1.20
1.11
1.09
1.02
0.93
0.91
0.89
0.88
0.13
0.08
TBSO
OH
(R)
(R)-13c
Using TIB ester 9
Using carbamate 10
Using stannane TMEDA-17
2H
1.01
0.15
1.07
4.0
ppm
2H
1.00
0.24
1.03
4.0
ppm
2H
1.00
0.66
1.05
4.0
ppm
1.00
1.09
2.08
1.01
0.15
1.07
1.13
1.17
3.25
1.02
3.00
9.24
3.06
3.36
3.01
3.07
8.5
8.0
7.5
7.0
6.5
6.0
5.5
5.0
4.5
4.0
3.5
3.0
2.5
2.0
1.5
1.0
0.5
0.0
ppm

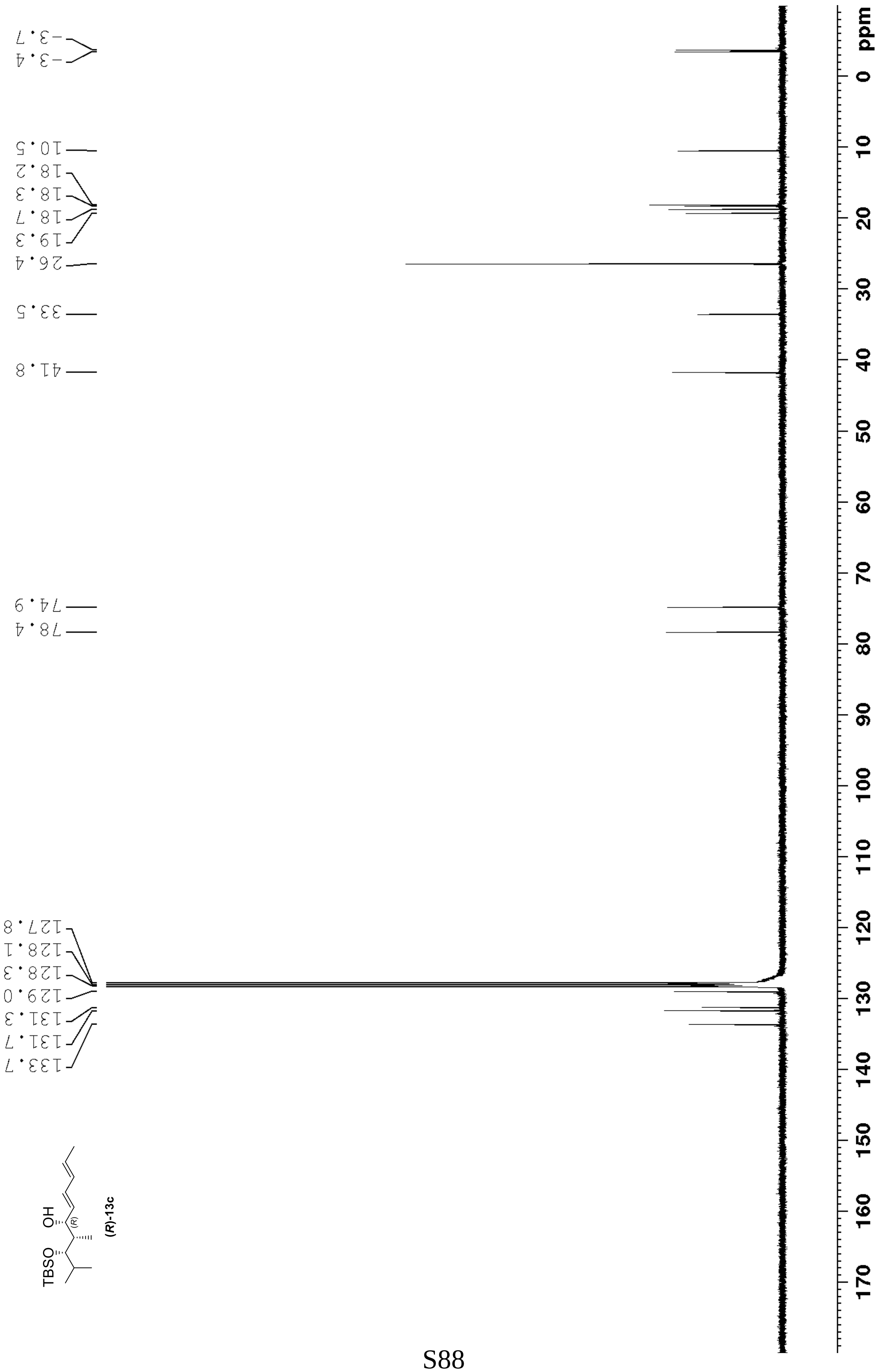

-3.7
-3.4
10.5
18.2
18.3
18.7
19.3
26.4
33.5
41.8
74.9
78.4
127.8
128.1
128.3
129.0
131.3
131.7
133.7
TBSO
OH
(R)
(R)-13c
ppm
0
10
20
30
40
50
60
70
80
90
100
110
120
130
140
150
160
170

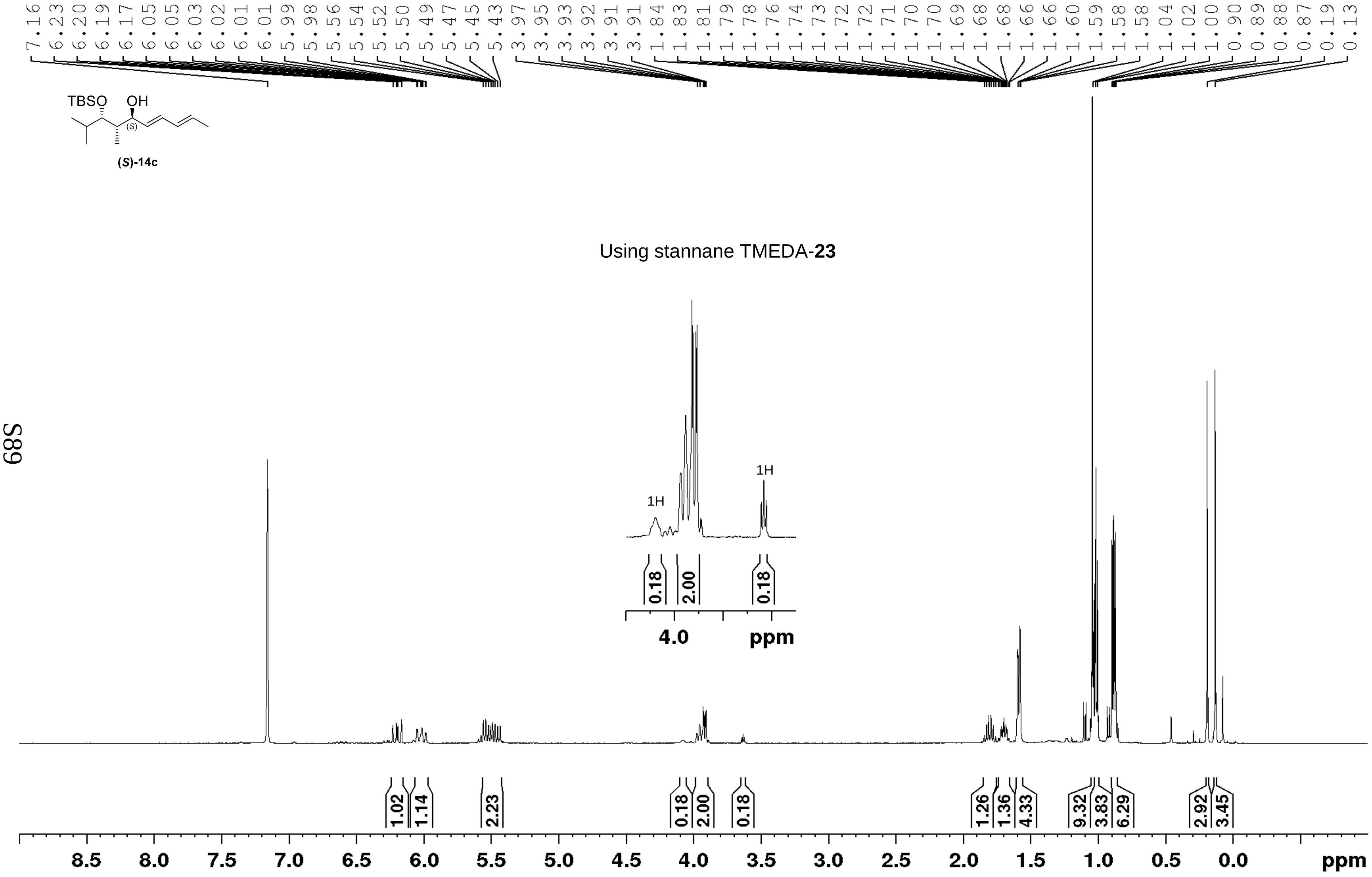
7.16 6.23 6.20 6.19 6.17 6.05 6.05 6.03 6.02 6.01 6.01 5.99 5.98 5.56 5.54 5.52 5.50 5.49 5.47 5.45 5.43 3.97 3.95 3.93 3.92 3.91 3.91 1.84 1.83 1.81 1.79 1.78 1.76 1.74 1.73 1.72 1.72 1.71 1.70 1.70 1.69 1.68 1.68 1.66 1.66 1.60 1.59 1.58 1.58 1.04 1.02 1.00 0.90 0.89 0.88 0.87 0.19 0.13
TBSO
OH
(S)
(S)-14c
Using stannane TMEDA-23
1H
1H
0.18
2.00
0.18
4.0
ppm
1.02
1.14
2.23
0.18
2.00
0.18
1.26
1.36
4.33
9.32
3.83
6.29
2.92
3.45
8.5 8.0 7.5 7.0 6.5 6.0 5.5 5.0 4.5 4.0 3.5 3.0 2.5 2.0 1.5 1.0 0.5 0.0
ppm

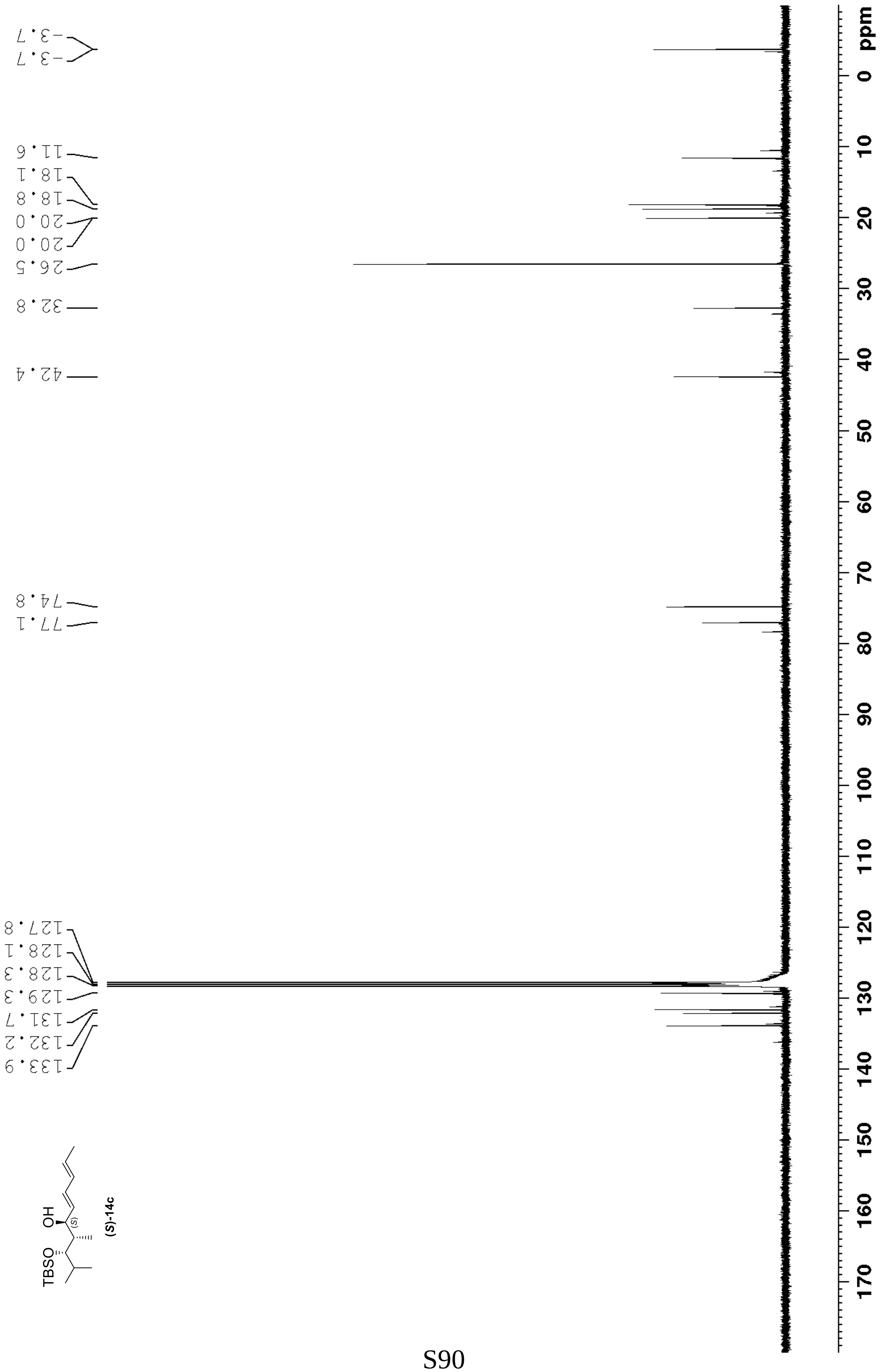
-3.7
-3.7
11.6
18.1
18.8
20.0
20.0
26.5
32.8
42.4
74.8
77.1
127.8
128.1
128.3
129.3
131.7
132.2
133.9
TBSO
OH
(S)
(S)-14c
ppm
0
10
20
30
40
50
60
70
80
90
100
110
120
130
140
150
160
170

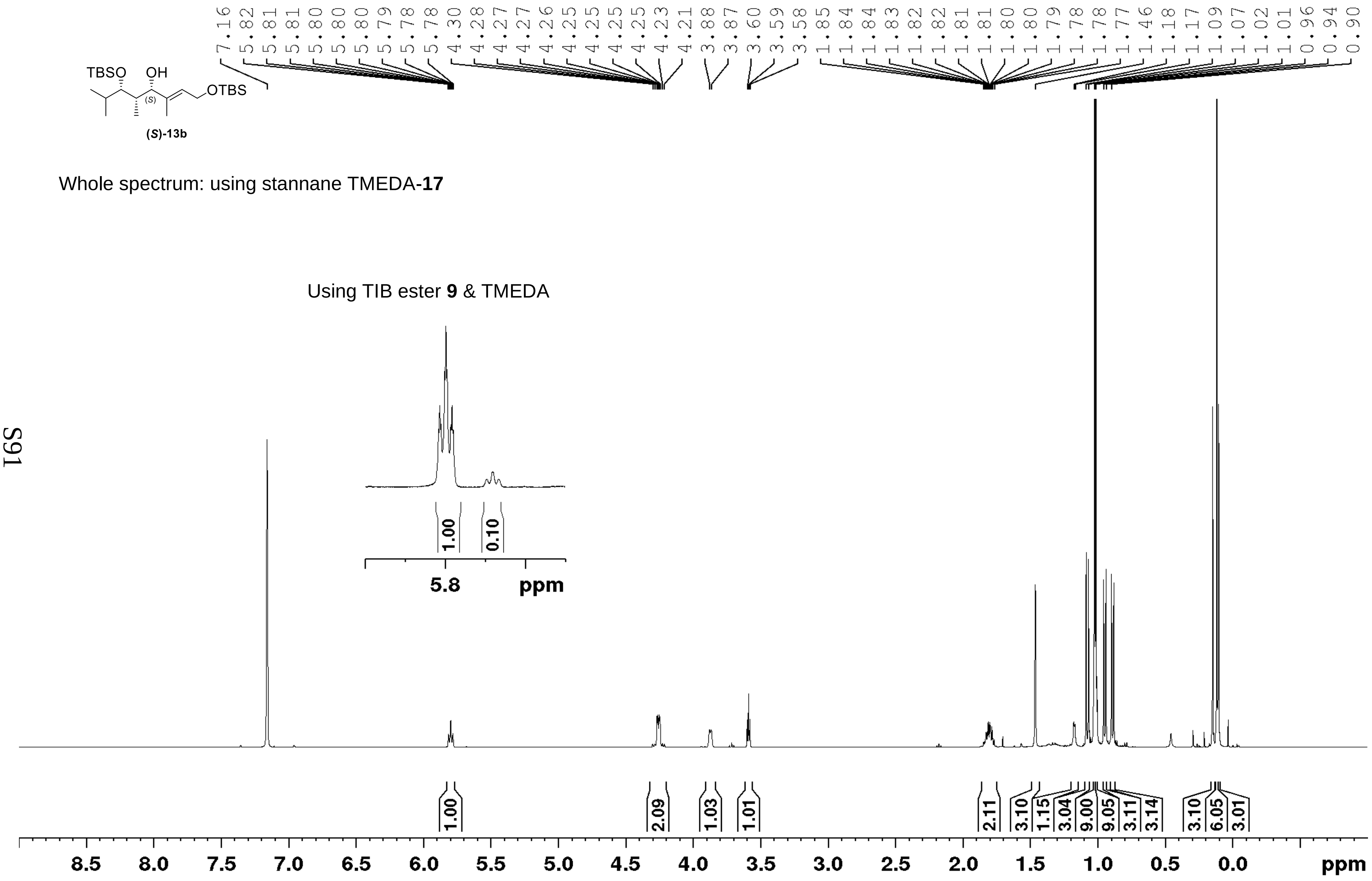
7.16 5.82 5.81 5.81 5.80 5.80 5.80 5.79 5.78 5.78 4.30 4.28 4.27 4.27 4.26 4.25 4.25 4.25 4.25 4.23 4.21 3.88 3.87 3.60 3.59 3.58 1.85 1.84 1.84 1.83 1.82 1.82 1.81 1.81 1.80 1.80 1.79 1.78 1.78 1.77 1.46 1.18 1.17 1.09 1.07 1.02 1.01 0.96 0.94 0.90
TBSO OH
OTBS
(S)
(S)-13b
Whole spectrum: using stannane TMEDA-17
Using TIB ester 9 & TMEDA
1.00 0.10
5.8 ppm
1.00 2.09 1.03 1.01 2.11 3.10 1.15 3.04 9.00 9.05 3.11 3.14 3.10 6.05 3.01
8.5 8.0 7.5 7.0 6.5 6.0 5.5 5.0 4.5 4.0 3.5 3.0 2.5 2.0 1.5 1.0 0.5 0.0 ppm

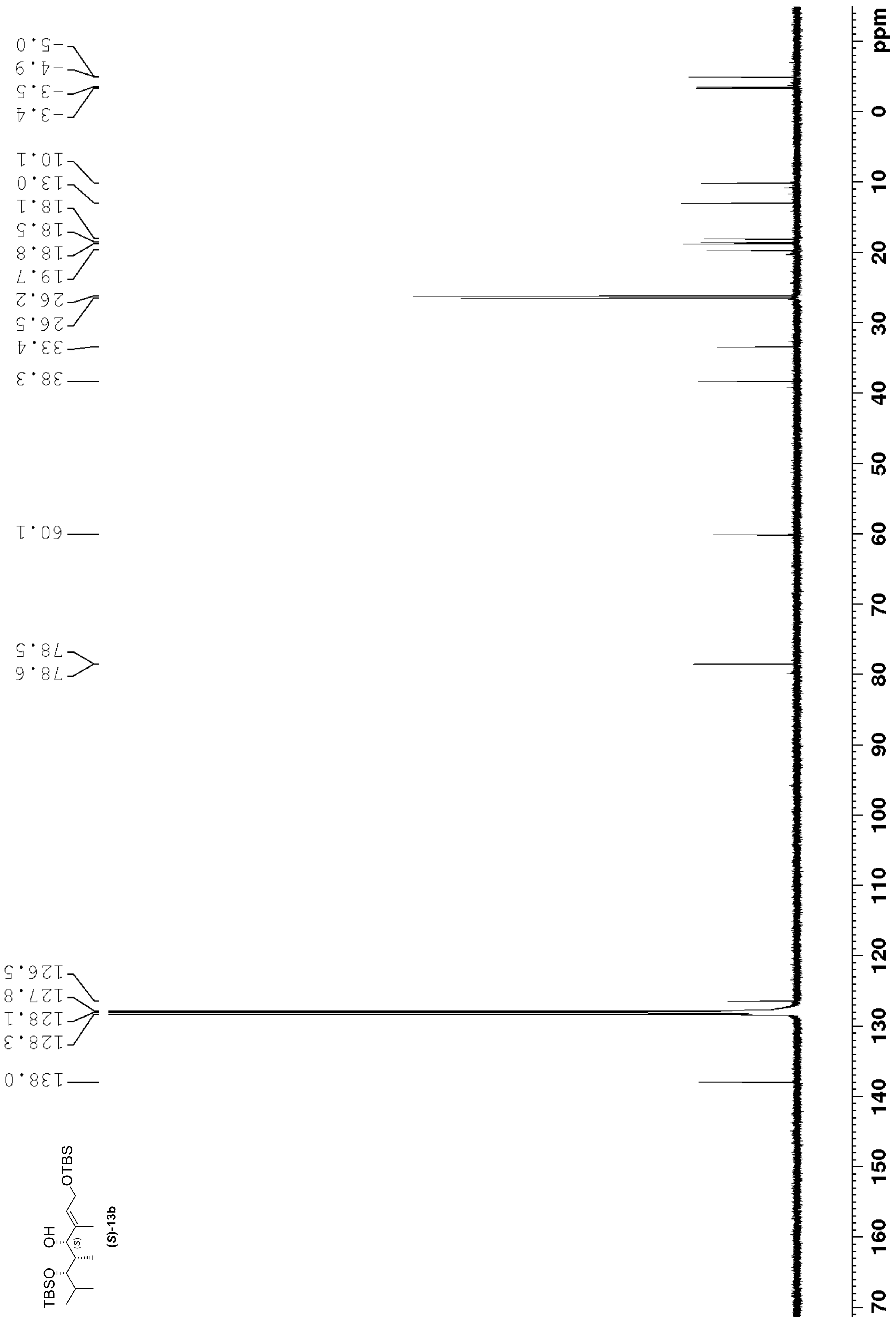

-5.0
-4.9
-3.5
-3.4
10.1
13.0
18.1
18.5
18.8
19.7
26.2
26.5
33.4
38.3
60.1
78.5
78.6
126.5
127.8
128.1
128.3
138.0
TBSO
OH
OTBS
(S)
(S)-13b
ppm
0 10 20 30 40 50 60 70 80 90 100 110 120 130 140 150 160 170

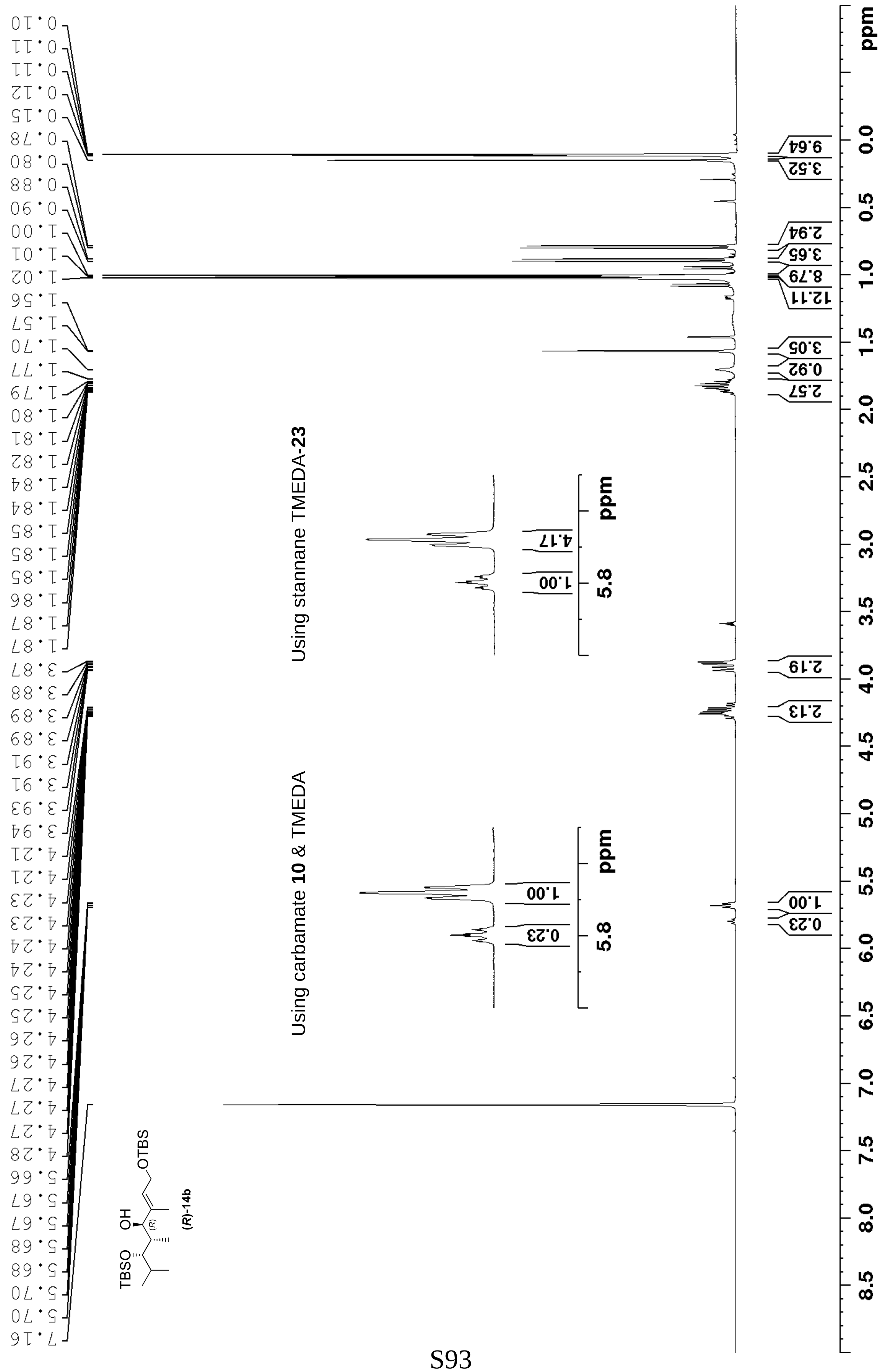

Using stannane TMEDA-23
Using carbamate 10 & TMEDA
(R)-14b
TBSO
OH
OTBS
(R)
4.17
1.00
5.8
ppm
1.00
0.23
5.8
ppm
9.64
3.52
2.94
3.65
8.79
12.11
3.05
0.92
2.57
2.19
2.13
1.00
0.23
ppm
0.0
0.5
1.0
1.5
2.0
2.5
3.0
3.5
4.0
4.5
5.0
5.5
6.0
6.5
7.0
7.5
8.0
8.5
0.10
0.11
0.11
0.12
0.15
0.78
0.80
0.88
0.90
1.00
1.01
1.02
1.56
1.57
1.70
1.77
1.79
1.80
1.81
1.82
1.84
1.84
1.85
1.85
1.85
1.86
1.87
1.87
3.87
3.88
3.89
3.89
3.91
3.91
3.93
3.94
4.21
4.21
4.23
4.23
4.24
4.24
4.25
4.25
4.26
4.26
4.27
4.27
4.27
4.28
5.66
5.67
5.67
5.68
5.68
5.70
5.70
7.16

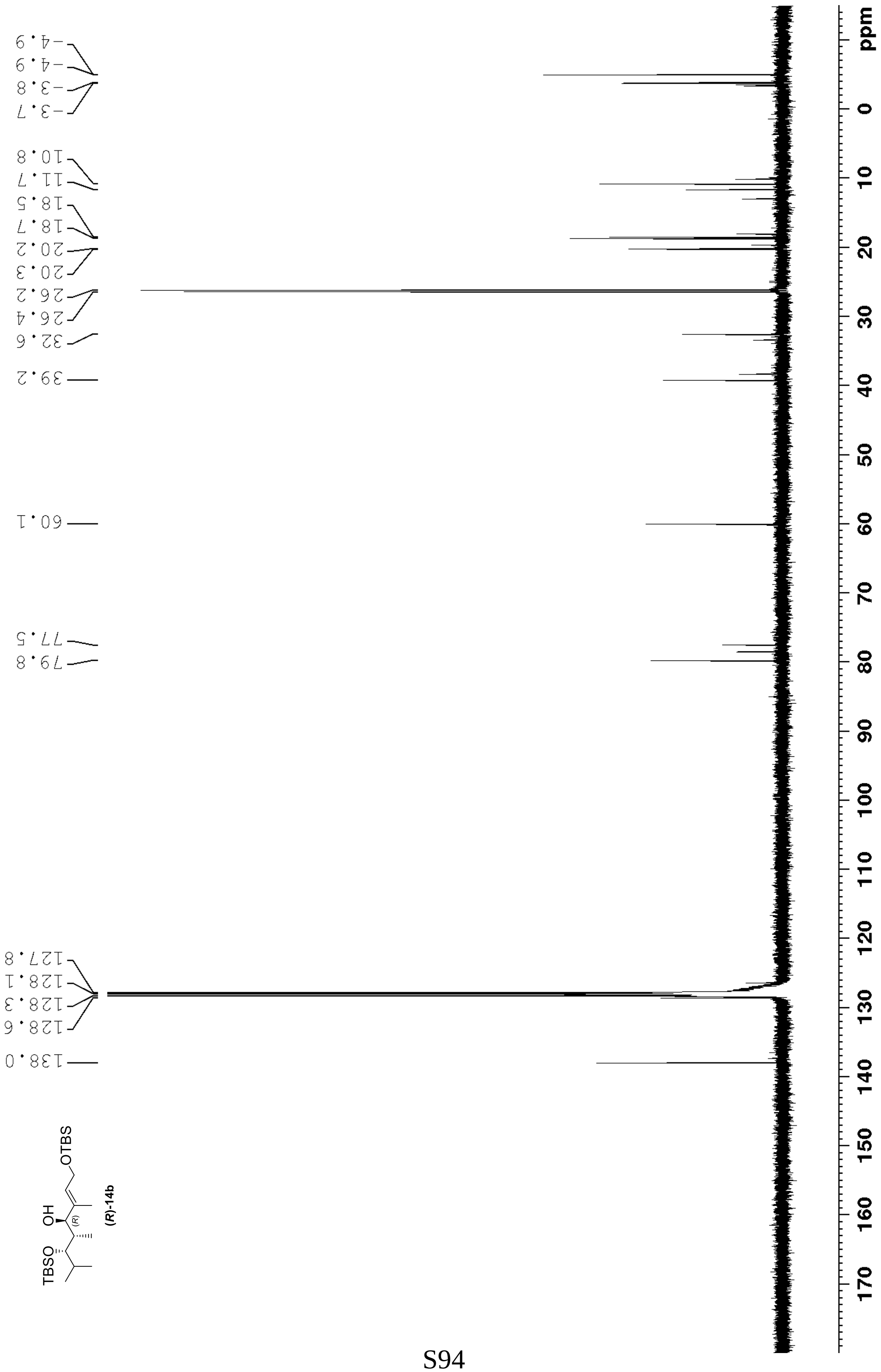
TBSO
OH
OTBS
(R)
(R)-14b
-4.9
-4.9
-3.8
-3.7
10.8
11.7
18.5
18.7
20.2
20.3
26.2
26.4
32.6
39.2
60.1
77.5
79.8
127.8
128.1
128.3
128.6
138.0
ppm
0
10
20
30
40
50
60
70
80
90
100
110
120
130
140
150
160
170

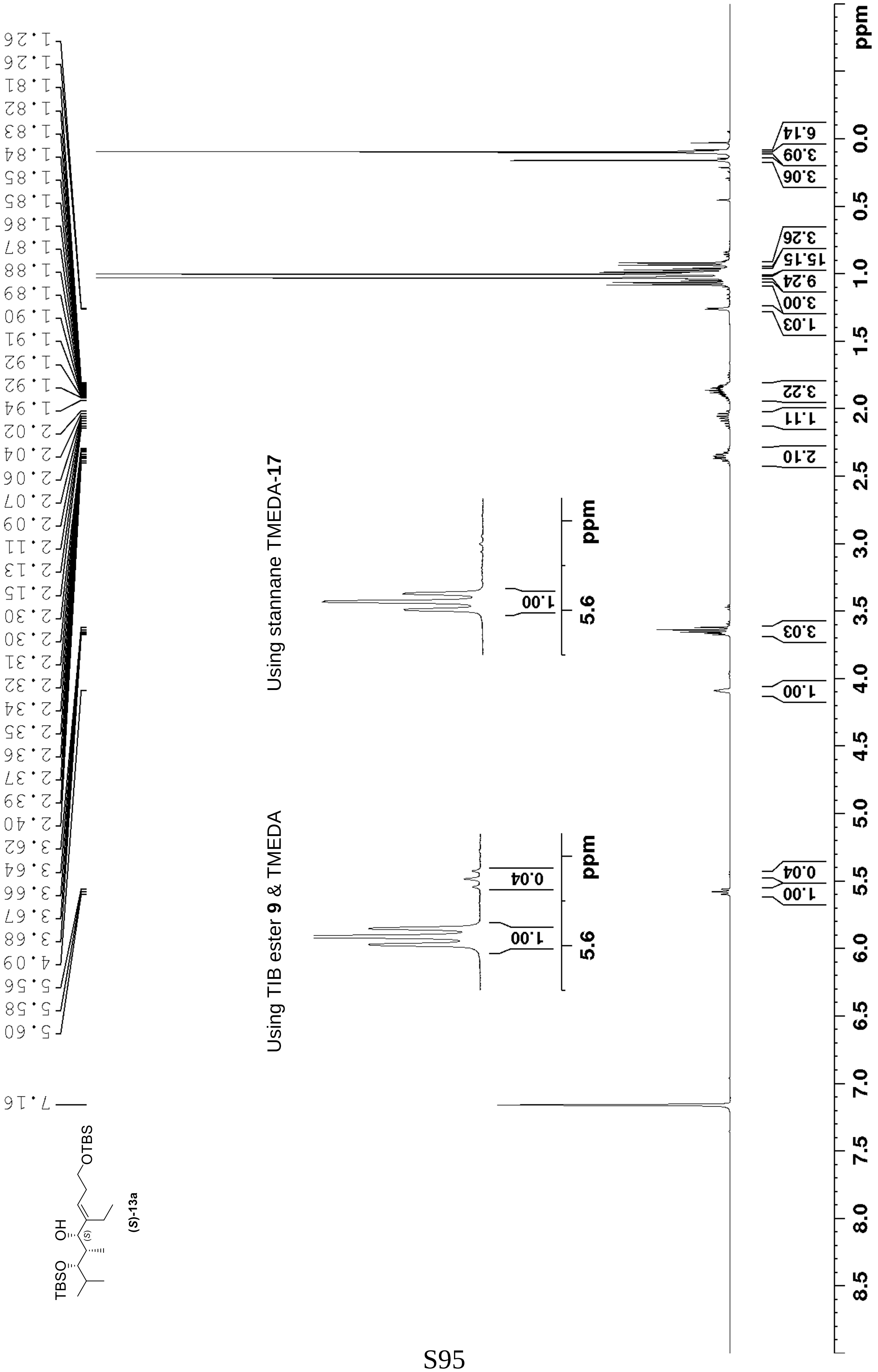
TBSO
OH
OTBS
(S)-13a
Using TIB ester 9 & TMEDA
Using stannane TMEDA-17
ppm

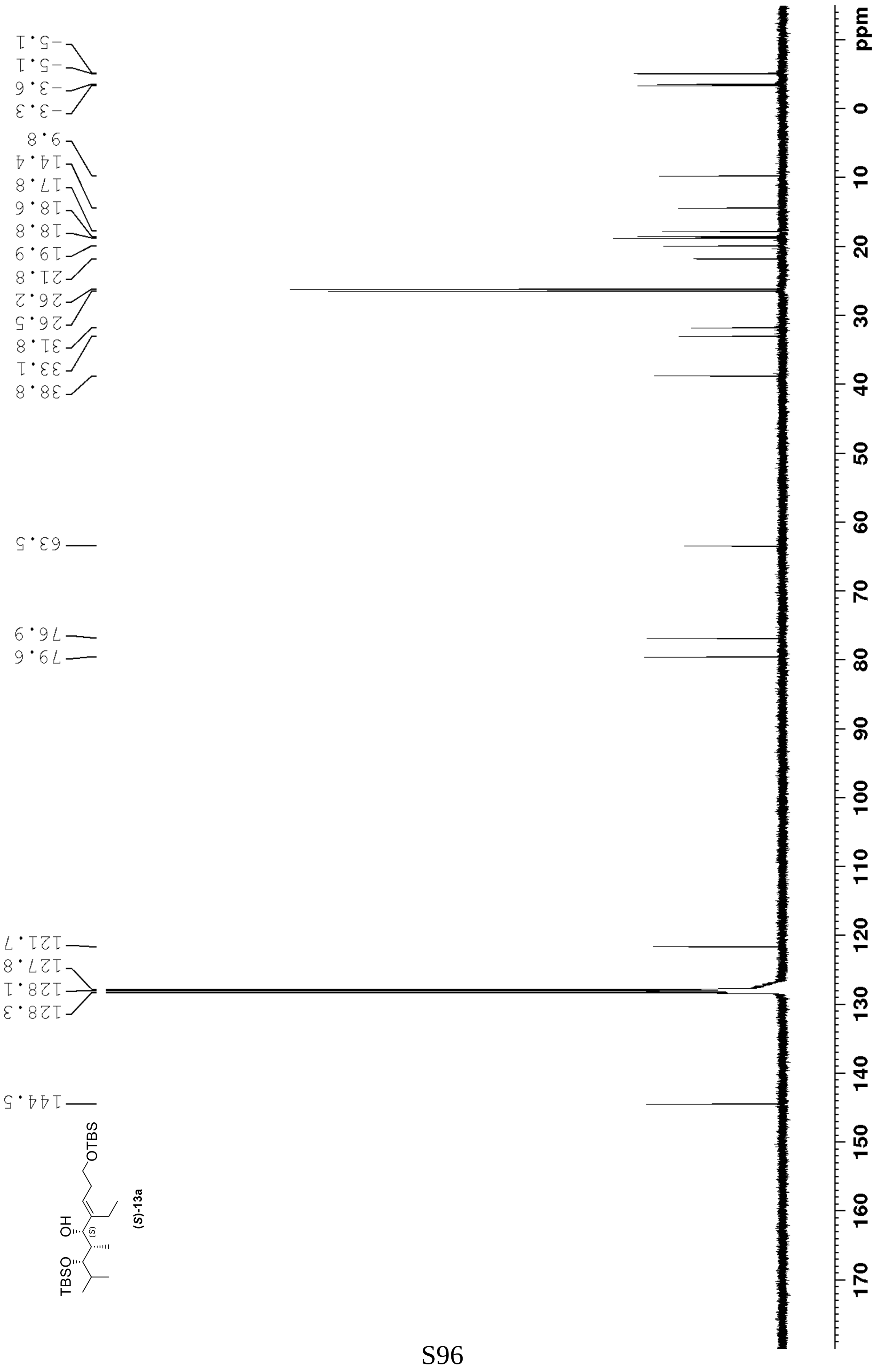
-5.1
-5.1
-3.6
-3.3
9.8
14.4
17.8
18.6
18.8
19.9
21.8
26.2
26.5
31.8
33.1
38.8
63.5
76.9
79.6
121.7
127.8
128.1
128.3
144.5
TBSO
OH
OTBS
(S)-13a
ppm
0
10
20
30
40
50
60
70
80
90
100
110
120
130
140
150
160
170

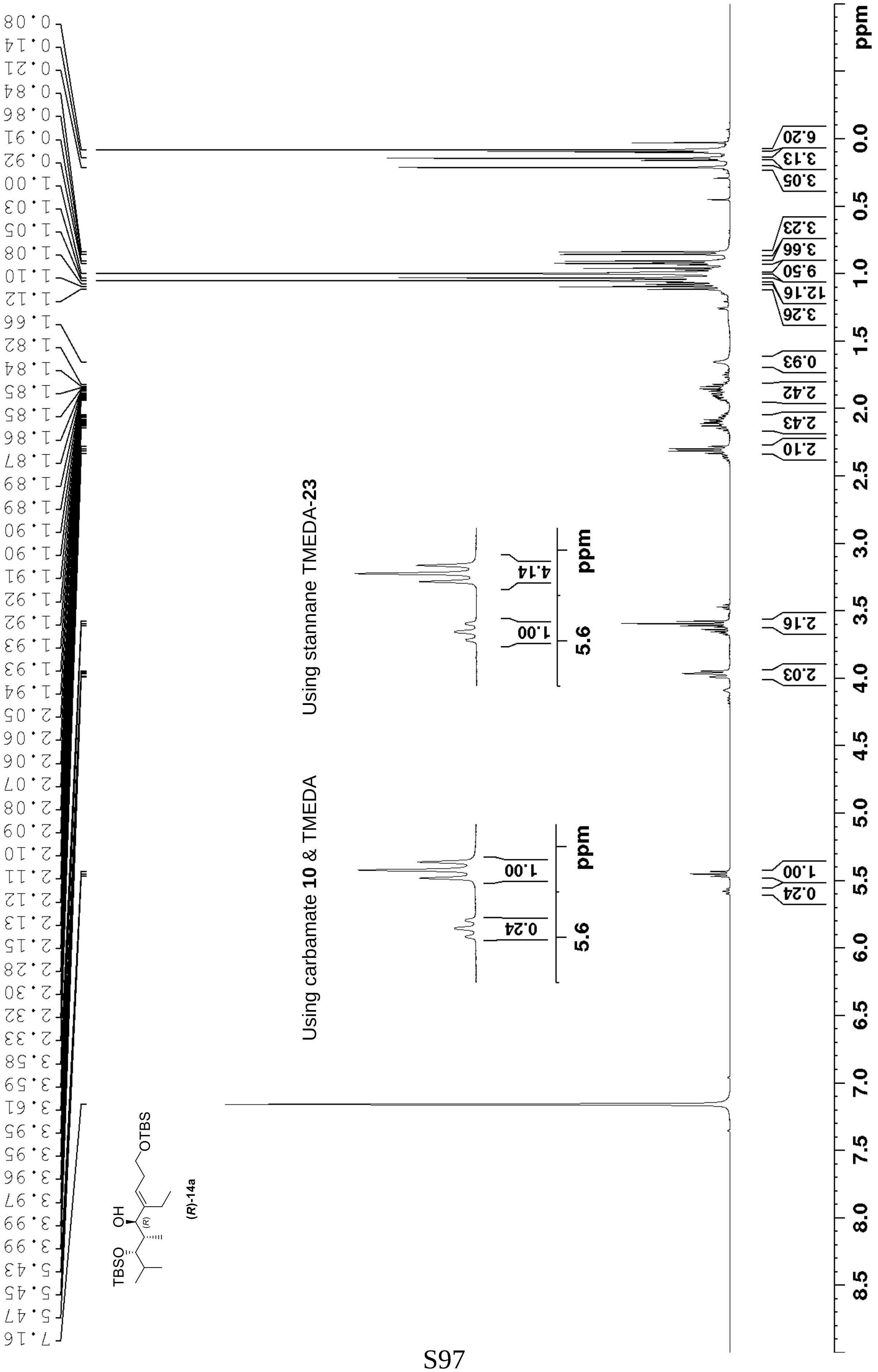

Using stannane TMEDA-23
Using carbamate 10 & TMEDA
TBSO
OH
OTBS
(R)-14a
ppm

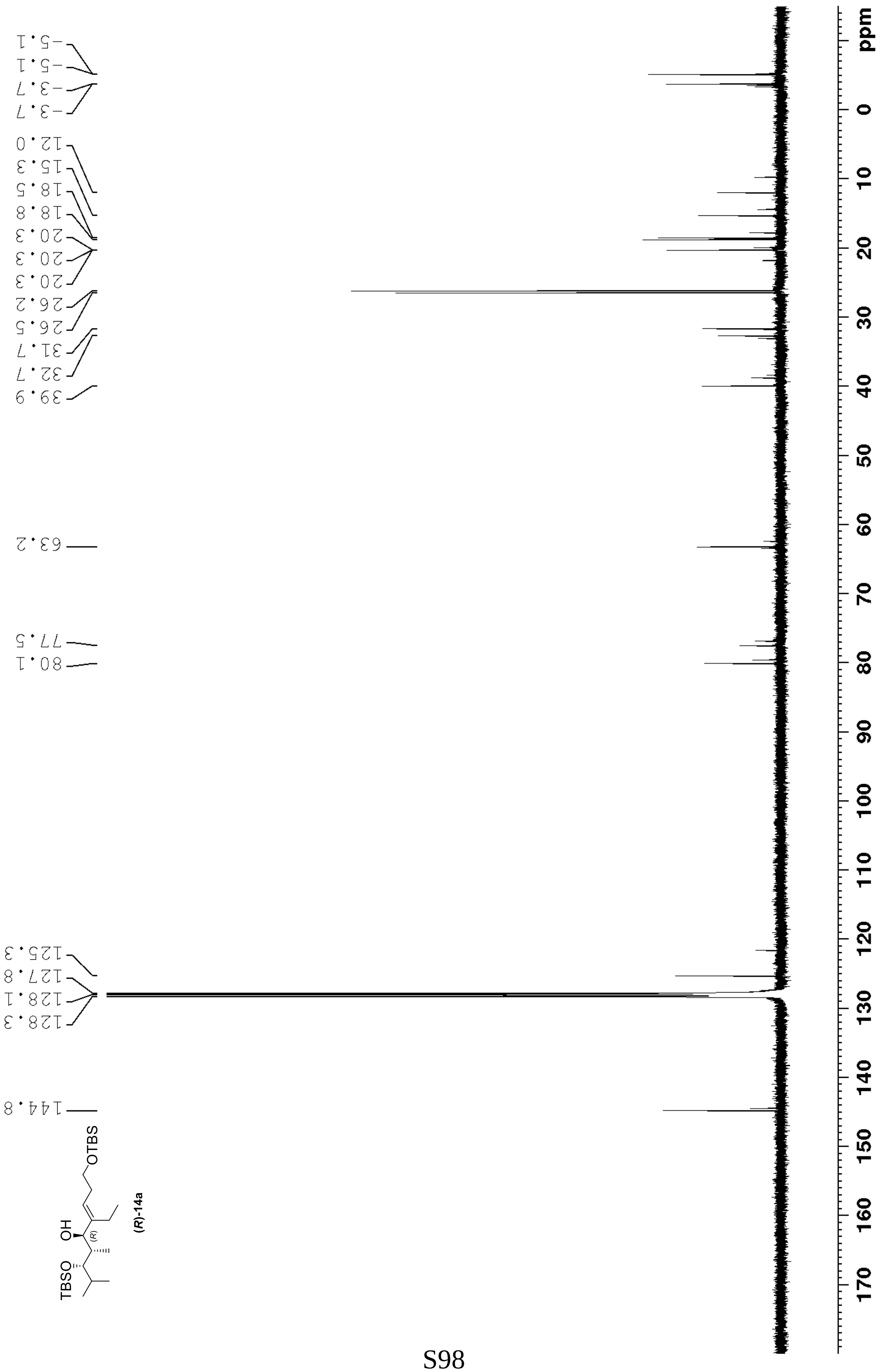

-5.1
-5.1
-3.7
-3.7
12.0
15.3
18.5
18.8
20.3
20.3
20.3
26.2
26.5
31.7
32.7
39.9
63.2
77.5
80.1
125.3
127.8
128.1
128.3
144.8
TBSO
OH
OTBS
(R)-14a
ppm

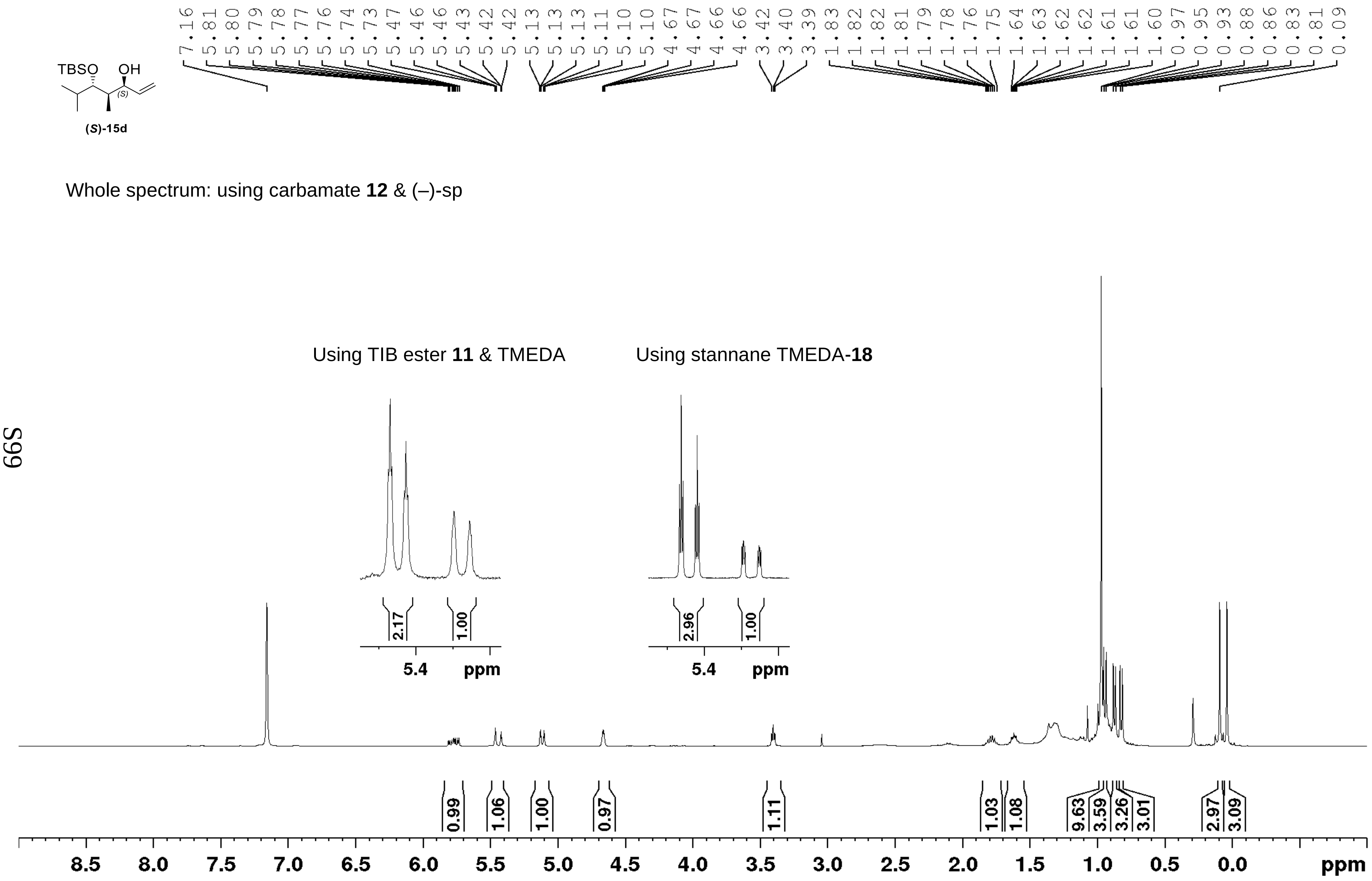
TBSO OH
(S)
(S)-15d
7.16 5.81 5.80 5.79 5.78 5.77 5.76 5.74 5.73 5.47 5.46 5.46 5.43 5.42 5.42 5.13 5.13 5.13 5.11 5.10 5.10 4.67 4.67 4.66 4.66 3.42 3.40 3.39 1.83 1.82 1.82 1.81 1.79 1.78 1.76 1.75 1.64 1.63 1.62 1.62 1.61 1.61 1.60 0.97 0.95 0.93 0.88 0.86 0.83 0.81 0.09
Whole spectrum: using carbamate 12 & (–)-sp
Using TIB ester 11 & TMEDA
Using stannane TMEDA-18
2.17 1.00
5.4 ppm
2.96 1.00
5.4 ppm
0.99 1.06 1.00 0.97 1.11 1.03 1.08 9.63 3.59 3.26 3.01 2.97 3.09
8.5 8.0 7.5 7.0 6.5 6.0 5.5 5.0 4.5 4.0 3.5 3.0 2.5 2.0 1.5 1.0 0.5 0.0 ppm

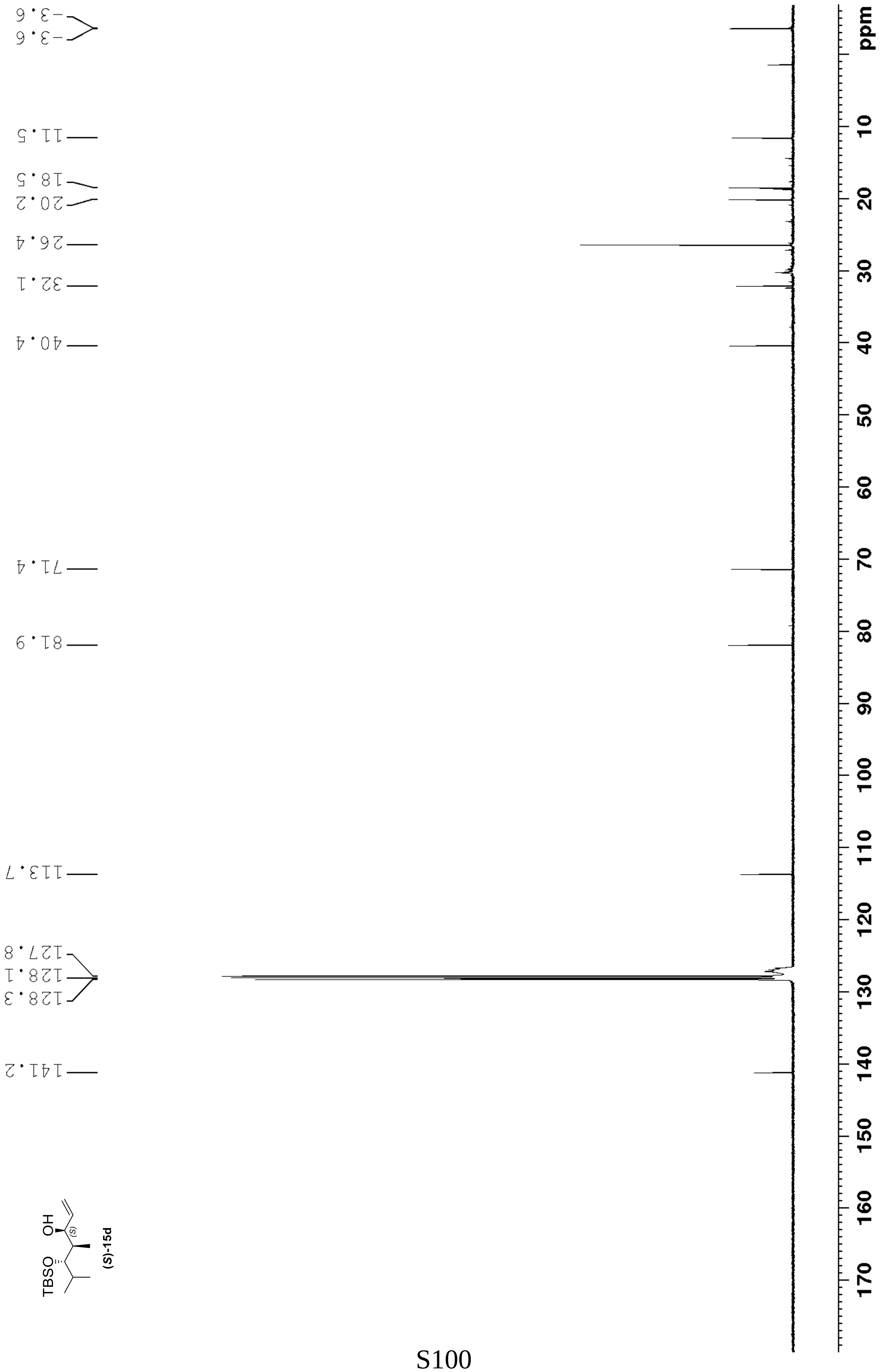
-3.6
-3.6
11.5
18.5
20.2
26.4
32.1
40.4
71.4
81.9
113.7
127.8
128.1
128.3
141.2
TBSO
OH
(S)
(S)-15d
ppm

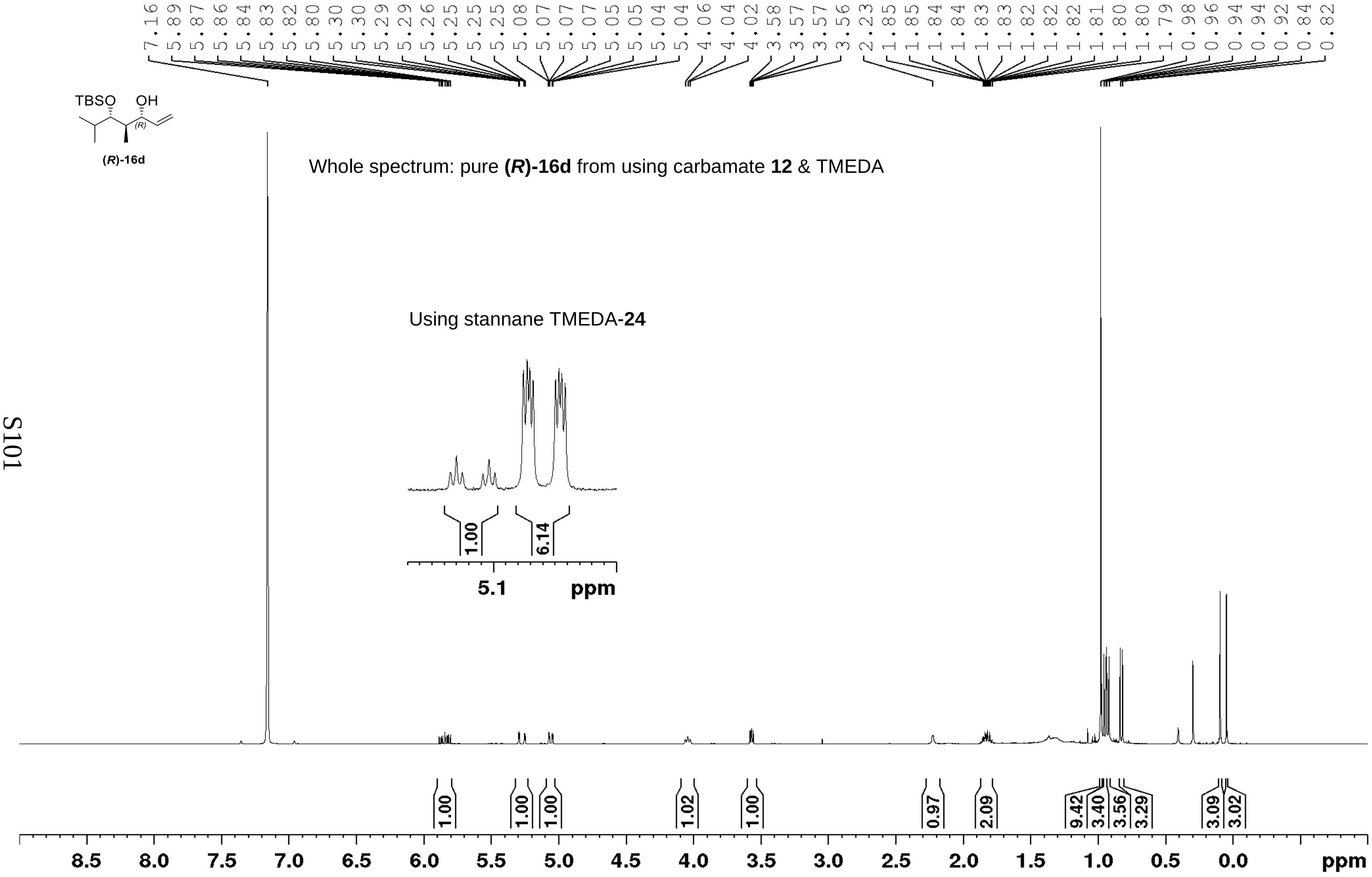

TBSO
OH
(R)
(R)-16d
7.16
5.89
5.87
5.86
5.84
5.83
5.82
5.80
5.30
5.30
5.29
5.29
5.26
5.25
5.25
5.25
5.08
5.07
5.07
5.07
5.05
5.05
5.04
5.04
4.06
4.04
4.02
3.58
3.57
3.57
3.56
2.23
1.85
1.85
1.84
1.84
1.83
1.83
1.82
1.82
1.82
1.81
1.80
1.80
1.79
0.98
0.96
0.94
0.94
0.92
0.84
0.82
Whole spectrum: pure (R)-16d from using carbamate 12 & TMEDA
Using stannane TMEDA-24
1.00
6.14
5.1
ppm
1.00
1.00
1.00
1.02
1.00
0.97
2.09
9.42
3.40
3.56
3.29
3.09
3.02
8.5
8.0
7.5
7.0
6.5
6.0
5.5
5.0
4.5
4.0
3.5
3.0
2.5
2.0
1.5
1.0
0.5
0.0
ppm

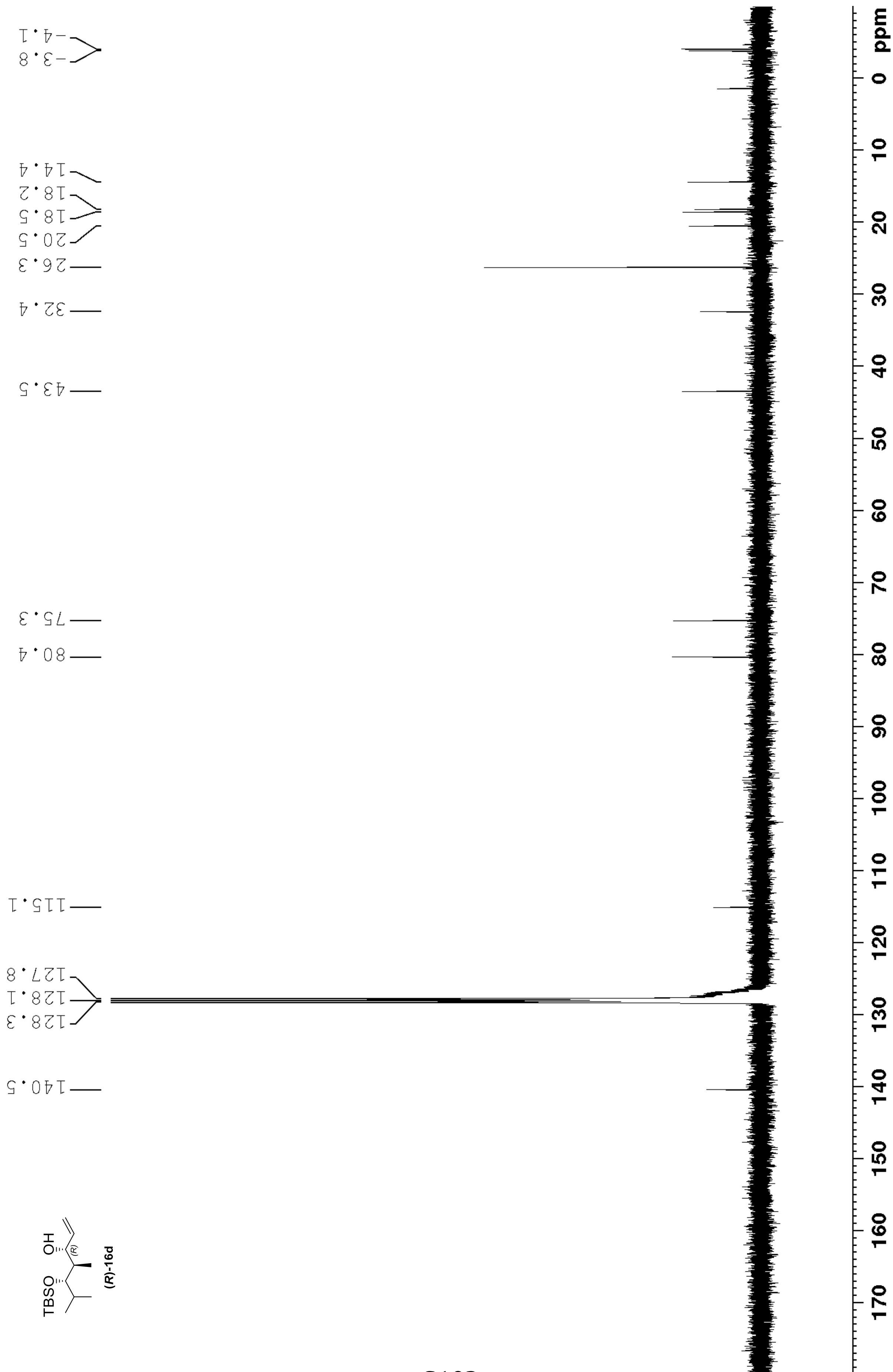
-4.1
-3.8
14.4
18.2
18.5
20.5
26.3
32.4
43.5
75.3
80.4
115.1
127.8
128.1
128.3
140.5
ppm
TBSO
OH
(R)-16d

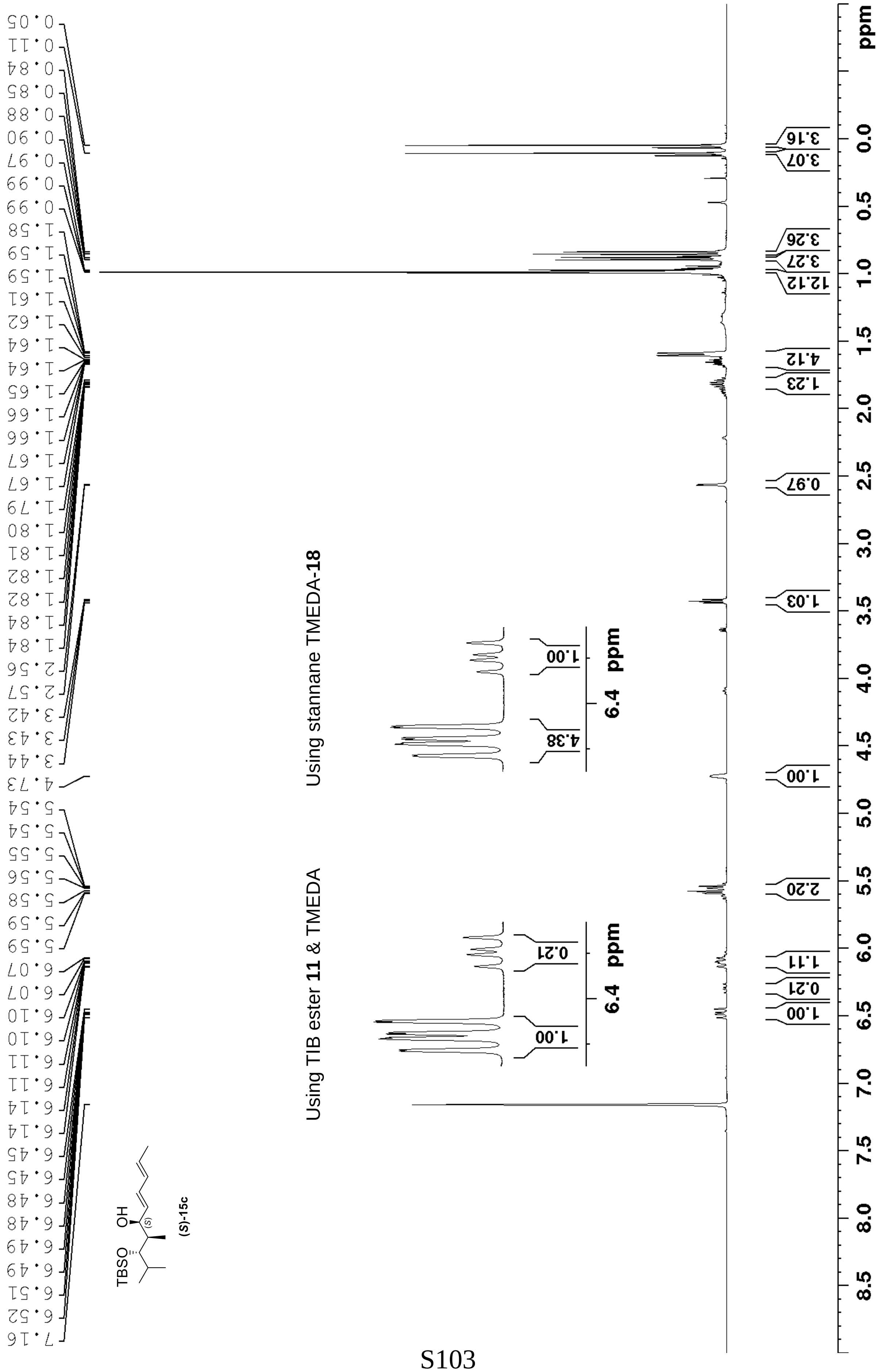
0.05 0.11 0.84 0.85 0.88 0.90 0.97 0.99 0.99 1.58 1.59 1.59 1.61 1.62 1.64 1.64 1.65 1.66 1.66 1.67 1.67 1.79 1.80 1.81 1.82 1.82 1.84 1.84 2.56 2.57 3.42 3.43 3.44 4.73 5.54 5.54 5.55 5.56 5.58 5.59 5.59 6.07 6.07 6.10 6.10 6.11 6.11 6.14 6.14 6.45 6.45 6.48 6.48 6.49 6.49 6.51 6.52 7.16
TBSO
OH
(S)
(S)-15c
Using stannane TMEDA-18
Using TIB ester 11 & TMEDA
1.00
4.38
6.4 ppm
0.21
1.00
6.4 ppm
3.16
3.07
3.26
3.27
12.12
4.12
1.23
0.97
1.03
1.00
2.20
1.11
0.21
1.00
ppm

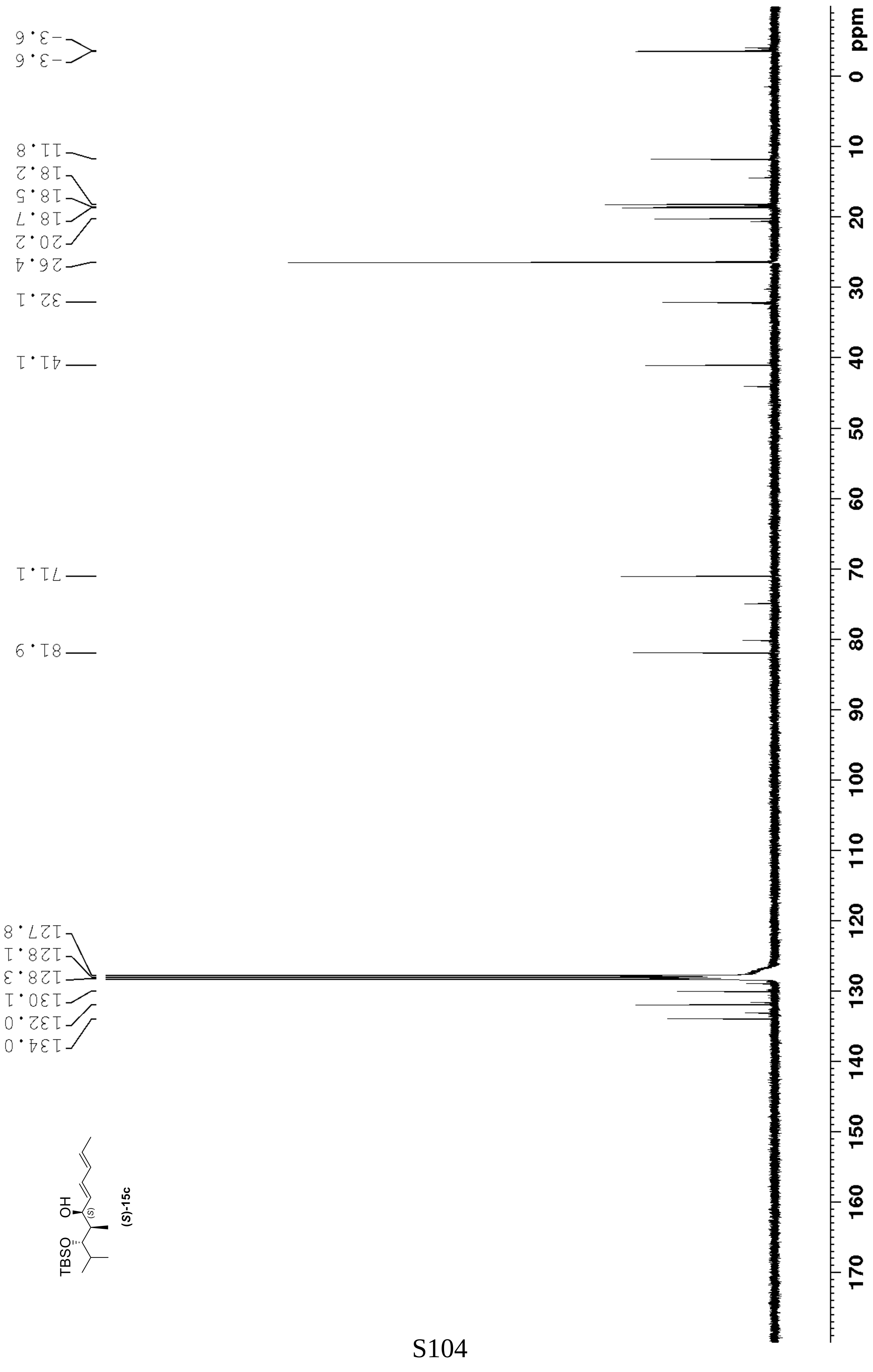
-3.6
-3.6
11.8
18.2
18.5
18.7
20.2
26.4
32.1
41.1
71.1
81.9
127.8
128.1
128.3
130.1
132.0
134.0
TBSO
OH
(S)
(S)-15c
ppm
0
10
20
30
40
50
60
70
80
90
100
110
120
130
140
150
160
170

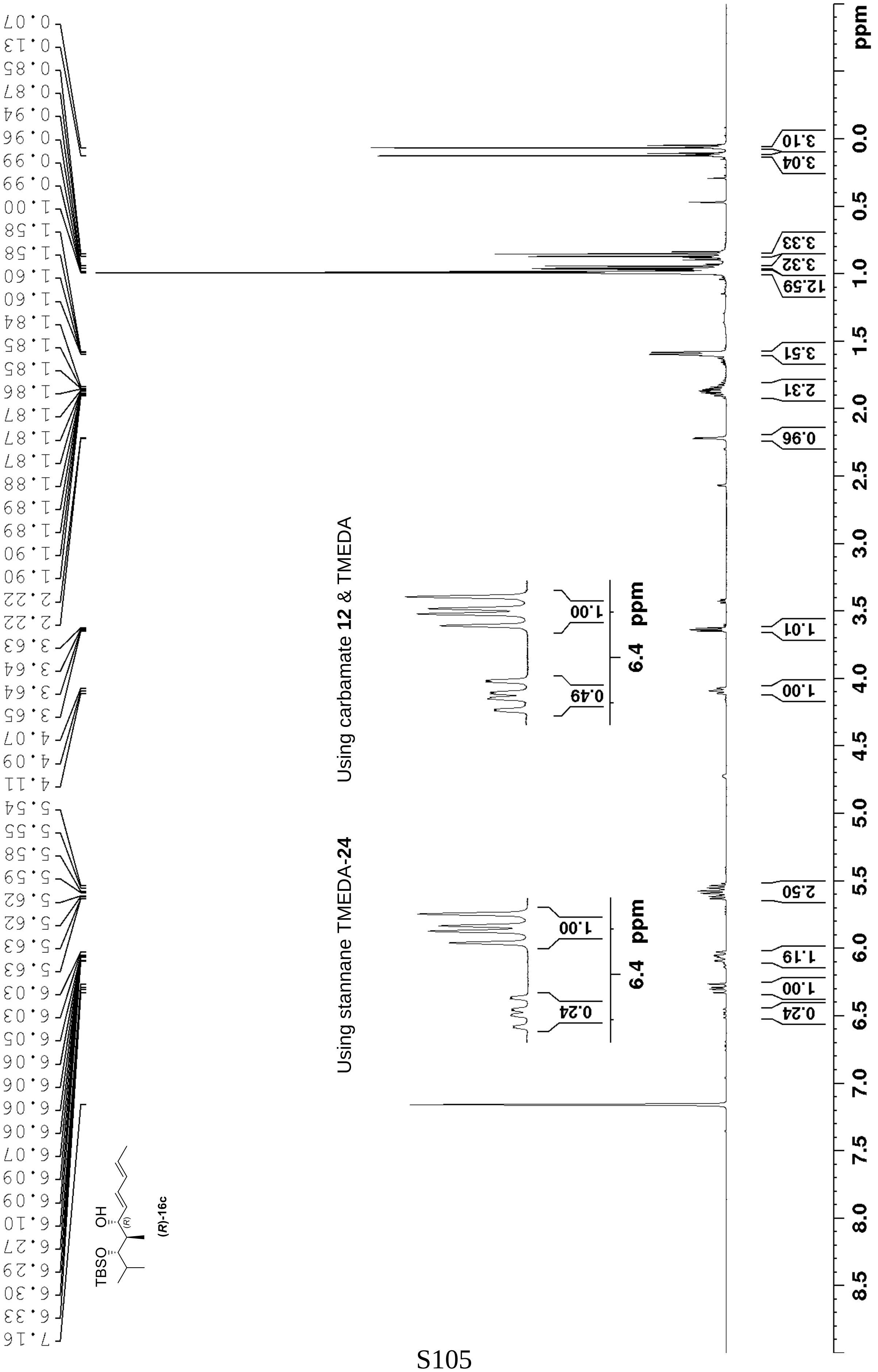
Using carbamate 12 & TMEDA
Using stannane TMEDA-24
6.4 ppm
ppm
TBSO
OH
(R)-16c
0.07 0.13 0.85 0.87 0.94 0.96 0.99 0.99 1.00 1.58 1.58 1.60 1.60 1.84 1.85 1.85 1.86 1.87 1.87 1.87 1.88 1.89 1.89 1.90 1.90 2.22 2.22 3.63 3.64 3.64 3.65 4.07 4.09 4.11 5.54 5.55 5.58 5.59 5.62 5.62 5.63 5.63 6.03 6.03 6.05 6.06 6.06 6.06 6.06 6.07 6.09 6.09 6.10 6.27 6.29 6.30 6.33 7.16

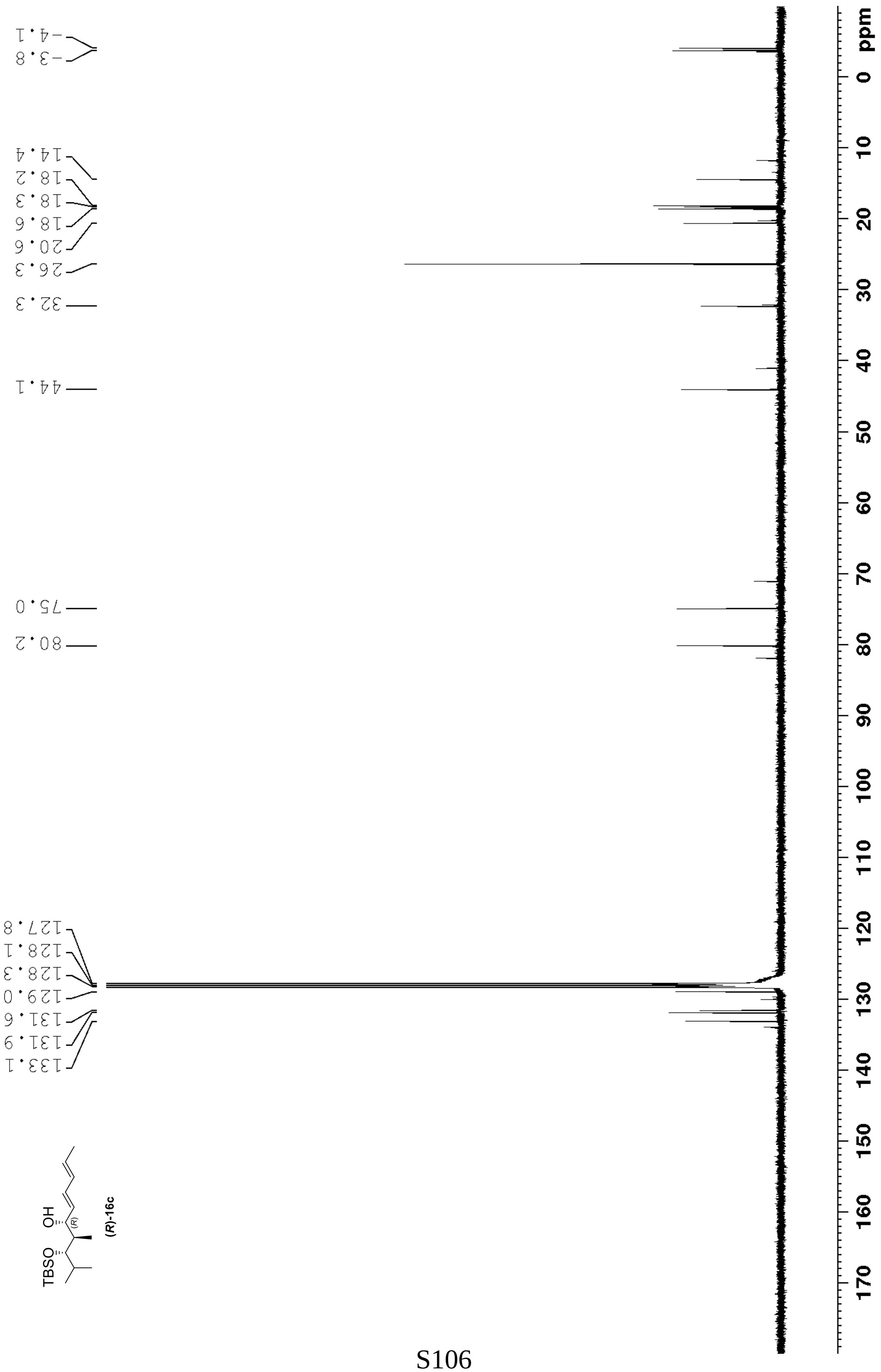
-4.1
-3.8
14.4
18.2
18.3
18.6
20.6
26.3
32.3
44.1
75.0
80.2
127.8
128.1
128.3
129.0
131.6
131.9
133.1
TBSO
OH
(R)
(R)-16c
ppm

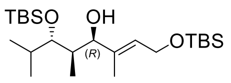
TBSO
OH
(R)
OTBS

(*R*)-**15b**

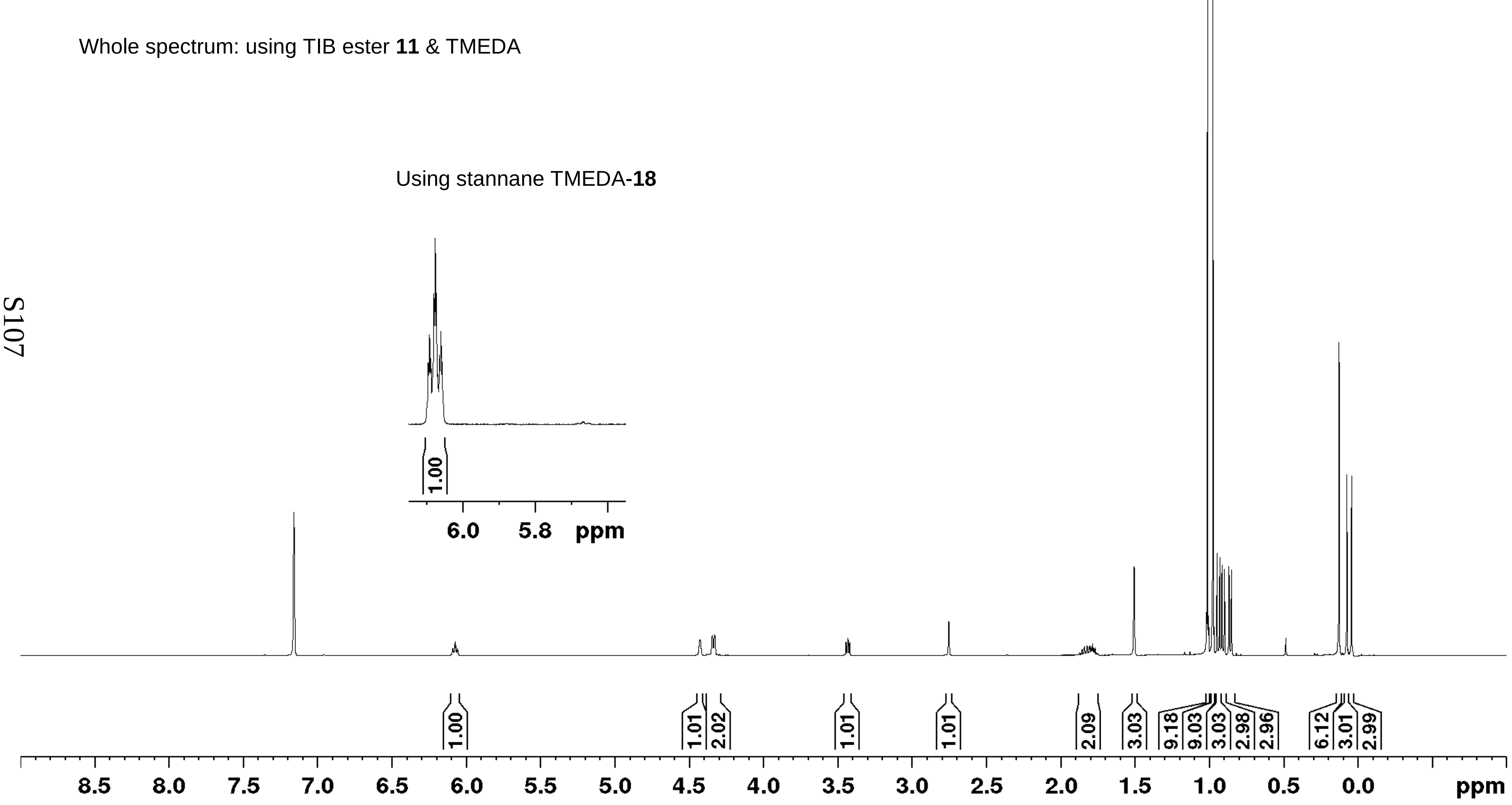
7.16
6.09
6.09
6.09
6.08
6.08
6.07
6.06
6.06
6.06
4.43
4.38
4.36
4.35
4.34
4.33
4.31
4.29
3.44
3.43
3.42
2.75
2.75
1.87
1.86
1.84
1.84
1.82
1.82
1.81
1.81
1.80
1.80
1.79
1.78
1.78
1.77
1.77
1.76
1.76
1.75
1.51
1.01
0.98
0.95
0.93
0.91
0.90
0.87
Whole spectrum: using TIB ester 11 & TMEDA
Using stannane TMEDA-18
1.00
6.0
5.8
ppm
1.00
1.01
2.02
1.01
1.01
2.09
3.03
9.18
9.03
3.03
2.98
2.96
6.12
3.01
2.99
8.5
8.0
7.5
7.0
6.5
6.0
5.5
5.0
4.5
4.0
3.5
3.0
2.5
2.0
1.5
1.0
0.5
0.0
ppm

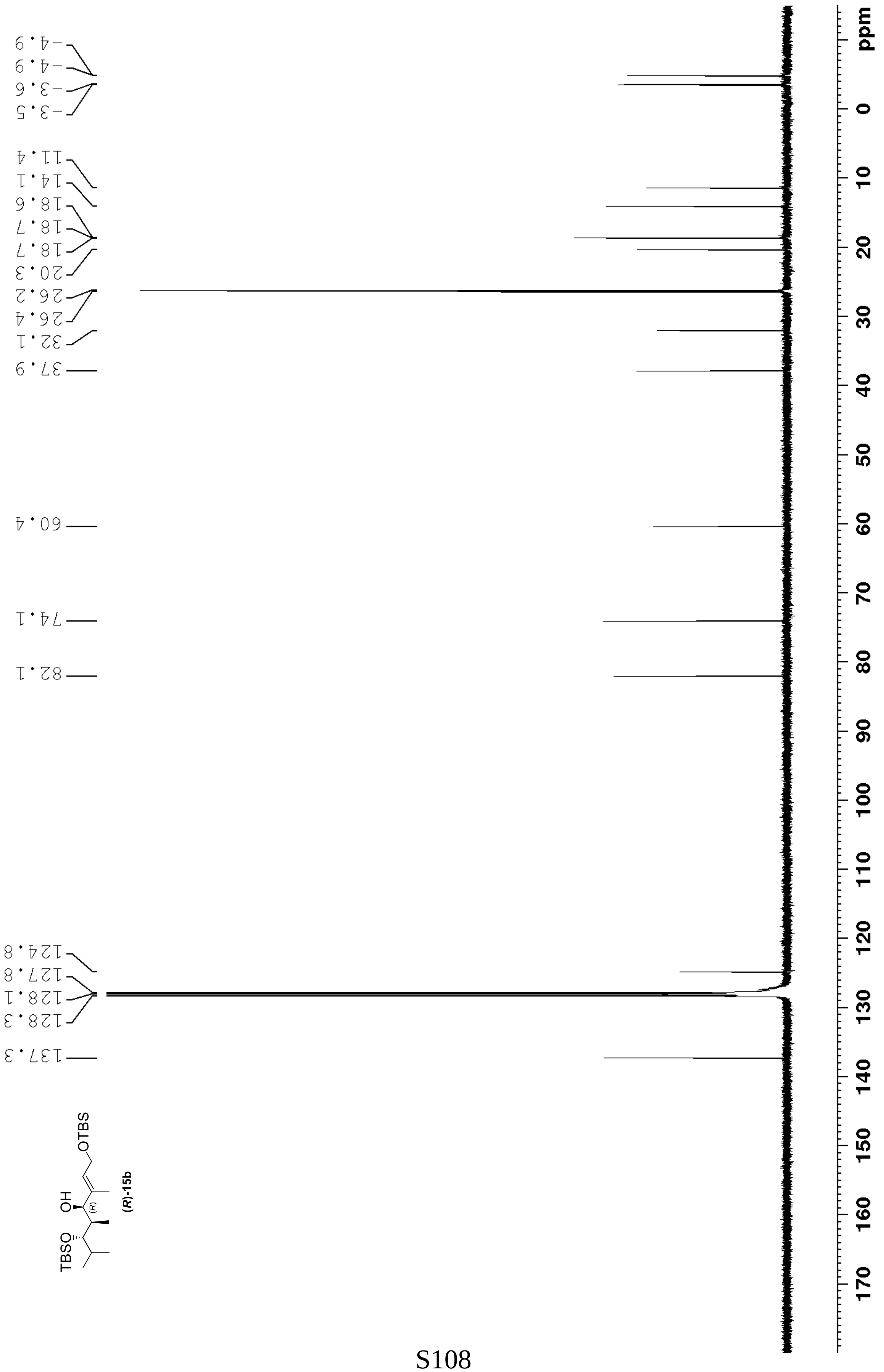

-4.9
-4.9
-3.6
-3.5
11.4
14.1
18.6
18.7
18.7
20.3
26.2
26.4
32.1
37.9
60.4
74.1
82.1
124.8
127.8
128.1
128.3
137.3
TBSO
OH
(R)
OTBS
(R)-15b
ppm

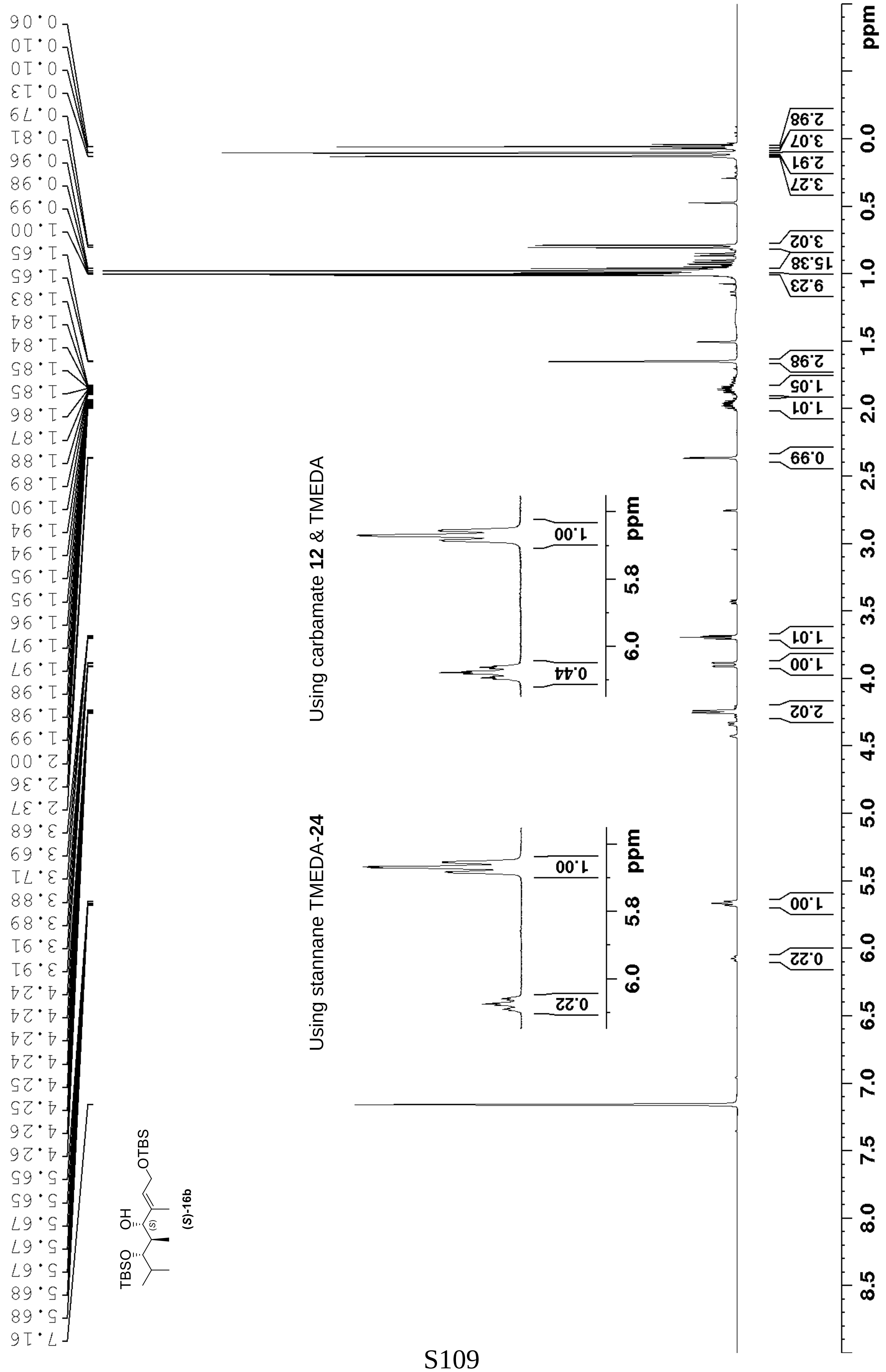

TBSO OH OTBS
(S)
(S)-16b
Using stannane TMEDA-24
Using carbamate 12 & TMEDA
1.00
0.22
1.00
0.44
6.0 5.8 ppm
0.06 0.10 0.10 0.13 0.79 0.81 0.96 0.98 0.99 1.00 1.65 1.83 1.84 1.84 1.85 1.85 1.86 1.87 1.88 1.89 1.90 1.94 1.94 1.95 1.95 1.96 1.97 1.97 1.98 1.98 1.99 2.00 2.36 2.37 3.68 3.69 3.71 3.88 3.89 3.91 3.91 4.24 4.24 4.24 4.24 4.25 4.25 4.26 4.26 5.65 5.65 5.67 5.67 5.67 5.68 5.68 7.16
2.98 3.07 2.91 3.27 3.02 15.38 9.23 2.98 1.05 1.01 0.99 1.01 1.00 2.02 1.00 0.22
ppm 0.0 0.5 1.0 1.5 2.0 2.5 3.0 3.5 4.0 4.5 5.0 5.5 6.0 6.5 7.0 7.5 8.0 8.5

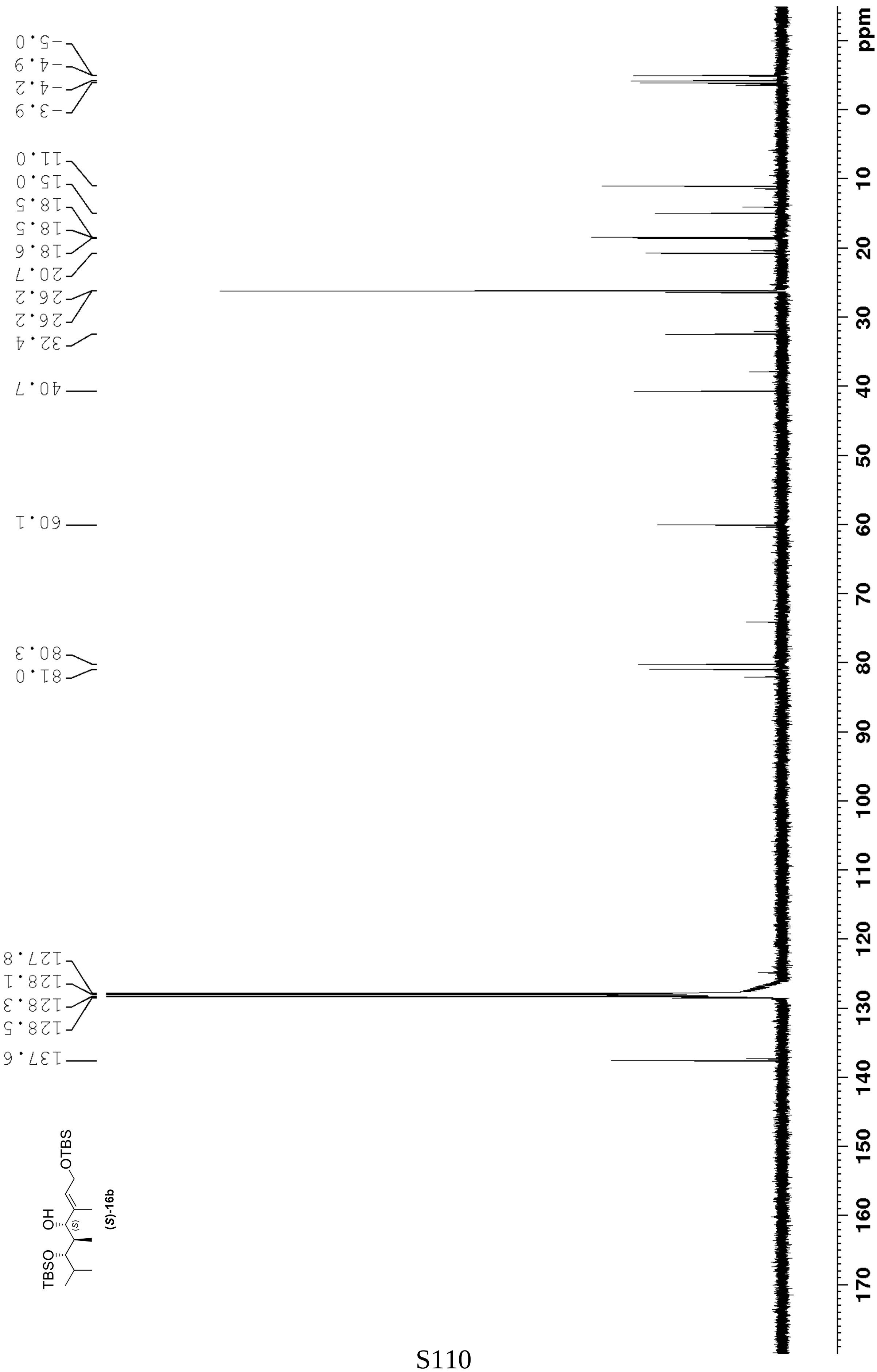
-5.0
-4.9
-4.2
-3.9
11.0
15.0
18.5
18.5
18.6
20.7
26.2
26.2
32.4
40.7
60.1
80.3
81.0
127.8
128.1
128.3
128.5
137.6
TBSO
OH
OTBS
(S)
(S)-16b
ppm

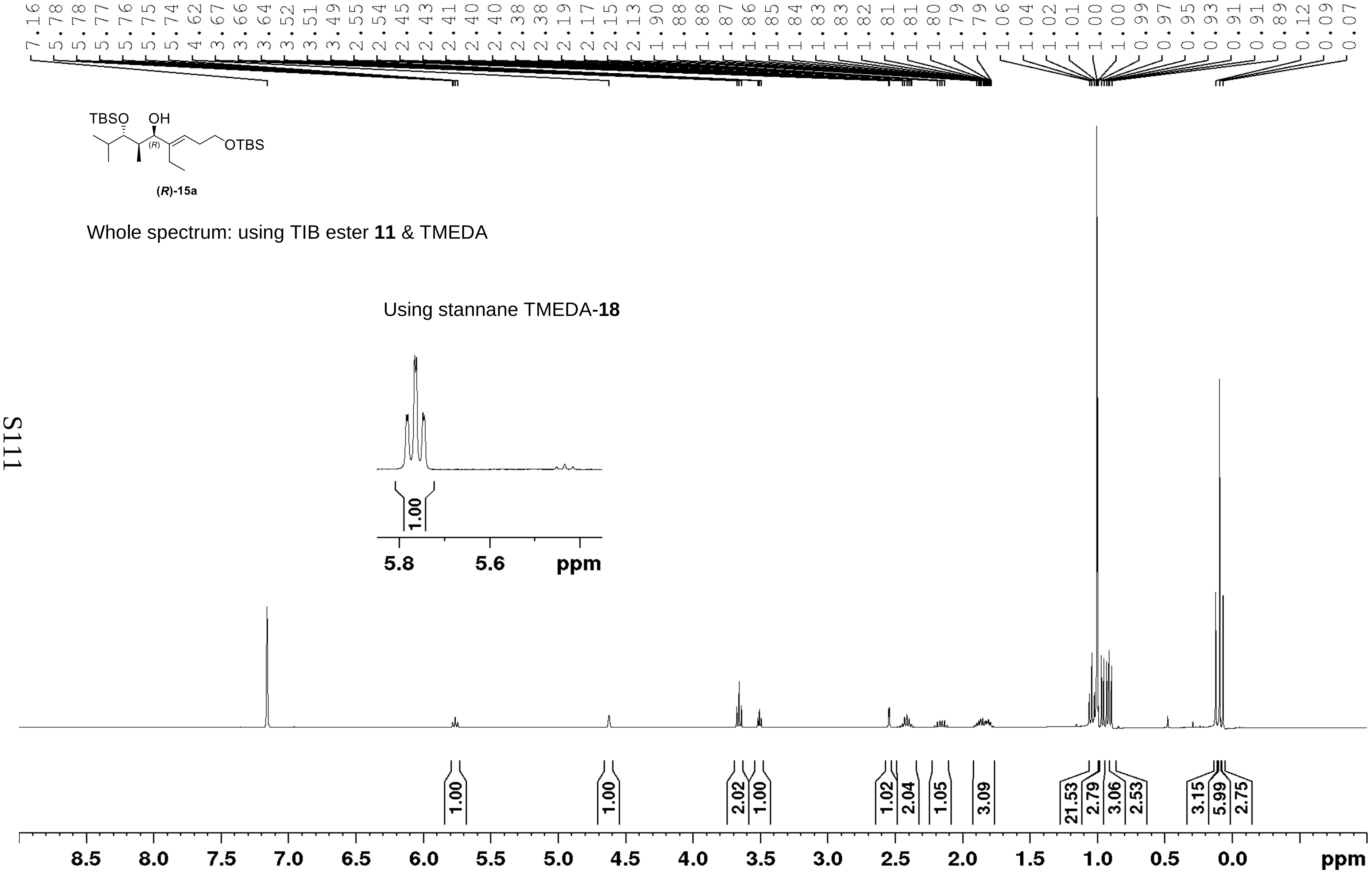
7.16 5.78 5.78 5.77 5.76 5.75 5.74 4.62 3.67 3.66 3.64 3.52 3.51 3.49 2.55 2.54 2.45 2.43 2.41 2.40 2.40 2.38 2.38 2.19 2.17 2.15 2.13 1.90 1.88 1.88 1.87 1.86 1.85 1.84 1.83 1.83 1.82 1.81 1.81 1.80 1.79 1.79 1.06 1.04 1.02 1.01 1.00 1.00 0.99 0.97 0.95 0.93 0.91 0.91 0.89 0.12 0.09 0.07
TBSO
OH
(R)
OTBS
(R)-15a
Whole spectrum: using TIB ester 11 & TMEDA
Using stannane TMEDA-18
1.00
5.8
5.6
ppm
1.00
1.00
2.02
1.00
1.02
2.04
1.05
3.09
21.53
2.79
3.06
2.53
3.15
5.99
2.75
8.5
8.0
7.5
7.0
6.5
6.0
5.5
5.0
4.5
4.0
3.5
3.0
2.5
2.0
1.5
1.0
0.5
0.0
ppm

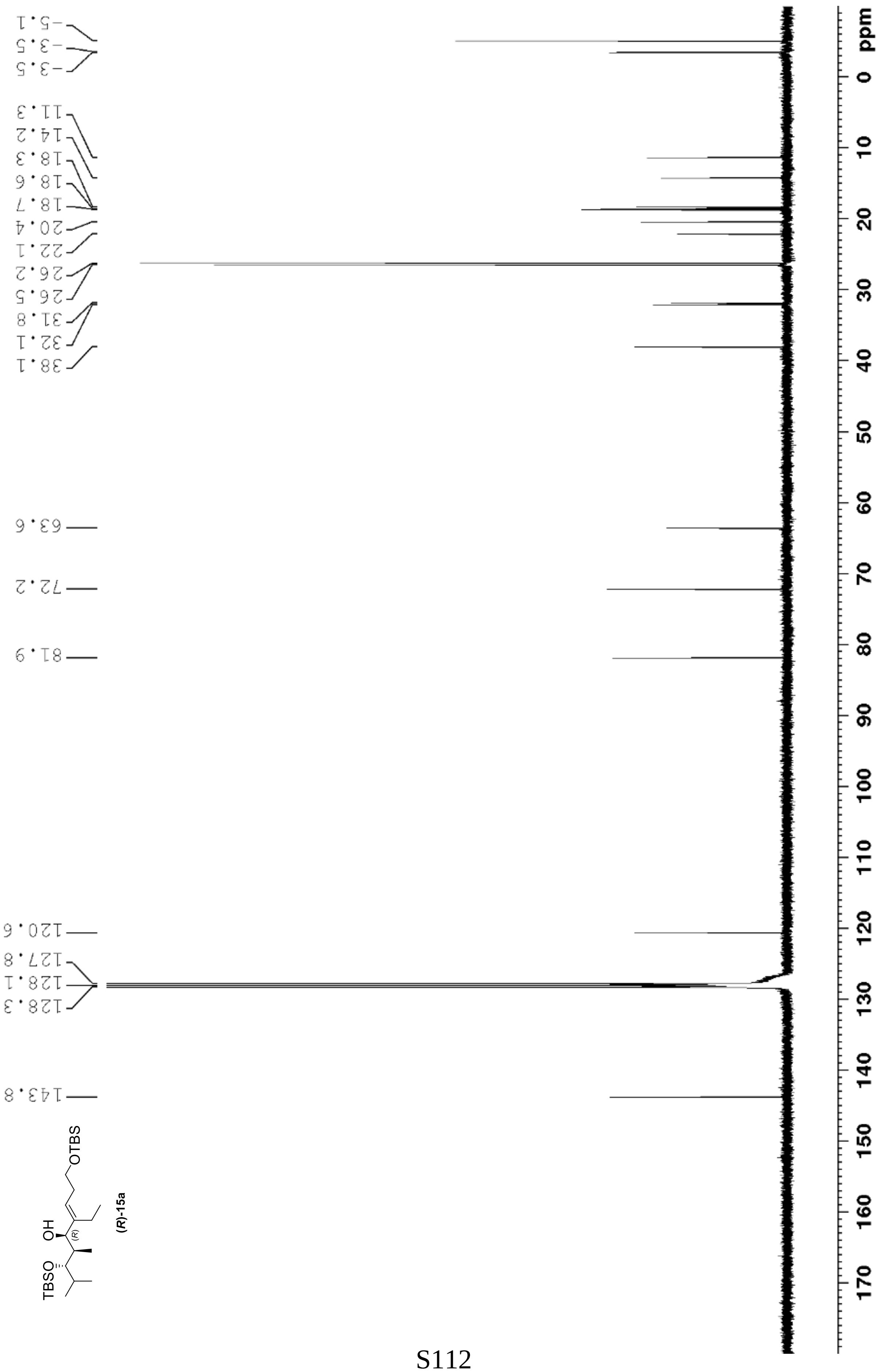
-5.1
-3.5
-3.5
11.3
14.2
18.3
18.6
18.7
20.4
22.1
26.2
26.5
31.8
32.1
38.1
63.6
72.2
81.9
120.6
127.8
128.1
128.3
143.8
TBSO
OH
(R)
OTBS
(R)-15a
ppm
0
10
20
30
40
50
60
70
80
90
100
110
120
130
140
150
160
170

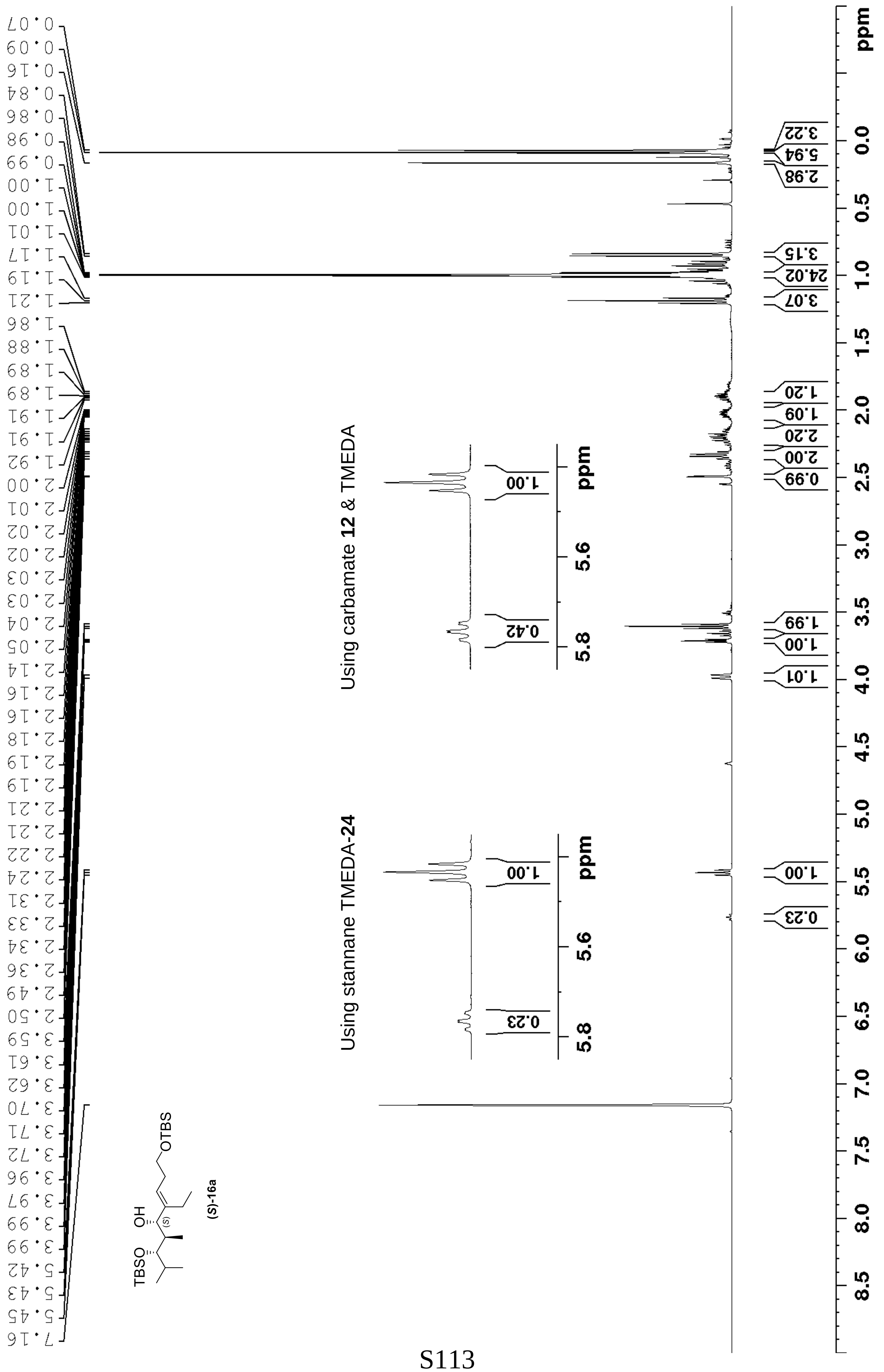
Using carbamate 12 & TMEDA
Using stannane TMEDA-24
TBSO
OH
OTBS
(S)
(S)-16a
ppm

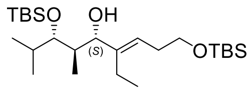
TBSO
OH
(S)
OTBS

(*S*)-16a

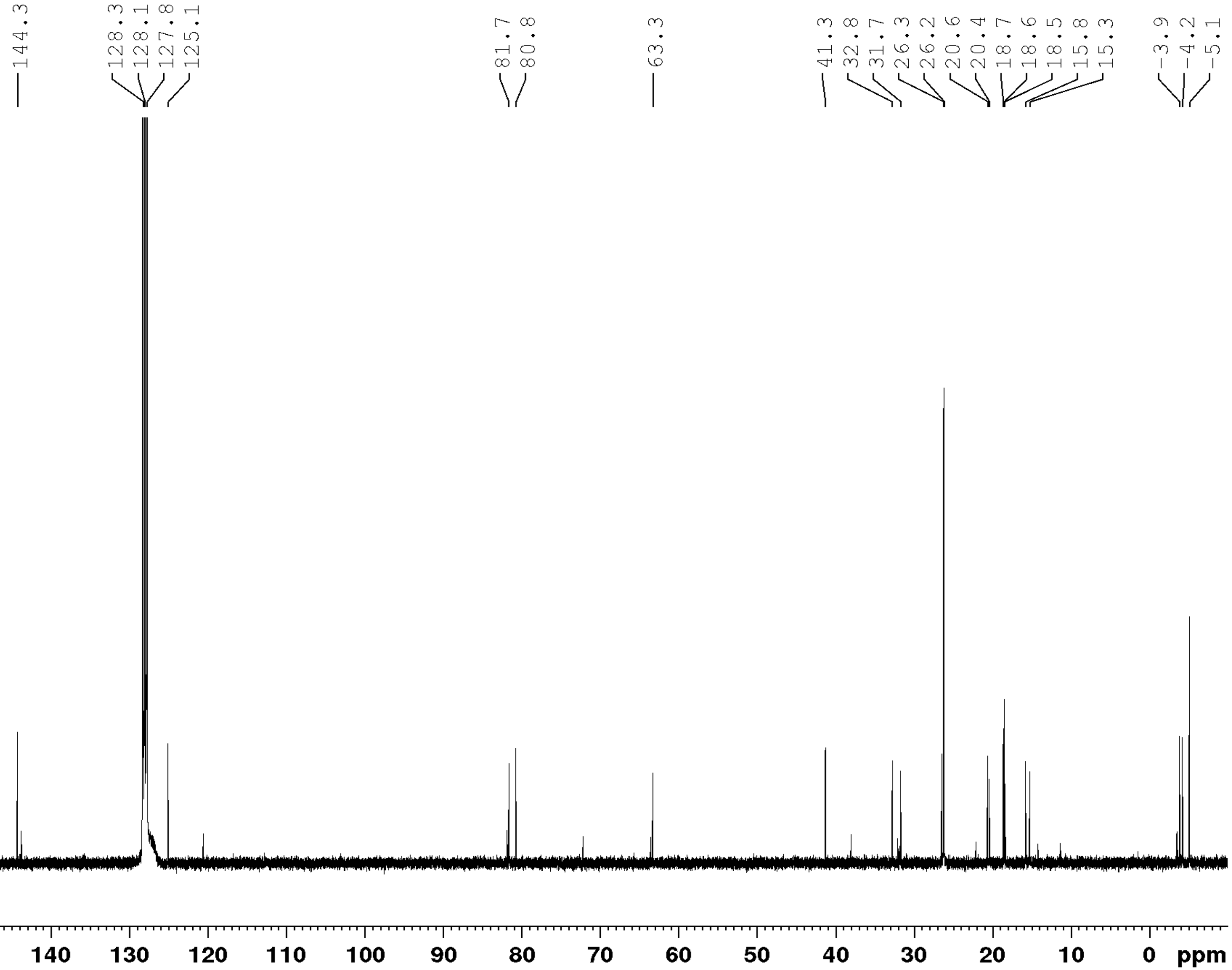
144.3
128.3
128.1
127.8
125.1
81.7
80.8
63.3
41.3
32.8
31.7
26.3
26.2
20.6
20.4
18.7
18.6
18.5
15.8
15.3
-3.9
-4.2
-5.1
170 160 150 140 130 120 110 100 90 80 70 60 50 40 30 20 10 0 ppm

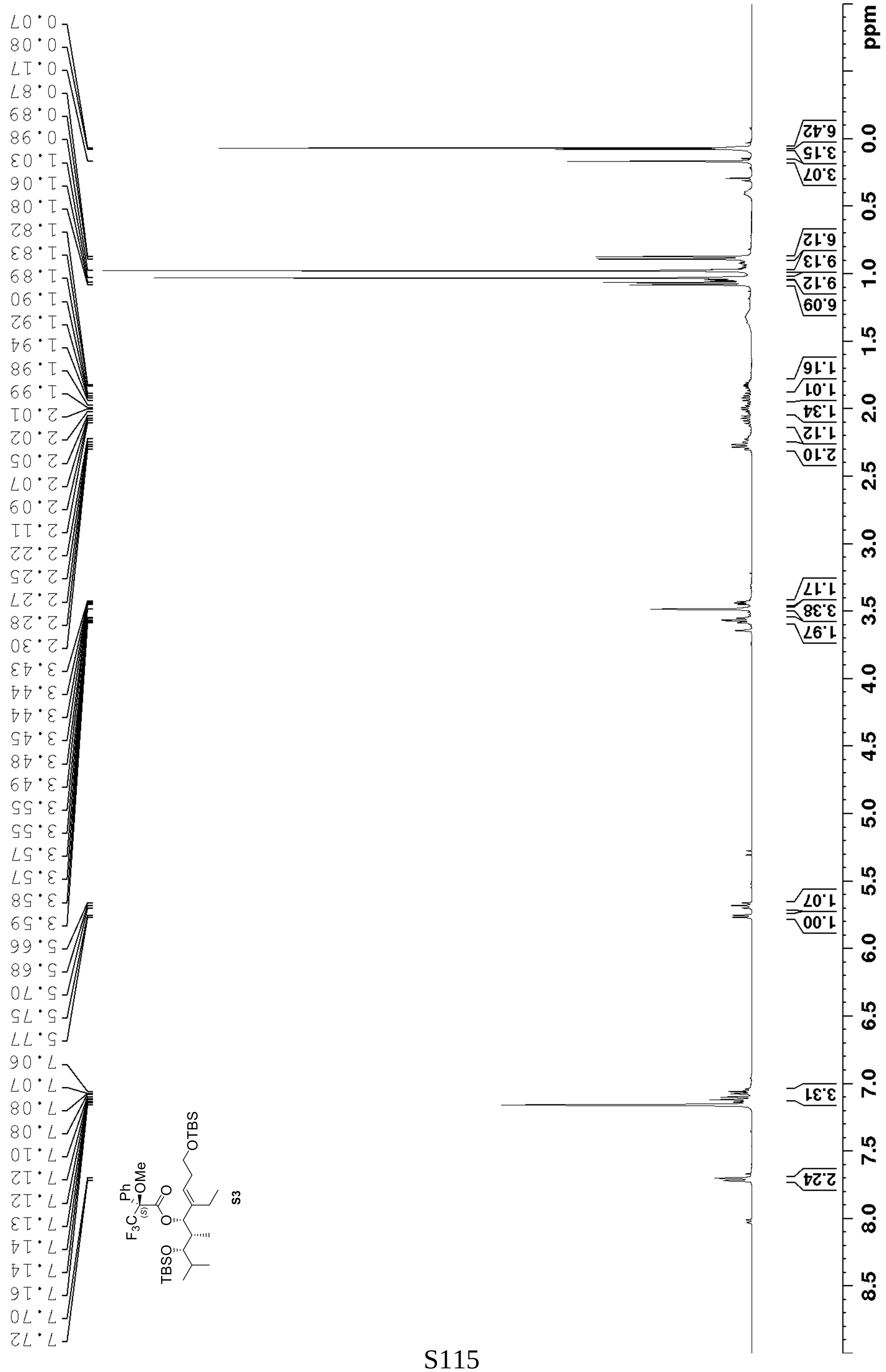
0.07
0.08
0.17
0.87
0.89
0.98
1.03
1.06
1.08
1.82
1.83
1.89
1.90
1.92
1.94
1.98
1.99
2.01
2.02
2.05
2.07
2.09
2.11
2.22
2.25
2.27
2.28
2.30
3.43
3.44
3.44
3.45
3.48
3.49
3.55
3.55
3.57
3.57
3.58
3.59
5.66
5.68
5.70
5.75
5.77
7.06
7.07
7.08
7.08
7.10
7.12
7.12
7.13
7.14
7.14
7.16
7.70
7.72
TBSO
OTBS
Ph
OMe
(S)
S3
6.42
3.15
3.07
6.12
9.13
9.12
6.09
1.16
1.01
1.34
1.12
2.10
1.17
3.38
1.97
1.07
1.00
3.31
2.24
ppm

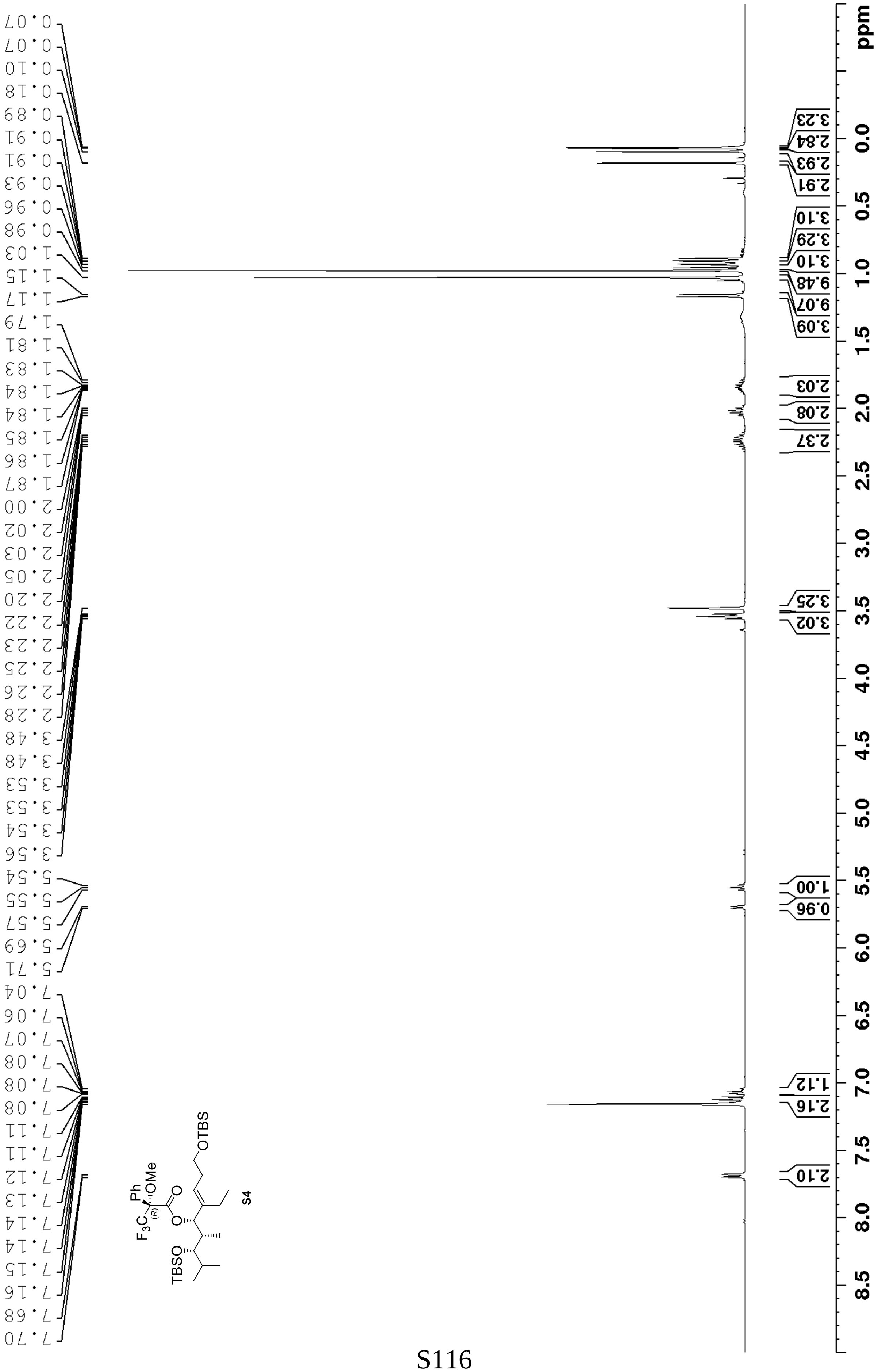

0.07
0.07
0.10
0.18
0.89
0.91
0.91
0.93
0.96
0.98
1.03
1.15
1.17
1.79
1.81
1.83
1.84
1.84
1.85
1.86
1.87
2.00
2.02
2.03
2.05
2.20
2.22
2.23
2.25
2.26
2.28
3.48
3.48
3.53
3.53
3.54
3.56
5.54
5.55
5.57
5.69
5.71
7.04
7.06
7.07
7.08
7.08
7.08
7.11
7.11
7.12
7.13
7.14
7.14
7.15
7.16
7.68
7.70
3.23
2.84
2.93
2.91
3.10
3.29
3.10
9.48
9.07
3.09
2.03
2.08
2.37
3.25
3.02
1.00
0.96
1.12
2.16
2.10
ppm
0.0
0.5
1.0
1.5
2.0
2.5
3.0
3.5
4.0
4.5
5.0
5.5
6.0
6.5
7.0
7.5
8.0
8.5
OTBS
Ph
OMe
F_3C
(R)
O
TBSO
S4

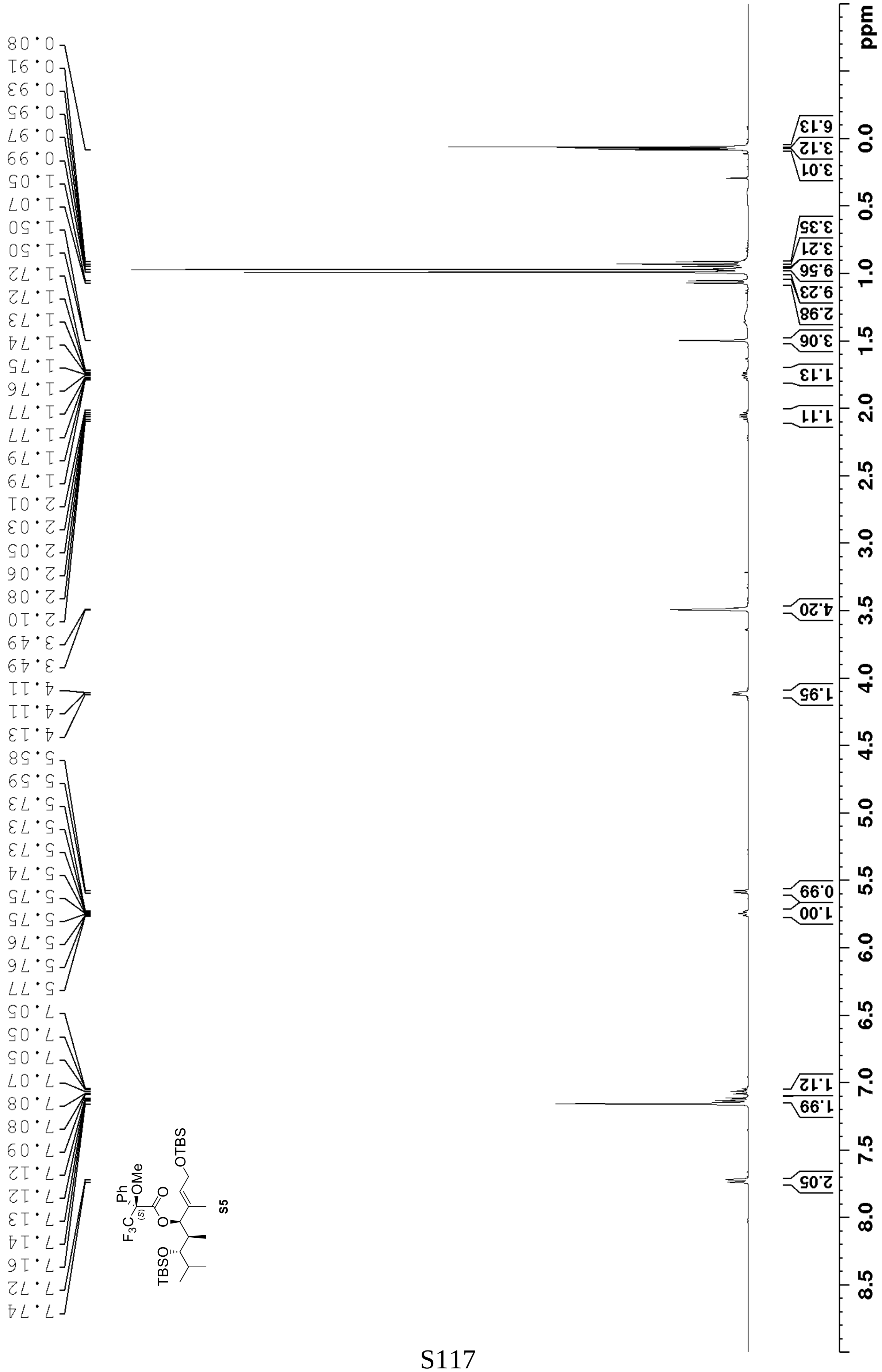
0.08
0.91
0.93
0.95
0.97
0.99
1.05
1.07
1.50
1.50
1.72
1.72
1.73
1.74
1.75
1.76
1.77
1.77
1.79
1.79
2.01
2.03
2.05
2.06
2.08
2.10
3.49
3.49
4.11
4.11
4.13
5.58
5.59
5.73
5.73
5.73
5.74
5.75
5.75
5.76
5.76
5.77
7.05
7.05
7.05
7.07
7.08
7.08
7.09
7.12
7.12
7.13
7.14
7.16
7.72
7.74
6.13
3.12
3.01
3.35
3.21
9.56
9.23
2.98
3.06
1.13
1.11
4.20
1.95
0.99
1.00
1.12
1.99
2.05
ppm
0.0
0.5
1.0
1.5
2.0
2.5
3.0
3.5
4.0
4.5
5.0
5.5
6.0
6.5
7.0
7.5
8.0
8.5
Ph
OMe
F3C
(S)
O
O
OTBS
TBSO
S5

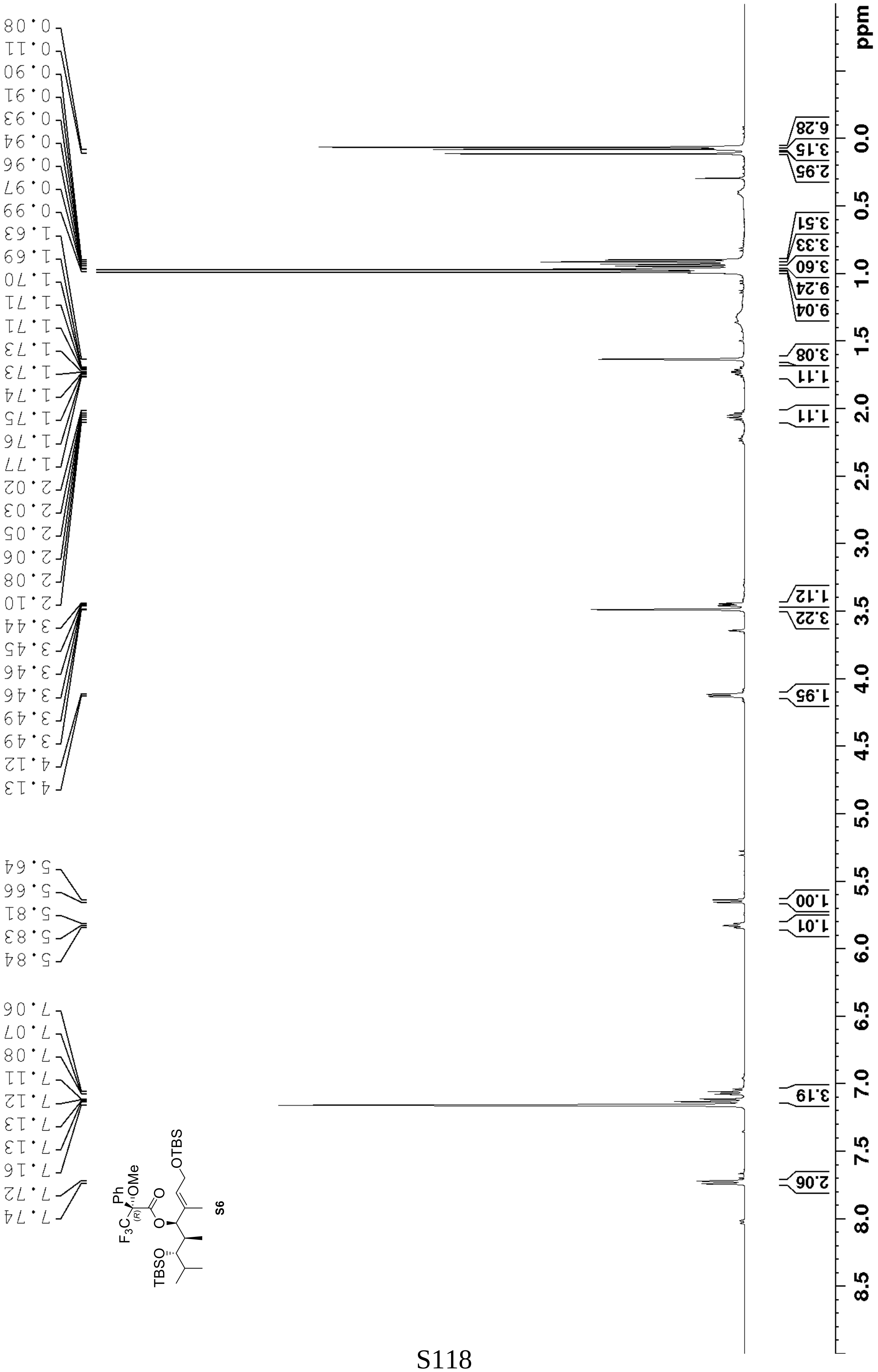
0.08
0.11
0.90
0.91
0.93
0.94
0.96
0.97
0.99
1.63
1.69
1.70
1.71
1.71
1.73
1.73
1.74
1.75
1.76
1.77
2.02
2.03
2.05
2.06
2.08
2.10
3.44
3.45
3.46
3.46
3.49
3.49
4.12
4.13
5.64
5.66
5.81
5.83
5.84
7.06
7.07
7.08
7.11
7.12
7.13
7.13
7.16
7.72
7.74
TBSO
F_3C
(R)
Ph
OMe
OTBS
S6
6.28
3.15
2.95
3.51
3.33
3.60
9.24
9.04
3.08
1.11
1.11
1.12
3.22
1.95
1.00
1.01
3.19
2.06
ppm
0.0
0.5
1.0
1.5
2.0
2.5
3.0
3.5
4.0
4.5
5.0
5.5
6.0
6.5
7.0
7.5
8.0
8.5

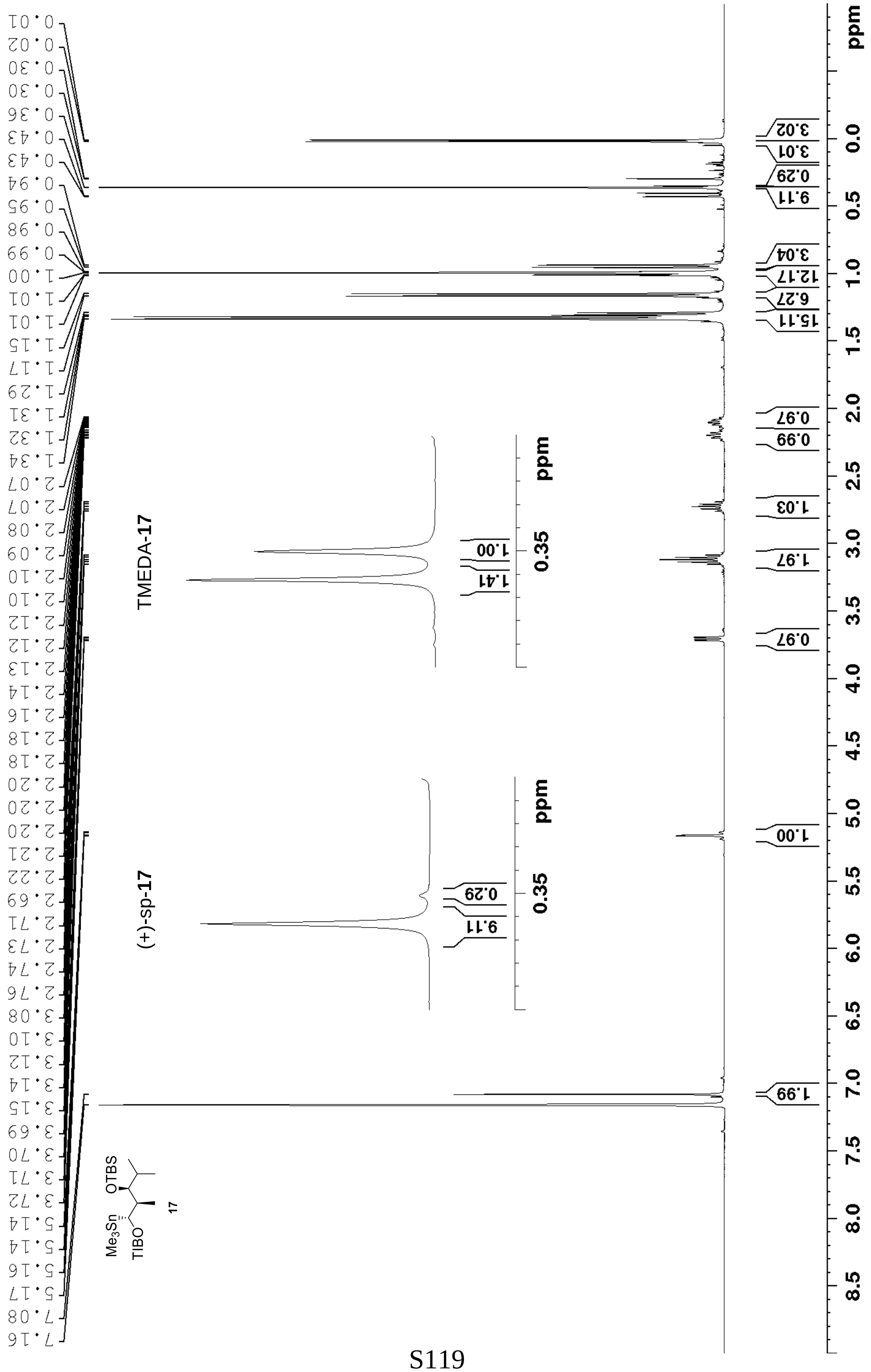
TMEDA-17
(+)-sp-17
ppm
0.35
1.00
1.41
0.29
9.11
Me3Sn
TIBO
OTBS
17

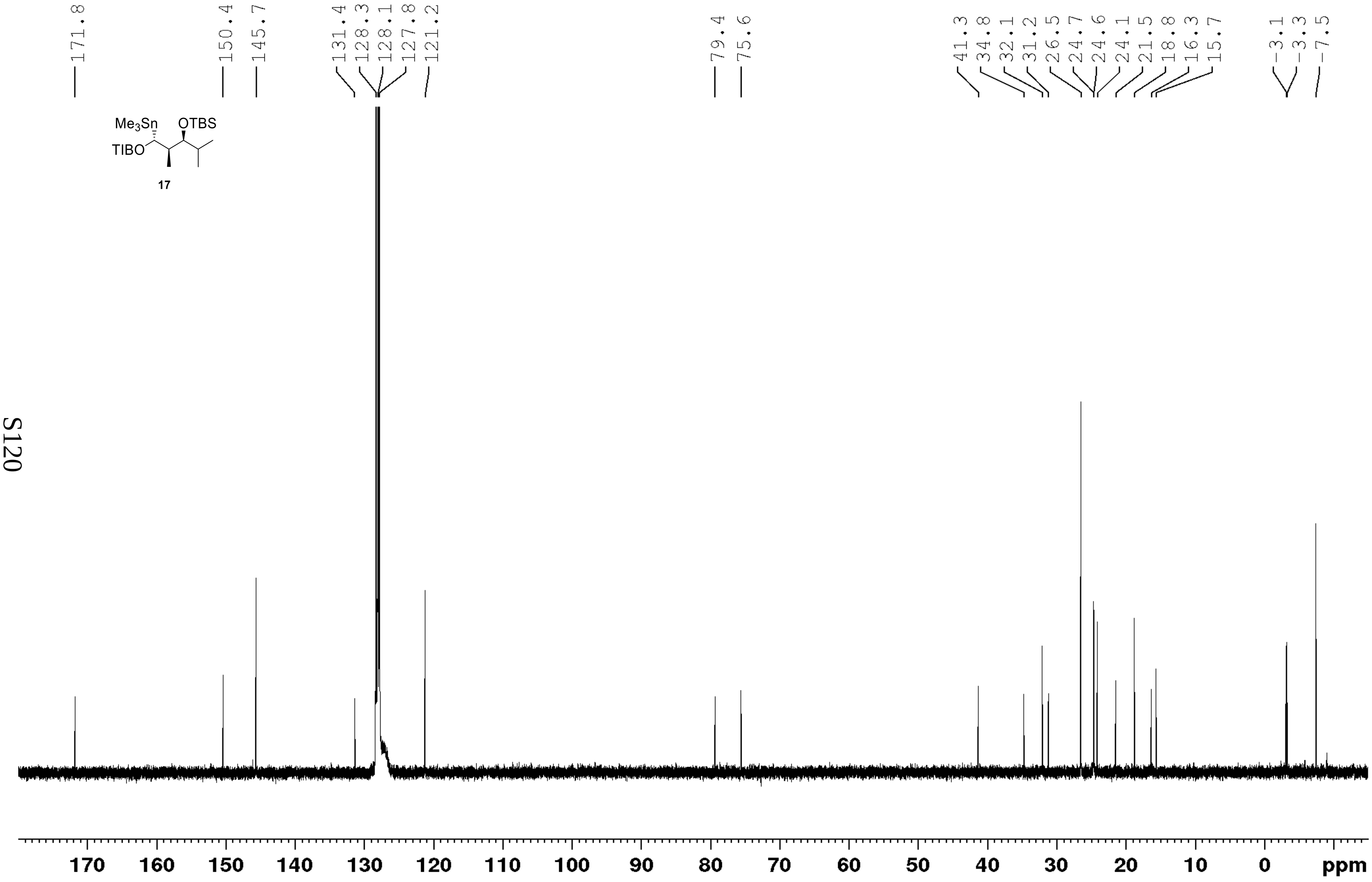
171.8
150.4
145.7
131.4
128.3
128.1
127.8
121.2
79.4
75.6
41.3
34.8
32.1
31.2
26.5
24.7
24.6
24.1
21.5
18.8
16.3
15.7
-3.1
-3.3
-7.5
Me3Sn
OTBS
TIBO
17
170
160
150
140
130
120
110
100
90
80
70
60
50
40
30
20
10
0
ppm

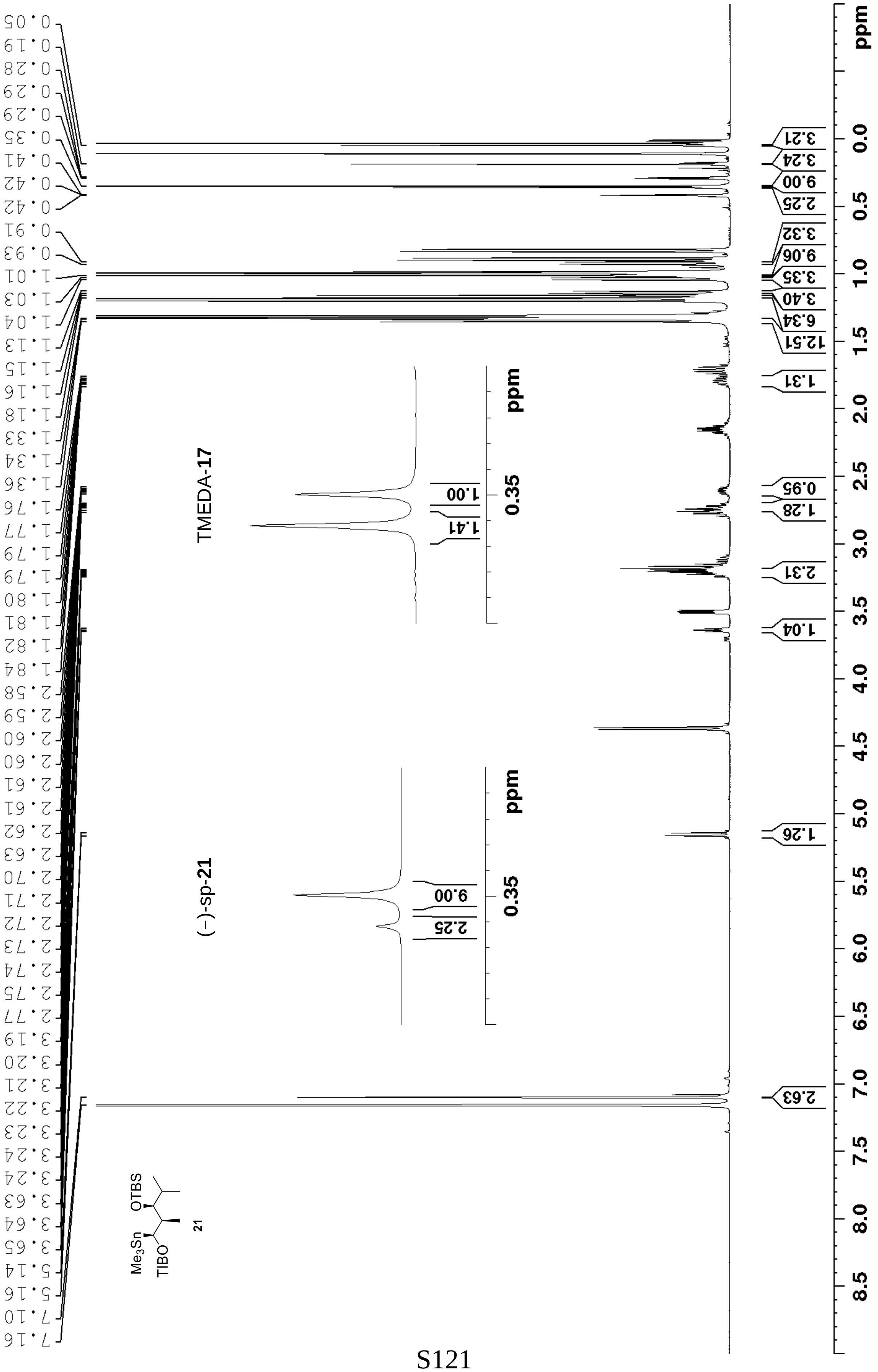

TMEDA-17
(–)-sp-21
Me3Sn
OTBS
TIBO
21
ppm

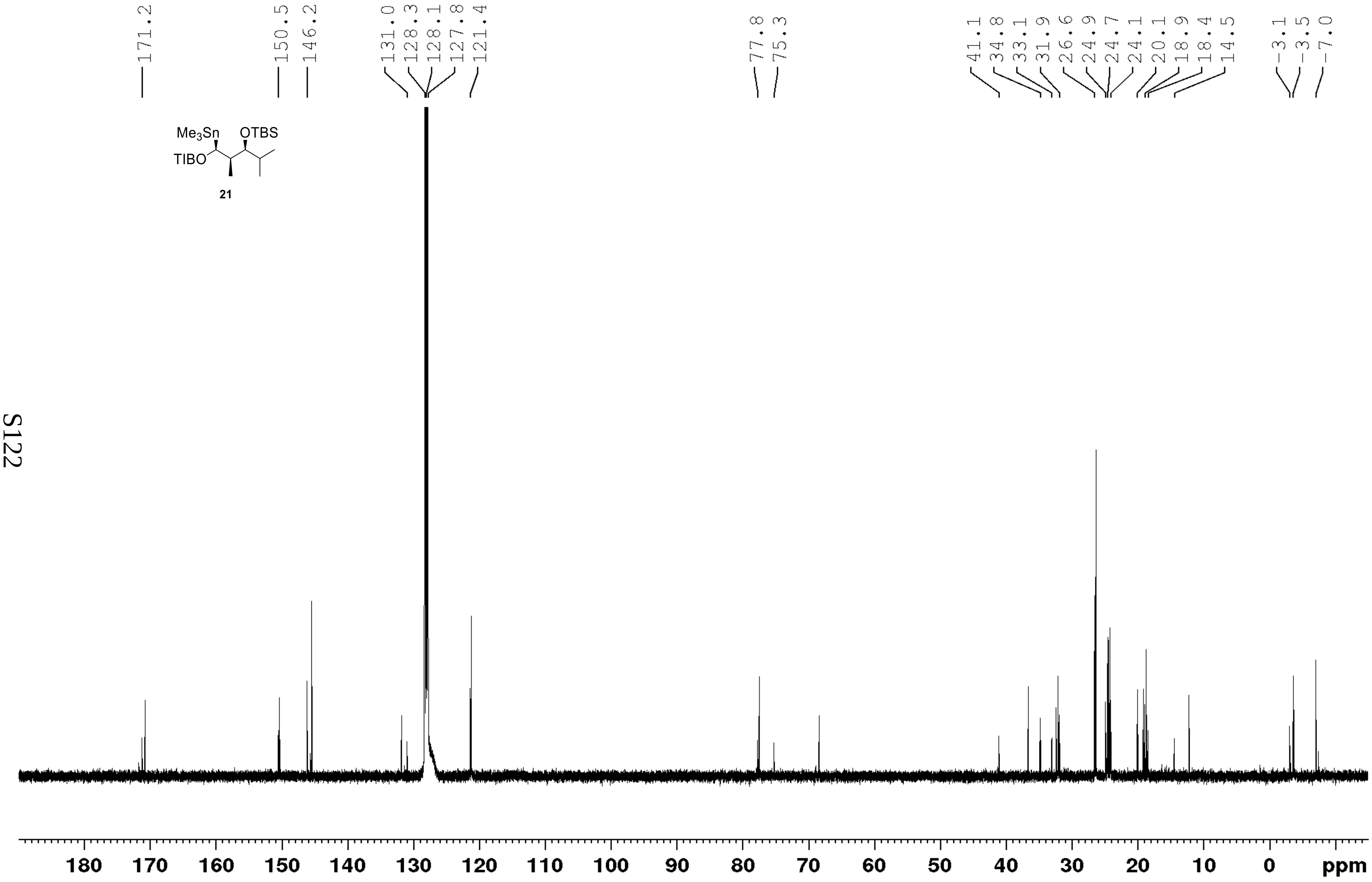
171.2
150.5
146.2
131.0
128.3
128.1
127.8
121.4
77.8
75.3
41.1
34.8
33.1
31.9
26.6
24.9
24.7
24.1
20.1
18.9
18.4
14.5
-3.1
-3.5
-7.0
Me3Sn
OTBS
TIBO
21
180
170
160
150
140
130
120
110
100
90
80
70
60
50
40
30
20
10
0
ppm

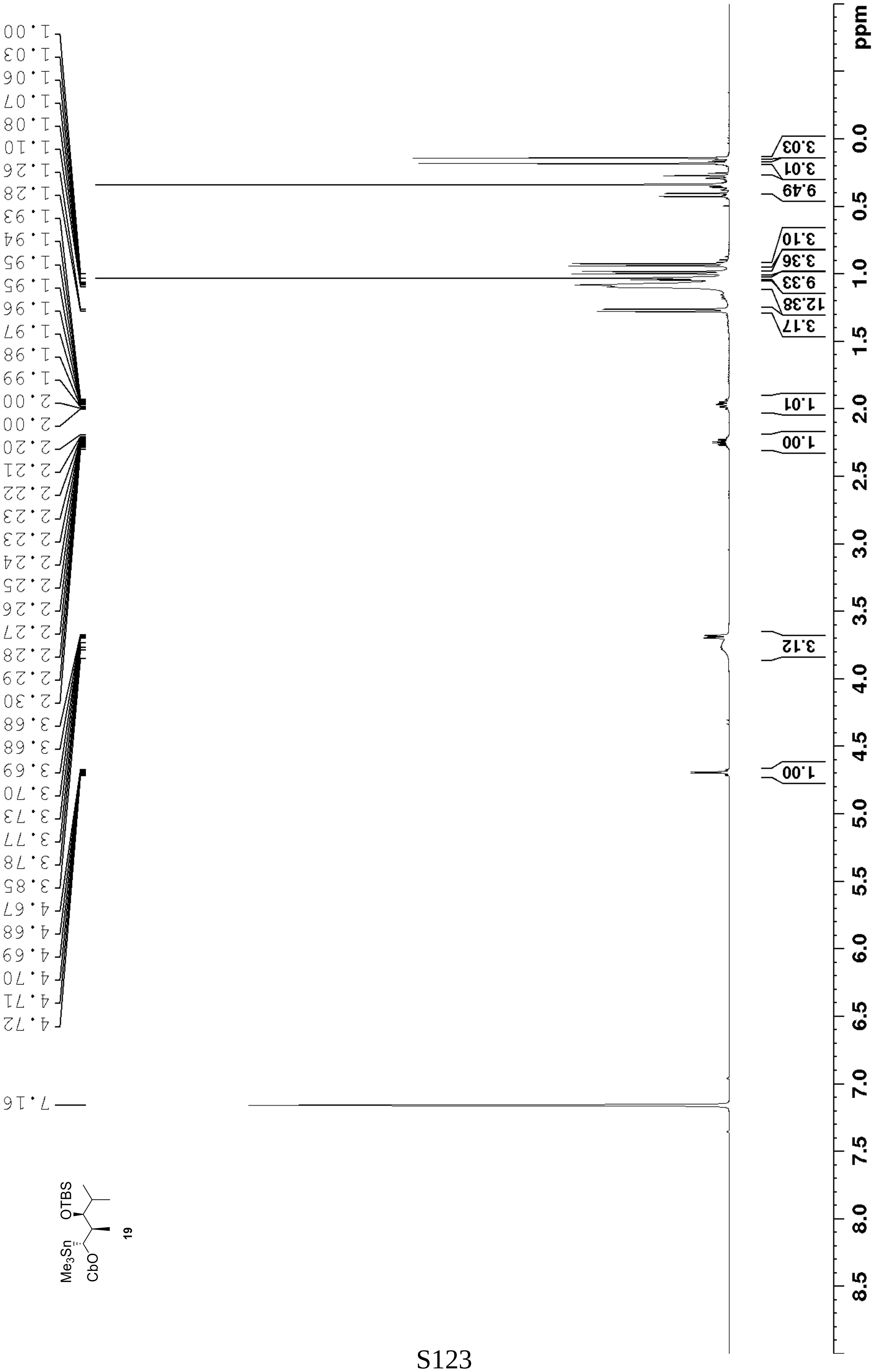
1.00
1.03
1.06
1.07
1.08
1.10
1.26
1.28
1.93
1.94
1.95
1.95
1.96
1.97
1.98
1.99
2.00
2.00
2.20
2.21
2.22
2.23
2.23
2.24
2.25
2.26
2.27
2.28
2.29
2.30
3.68
3.68
3.69
3.70
3.73
3.77
3.78
3.85
4.67
4.68
4.69
4.70
4.71
4.72
7.16
Me3Sn
OTBS
CbO
19
3.03
3.01
9.49
3.10
3.36
9.33
12.38
3.17
1.01
1.00
3.12
1.00
ppm
0.0
0.5
1.0
1.5
2.0
2.5
3.0
3.5
4.0
4.5
5.0
5.5
6.0
6.5
7.0
7.5
8.0
8.5

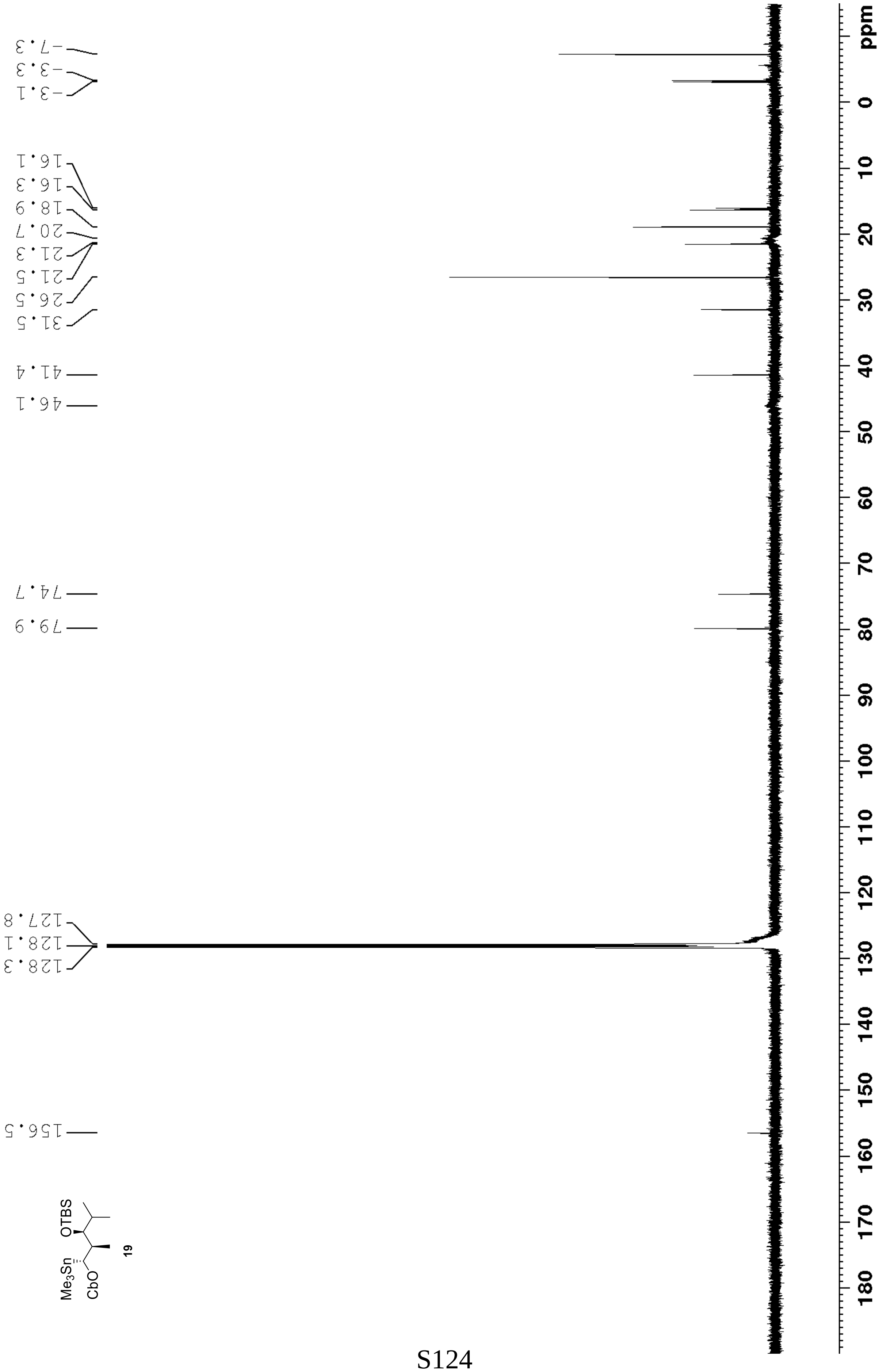
-7.3
-3.3
-3.1
16.1
16.3
18.9
20.7
21.3
21.5
26.5
31.5
41.4
46.1
74.7
79.9
127.8
128.1
128.3
156.5
Me3Sn
CbO
OTBS
19
ppm

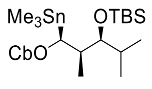

23

Whole spectrum: (−)-sp-**23**

TMEDA-**23**

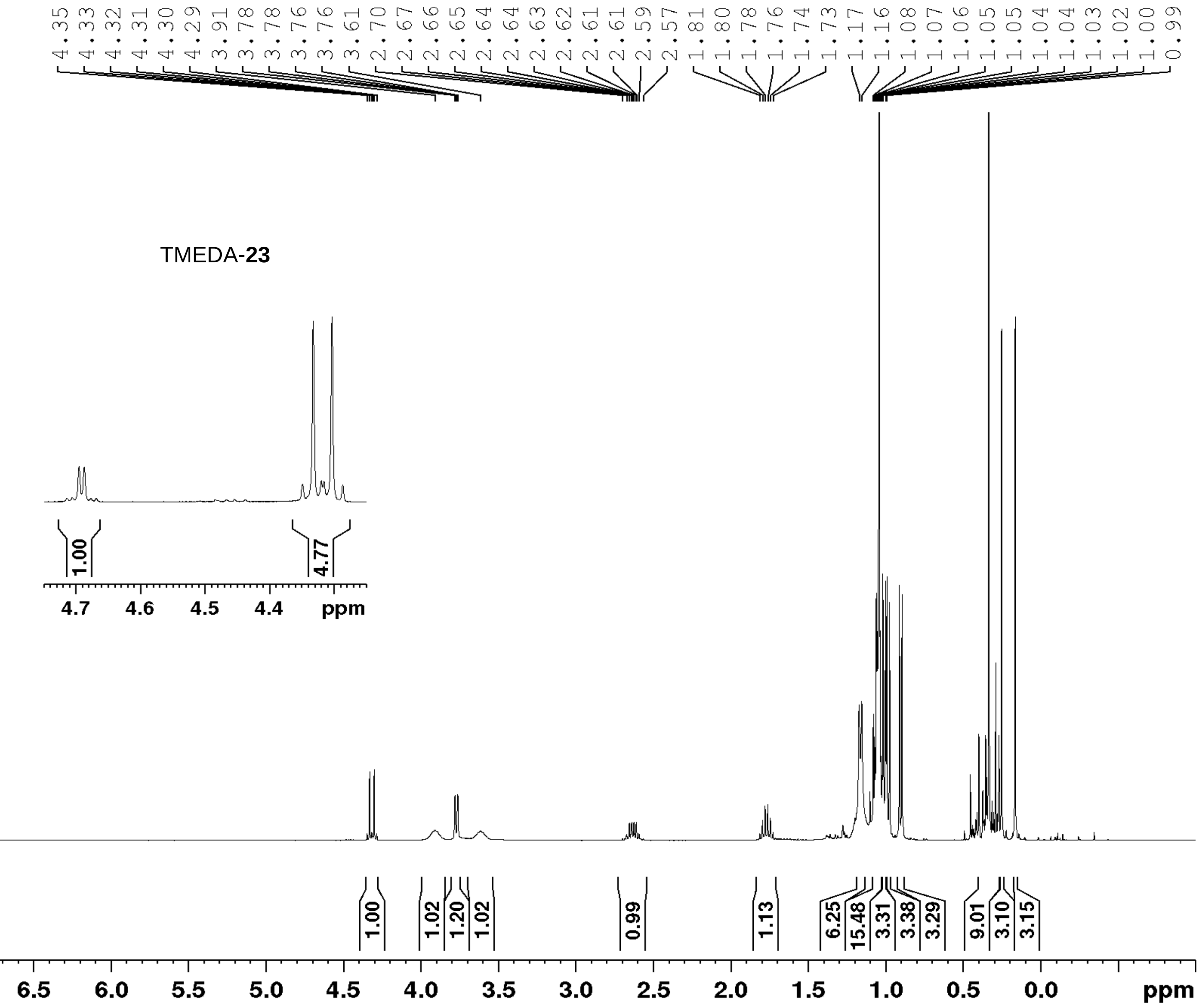

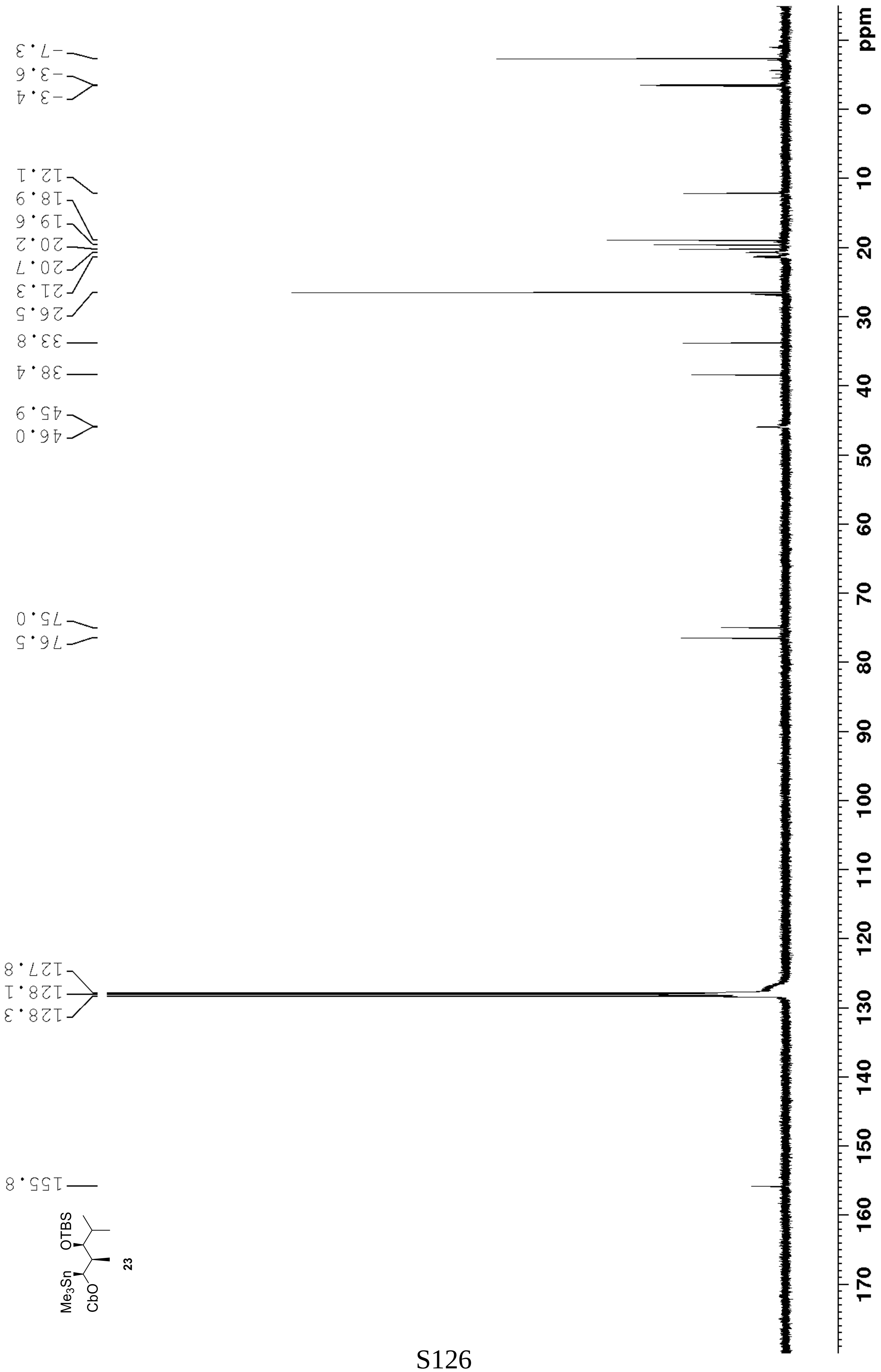
-7.3
-3.6
-3.4
12.1
18.9
19.6
20.2
20.7
21.3
26.5
33.8
38.4
45.9
46.0
75.0
76.5
127.8
128.1
128.3
155.8
Me3Sn
OTBS
CbO
23
ppm

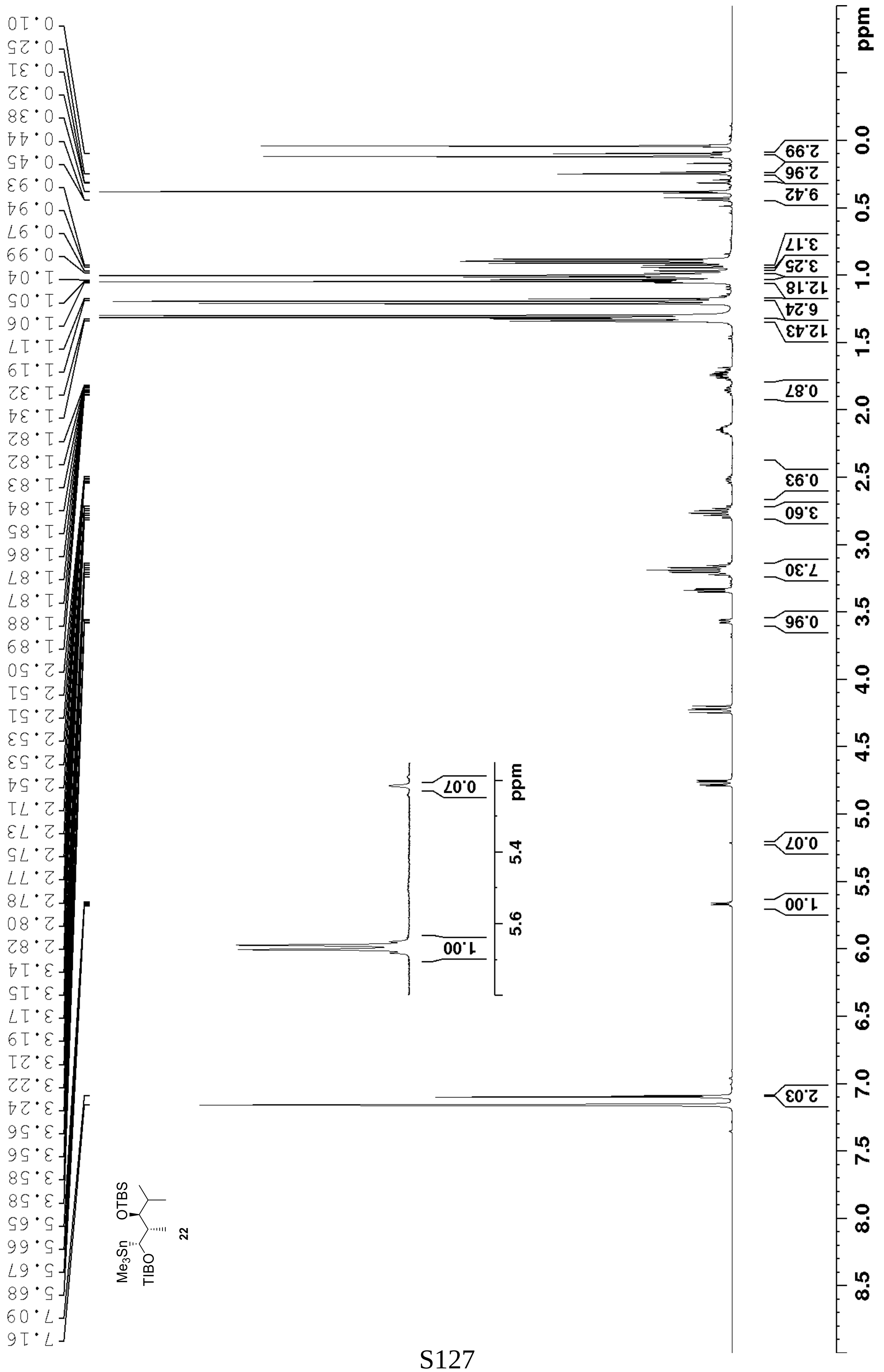
0.10
0.25
0.31
0.32
0.38
0.44
0.45
0.93
0.94
0.97
0.99
1.04
1.05
1.06
1.17
1.19
1.32
1.34
1.82
1.82
1.83
1.84
1.85
1.86
1.87
1.87
1.88
1.89
2.50
2.51
2.51
2.53
2.53
2.54
2.71
2.73
2.75
2.77
2.78
2.80
2.82
3.14
3.15
3.17
3.19
3.21
3.22
3.24
3.56
3.56
3.58
3.58
5.65
5.66
5.67
5.68
7.09
7.16
Me_3Sn
OTBS
TIBO
22
0.07
1.00
ppm
5.4
5.6
2.99
2.96
9.42
3.17
3.25
12.18
6.24
12.43
0.87
0.93
3.60
7.30
0.96
0.07
1.00
2.03
ppm

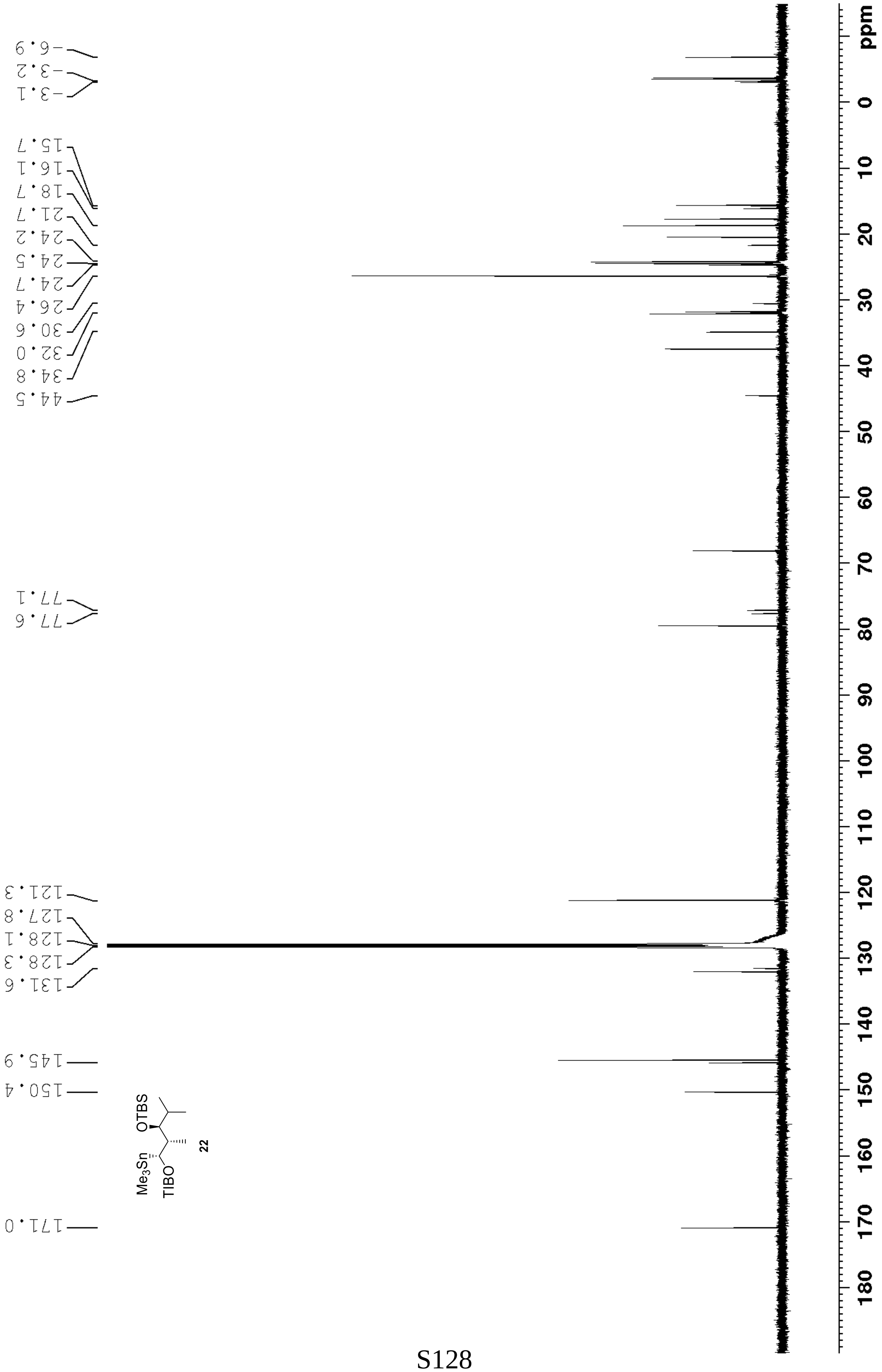
ppm
-6.9
-3.2
-3.1
15.7
16.1
18.7
21.7
24.2
24.5
24.7
26.4
30.6
32.0
34.8
44.5
77.1
77.6
121.3
127.8
128.1
128.3
131.6
145.9
150.4
171.0
OTBS
Me3Sn
TIBO
22

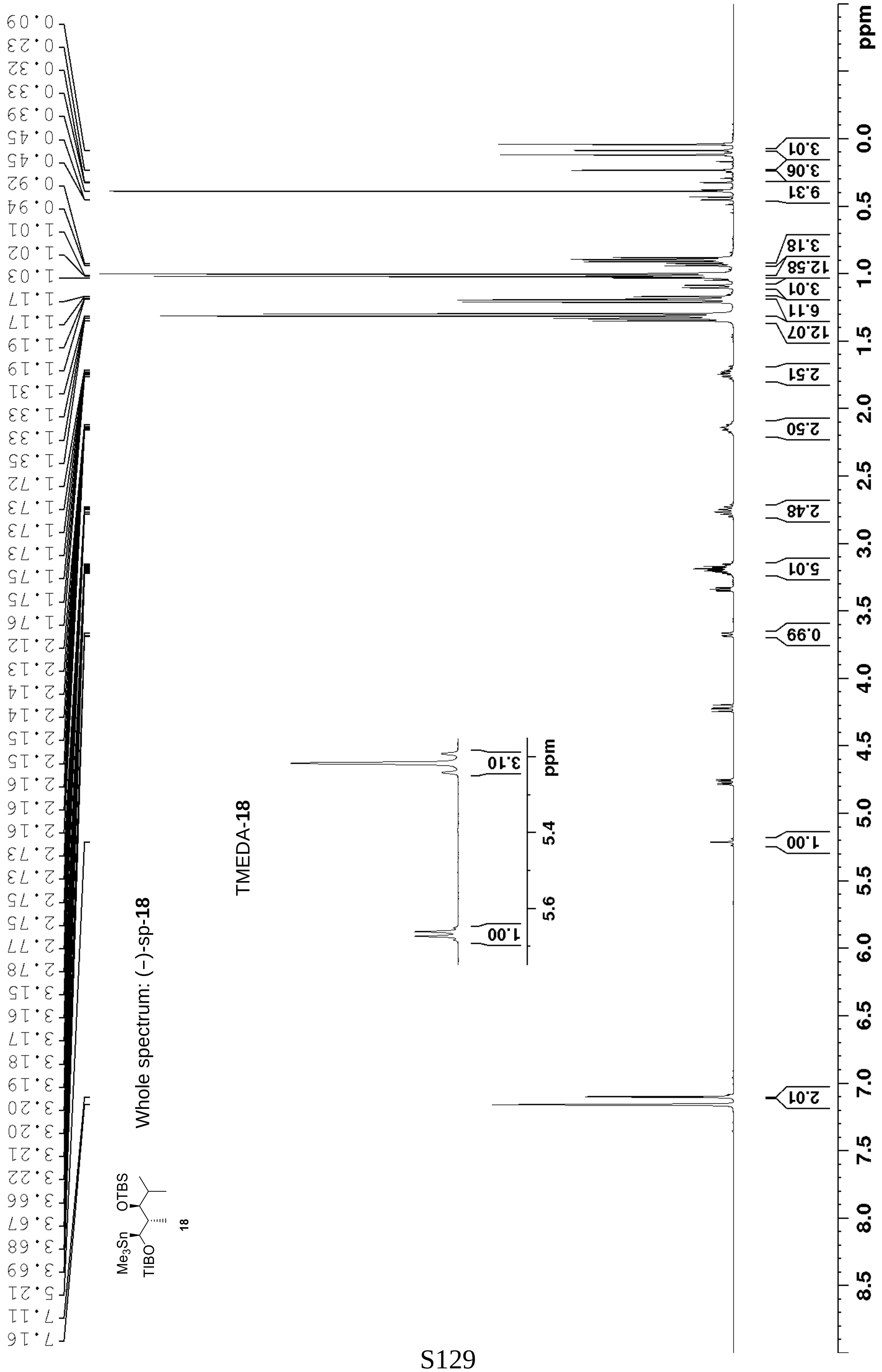

Whole spectrum: (−)-sp-18
TMEDA-18
Me3Sn
TIBO
OTBS
18
0.09 0.23 0.32 0.33 0.39 0.45 0.45 0.92 0.94 1.01 1.02 1.03 1.17 1.17 1.19 1.19 1.31 1.33 1.33 1.35 1.72 1.73 1.73 1.73 1.75 1.75 1.76 2.12 2.13 2.14 2.14 2.15 2.15 2.16 2.16 2.16 2.73 2.73 2.75 2.75 2.77 2.78 3.15 3.16 3.17 3.18 3.19 3.20 3.20 3.21 3.22 3.66 3.67 3.68 3.69 5.21 7.11 7.16
3.01 3.06 9.31 3.18 12.58 3.01 6.11 12.07 2.51 2.50 2.48 5.01 0.99 1.00 2.01
3.10 1.00
5.4 5.6
ppm
0.0 0.5 1.0 1.5 2.0 2.5 3.0 3.5 4.0 4.5 5.0 5.5 6.0 6.5 7.0 7.5 8.0 8.5
ppm

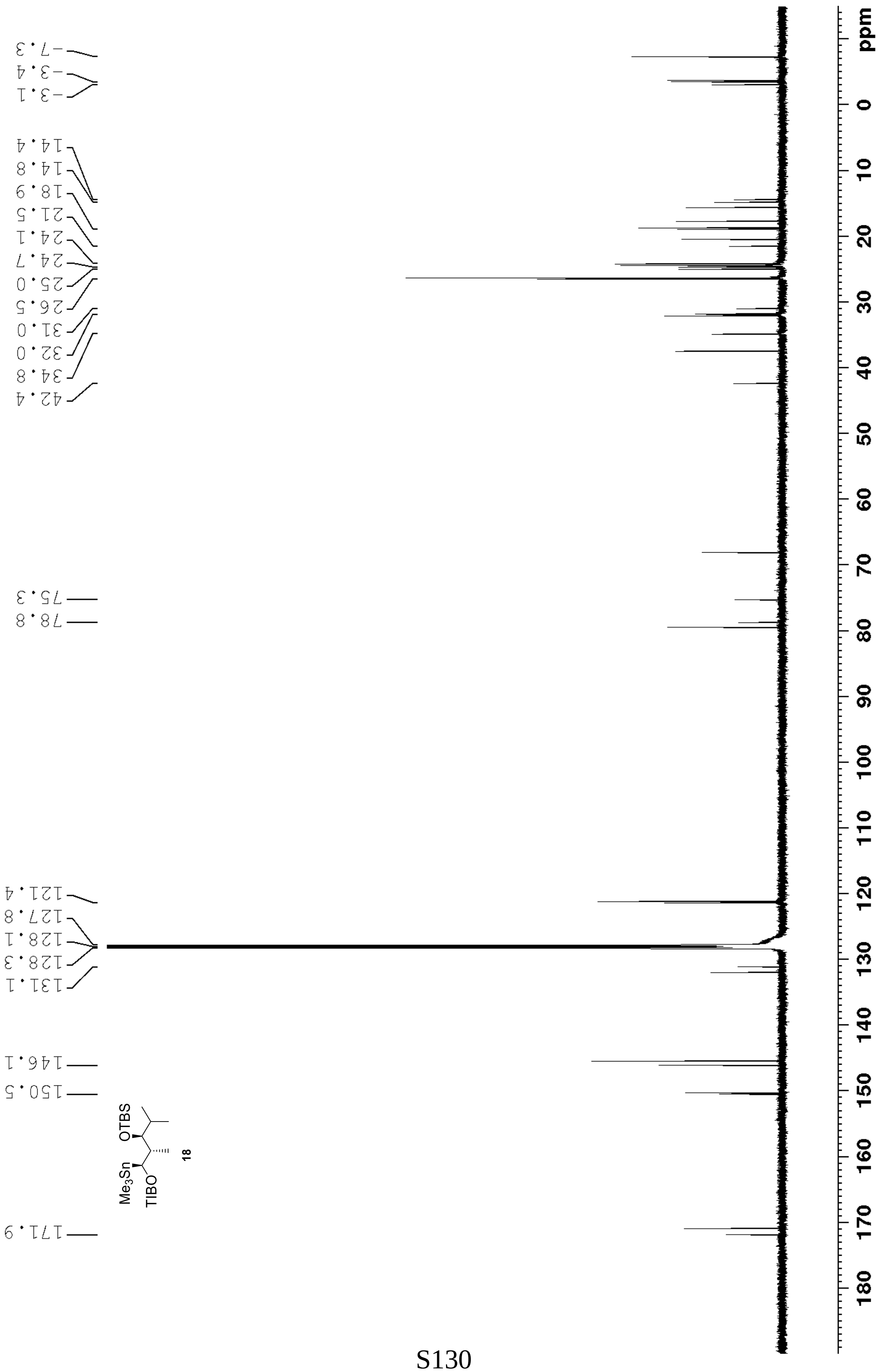

-7.3
-3.4
-3.1
14.4
14.8
18.9
21.5
24.1
24.7
25.0
26.5
31.0
32.0
34.8
42.4
75.3
78.8
121.4
127.8
128.1
128.3
131.1
146.1
150.5
171.9
Me3Sn
TIBO
OTBS
18
ppm

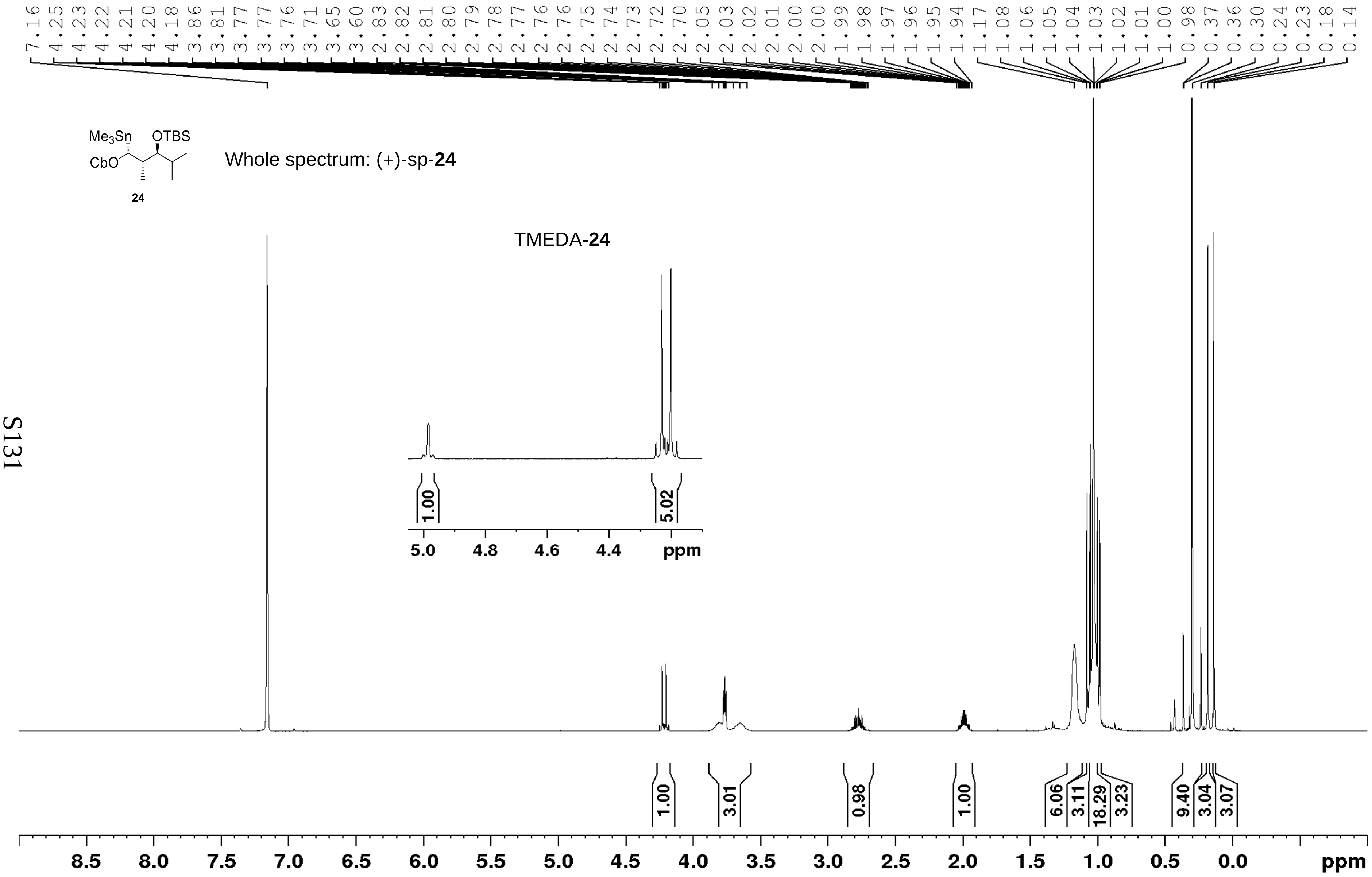
7.16 4.25 4.23 4.22 4.21 4.20 4.18 3.86 3.81 3.77 3.77 3.76 3.71 3.65 3.60 2.83 2.82 2.81 2.80 2.79 2.78 2.77 2.76 2.76 2.75 2.74 2.73 2.72 2.70 2.05 2.03 2.02 2.01 2.00 2.00 1.99 1.98 1.97 1.96 1.95 1.94 1.17 1.08 1.06 1.05 1.04 1.03 1.02 1.01 1.00 0.98 0.37 0.36 0.30 0.24 0.23 0.18 0.14
Me_3Sn
OTBS
CbO
24
Whole spectrum: (+)-sp-24
TMEDA-24
1.00
5.02
5.0
4.8
4.6
4.4
ppm
1.00
3.01
0.98
1.00
6.06
3.11
18.29
3.23
9.40
3.04
3.07
8.5 8.0 7.5 7.0 6.5 6.0 5.5 5.0 4.5 4.0 3.5 3.0 2.5 2.0 1.5 1.0 0.5 0.0 ppm

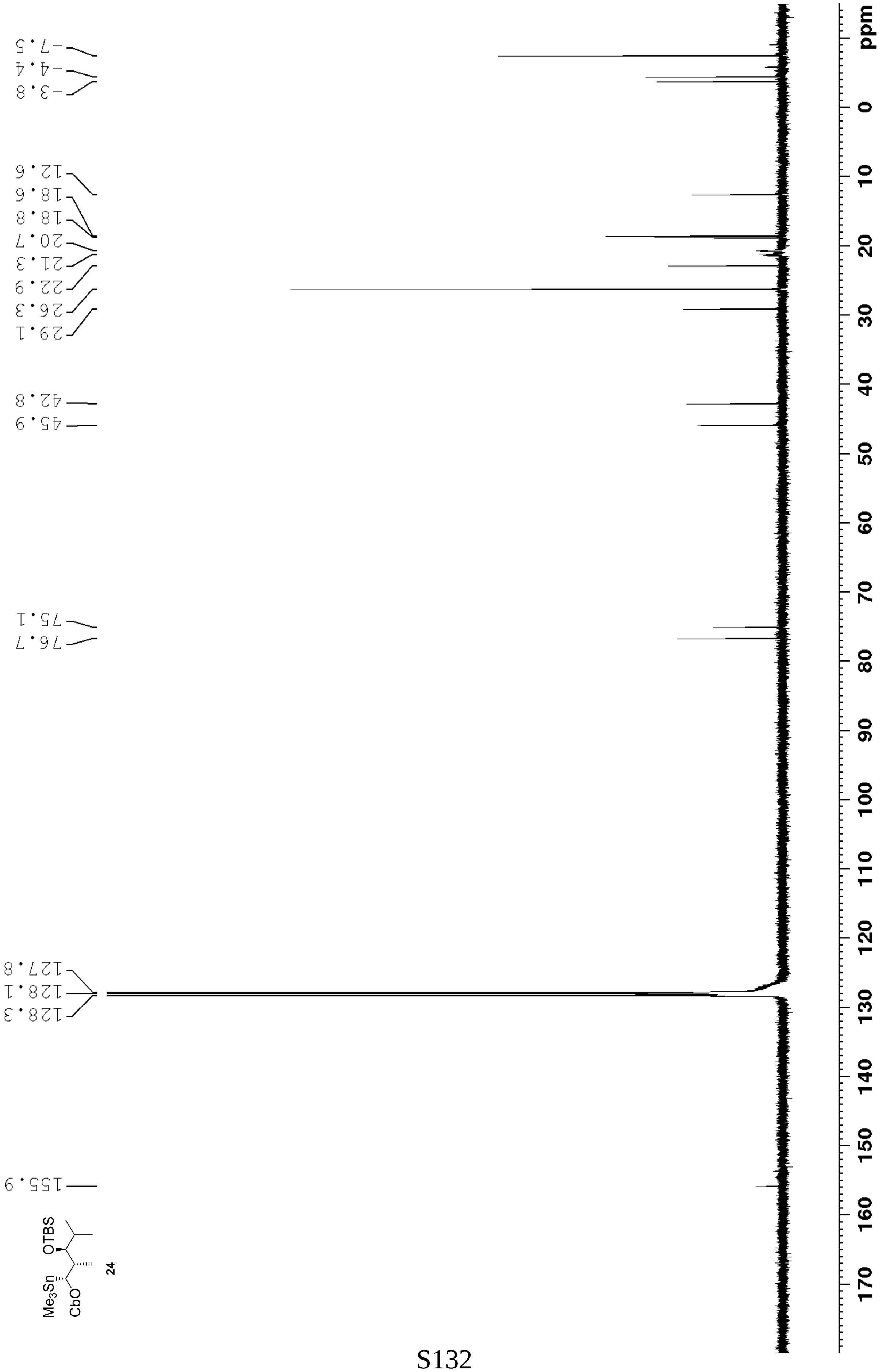
-7.5
-4.4
-3.8
12.6
18.6
18.8
20.7
21.3
22.9
26.3
29.1
42.8
45.9
75.1
76.7
127.8
128.1
128.3
155.9
Me3Sn
OTBS
CbO
24
ppm

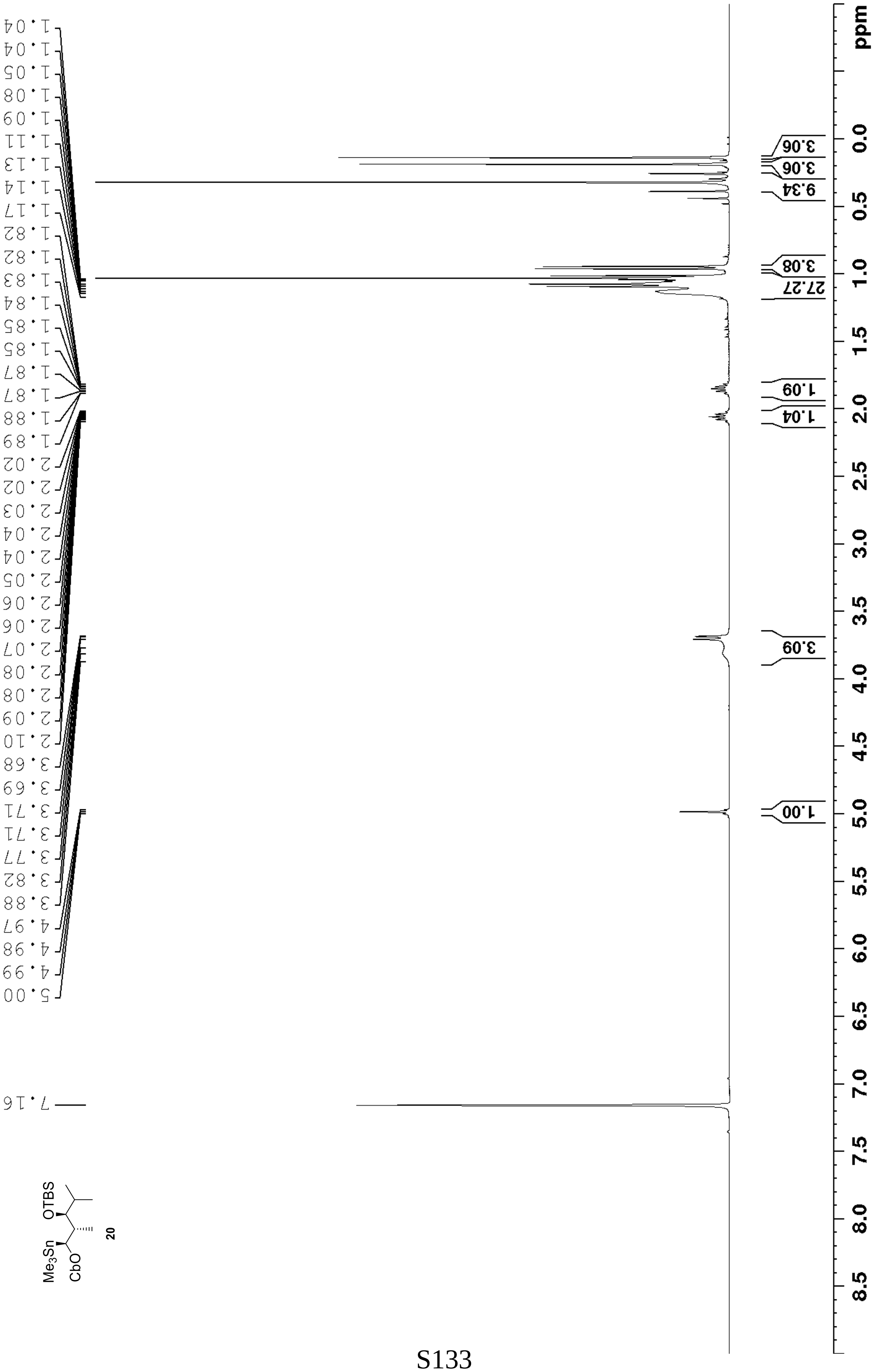
1.04
1.04
1.05
1.08
1.09
1.11
1.13
1.14
1.17
1.82
1.82
1.83
1.84
1.85
1.85
1.87
1.87
1.88
1.89
2.02
2.02
2.03
2.04
2.04
2.05
2.06
2.06
2.07
2.08
2.08
2.09
2.10
3.68
3.69
3.71
3.71
3.77
3.82
3.88
4.97
4.98
4.99
5.00
7.16
3.06
3.06
9.34
3.08
27.27
1.09
1.04
3.09
1.00
ppm
Me3Sn
CbO
OTBS
20

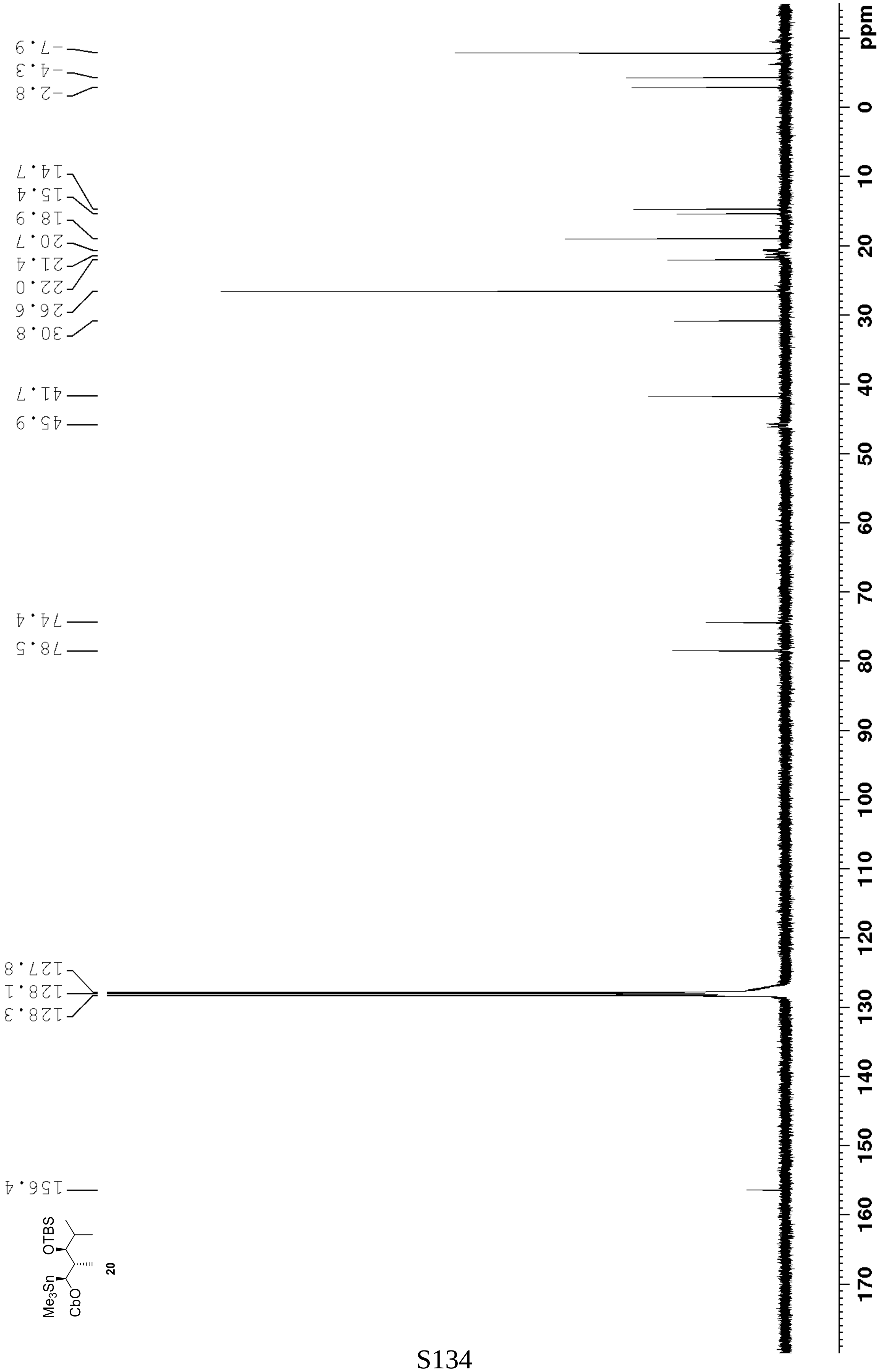

-7.9
-4.3
-2.8
14.7
15.4
18.9
20.7
21.4
22.0
26.6
30.8
41.7
45.9
74.4
78.5
127.8
128.1
128.3
156.4
Me3Sn
CbO
OTBS
20
ppm
0
10
20
30
40
50
60
70
80
90
100
110
120
130
140
150
160
170

6. Appendix

Below are the coordinates of the conformers that were considered for the Boltzmann factors but are not shown in the main paper.

Coordinates of **10-c4** (2.73 kJ/mol).

	Coordinates		
Atom	x / A	y / A	z / A
H	4.0593	-4.1175	0.1459
H	2.2965	-4.1035	0.0348
H	2.4984	-2.7842	-2.1744
H	4.2603	-2.7900	-2.0225
H	2.3485	1.8418	2.6031
H	3.8949	2.6015	2.2010
H	3.3933	-1.2571	-2.2149
H	-1.7931	0.8810	1.4029
H	-1.0037	1.7848	-2.5664
H	2.2808	-0.8389	2.6530
H	4.5770	-0.4045	-0.0943
C	-2.4048	0.2590	-0.9536
O	-1.3260	1.0361	-0.6573
C	-0.4400	1.3121	-1.7582
C	0.6642	2.2307	-1.2544
C	1.3852	1.6379	-0.0249
C	2.4849	2.5539	0.5487
C	1.9566	3.9645	0.8449
C	3.1029	1.9516	1.8151
O	-2.6707	-0.0799	-2.1020
H	-2.1216	-0.4898	3.4101
H	-0.7674	-1.5546	-0.1577
H	5.3926	-1.9698	0.0732
H	4.4570	-1.3132	1.4212
H	0.1628	-3.0415	-0.3170
H	0.1418	-1.8706	-1.6397
H	-3.4055	-1.4160	2.6311
C	1.6217	2.5329	-2.4133
O	1.9616	0.3813	-0.3884
Si	1.6662	-1.1488	0.2314
C	0.1618	-1.9710	-0.5512
C	1.3771	-1.0935	2.0937
C	3.2367	-2.1302	-0.2114
C	4.4839	-1.4085	0.3298
C	3.1610	-3.5406	0.4034
C	3.3485	-2.2444	-1.7439
H	1.0256	-2.0706	2.4420
H	0.6065	-0.3603	2.3525

Coordinates of **10-c5** (2.88 kJ/mol).

	Coordinates		
Atom	x / A	y / A	z / A
H	4.2685	-3.2084	-0.3056
H	4.6406	-2.5817	1.3083
H	4.0873	0.1653	-2.0179
H	3.9813	-1.5820	-2.2849
H	1.2740	1.6721	2.8353
H	2.2621	3.1372	2.7520
H	5.5427	-0.8400	-1.9160
H	-3.8748	-2.0000	1.1857
H	-1.0680	0.8228	-2.7986
H	1.0125	-0.9495	2.3058
H	4.6387	1.0768	0.3258
C	-2.6684	0.2996	-0.9013
O	-1.4096	-0.2060	-1.0309
C	-0.5231	0.5368	-1.8972
C	0.0532	1.7772	-1.2178
C	0.8070	1.4261	0.0799
C	1.2418	2.6618	0.8941
C	0.0539	3.5821	1.2083
C	1.9464	2.2514	2.1916
O	-3.0384	1.3088	-1.4945
H	-1.6877	-2.3914	2.1693
H	1.6235	-2.1086	-1.8664
H	6.0672	0.0287	0.3848
H	4.8706	-0.0355	1.6840
H	0.3455	-2.0891	-0.6450
H	1.7075	-3.2032	-0.4793
H	-0.9388	-1.2072	1.0873
C	0.9276	2.5381	-2.2208
O	1.9638	0.6514	-0.2489
Si	2.3750	-0.9078	0.2038
C	1.4231	-2.1931	-0.7939
C	2.0350	-1.2250	2.0316
C	4.2362	-1.0145	-0.1777
C	4.9914	0.0783	0.6010
C	4.7702	-2.3990	0.2359
C	4.4691	-0.8054	-1.6861
H	2.7141	-0.6683	2.6823
H	2.1551	-2.2917	2.2492

H	3.0971	-3.5050	1.4965	H	5.8438	-2.4754	0.0176
H	-0.0296	0.3722	-2.1336	H	0.2638	-0.1711	-2.1561
H	-1.7446	-1.5203	2.0193	H	-2.1115	-0.6743	2.3038
H	0.6323	1.4893	0.7611	H	0.1277	0.8364	0.7124
H	3.2749	2.6244	-0.2095	H	1.9650	3.2158	0.2824
H	1.6620	4.5020	-0.0600	H	-0.3616	4.0516	0.3133
H	2.7256	4.5599	1.3464	H	0.3625	4.3823	1.8879
H	1.0857	3.9233	1.5104	H	-0.7529	3.0243	1.6996
H	3.5376	0.9709	1.6213	H	2.8312	1.6458	1.9921
N	-3.1149	-0.0885	0.1550	N	-3.4397	-0.4363	-0.0529
C	-2.7361	0.3503	1.5148	C	-3.0050	-1.6888	0.6024
C	-2.4914	-0.8446	2.4434	C	-1.8619	-1.4732	1.5997
H	-4.9463	-2.9055	-0.5533	H	-4.9610	-0.4925	2.3129
H	-3.6259	-2.2501	-1.5455	H	-4.2933	1.1211	1.9991
H	-3.3040	-2.8030	0.1095	H	-6.0301	0.8167	1.8047
C	-4.0328	-2.3029	-0.5349	C	-5.0331	0.3879	1.6658
C	-3.7648	1.3315	2.0874	C	-2.7221	-2.8109	-0.4029
H	-4.7433	0.8602	2.2248	H	-1.8659	-2.5715	-1.0345
H	-3.4329	1.6961	3.0643	H	-2.5087	-3.7422	0.1311
H	-3.8875	2.1899	1.4212	H	-3.5912	-2.9758	-1.0457
C	-4.3433	-0.8984	-0.0072	C	-4.8176	0.0377	0.1901
H	-4.7383	-1.0083	1.0054	H	-4.9006	0.9540	-0.3938
C	-5.4115	-0.1752	-0.8342	C	-5.8570	-0.9580	-0.3341
H	-6.3360	-0.7611	-0.8383	H	-5.6915	-1.1637	-1.3952
H	-5.0766	-0.0410	-1.8633	H	-6.8625	-0.5426	-0.2166
H	-5.6302	0.8069	-0.4050	H	-5.8280	-1.9078	0.2098
H	0.1832	3.1607	-0.9309	H	-0.8032	2.4003	-0.9485
H	2.1936	1.6388	-2.6766	H	0.3757	2.7352	-3.1451
H	2.3297	3.3234	-2.1533	H	1.8224	1.9604	-2.4681
H	1.0703	2.8613	-3.3000	H	1.2500	3.5016	-1.8180

Coordinates of **10-c6** (3.23 kJ/mol).

Atom	Coordinates x / A	y / A	z / A
H	2.2558	-1.4762	-2.1838
H	1.0363	-2.5570	-1.4942
H	4.8711	-1.9705	0.4845
H	4.4652	-1.0470	-0.9706
H	3.3997	3.5928	1.9463
H	3.4575	1.8391	1.7251
H	4.7655	-2.7897	-1.0789
H	-2.4481	1.7883	0.5197
H	-0.5802	0.7249	-2.8852
H	4.0290	-0.4260	2.2869
H	3.1542	-3.6142	1.4929
C	-2.1297	-0.2769	-0.8687
O	-1.2852	0.7879	-0.9379
C	-0.1892	0.6592	-1.8648
C	0.7893	1.7919	-1.5854
C	1.4083	1.6700	-0.1752
C	2.2622	2.8866	0.2338
C	1.4721	4.1986	0.1276
C	2.8143	2.7158	1.6523
O	-1.9638	-1.2892	-1.5456
H	-3.9902	0.2655	2.6854
H	-0.3036	-1.6302	0.3485
H	3.1069	-4.4429	-0.0667
H	1.6052	-3.8799	0.6760
H	-0.2669	-0.3713	1.5881
H	0.1981	-2.0235	1.9982
H	-2.2273	0.3722	2.5435
C	1.8512	1.8381	-2.6898
O	2.2098	0.4870	-0.1302
Si	2.0502	-0.8915	0.8120
C	0.2492	-1.2603	1.2144
C	2.9856	-0.7174	2.4403
C	2.8185	-2.2881	-0.2305
C	2.6589	-3.6269	0.5160
C	2.1138	-2.3876	-1.5959
C	4.3150	-2.0030	-0.4585
H	2.9799	-1.6702	2.9810
H	2.5194	0.0318	3.0871
H	2.5249	-3.2247	-2.1763
H	0.2774	-0.3175	-1.7456
H	-3.1891	1.8282	2.8643
H	0.5804	1.5876	0.5435
H	3.1162	2.9342	-0.4535

Coordinates of **10-c7** (3.83 kJ/mol).

Atom	Coordinates x / A	y / A	z / A
H	4.7741	-0.4147	-1.8128
H	5.9292	-0.1660	-0.4967
H	5.3169	-0.7123	1.8962
H	3.6902	-1.2676	2.3132
H	1.7559	3.6893	2.4531
H	1.5045	3.9691	0.7287
H	3.9441	0.4012	1.7794
H	-4.6749	-0.0690	1.1354
H	-1.1236	1.9708	-2.8168
H	0.0842	-1.9571	0.4723
H	4.4264	-2.8551	-1.0579
C	-2.1292	-0.3702	-0.9842
O	-1.9838	0.9493	-1.2795
C	-0.7887	1.3449	-1.9854
C	0.1416	2.1349	-1.0618
C	0.5829	1.2939	0.1671
C	0.6335	2.1042	1.4791
C	0.8075	1.1703	2.6817
C	1.7075	3.1973	1.4763
O	-1.3952	-1.2440	-1.4427
H	-5.5418	1.8889	-0.0419
H	0.8687	-1.4467	-2.3252
H	3.9927	-3.2084	0.6218
H	5.6025	-2.5897	0.2343
H	2.3435	-2.4225	-2.3021
H	2.4271	-0.7228	-2.7877
H	-4.2358	1.7907	-1.2383
C	1.3115	2.6703	-1.8963
O	1.8473	0.6822	-0.0640
Si	2.2035	-0.9389	-0.3037
C	1.9389	-1.4239	-2.1033
C	1.1284	-2.0306	0.7900
C	4.0481	-1.0620	0.1560
C	4.5378	-2.5110	-0.0240
C	4.8630	-0.1282	-0.7592
C	4.2562	-0.6351	1.6202
H	1.1981	-1.7582	1.8467
H	1.4280	-3.0794	0.6886
H	4.5298	0.9102	-0.6643
H	-0.2985	0.4569	-2.3839
H	-5.4470	0.4978	-1.1376
H	-0.1700	0.5142	0.3306
H	-0.3498	2.5850	1.5708

H	2.0589	5.0285	0.5333	H	0.7517	1.7301	3.6207
H	0.5397	4.1417	0.7026	H	1.7770	0.6665	2.6471
H	1.2150	4.4518	-0.9041	H	0.0288	0.3998	2.7009
H	1.9989	2.6021	2.3778	H	2.6898	2.7654	1.2639
N	-3.1379	-0.0881	0.0233	N	-3.1668	-0.5953	-0.1347
C	-3.2831	1.1545	0.8099	C	-3.9922	0.4712	0.4758
C	-3.1663	0.8828	2.3140	C	-4.8546	1.2082	-0.5542
H	-2.8130	-2.8876	0.0806	H	-5.0538	-2.2827	-1.1192
H	-2.9375	-2.2059	1.7133	H	-4.9562	-3.5020	0.1650
H	-4.2767	-3.1621	1.0490	H	-5.5916	-1.8967	0.5269
C	-3.4956	-2.4363	0.8015	C	-4.8404	-2.4372	-0.0580
C	-4.5792	1.8898	0.4511	C	-3.1848	1.4249	1.3643
H	-5.4669	1.3094	0.7215	H	-2.5430	2.0732	0.7679
H	-4.6302	2.8412	0.9894	H	-3.8689	2.0535	1.9429
H	-4.6182	2.0973	-0.6218	H	-2.5598	0.8648	2.0654
C	-4.1309	-1.1675	0.2228	C	-3.4133	-1.9935	0.2759
H	-4.8176	-0.7746	0.9761	H	-2.7247	-2.5842	-0.3276
C	-4.9502	-1.4397	-1.0429	C	-3.0592	-2.2071	1.7514
H	-4.3129	-1.8359	-1.8344	H	-3.7041	-1.6220	2.4154
H	-5.4237	-0.5205	-1.3999	H	-2.0202	-1.9234	1.9420
H	-5.7370	-2.1691	-0.8263	H	-3.1812	-3.2616	2.0169
H	0.2074	2.7196	-1.6114	H	-0.4376	2.9867	-0.6828
H	1.3844	1.8680	-3.6791	H	2.0117	3.2440	-1.2883
H	2.4963	0.9573	-2.6385	H	0.9416	3.3206	-2.6959
H	2.4843	2.7239	-2.5955	H	1.8705	1.8488	-2.3504

Coordinates of **10-c8** (4.03 kJ/mol).

Atom	Coordinates x / A	y / A	z / A
H	-4.3277	-0.9307	1.2100
H	-4.6567	-2.6485	1.4858
H	-3.1638	-4.4220	0.4430
H	-1.6820	-3.9766	-0.4120
H	0.0366	3.4961	-1.6248
H	-0.9328	4.4241	-0.4689
H	-3.2605	-3.6966	-1.1654
H	4.4745	0.4004	-1.1477
H	0.6940	1.4923	2.8861
H	-4.1471	-0.2087	-1.8736
H	-2.3290	-3.0890	2.4170
C	1.9826	-0.3278	0.9362
O	1.4949	0.9327	1.0755
C	0.3404	1.1102	1.9230
C	-0.6141	2.1094	1.2679
C	-1.1087	1.5468	-0.0805
C	-1.8323	2.5170	-1.0437
C	-3.2630	2.8889	-0.6379
C	-0.9873	3.7614	-1.3390
O	1.4972	-1.3014	1.5079
H	3.4811	2.2753	1.0390
H	-0.4755	-2.3550	-2.0741
H	-2.0565	-1.3431	2.2846
H	-0.8958	-2.4660	1.5775
H	0.2982	-1.7883	-0.5798
H	0.1114	-0.6944	-1.9609
H	4.9520	1.2852	1.1065
C	-1.7327	2.4345	2.2656
O	-1.9234	0.4082	0.1928
Si	-2.0536	-0.9782	-0.7421
C	-0.3682	-1.5012	-1.3968
C	-3.2181	-0.6872	-2.1979
C	-2.7759	-2.2765	0.4448
C	-1.9623	-2.2943	1.7527
C	-4.2415	-1.9288	0.7672
C	-2.7139	-3.6670	-0.2156
H	-3.4787	-1.6334	-2.6852
H	-2.7562	-0.0441	-2.9540
H	-4.8735	-1.9557	-0.1270
H	-0.1419	0.1463	2.0785
H	4.8070	2.5768	-0.0998
H	-0.2065	1.2216	-0.6138
H	-1.9113	1.9519	-1.9825

Coordinates of **10-c9** (4.95 kJ/mol).

Atom	Coordinates x / A	y / A	z / A
H	5.4471	-1.9639	-0.7848
H	4.4660	-0.5929	-1.3286
H	5.2766	-1.2909	1.6309
H	3.7135	-0.9175	2.3670
H	1.3132	2.1275	2.6291
H	2.9113	2.8855	2.5774
H	4.3841	0.1500	1.1214
H	-2.5667	1.6293	0.7685
H	-1.0031	1.5404	-2.8994
H	1.0449	-0.7144	2.3483
H	2.9658	-3.3131	1.7167
C	-2.2471	-0.1614	-0.9648
O	-1.5410	1.0011	-0.9721
C	-0.5300	1.1586	-1.9887
C	0.4957	2.1506	-1.4512
C	1.1272	1.6409	-0.1400
C	1.9497	2.6954	0.6303
C	1.2616	4.0656	0.7008
C	2.2428	2.1970	2.0507
O	-2.1114	-1.0224	-1.8305
H	-2.9707	1.2738	3.1727
H	1.6199	-1.8567	-2.3849
H	4.5400	-3.5811	0.9594
H	3.0609	-3.8429	0.0302
H	0.0356	-1.8910	-1.5939
H	1.2196	-3.1593	-1.2607
H	-3.5552	-0.3643	2.8742
C	1.5373	2.4674	-2.5277
O	1.9765	0.5241	-0.4224
Si	1.8166	-1.0955	-0.0161
C	1.1013	-2.0862	-1.4482
C	0.6872	-1.2767	1.4809
C	3.5854	-1.7258	0.3286
C	3.5262	-3.1976	0.7824
C	4.4178	-1.6246	-0.9647
C	4.2728	-0.8946	1.4249
H	0.6016	-2.3299	1.7675
H	-0.3202	-0.9244	1.2392
H	4.0032	-2.2452	-1.7657
H	-0.0825	0.1924	-2.2166
H	-1.8649	0.0452	2.5321
H	0.3040	1.3248	0.5139
H	2.9020	2.8060	0.0962

H	-3.7960	3.3167	-1.4934
H	-3.2813	3.6347	0.1615
H	-3.8126	2.0088	-0.2969
H	-1.4251	4.3358	-2.1614
N	3.0485	-0.3724	0.0900
C	3.6468	0.8114	-0.5652
C	4.2558	1.7983	0.4372
H	5.1417	-1.5096	1.4008
H	5.4859	-2.7794	0.2109
H	5.7586	-1.0866	-0.2082
C	5.0955	-1.7652	0.3385
C	2.7015	1.4906	-1.5628
H	2.2687	0.7549	-2.2464
H	1.8915	2.0072	-1.0479
H	3.2571	2.2240	-2.1557
C	3.6524	-1.6952	-0.1707
H	3.0517	-2.3915	0.4138
C	3.5321	-2.0825	-1.6481
H	3.9114	-3.0980	-1.7976
H	4.1116	-1.4168	-2.2962
H	2.4876	-2.0532	-1.9687
H	-0.0407	3.0200	1.0586
H	-2.3123	3.3042	1.9543
H	-1.3158	2.6536	3.2543
H	-2.4147	1.5862	2.3610

H	1.8220	4.7337	1.3623
H	0.2473	3.9755	1.1084
H	1.1909	4.5521	-0.2753
H	2.7102	1.2130	2.0441
N	-3.0861	-0.2506	0.1026
C	-3.2640	0.8520	1.0728
C	-2.8913	0.4187	2.4947
H	-2.3789	-2.5518	1.3646
H	-3.6840	-3.5723	0.7305
H	-2.4397	-2.9380	-0.3665
C	-3.0451	-2.7109	0.5120
C	-4.6806	1.4327	0.9959
H	-4.7666	2.2949	1.6642
H	-4.9070	1.7610	-0.0222
H	-5.4384	0.7032	1.2987
C	-3.9100	-1.4705	0.2650
H	-4.4903	-1.2919	1.1730
C	-4.9061	-1.6603	-0.8837
H	-5.5630	-2.5086	-0.6673
H	-4.3812	-1.8527	-1.8200
H	-5.5277	-0.7687	-1.0061
H	-0.0581	3.0641	-1.2101
H	1.0576	2.8230	-3.4450
H	2.1257	1.5780	-2.7663
H	2.2282	3.2446	-2.1896

Coordinates of **10-c10** (5.03 kJ/mol).

Atom	Coordinates x / A	y / A	z / A
H	-4.1910	-1.9980	1.8801
H	-5.6032	-1.6660	0.8693
H	-4.3815	0.2585	-1.1369
H	-5.3695	-1.1436	-1.5737
H	-2.6968	2.7442	-2.7942
H	-2.6173	1.1069	-2.1352
H	-3.7802	-0.9215	-2.3145
H	4.3316	0.0715	-1.5284
H	1.0650	1.5500	2.8214
H	-1.0968	-0.9506	-2.2743
H	-4.7933	-3.4408	-0.7684
C	2.2189	-0.3507	1.0096
O	1.6078	0.8595	0.9457
C	0.5851	1.1385	1.9272
C	-0.3745	2.1502	1.3115
C	-1.0381	1.5871	0.0386
C	-1.7751	2.6364	-0.8213
C	-0.9922	3.9469	-0.9827
C	-2.0837	2.0538	-2.2061
O	1.9538	-1.1859	1.8705
H	3.4949	2.2641	0.4159
H	-1.7924	-1.6677	2.5249
H	-3.3385	-3.7478	0.1850
H	-3.2017	-3.3190	-1.5271
H	-0.2178	-1.9138	1.7490
H	-1.5152	-3.0929	1.5185
H	5.0282	1.3710	0.4503
C	-1.3957	2.6106	2.3554
O	-1.9673	0.5595	0.3961
Si	-1.9161	-1.0920	0.1016
C	-1.2973	-2.0235	1.6154
C	-0.7801	-1.4570	-1.3579
C	-3.7203	-1.6203	-0.2331
C	-3.7570	-3.1155	-0.6050
C	-4.5537	-1.3922	1.0435
C	-4.3418	-0.8068	-1.3805
H	-0.7563	-2.5340	-1.5548
H	0.2455	-1.1436	-1.1377
H	-4.5303	-0.3421	1.3534
H	0.0814	0.2140	2.2045
H	4.6422	2.4194	-0.9267
H	-0.2411	1.1594	-0.5805
H	-2.7240	2.8529	-0.3138

Coordinates of **10-c11** (5.12 kJ/mol).

Atom	Coordinates x / A	y / A	z / A
H	4.5804	0.3683	-0.8394
H	4.6916	-1.1597	-1.7242
H	3.4391	-1.1399	2.4423
H	3.9012	0.3707	1.6426
H	0.0950	3.7539	1.5031
H	0.8908	3.2556	2.9962
H	5.1261	-0.8531	1.9966
H	-4.6668	-0.0922	1.2335
H	-1.2041	2.0463	-2.6663
H	1.0889	-2.7870	1.3359
H	3.5438	-3.3741	1.1202
C	-2.1214	-0.3090	-0.9005
O	-1.8923	1.0235	-1.0397
C	-0.7816	1.4336	-1.8649
C	0.2201	2.2468	-1.0410
C	0.8474	1.3605	0.0569
C	1.4812	2.0772	1.2703
C	2.7195	2.9202	0.9490
C	0.4492	2.8837	2.0661
O	-1.4637	-1.1684	-1.4833
H	-5.3583	0.6973	-1.0001
H	0.7080	-1.8980	-2.1793
H	5.2247	-3.0052	0.7196
H	4.0710	-3.3678	-0.5697
H	1.9180	-3.1271	-1.7669
H	2.4037	-1.6831	-2.6692
H	-5.4358	1.9867	0.2146
C	1.2362	2.8681	-2.0084
O	1.8059	0.4981	-0.5521
Si	2.0445	-1.1394	-0.2601
C	1.7465	-2.0509	-1.8746
C	0.8510	-1.7533	1.0630
C	3.8572	-1.3593	0.2970
C	4.1854	-2.8617	0.3944
C	4.7921	-0.7005	-0.7347
C	4.0866	-0.7067	1.6720
H	-0.1705	-1.7389	0.6741
H	0.8877	-1.1509	1.9760
H	5.8415	-0.8077	-0.4275
H	-0.3102	0.5505	-2.2964
H	-4.1037	1.9514	-0.9558
H	0.0290	0.7593	0.4737
H	1.8128	1.2532	1.9163

H	0.0171	3.7579	-1.3682
H	-0.8973	4.4995	-0.0445
H	-1.4987	4.6002	-1.7001
H	-1.1535	1.8787	-2.7606
N	3.1257	-0.5211	0.0069
C	3.5573	0.5475	-0.9224
C	4.2192	1.7235	-0.1954
H	5.4444	-1.2857	1.2161
H	5.7326	-2.7391	0.2414
H	5.8280	-1.1450	-0.5109
C	5.2880	-1.7393	0.2335
C	2.4583	0.9999	-1.8902
H	1.6974	1.5880	-1.3771
H	2.8977	1.6195	-2.6784
H	1.9758	0.1392	-2.3611
C	3.7924	-1.8367	-0.0825
H	3.3207	-2.4376	0.6947
C	3.5184	-2.5011	-1.4352
H	3.9448	-3.5087	-1.4476
H	3.9653	-1.9447	-2.2656
H	2.4424	-2.5796	-1.6139
H	0.2362	3.0069	1.0079
H	-2.0395	3.3980	1.9541
H	-0.8966	3.0077	3.2449
H	-2.0357	1.7783	2.6587

H	3.3880	2.3932	0.2640
H	3.2748	3.1413	1.8667
H	2.4479	3.8792	0.4971
H	-0.4218	2.2754	2.3282
N	-3.1464	-0.5660	-0.0431
C	-3.9479	0.4752	0.6379
C	-4.7582	1.3313	-0.3416
H	-5.0743	-3.3990	-0.1266
H	-5.6576	-1.7965	0.3280
H	-5.0233	-2.0748	-1.3055
C	-4.8934	-2.3269	-0.2495
C	-3.1258	1.3160	1.6206
H	-2.4350	1.9740	1.0939
H	-3.7956	1.9317	2.2292
H	-2.5510	0.6704	2.2909
C	-3.4754	-1.9831	0.2165
H	-2.7712	-2.5463	-0.3952
C	-3.2344	-2.3498	1.6844
H	-2.2040	-2.1254	1.9736
H	-3.4081	-3.4197	1.8337
H	-3.9072	-1.8096	2.3586
H	-0.3419	3.0478	-0.5461
H	1.9028	3.5688	-1.5064
H	0.7197	3.4129	-2.8055
H	1.8503	2.0882	-2.4642

Coordinates of **10-c12** (5.18 kJ/mol).

Atom	x / A	y / A	z / A
H	-3.3701	-3.8085	0.3727
H	-2.9153	-3.5859	-1.3238
H	-5.5832	-1.6022	0.4661
H	-4.5388	-0.2584	0.9602
H	-0.1119	2.8539	-2.2035
H	-0.8734	4.1007	-1.2022
H	-4.3539	-1.8486	1.7135
H	4.3705	0.0303	-1.5441
H	1.1362	2.0978	2.5798
H	-0.7405	-1.0227	-2.0561
H	-3.2445	-1.2736	-2.4542
C	2.2030	-0.1479	0.9767
O	1.6929	1.1065	0.8633
C	0.6642	1.4938	1.7984
C	-0.4034	2.3046	1.0629
C	-1.0435	1.4414	-0.0442
C	-1.8840	2.1638	-1.1205
C	-3.2321	2.7088	-0.6354
C	-1.0738	3.2411	-1.8505
O	1.8512	-0.9375	1.8492
H	4.7982	2.3839	-1.0814
H	-0.2949	-1.8750	2.0702
H	-3.9494	0.0593	-1.5262
H	-4.9415	-1.3368	-1.9625
H	-1.3724	-3.2226	1.6614
H	-1.9835	-1.8664	2.6211
H	3.6286	2.3779	0.2514
C	-1.3950	2.8443	2.1012
O	-1.8196	0.4128	0.5704
Si	-1.7967	-1.2301	0.2197
C	-1.3201	-2.1366	1.7931
C	-0.5427	-1.5909	-1.1419
C	-3.5540	-1.7326	-0.3282
C	-3.9373	-1.0279	-1.6417
C	-3.6100	-3.2575	-0.5426
C	-4.5610	-1.3359	0.7680
H	-0.5693	-2.6555	-1.3981
H	0.4736	-1.3592	-0.8118
H	-4.6184	-3.5620	-0.8539
H	0.2374	0.6008	2.2543
H	5.1111	1.4094	0.3662
H	-0.2077	0.9855	-0.5868
H	-2.1055	1.3745	-1.8518

Coordinates of **10-c13** (5.53 kJ/mol).

Atom	x / A	y / A	z / A
H	0.6640	-1.9273	-1.1917
H	1.0912	-3.0483	0.1010
H	2.6422	-0.7000	-2.4861
H	3.4043	-2.2781	-2.7459
H	2.0675	3.5568	2.7070
H	2.6961	2.0094	2.1419
H	4.3052	-0.9728	-1.9632
H	-1.1724	-1.6746	0.4378
H	-0.8474	0.8242	-2.8758
H	5.0901	0.1900	0.2316
H	4.8215	-2.5808	-0.0631
C	-2.3065	0.1221	-0.8996
O	-1.0019	-0.2290	-1.0955
C	-0.2463	0.6106	-1.9900
C	0.1970	1.9105	-1.3242
C	0.8960	1.6485	0.0235
C	1.2455	2.9400	0.7927
C	0.0314	3.8724	0.9211
C	1.8015	2.6313	2.1869
O	-2.8458	1.0249	-1.5330
H	-2.7452	-3.2744	-0.5816
H	1.9856	-1.9337	2.4144
H	3.8304	-3.8535	-0.7853
H	3.5181	-3.3295	0.8723
H	0.5984	-1.0631	1.7584
H	1.7217	-0.2396	2.8467
H	-2.1840	-3.8484	1.0006
C	1.0693	2.7018	-2.3048
O	2.0769	0.8801	-0.2246
Si	2.7049	-0.4672	0.5504
C	1.6518	-0.9635	2.0309
C	4.4524	-0.0202	1.0967
C	2.8093	-1.9313	-0.6756
C	3.8031	-2.9759	-0.1256
C	1.4381	-2.6140	-0.8432
C	3.3165	-1.4383	-2.0433
H	4.9110	-0.8409	1.6575
H	4.4614	0.8665	1.7369
H	1.5094	-3.4337	-1.5711
H	0.6144	0.0034	-2.2692
H	-3.8093	-3.1765	0.8348
H	0.2051	1.0655	0.6482
H	2.0285	3.4554	0.2224

H	-3.7302	1.9920	0.0215	H	-0.8239	3.3422	1.3576
H	-3.8887	2.9050	-1.4896	H	-0.2857	4.2845	-0.0397
H	-3.1188	3.6511	-0.0920	H	0.2679	4.7139	1.5795
H	-1.6249	3.6098	-2.7213	H	1.0534	2.1111	2.7969
N	3.1230	-0.4158	0.0080	N	-2.9092	-0.6353	0.0548
C	3.6157	0.5764	-0.9738	C	-2.1968	-1.6929	0.8013
C	4.3295	1.7612	-0.3129	C	-2.7737	-3.0785	0.4937
H	5.3545	-1.2020	1.3523	H	-5.0752	-1.6981	-1.2107
H	5.6316	-2.7098	0.4601	H	-6.2938	-0.5999	-0.5366
H	5.8109	-1.1575	-0.3620	H	-5.0524	0.0237	-1.6437
C	5.2222	-1.6978	0.3867	C	-5.2445	-0.6842	-0.8365
C	2.5401	1.0174	-1.9724	C	-2.1685	-1.3904	2.3038
H	2.0329	0.1499	-2.4031	H	-3.1679	-1.4165	2.7486
H	1.7958	1.6521	-1.4914	H	-1.5575	-2.1363	2.8208
H	3.0034	1.5844	-2.7861	H	-1.7355	-0.4036	2.4905
C	3.7381	-1.7600	0.0119	C	-4.3371	-0.3956	0.3637
H	3.2106	-2.3017	0.7965	H	-4.5794	-1.1286	1.1366
C	3.5002	-2.4877	-1.3149	C	-4.5671	0.9961	0.9621
H	2.4324	-2.5379	-1.5435	H	-3.9347	1.1452	1.8422
H	3.8866	-3.5092	-1.2493	H	-5.6124	1.1022	1.2687
H	4.0088	-1.9983	-2.1519	H	-4.3355	1.7702	0.2295
H	0.1038	3.1458	0.5756	H	-0.7161	2.4750	-1.1173
H	-2.0466	3.6103	1.6803	H	2.0245	2.1935	-2.4629
H	-0.8618	3.2933	2.9459	H	1.2794	3.7076	-1.9323
H	-2.0224	2.0350	2.4820	H	0.5693	2.8066	-3.2729

Coordinates of **10-c14** (5.81 kJ/mol).

	Coordinates		
Atom	x / A	y / A	z / A
H	4.0117	0.3589	1.4236
H	5.1255	-0.9251	1.9179
H	3.5331	-3.6952	-0.0733
H	3.1578	-3.3292	1.6178
H	0.2232	4.0231	1.0160
H	0.9496	3.6864	2.5881
H	4.8327	-3.2924	1.0551
H	-2.6849	1.6375	0.7228
H	-1.0660	1.8860	-2.8945
H	0.9337	-0.7672	2.2375
H	5.6477	-1.4317	-0.4955
C	-2.2282	-0.1326	-0.9964
O	-1.7420	1.1357	-1.1016
C	-0.6584	1.3629	-2.0239
C	0.4110	2.2098	-1.3310
C	0.9474	1.4563	-0.0950
C	1.5752	2.3025	1.0319
C	2.8347	3.0763	0.6322
C	0.5356	3.2181	1.6896
O	-1.9287	-1.0271	-1.7832
H	-3.5387	-0.4153	2.8299
H	0.2742	-1.9603	-1.7718
H	4.5247	-0.1762	-1.0478
H	4.3649	-1.8351	-1.6441
H	1.3562	-3.2736	-1.2766
H	1.9326	-2.0321	-2.3997
H	-1.8786	0.0982	2.4786
C	1.4802	2.5758	-2.3679
O	1.8709	0.4577	-0.5277
Si	1.8738	-1.1569	-0.0636
C	1.3099	-2.2056	-1.5161
C	0.7002	-1.4179	1.3892
C	3.6708	-1.5589	0.4210
C	4.6011	-1.2301	-0.7620
C	4.0850	-0.7116	1.6379
C	3.7986	-3.0527	0.7730
H	0.7567	-2.4551	1.7361
H	-0.3357	-1.2328	1.0923
H	3.4603	-0.9236	2.5127
H	-0.2557	0.4035	-2.3513
H	-3.0559	1.2566	3.1261
H	0.0787	0.9717	0.3660
H	1.8772	1.5605	1.7837

Coordinates of **10-c15** (6.66 kJ/mol).

	Coordinates		
Atom	x / A	y / A	z / A
H	4.7545	-1.0518	-1.7713
H	5.9836	-0.2231	-0.8074
H	4.1170	1.2445	1.1686
H	5.5783	0.3468	1.6140
H	2.6163	3.0219	0.1819
H	1.6235	4.2604	0.9701
H	4.0381	-0.0276	2.3969
H	-2.6367	-1.1891	-1.7868
H	-1.5828	2.8087	-1.1887
H	1.6457	-2.5248	1.8053
H	4.6188	-2.9146	0.0045
C	-2.6857	0.8044	-0.2619
O	-2.1861	0.8567	-1.5312
C	-1.3472	1.9842	-1.8605
C	0.1300	1.5774	-1.8157
C	0.5780	1.2014	-0.3838
C	0.5782	2.3941	0.5971
C	0.7915	1.9233	2.0396
C	1.6151	3.4604	0.2230
O	-2.6951	1.7757	0.4892
H	-4.9722	-2.5501	-0.3454
H	2.4512	-1.9473	-2.2390
H	4.3192	-2.5031	1.7007
H	5.8590	-2.0771	0.9444
H	0.8961	-2.2824	-1.4710
H	2.3318	-3.1807	-0.9739
H	-5.1006	-1.3274	-1.6241
C	0.3998	0.4582	-2.8256
O	1.8867	0.6260	-0.4231
Si	2.3385	-0.9175	0.0500
C	1.9713	-2.1951	-1.2881
C	1.4326	-1.4684	1.6099
C	4.2135	-0.7804	0.3405
C	4.7769	-2.1480	0.7707
C	4.9011	-0.3283	-0.9620
C	4.4970	0.2562	1.4434
H	0.3483	-1.3709	1.4965
H	1.7248	-0.8931	2.4913
H	4.5147	0.6381	-1.3005
H	-1.6249	2.2648	-2.8794
H	-4.3809	-2.9123	-1.9685
H	-0.1292	0.4527	-0.0007
H	-0.4221	2.8351	0.5524

H	3.3593	3.4356	1.5237
H	2.5921	3.9527	0.0230
H	3.5204	2.4413	0.0654
H	-0.3604	2.6602	1.9842
N	-3.0686	-0.2763	0.0635
C	-3.3295	0.8159	1.0264
C	-2.9268	0.4078	2.4478
H	-2.2769	-2.5079	1.4538
H	-3.4577	-3.6252	0.7487
H	-2.1880	-2.9154	-0.2714
C	-2.8678	-2.7228	0.5595
C	-4.7805	1.3012	0.9442
H	-5.0233	1.6128	-0.0755
H	-5.4904	0.5242	1.2451
H	-4.9257	2.1568	1.6106
C	-3.8013	-1.5531	0.2322
H	-4.4391	-1.3910	1.1041
C	-4.7223	-1.8470	-0.9566
H	-4.1398	-2.0264	-1.8612
H	-5.3995	-1.0069	-1.1355
H	-5.3258	-2.7355	-0.7466
H	-0.0780	3.1258	-0.9785
H	2.0119	1.6801	-2.6970
H	2.2158	3.2732	-1.9675
H	1.0170	3.0446	-3.2424

H	1.7923	1.5039	2.1743
H	0.0595	1.1602	2.3202
H	0.6851	2.7619	2.7352
H	1.4090	3.9215	-0.7475
N	-3.1637	-0.4284	0.0536
C	-3.0779	-1.5782	-0.8722
C	-4.4685	-2.1215	-1.2174
H	-5.4841	-0.0848	2.5632
H	-4.7944	1.2435	1.6069
H	-5.7486	-0.0247	0.8104
C	-5.0246	0.1774	1.6050
C	-2.1442	-2.6629	-0.3241
H	-2.5223	-3.0998	0.6055
H	-2.0441	-3.4727	-1.0532
H	-1.1501	-2.2523	-0.1295
C	-3.7512	-0.6476	1.3951
H	-4.0398	-1.7012	1.4013
C	-2.7255	-0.4491	2.5163
H	-2.4005	0.5909	2.5605
H	-1.8497	-1.0846	2.3560
H	-3.1707	-0.7202	3.4787
H	0.6996	2.4603	-2.1315
H	-0.1421	-0.4521	-2.5546
H	1.4629	0.2230	-2.8709
H	0.0690	0.7591	-3.8251

Coordinates of **10-c16** (7.04 kJ/mol).

Atom	Coordinates x / A	y / A	z / A
H	5.4993	-0.2260	1.8124
H	3.9250	-0.7098	2.4563
H	5.7037	-2.4328	0.5745
H	4.4453	-2.9666	-0.5458
H	2.5601	3.0624	0.8800
H	1.5067	4.1234	1.8307
H	4.1335	-2.9545	1.1970
H	-3.3884	1.6700	-0.1680
H	-1.0010	1.4749	-3.1550
H	1.5182	-1.4142	2.2949
H	4.5211	0.7968	-0.9664
C	-2.2159	-0.4006	-0.9608
O	-1.9490	0.8379	-1.4653
C	-0.7235	1.0135	-2.2031
C	0.2182	1.9245	-1.4153
C	0.5941	1.2996	-0.0424
C	0.5382	2.3121	1.1184
C	0.7234	1.6046	2.4648
C	1.5435	3.4582	0.9620
O	-1.5916	-1.4061	-1.2923
H	-4.0608	0.3397	2.5080
H	2.2663	-1.2040	-2.5480
H	4.7011	-0.7413	-1.8241
H	5.9426	-0.2220	-0.6763
H	0.7610	-1.8192	-1.8286
H	2.2456	-2.7754	-1.7342
H	-2.5269	1.1193	2.0770
C	1.4289	2.2745	-2.2874
O	1.8857	0.7011	-0.0888
Si	2.2528	-0.9350	-0.0572
C	1.8459	-1.7557	-1.7013
C	1.2841	-1.8059	1.3014
C	4.1247	-0.9567	0.2834
C	4.8602	-0.2386	-0.8638
C	4.4201	-0.2266	1.6067
C	4.6226	-2.4107	0.3813
H	1.5023	-2.8792	1.2964
H	0.2094	-1.6931	1.1339
H	4.0842	0.8139	1.5700
H	-0.2787	0.0380	-2.3999
H	-4.0170	2.0755	2.1851
H	-0.1503	0.5301	0.1966
H	-0.4777	2.7325	1.0937

Coordinates of **10-c17** (7.32 kJ/mol).

Atom	Coordinates x / A	y / A	z / A
H	6.2050	-2.0958	0.0989
H	4.7239	-2.8836	-0.4585
H	4.3835	-0.9842	-2.1709
H	5.8695	-0.2193	-1.5945
H	-1.4517	2.7279	0.9954
H	-0.8875	2.8555	2.6649
H	4.3535	0.6996	-1.6240
H	-3.9611	-2.1227	0.9027
H	-1.0767	0.9285	-2.8855
H	1.0916	-1.2711	2.2090
H	6.1890	0.3390	0.8417
C	-2.6592	0.3387	-0.9240
O	-1.4799	-0.2661	-1.2354
C	-0.5533	0.5165	-2.0199
C	0.1146	1.6382	-1.2180
C	0.7075	1.1036	0.1055
C	0.6172	2.0682	1.3045
C	1.3014	3.4174	1.0698
C	-0.8382	2.2411	1.7604
O	-2.9570	1.4567	-1.3351
H	-2.0002	-1.1558	2.0769
H	0.7212	-2.0601	-0.8099
H	4.9466	-0.0433	2.0404
H	4.6737	1.2573	0.8704
H	2.1216	-3.1183	-0.6149
H	2.0907	-1.9212	-1.9190
H	-1.7909	-2.8693	1.6744
C	1.1359	2.3340	-2.1259
O	2.0683	0.7048	-0.0938
Si	2.6126	-0.8379	0.2747
C	1.8130	-2.1003	-0.8754
C	2.1759	-1.2769	2.0555
C	4.4916	-0.7498	0.0111
C	5.1050	0.2596	0.9998
C	5.1170	-2.1384	0.2417
C	4.7858	-0.2864	-1.4283
H	2.6174	-0.5693	2.7636
H	2.5365	-2.2794	2.3096
H	4.9351	-2.5043	1.2584
H	0.1851	-0.2134	-2.3580
H	-0.9623	-1.6309	0.7199
H	0.1133	0.2276	0.3907
H	1.1580	1.5588	2.1131

H	0.5878	2.3051	3.2949
H	1.7274	1.1783	2.5425
H	0.0027	0.7888	2.5889
H	1.3407	4.0631	0.0736
N	-3.2431	-0.3825	-0.0716
C	-3.8743	0.8731	0.3896
C	-3.6027	1.1099	1.8796
H	-1.8299	-2.7132	0.7386
H	-2.2368	-1.6591	2.1074
H	-3.0873	-3.1995	1.8939
C	-2.6454	-2.3497	1.3640
C	-5.3679	0.9078	0.0503
H	-5.7872	1.8806	0.3243
H	-5.5213	0.7553	-1.0215
H	-5.9327	0.1423	0.5914
C	-3.7147	-1.6594	0.5112
H	-4.5285	-1.3718	1.1805
C	-4.3039	-2.5870	-0.5558
H	-3.5311	-2.8982	-1.2603
H	-5.1010	-2.0812	-1.1082
H	-4.7259	-3.4790	-0.0824
H	-0.3422	2.8459	-1.2119
H	1.1048	2.7963	-3.1939
H	1.9705	1.3733	-2.5842
H	2.1324	2.9174	-1.7566

H	0.7544	4.0213	0.3381
H	2.3237	3.2784	0.7075
H	1.3447	3.9918	2.0010
H	-1.3004	1.2737	1.9851
N	-3.4370	-0.4343	-0.1160
C	-3.0959	-1.8082	0.3145
C	-1.8845	-1.8640	1.2518
H	-4.7366	-0.6803	2.3613
H	-3.9284	0.8914	2.2022
H	-5.6982	0.7848	2.1489
C	-4.7700	0.2879	1.8510
C	-2.9801	-2.7864	-0.8598
H	-2.8747	-3.8074	-0.4797
H	-3.8772	-2.7440	-1.4839
H	-2.1130	-2.5574	-1.4808
C	-4.7252	0.1374	0.3272
H	-4.7506	1.1331	-0.1148
C	-5.9144	-0.6577	-0.2205
H	-5.8625	-0.7251	-1.3106
H	-6.8505	-0.1596	0.0496
H	-5.9538	-1.6736	0.1860
H	-0.6755	2.3509	-0.9697
H	0.6424	2.7166	-3.0255
H	1.9225	1.6393	-2.4299
H	1.6154	3.1747	-1.6233

Coordinates of **10-c18** (7.40 kJ/mol).

Atom	Coordinates x / A	y / A	z / A
H	4.5306	0.9666	-1.3307
H	4.7121	-0.6180	-2.0995
H	4.3708	-0.3066	2.2267
H	4.4165	1.1469	1.2171
H	1.3904	2.5660	3.2823
H	2.4558	1.5983	2.2472
H	5.8670	0.1465	1.3990
H	-3.8425	1.3190	-0.8920
H	-0.8477	1.3405	-3.0617
H	1.9920	-2.7804	1.3791
H	4.5925	-2.6370	1.1024
C	-2.0742	-0.4575	-0.7495
O	-1.9072	0.6887	-1.4638
C	-0.6207	0.9273	-2.0755
C	0.1780	1.9303	-1.2447
C	0.6719	1.3227	0.0952
C	0.6705	2.3531	1.2435
C	-0.7698	2.7157	1.6359
C	1.4149	1.8271	2.4746
O	-1.2128	-1.3328	-0.6778
H	-3.6478	1.5922	1.5548
H	2.4807	-2.6810	-1.7186
H	6.0475	-2.0903	0.2599
H	4.6966	-2.7614	-0.6604
H	2.2685	-1.1314	-2.5455
H	0.9062	-1.8711	-1.6741
H	-5.2616	2.0543	0.9828
C	1.3232	2.5181	-2.0770
O	1.9830	0.7893	-0.0840
Si	2.4696	-0.8147	-0.0694
C	1.9864	-1.7051	-1.6569
C	1.7051	-1.7235	1.3906
C	4.3680	-0.7122	0.0676
C	4.9515	-2.1319	0.1991
C	4.9379	-0.0408	-1.1966
C	4.7732	0.1163	1.2995
H	0.6142	-1.6786	1.3198
H	2.0089	-1.2973	2.3508
H	6.0310	0.0461	-1.1265
H	-0.0926	-0.0179	-2.1957
H	-5.0326	0.5139	1.8137
H	-0.0071	0.5157	0.3938
H	1.1824	3.2593	0.8891

H	-1.2945	1.8256	2.0041
H	-1.3521	3.1218	0.8046
H	-0.7749	3.4592	2.4388
H	0.9413	0.9111	2.8472
N	-3.2832	-0.5126	-0.1329
C	-4.2898	0.5647	-0.2490
C	-4.5719	1.2143	1.1101
H	-1.6704	-2.0719	1.5999
H	-2.6553	-0.8829	2.4762
H	-3.0505	-2.6088	2.5775
C	-2.6842	-1.8202	1.9127
C	-5.5658	0.0593	-0.9312
H	-5.3337	-0.3741	-1.9079
H	-6.0793	-0.6988	-0.3315
H	-6.2624	0.8902	-1.0786
C	-3.6117	-1.6923	0.6996
H	-4.6152	-1.4887	1.0796
C	-3.6881	-2.9806	-0.1257
H	-2.7085	-3.2335	-0.5331
H	-4.3944	-2.8664	-0.9529
H	-4.0303	-3.8062	0.5062
H	-0.5177	2.7429	-1.0107
H	1.9891	1.7335	-2.4433
H	1.9271	3.2073	-1.4811
H	0.9264	3.0684	-2.9361

Coordinates of **12-c4** (5.52 kJ/mol).

Atom	Coordinates x / A	y / A	z / A
H	6.0486	-0.5260	0.4456
H	4.7703	-0.5274	1.6666
H	4.3142	-3.0127	1.0698
H	5.5943	-2.9243	-0.1457
H	2.1235	3.8846	-0.8765
H	3.4168	2.7616	-0.4336
H	3.9781	-3.4681	-0.6082
H	-3.9124	-1.3070	1.7441
H	-1.1541	1.0275	-2.5888
H	2.4196	-0.8608	2.4991
H	5.5614	-1.1166	-1.9491
C	-2.8015	0.1464	-1.0416
O	-1.4657	0.0616	-0.7836
C	-0.5747	0.7144	-1.7211
C	0.1089	1.9072	-1.0607
C	1.0076	1.4612	0.1110
C	1.6815	2.6261	0.8631
C	2.3899	2.1335	2.1295
C	2.6405	3.4190	-0.0319
O	-3.2533	0.6664	-2.0573
H	-3.0662	0.9532	2.2475
H	0.2343	-2.0513	-1.0302
H	4.2131	0.0301	-2.0372
H	3.9609	-1.6723	-2.4528
H	1.5365	-3.2346	-0.8884
H	1.5705	-2.0355	-2.1871
H	-2.1188	-0.3023	3.0666
C	-0.9272	2.9628	-0.6490
O	2.0103	0.5723	-0.3905
Si	2.2857	-1.0363	-0.0029
C	1.3133	-2.1908	-1.1346
C	1.7995	-1.4028	1.7806
C	4.1500	-1.2980	-0.2956
C	4.4846	-0.9959	-1.7691
C	4.9744	-0.3593	0.6034
C	4.5221	-2.7587	0.0244
H	1.9058	-2.4744	1.9802
H	0.7548	-1.1397	1.9737
H	4.7645	0.6909	0.3812
H	0.1551	-0.0347	-2.0220
H	-1.4675	0.5039	1.6302
H	0.3739	0.9294	0.8324
H	0.8742	3.2938	1.1867

Coordinates of **12-c5** (6.03 kJ/mol).

Atom	Coordinates x / A	y / A	z / A
H	1.7154	-4.0960	1.1655
H	3.2943	-3.9332	0.3817
H	-0.1851	-1.9294	-1.3193
H	-0.3522	-2.9715	0.0917
H	3.5869	3.0575	-1.7201
H	4.3836	1.6364	-1.0260
H	0.0928	-3.6607	-1.4724
H	-1.8071	1.5282	1.2034
H	-0.5564	1.2352	-2.7013
H	3.7570	-0.6831	2.4410
H	2.2170	-1.5511	-2.3298
C	-2.1076	0.1402	-0.8749
O	-1.0389	0.9647	-0.7007
C	-0.0646	0.9570	-1.7651
C	1.0291	1.9502	-1.4041
C	1.7595	1.5365	-0.1086
C	2.8923	2.5052	0.2877
C	3.5176	2.1194	1.6317
C	3.9732	2.6235	-0.7931
O	-2.2355	-0.5591	-1.8761
H	-2.1679	-0.4288	2.6955
H	0.3837	-1.8177	2.3952
H	2.3873	-3.3123	-2.4552
H	3.6093	-2.3574	-1.6027
H	-0.3187	-0.6353	1.2802
H	0.7733	-0.1025	2.5685
H	-2.6299	1.1040	3.4549
C	0.4715	3.3786	-1.3363
O	2.2713	0.2116	-0.2816
Si	2.0448	-1.1483	0.6803
C	0.5830	-0.8956	1.8382
C	3.5967	-1.4717	1.7017
C	1.7401	-2.6377	-0.4853
C	2.5335	-2.4489	-1.7920
C	2.2150	-3.9342	0.2029
C	0.2422	-2.7961	-0.8113
H	4.4893	-1.5285	1.0697
H	3.5099	-2.4194	2.2422
H	1.9857	-4.8006	-0.4316
H	0.3428	-0.0468	-1.8756
H	-3.8792	0.0072	2.8694
H	1.0206	1.5404	0.7039
H	2.4205	3.4863	0.4204

H	2.7844	2.9784	2.7030
H	3.2278	1.4775	1.8837
H	1.7033	1.5784	2.7773
H	3.1287	4.2157	0.5381
N	-3.5562	-0.4177	-0.0565
C	-3.0236	-0.9592	1.2128
C	-2.3777	0.1185	2.0898
H	-5.3207	1.4881	0.7546
H	-6.7950	0.5197	0.5683
H	-5.6106	0.0865	1.8036
C	-5.7235	0.4719	0.7849
C	-2.1225	-2.1827	1.0192
H	-1.1674	-1.9043	0.5761
H	-1.9284	-2.6533	1.9883
H	-2.6060	-2.9188	0.3710
C	-5.0218	-0.4164	-0.2475
H	-5.1713	0.0256	-1.2321
C	-5.5832	-1.8414	-0.2722
H	-5.4729	-2.3460	0.6932
H	-5.0769	-2.4414	-1.0334
H	-6.6513	-1.8151	-0.5081
H	0.7646	2.3293	-1.8332
H	-1.4905	2.6393	0.2323
H	-1.6465	3.1248	-1.4565
H	-0.4602	3.9221	-0.4153

H	4.1289	1.2205	1.5325
H	2.7522	1.9293	2.3929
H	4.1645	2.9222	1.9994
H	4.7939	3.2602	-0.4478
N	-2.9745	0.1784	0.1737
C	-2.8096	1.1175	1.3048
C	-2.8799	0.3987	2.6567
H	-3.3440	-2.4323	-0.7506
H	-3.2920	-2.3773	1.0231
H	-4.8235	-2.7118	0.1920
C	-3.8947	-2.1348	0.1429
C	-3.8115	2.2736	1.2112
H	-3.7056	2.7964	0.2565
H	-4.8437	1.9188	1.2967
H	-3.6384	2.9906	2.0197
C	-4.2094	-0.6359	0.1050
H	-4.7538	-0.3907	1.0195
C	-5.1089	-0.2477	-1.0740
H	-5.3321	0.8229	-1.0524
H	-6.0545	-0.7954	-1.0113
H	-4.6271	-0.4855	-2.0226
H	1.7534	1.8877	-2.2257
H	-0.1383	3.5900	-2.2203
H	1.2666	4.1264	-1.2972
H	-0.1651	3.5102	-0.4558

Coordinates of **12-c6** (6.42 kJ/mol).

Atom	Coordinates x / A	y / A	z / A
H	3.0164	1.9291	-2.1660
H	2.5725	3.3929	-1.2795
H	3.0635	2.0291	2.1845
H	1.5245	2.0566	1.3088
H	1.3081	-1.6622	3.0492
H	-0.0951	-0.6903	3.5141
H	2.6101	3.4533	1.2405
H	-3.5291	2.3806	0.4024
H	0.4126	-0.4552	-2.3045
H	5.0628	-0.6861	1.5655
H	4.7931	3.3318	-0.0203
C	-2.4720	-0.5141	-0.8585
O	-1.2392	-0.0975	-1.2377
C	-0.4018	-1.0598	-1.9048
C	0.1600	-2.1675	-1.0096
C	0.8221	-1.6439	0.2872
C	-0.1540	-1.3809	1.4559
C	-0.9961	-2.6187	1.7974
C	0.6038	-0.8985	2.6974
O	-2.8863	-1.6514	-1.0687
H	-1.7001	2.1179	-2.0203
H	3.6184	-0.5367	-2.3919
H	5.2623	1.8753	-0.9038
H	5.2877	1.8853	0.8668
H	4.0762	-1.9426	-1.4180
H	5.1219	-0.5220	-1.4585
H	-3.4483	2.4214	-2.0692
C	1.1380	-2.9946	-1.8555
O	1.5397	-0.4426	-0.0015
Si	3.1808	-0.1248	0.0454
C	4.0763	-0.8486	-1.4481
C	3.9789	-0.8415	1.5944
C	3.2661	1.7760	0.0114
C	4.7362	2.2351	-0.0132
C	2.5445	2.2952	-1.2474
C	2.5751	2.3555	1.2598
H	3.8057	-1.9210	1.6605
H	3.5938	-0.3864	2.5102
H	1.4953	1.9852	-1.2617
H	-0.9621	-1.5087	-2.7292
H	-2.3598	3.6146	-1.3369
H	1.5230	-2.4218	0.6324
H	-0.8282	-0.5800	1.1473

Coordinates of **12-c7** (7.16 kJ/mol).

Atom	Coordinates x / A	y / A	z / A
H	3.6000	-0.7410	-2.1707
H	2.8596	-2.3482	-2.2065
H	4.8948	-0.9733	1.3851
H	4.8152	0.0703	-0.0425
H	1.5761	2.1796	2.6177
H	2.7749	3.4424	2.3037
H	5.8026	-1.4026	-0.0703
H	-4.0149	-0.8300	1.7230
H	-1.1797	3.1224	-1.0486
H	1.0414	-0.4718	2.5935
H	4.6900	-3.6964	-0.1008
C	-2.3510	0.1379	-0.9936
O	-1.8732	1.3141	-0.5010
C	-1.0716	2.1006	-1.4085
C	0.3833	1.6289	-1.4375
C	1.0633	1.5370	-0.0505
C	1.6597	2.8294	0.5397
C	0.6282	3.9543	0.6994
C	2.3191	2.5355	1.8936
O	-2.1443	-0.2307	-2.1456
H	-4.5825	1.4710	2.2645
H	0.5359	-2.9269	-0.0277
H	2.9294	-3.8474	-0.1101
H	3.7640	-3.2898	1.3491
H	0.4683	-1.8229	-1.4119
H	-0.3878	-1.4291	0.0794
H	-3.6685	2.0747	0.8698
C	1.1939	2.4326	-2.4587
O	2.1336	0.5892	-0.1733
Si	2.0440	-0.9943	0.3723
C	0.5284	-1.8712	-0.3215
C	1.9162	-1.0354	2.2528
C	3.6606	-1.7794	-0.2428
C	3.7600	-3.2332	0.2551
C	3.6818	-1.7613	-1.7830
C	4.8588	-0.9712	0.2900
H	2.7980	-0.6032	2.7342
H	1.8033	-2.0649	2.6095
H	4.6207	-2.1903	-2.1586
H	-1.4967	2.0277	-2.4114
H	-5.1205	1.0866	0.6197
H	0.3159	1.1618	0.6626
H	2.4435	3.1676	-0.1498

H	-1.7216	-2.8522	1.0158
H	-1.5559	-2.4462	2.7225
H	-0.3616	-3.4992	1.9587
H	1.1661	0.0138	2.4940
N	-3.1604	0.4674	-0.2102
C	-2.7034	1.8727	-0.1018
C	-2.5409	2.5435	-1.4707
H	-5.5829	0.6662	-1.4386
H	-6.5826	0.5043	0.0177
H	-5.5695	1.9339	-0.1974
C	-5.6170	0.8524	-0.3616
C	-1.4665	2.0450	0.7871
H	-0.5910	1.5708	0.3434
H	-1.2576	3.1115	0.9197
H	-1.6359	1.6069	1.7748
C	-4.4793	0.1117	0.3482
H	-4.5876	-0.9529	0.1408
C	-4.5047	0.3100	1.8671
H	-5.4587	-0.0394	2.2735
H	-4.3935	1.3629	2.1463
H	-3.6988	-0.2579	2.3408
H	-0.6673	-2.8199	-0.7285
H	1.5404	-3.8306	-1.2754
H	1.9786	-2.3873	-2.2010
H	0.6412	-3.4107	-2.7380

H	0.2903	4.3551	-0.2587
H	1.0639	4.7859	1.2620
H	-0.2525	3.6086	1.2535
H	3.0930	1.7725	1.8004
N	-3.0584	-0.5588	-0.0596
C	-3.3900	-0.0461	1.2891
C	-4.2406	1.2289	1.2532
H	-5.6408	-1.5734	0.3794
H	-5.4606	-0.9512	-1.2727
H	-5.5754	-2.6972	-0.9814
C	-5.1716	-1.7574	-0.5928
C	-2.1604	0.0895	2.1941
H	-2.4785	0.2963	3.2208
H	-1.5788	-0.8366	2.1968
H	-1.5162	0.9054	1.8640
C	-3.6461	-1.8476	-0.4824
H	-3.2416	-2.0197	-1.4797
C	-3.1934	-2.9978	0.4219
H	-3.5678	-2.8931	1.4454
H	-2.1029	-3.0554	0.4572
H	-3.5756	-3.9450	0.0297
H	0.3421	0.5952	-1.7936
H	2.2288	2.0815	-2.4739
H	1.2021	3.5032	-2.2305
H	0.7797	2.3098	-3.4642

Coordinates of **12-c8** (7.24 kJ/mol).

Atom	Coordinates x / A	y / A	z / A
H	5.0549	-3.2475	0.4482
H	3.5547	-3.5152	-0.4470
H	4.2133	-1.5660	-2.0007
H	5.6829	-1.2896	-1.0576
H	0.8034	1.7921	2.6582
H	1.8936	3.1799	2.7837
H	4.5378	0.0369	-1.3227
H	-2.1250	-1.3533	-1.7859
H	-1.4821	2.7390	-1.0990
H	0.0549	-1.0540	1.5288
H	5.5900	-0.9708	1.4442
C	-2.4047	0.6214	-0.2494
O	-1.7266	0.7070	-1.4300
C	-1.0640	1.9573	-1.7301
C	0.4488	1.7899	-1.5730
C	0.8942	1.5378	-0.1112
C	1.2137	2.7693	0.7573
C	0.0458	3.7599	0.8592
C	1.6300	2.3169	2.1637
O	-2.5115	1.5674	0.5269
H	-4.0048	-2.8236	-2.4312
H	1.7666	-1.5496	-2.3771
H	4.0445	-0.9452	2.3031
H	4.4771	0.3911	1.2253
H	0.2035	-1.4008	-1.5664
H	1.1854	-2.8506	-1.3242
H	-4.8680	-2.4222	-0.9432
C	1.2249	2.9080	-2.2770
O	2.0788	0.7266	-0.1510
Si	2.0517	-0.9400	0.0346
C	1.2285	-1.7634	-1.4484
C	1.0916	-1.3987	1.5907
C	3.8829	-1.4303	0.1665
C	4.5308	-0.6941	1.3541
C	3.9995	-2.9513	0.3804
C	4.6162	-1.0376	-1.1300
H	1.5353	-0.9531	2.4857
H	1.0682	-2.4853	1.7262
H	3.5100	-3.2716	1.3066
H	-1.3073	2.1687	-2.7749
H	-4.5589	-1.1610	-2.1518
H	0.0927	0.9871	0.3974
H	2.0720	3.2747	0.2955

Coordinates of **12-c9** (7.24 kJ/mol).

Atom	Coordinates x / A	y / A	z / A
H	3.0741	2.0529	2.1949
H	1.5069	2.0328	1.3699
H	5.1765	1.9243	-0.9592
H	5.2576	1.9396	0.8099
H	1.2829	-1.7143	3.0361
H	-0.1336	-0.7674	3.5082
H	4.7054	3.3723	-0.0638
H	-3.4859	2.3847	0.4299
H	0.4455	-0.4441	-2.3136
H	5.0434	-0.6331	1.6049
H	2.4389	3.3767	-1.2593
C	-2.4398	-0.4998	-0.8710
O	-1.2075	-0.0778	-1.2533
C	-0.3784	-1.0428	-1.9243
C	0.1639	-2.1673	-1.0373
C	0.8129	-1.6640	0.2740
C	-0.1738	-1.4247	1.4385
C	-1.0108	-2.6730	1.7531
C	0.5715	-0.9538	2.6917
O	-2.8519	-1.6348	-1.0814
H	-3.4056	2.4684	-2.0406
H	4.0780	-1.9289	-1.3966
H	1.4016	1.9393	-1.2043
H	2.8974	1.9216	-2.1514
H	5.1221	-0.5079	-1.4182
H	3.6310	-0.5199	-2.3696
H	-2.3035	3.6393	-1.2922
C	1.1464	-2.9906	-1.8814
O	1.5270	-0.4552	0.0119
Si	3.1612	-0.1158	0.0640
C	4.0765	-0.8348	-1.4209
C	3.9633	-0.8153	1.6196
C	3.2127	1.7853	0.0185
C	2.4413	2.2790	-1.2213
C	2.5472	2.3577	1.2839
C	4.6710	2.2749	-0.0531
H	3.8177	-1.8991	1.6827
H	3.5564	-0.3733	2.5322
H	2.5524	3.4557	1.2549
H	-0.9410	-1.4792	-2.7541
H	-1.6613	2.1459	-1.9989
H	1.5150	-2.4442	0.6132
H	-0.8506	-0.6236	1.1363

H	-0.1830	4.2400	-0.0955
H	0.2932	4.5547	1.5704
H	-0.8614	3.2572	1.2053
H	2.4874	1.6429	2.1281
N	-2.9319	-0.6143	-0.0397
C	-2.7956	-1.7200	-1.0119
C	-4.1400	-2.0489	-1.6704
H	-5.5834	-0.0757	0.2867
H	-5.6369	-0.2475	2.0505
H	-4.7305	1.0922	1.3164
C	-5.0031	0.0422	1.2065
C	-2.1447	-2.9509	-0.3714
H	-1.9545	-3.7064	-1.1395
H	-1.1923	-2.6883	0.0939
H	-2.7854	-3.4087	0.3884
C	-3.7533	-0.8438	1.1720
H	-4.0940	-1.8777	1.0819
C	-2.9196	-0.7452	2.4532
H	-3.5396	-0.9880	3.3219
H	-2.5250	0.2644	2.5757
H	-2.0829	-1.4490	2.4238
H	0.6869	0.8598	-2.0989
H	2.2997	2.7452	-2.1611
H	0.9887	3.8987	-1.8793
H	0.9992	2.9173	-3.3481

H	-1.5758	-2.5206	2.6787
H	-0.3726	-3.5528	1.9034
H	-1.7316	-2.8955	0.9639
H	1.1231	-0.0312	2.5062
N	-3.1280	0.4808	-0.2149
C	-2.6634	1.8798	-0.0837
C	-2.4964	2.5721	-1.4416
H	-6.5490	0.5238	0.0285
H	-5.5323	1.9542	-0.1598
H	-5.5545	0.7127	-1.4274
C	-5.5832	0.8762	-0.3467
C	-1.4238	2.0283	0.8064
H	-0.5555	1.5463	0.3574
H	-1.1989	3.0903	0.9498
H	-1.5980	1.5824	1.7898
C	-4.4443	0.1164	0.3407
H	-4.5553	-0.9429	0.1074
C	-4.4628	0.2793	1.8640
H	-5.4169	-0.0724	2.2685
H	-4.3423	1.3247	2.1675
H	-3.6587	-0.3050	2.3202
H	-0.6735	-2.8151	-0.7771
H	0.6584	-3.3901	-2.7764
H	1.5330	-3.8394	-1.3090
H	1.9976	-2.3870	-2.2070

Coordinates of **9-c4** (5.88 kJ/mol).

Atom	Coordinates x / A	y / A	z / A
C	1.2882	1.3495	2.2439
H	0.9660	2.0623	3.0056
H	1.7826	0.5002	2.7150
C	2.2271	2.0250	1.2367
H	1.7471	2.9543	0.9131
C	3.5289	2.3596	1.9751
H	3.3143	2.7870	2.9607
H	4.1304	1.4579	2.1187
H	4.1265	3.0902	1.4281
C	2.3926	1.1377	-0.0124
H	1.3848	1.0197	-0.4271
C	3.2483	1.6865	-1.1764
H	3.0253	1.0043	-2.0092
C	2.7985	3.0894	-1.5992
H	3.0561	3.8372	-0.8419
H	3.2886	3.3844	-2.5323
H	1.7168	3.1366	-1.7620
C	4.7645	1.6210	-0.9562
H	5.0531	0.6562	-0.5337
H	5.2853	1.7479	-1.9111
H	5.1195	2.4067	-0.2850
O	2.8866	-0.1411	0.3815
Si	2.1600	-1.6274	0.0848
C	0.7263	-1.4478	-1.1223
H	0.3462	-2.4366	-1.4007
H	-0.1014	-0.8892	-0.6778
H	1.0283	-0.9389	-2.0431
C	1.5367	-2.3282	1.7159
H	1.0902	-3.3187	1.5784
H	2.3423	-2.4219	2.4510
H	0.7703	-1.6674	2.1296
C	3.5257	-2.7387	-0.6425
C	3.0037	-4.1789	-0.8012
H	2.7140	-4.6159	0.1603
H	3.7821	-4.8226	-1.2327
H	2.1348	-4.2279	-1.4672
C	3.9540	-2.1999	-2.0201
H	4.3257	-1.1730	-1.9524
H	4.7591	-2.8178	-2.4406
H	3.1247	-2.2109	-2.7357
C	4.7431	-2.7386	0.3011
H	5.5498	-3.3597	-0.1118
H	5.1372	-1.7277	0.4441

Coordinates of **9-c5** (6.26 kJ/mol).

Atom	Coordinates x / A	y / A	z / A
C	-1.7412	-0.8887	2.2977
H	-1.5346	-1.5394	3.1497
H	-2.1587	0.0522	2.6495
C	-2.6856	-1.5809	1.3153
H	-2.2668	-2.5741	1.1275
C	-4.0570	-1.7323	1.9824
H	-3.9583	-2.1610	2.9849
H	-4.5470	-0.7586	2.0716
H	-4.7119	-2.3907	1.4067
C	-2.7486	-0.8573	-0.0462
H	-1.7296	-0.8178	-0.4461
C	-3.6301	-1.5883	-1.0796
H	-4.6600	-1.5641	-0.7020
C	-3.6104	-0.8576	-2.4253
H	-3.9640	0.1693	-2.3204
H	-2.5956	-0.8319	-2.8407
H	-4.2525	-1.3667	-3.1510
C	-3.2098	-3.0527	-1.2666
H	-3.3682	-3.6548	-0.3684
H	-3.7864	-3.5108	-2.0761
H	-2.1503	-3.1255	-1.5373
O	-3.2522	0.4672	0.1163
Si	-2.6315	2.0243	0.0925
C	-1.9531	2.4939	1.7890
H	-1.0269	1.9652	2.0261
H	-1.7499	3.5687	1.8309
H	-2.6875	2.2692	2.5696
C	-4.1561	3.0768	-0.2379
H	-4.6435	2.7897	-1.1745
H	-4.8844	2.9546	0.5705
H	-3.9021	4.1402	-0.3010
C	-1.2989	2.3897	-1.2416
C	-1.9341	2.5322	-2.6376
H	-2.6767	3.3359	-2.6682
H	-2.4237	1.6107	-2.9617
H	-1.1598	2.7716	-3.3793
C	-0.6402	3.7375	-0.8716
H	0.0621	4.0355	-1.6615
H	-1.3734	4.5453	-0.7655
H	-0.0738	3.6719	0.0621
C	-0.1978	1.3173	-1.2935
H	0.2423	1.1239	-0.3133
H	-0.5774	0.3674	-1.6785

H	4.4911	-3.1425	1.2879
O	0.1201	0.7694	1.6139
C	-0.8823	1.6045	1.2668
O	-0.8827	2.7939	1.5202
C	-3.8907	-0.4810	-0.9894
C	-3.7043	-0.7577	0.3671
C	-2.7372	-0.0989	1.1290
C	-1.9438	0.8783	0.4992
C	-2.1093	1.1869	-0.8658
C	-3.0868	0.4933	-1.5839
H	-4.3275	-1.5065	0.8458
H	-3.2254	0.7100	-2.6384
C	-2.6134	-0.4112	2.6143
H	-1.7534	0.1340	3.0114
C	-1.2655	2.2542	-1.5507
H	-0.3648	2.4103	-0.9500
C	-4.9314	-1.2288	-1.8043
H	-4.9054	-0.8157	-2.8203
C	-4.5909	-2.7257	-1.8987
H	-5.3141	-3.2474	-2.5340
H	-3.5918	-2.8761	-2.3186
H	-4.6129	-3.1957	-0.9097
C	-6.3490	-1.0170	-1.2489
H	-6.5990	0.0470	-1.2014
H	-7.0891	-1.5166	-1.8825
H	-6.4430	-1.4291	-0.2386
C	-3.8594	0.0758	3.3742
H	-4.0175	1.1476	3.2218
H	-4.7580	-0.4486	3.0329
H	-3.7498	-0.1066	4.4483
C	-2.3565	-1.9052	2.8660
H	-2.2015	-2.0898	3.9339
H	-3.2057	-2.5168	2.5445
H	-1.4704	-2.2493	2.3264
C	-2.0214	3.5938	-1.5789
H	-1.4086	4.3717	-2.0466
H	-2.9504	3.5010	-2.1518
H	-2.2701	3.9121	-0.5638
C	-0.8010	1.8444	-2.9553
H	-0.1005	2.5879	-3.3483
H	-0.2976	0.8736	-2.9399
H	-1.6361	1.7818	-3.6597

H	0.6138	1.6345	-1.9626
O	-0.4887	-0.5170	1.6722
C	0.4376	-1.4884	1.5150
O	0.2965	-2.6214	1.9342
C	3.7165	-0.0082	-0.8368
C	3.4936	0.4801	0.4522
C	2.4454	0.0118	1.2473
C	1.6073	-0.9901	0.7246
C	1.8108	-1.5141	-0.5680
C	2.8699	-1.0061	-1.3239
H	4.1477	1.2506	0.8482
H	3.0375	-1.3850	-2.3267
C	2.2683	0.5707	2.6527
H	1.3755	0.1210	3.0945
C	0.9138	-2.6078	-1.1337
H	-0.0490	-2.5548	-0.6159
C	4.8343	0.5473	-1.7015
H	4.8290	-0.0189	-2.6412
C	4.5814	2.0255	-2.0437
H	5.3581	2.4061	-2.7151
H	3.6103	2.1554	-2.5309
H	4.5869	2.6429	-1.1392
C	6.2125	0.3571	-1.0480
H	6.2870	0.9190	-0.1111
H	6.3998	-0.6970	-0.8224
H	7.0060	0.7127	-1.7133
C	3.4627	0.1939	3.5458
H	4.3918	0.6344	3.1697
H	3.3075	0.5576	4.5668
H	3.5967	-0.8912	3.5841
C	2.0477	2.0918	2.6312
H	1.1980	2.3575	1.9967
H	1.8511	2.4644	3.6416
H	2.9287	2.6167	2.2481
C	1.5115	-3.9939	-0.8339
H	1.6381	-4.1302	0.2421
H	0.8548	-4.7855	-1.2105
H	2.4885	-4.1026	-1.3172
C	0.6256	-2.4451	-2.6326
H	0.2509	-1.4438	-2.8639
H	1.5180	-2.6210	-3.2412
H	-0.1280	-3.1736	-2.9473

Coordinates of **11-c4** (3.77 kJ/mol).

Atom	Coordinates x / A	y / A	z / A
C	-1.9849	0.4551	0.6084
C	-3.1564	1.2188	0.4539
C	-4.3355	0.5513	0.1114
C	-4.3701	-0.8306	-0.0870
C	-3.1867	-1.5571	0.0705
C	-1.9831	-0.9400	0.4186
C	-3.1465	2.7158	0.7392
H	-5.2539	1.1162	-0.0085
C	-5.6662	-1.5243	-0.4699
H	-3.2016	-2.6304	-0.0846
C	-0.7172	-1.7629	0.6262
C	-0.7009	1.1213	1.0018
O	-0.1780	1.8171	-0.0259
C	1.1362	2.3944	0.1558
C	1.9047	2.3184	-1.1570
C	2.0704	0.8678	-1.6755
C	0.8515	0.2624	-2.4073
C	0.2885	1.2003	-3.4829
C	1.2327	-1.0852	-3.0292
O	-0.1806	1.0371	2.0988
C	-6.0918	-2.5526	0.5909
C	-5.5654	-2.1739	-1.8601
C	-0.6534	-2.2664	2.0795
C	-0.5887	-2.9300	-0.3620
C	-3.2680	2.9596	2.2565
C	-4.2201	3.5003	-0.0243
C	3.2656	3.0018	-0.9620
H	1.3563	2.8870	-1.9134
O	2.4221	0.0256	-0.5750
Si	3.8359	-0.8232	-0.2681
C	5.2723	-0.1182	-1.2637
C	3.6250	-2.6367	-0.7377
C	4.1391	-0.6893	1.6091
C	4.4696	0.7588	2.0127
C	2.8773	-1.1459	2.3662
C	5.3245	-1.5942	2.0005
H	-2.1749	3.1040	0.4161
H	-6.4447	-0.7526	-0.5179
H	0.1474	-1.1113	0.4613
H	1.0055	3.4342	0.4713
H	1.6495	1.8467	0.9438
H	2.8955	0.8881	-2.4045
H	0.0702	0.0897	-1.6633

Coordinates of **11-c5** (4.40 kJ/mol).

Atom	Coordinates x / A	y / A	z / A
C	2.0628	0.3930	-0.5642
C	2.0873	-0.9762	-0.2402
C	3.3057	-1.5411	0.1435
C	4.4802	-0.7871	0.2083
C	4.4203	0.5687	-0.1206
C	3.2260	1.1840	-0.5049
C	0.8317	-1.8338	-0.3356
H	3.3397	-2.5941	0.4008
C	5.7935	-1.4232	0.6304
H	5.3317	1.1551	-0.0726
C	3.1931	2.6457	-0.9354
C	0.7694	1.0028	-1.0152
O	0.2531	1.8111	-0.0693
C	-1.0120	2.4575	-0.3527
C	-1.8394	2.5466	0.9227
C	-2.2575	1.1568	1.4666
C	-1.1717	0.3947	2.2590
C	-0.5676	1.2431	3.3856
C	-1.7458	-0.9068	2.8300
O	0.2445	0.7945	-2.0935
C	6.2192	-2.5369	-0.3405
C	5.7260	-1.9455	2.0752
C	4.2641	3.5144	-0.2646
C	3.2962	2.7412	-2.4706
C	0.7171	-2.8523	0.8072
C	0.7665	-2.5271	-1.7080
C	-3.0571	3.4369	0.6377
H	-1.2402	3.0439	1.6910
O	-2.6834	0.3397	0.3760
Si	-4.1998	-0.2652	-0.0022
C	-5.3049	1.0396	-0.7966
C	-5.0796	-0.9014	1.5380
C	-3.8317	-1.6584	-1.2436
C	-3.0483	-1.0862	-2.4414
C	-2.9830	-2.7463	-0.5622
C	-5.1498	-2.2813	-1.7407
H	-0.0382	-1.1722	-0.2646
H	6.5597	-0.6386	0.5947
H	2.2204	3.0523	-0.6405
H	-0.7865	3.4572	-0.7374
H	-1.5362	1.8892	-1.1190
H	-3.0971	1.3238	2.1606
H	-0.3719	0.1399	1.5590

H	-0.1940	2.0799	-3.0505
H	-0.4632	0.6763	-4.0813
H	1.0747	1.5418	-4.1680
H	1.5599	-1.7949	-2.2701
H	2.0436	-0.9646	-3.7592
H	0.3779	-1.5243	-3.5535
H	-7.0552	-3.0019	0.3282
H	-6.1869	-2.0860	1.5759
H	-5.3576	-3.3610	0.6727
H	-4.8077	-2.9646	-1.8702
H	-5.2898	-1.4367	-2.6202
H	-6.5218	-2.6233	-2.1471
H	0.2575	-2.8503	2.2455
H	-1.5147	-2.9067	2.2998
H	-0.6572	-1.4262	2.7770
H	-0.7037	-2.5948	-1.3961
H	-1.3329	-3.7101	-0.1719
H	0.3975	-3.3936	-0.2629
H	-2.4694	2.4526	2.8043
H	-4.2266	2.5807	2.6270
H	-3.2140	4.0301	2.4811
H	-5.2278	3.2621	0.3313
H	-4.1809	3.2938	-1.0981
H	-4.0704	4.5741	0.1232
H	3.8268	3.0162	-1.9006
H	3.8692	2.4804	-0.2154
H	3.1434	4.0370	-0.6280
H	5.4687	0.9331	-1.0388
H	5.0859	-0.2026	-2.3395
H	6.1843	-0.6849	-1.0472
H	3.5159	-2.7574	-1.8192
H	2.7438	-3.0760	-0.2599
H	4.4990	-3.2185	-0.4273
H	3.6409	1.4381	1.7953
H	5.3603	1.1352	1.4977
H	4.6647	0.8174	3.0918
H	2.0170	-0.5096	2.1418
H	3.0480	-1.1066	3.4506
H	2.6078	-2.1779	2.1166
H	5.1221	-2.6490	1.7897
H	5.5253	-1.5085	3.0767
H	6.2450	-1.3163	1.4744

H	0.0399	2.0665	3.0029
H	0.0800	0.6253	4.0153
H	-1.3491	1.6642	4.0303
H	-2.1255	-1.5568	2.0409
H	-2.5673	-0.6996	3.5266
H	-0.9755	-1.4579	3.3782
H	6.2910	-2.1601	-1.3651
H	5.4970	-3.3601	-0.3362
H	7.1937	-2.9462	-0.0548
H	4.9833	-2.7447	2.1697
H	5.4490	-1.1471	2.7701
H	6.6948	-2.3509	2.3851
H	5.2713	3.2561	-0.6073
H	4.2390	3.4131	0.8245
H	4.0977	4.5667	-0.5140
H	4.2563	2.3402	-2.8130
H	3.2253	3.7838	-2.7980
H	2.4996	2.1729	-2.9578
H	-0.2552	-3.3515	0.7692
H	0.8139	-2.3684	1.7832
H	1.4834	-3.6306	0.7362
H	-0.1489	-3.1206	-1.7964
H	1.6236	-3.1965	-1.8408
H	0.7774	-1.7862	-2.5105
H	-3.6473	3.0493	-0.1952
H	-2.7462	4.4546	0.3817
H	-3.7081	3.4959	1.5151
H	-5.5751	1.8243	-0.0838
H	-6.2359	0.5784	-1.1440
H	-4.8253	1.5138	-1.6584
H	-6.0934	-1.2280	1.2822
H	-5.1718	-0.1127	2.2924
H	-4.5574	-1.7434	1.9989
H	-3.6281	-0.3259	-2.9768
H	-2.8155	-1.8844	-3.1593
H	-2.1040	-0.6302	-2.1309
H	-2.0430	-2.3338	-0.1893
H	-2.7397	-3.5460	-1.2749
H	-3.5085	-3.2076	0.2813
H	-4.9422	-3.1017	-2.4406
H	-5.7732	-1.5522	-2.2689
H	-5.7420	-2.6963	-0.9172

Coordinates of **11-c6** (4.44 kJ/mol).

Atom	Coordinates x / A	y / A	z / A
C	2.0372	0.1885	-0.5612
C	1.9939	-1.1553	-0.1541
C	3.1853	-1.7523	0.2723
C	4.3922	-1.0543	0.2988
C	4.3996	0.2825	-0.1146
C	3.2388	0.9259	-0.5436
C	0.6989	-1.9558	-0.2090
H	3.1760	-2.7883	0.5955
C	5.6680	-1.7317	0.7691
H	5.3334	0.8330	-0.0994
C	3.2743	2.3578	-1.0646
C	0.7766	0.8331	-1.0551
O	0.2888	1.7076	-0.1542
C	-0.9432	2.3949	-0.4840
C	-1.7697	2.6005	0.7784
C	-2.2545	1.2670	1.4028
C	-1.2083	0.5102	2.2518
C	-0.5982	1.3960	3.3461
C	-1.8336	-0.7416	2.8774
O	0.2531	0.5984	-2.1286
C	6.2293	-1.0585	2.0325
C	6.7250	-1.7819	-0.3465
C	4.3898	3.2133	-0.4524
C	3.3739	2.3519	-2.6031
C	0.5368	-2.9128	0.9800
C	0.5993	-2.7088	-1.5477
C	-2.9426	3.5281	0.4317
H	-1.1501	3.1150	1.5186
O	-2.7091	0.4043	0.3598
Si	-4.2498	-0.1562	0.0114
C	-5.3055	1.1537	-0.8396
C	-5.1518	-0.6861	1.5791
C	-3.9395	-1.6183	-1.1646
C	-3.1524	-2.7161	-0.4274
C	-5.2817	-2.1970	-1.6505
C	-3.1172	-1.1368	-2.3759
H	-0.1383	-1.2503	-0.1697
H	5.4084	-2.7656	1.0292
H	2.3238	2.8288	-0.7934
H	-0.6694	3.3574	-0.9275
H	-1.4913	1.8063	-1.2176
H	-3.0908	1.5130	2.0770
H	-0.4048	0.1952	1.5810

Coordinates of **11-c7** (5.89 kJ/mol).

Atom	Coordinates x / A	y / A	z / A
C	-1.6676	-0.9000	0.6721
C	-1.7716	-1.5396	-0.5738
C	-2.8047	-1.1472	-1.4306
C	-3.7103	-0.1450	-1.0797
C	-3.5769	0.4709	0.1695
C	-2.5630	0.1139	1.0595
C	-0.8014	-2.6500	-0.9517
H	-2.9023	-1.6214	-2.4016
C	-4.8043	0.2797	-2.0441
H	-4.2737	1.2529	0.4496
C	-2.4521	0.7481	2.4394
C	-0.5479	-1.2815	1.5977
O	0.4958	-0.4538	1.4149
C	1.7029	-0.6267	2.2008
C	2.7784	-1.3739	1.4048
C	2.8592	-0.7974	-0.0242
C	4.0094	-1.3024	-0.9275
C	5.3719	-1.2805	-0.2298
C	3.7230	-2.6491	-1.6069
O	-0.5670	-2.1965	2.3984
C	-4.6128	1.7378	-2.4945
C	-6.2046	0.0639	-1.4477
C	-2.9518	2.1966	2.4969
C	-3.1812	-0.1264	3.4778
C	-0.5084	-2.7310	-2.4545
C	-1.3001	-4.0029	-0.4117
C	2.5660	-2.8932	1.4484
H	3.7215	-1.1419	1.9157
O	2.9613	0.6251	0.1115
Si	2.2719	1.7454	-0.9380
C	0.8393	0.9724	-1.8831
C	3.5907	2.3313	-2.1526
C	1.6770	3.2112	0.1258
C	1.4048	4.4244	-0.7856
C	0.3765	2.8493	0.8672
C	2.7633	3.5819	1.1524
H	0.1511	-2.4300	-0.4569
H	-4.7181	-0.3581	-2.9327
H	-1.3894	0.7662	2.7075
H	2.0267	0.3899	2.4147
H	1.4536	-1.1486	3.1260
H	1.9162	-1.0415	-0.5280
H	4.0487	-0.5606	-1.7345

H	-1.3781	1.8686	3.9562
H	0.0378	2.1837	2.9355
H	0.0221	0.7924	4.0159
H	-2.6606	-0.4736	3.5461
H	-1.0908	-1.2863	3.4684
H	-2.2190	-1.4201	2.1156
H	7.1168	-1.5900	2.3915
H	5.4865	-1.0475	2.8358
H	6.5193	-0.0221	1.8301
H	7.0376	-0.7737	-0.6385
H	6.3340	-2.2823	-1.2373
H	7.6154	-2.3232	-0.0102
H	4.2734	4.2550	-0.7663
H	5.3812	2.8853	-0.7816
H	4.3667	3.1810	0.6411
H	2.5491	1.7924	-3.0518
H	4.3125	1.8851	-2.9210
H	3.3507	3.3741	-2.9953
H	1.2653	-3.7289	0.9467
H	-0.4581	-3.3666	0.9637
H	0.6564	-2.3889	1.9326
H	1.4229	-3.4243	-1.6473
H	0.6455	-2.0075	-2.3839
H	-0.3438	-3.2607	-1.6096
H	-3.5492	3.1168	-0.3779
H	-2.5826	4.5113	0.1131
H	-3.5918	3.6739	1.3002
H	-5.5314	1.9860	-0.1662
H	-6.2604	0.7171	-1.1519
H	-4.8169	1.5605	-1.7303
H	-6.1767	-0.9878	1.3371
H	-5.2161	0.1403	2.2950
H	-4.6605	-1.5234	2.0806
H	-3.7084	-3.1146	0.4283
H	-2.1967	-2.3348	-0.0610
H	-2.9416	-3.5565	-1.1026
H	-5.1077	-3.0567	-2.3113
H	-5.8626	-1.4622	-2.2178
H	-5.9025	-2.5460	-0.8175
H	-2.9182	-1.9750	-3.0576
H	-2.1543	-0.7161	-2.0733
H	-3.6527	-0.3713	-2.9486
H	6.1774	-1.4598	-0.9489
H	5.4426	-2.0553	0.5414
H	5.5484	-0.3101	0.2431
H	2.7283	-2.6562	-2.0661
H	3.7772	-3.4923	-0.9152
H	4.4565	-2.8304	-2.3991
H	-5.3712	2.0175	-3.2330
H	-3.6252	1.8843	-2.9422
H	-4.7013	2.4251	-1.6464
H	-6.3575	0.6909	-0.5631
H	-6.3491	-0.9784	-1.1484
H	-6.9786	0.3229	-2.1776
H	-2.7205	2.6305	3.4744
H	-4.0370	2.2559	2.3649
H	-2.4793	2.8143	1.7284
H	-4.2502	-0.1806	3.2452
H	-3.0712	0.2953	4.4825
H	-2.7806	-1.1431	3.4836
H	-1.3808	-3.0742	-3.0199
H	0.2979	-3.4481	-2.6372
H	-0.2034	-1.7605	-2.8554
H	-1.4370	-3.9620	0.6713
H	-0.5821	-4.7973	-0.6418
H	-2.2587	-4.2659	-0.8717
H	2.3387	-3.2135	2.4686
H	3.4576	-3.4313	1.1226
H	1.7321	-3.2093	0.8154
H	0.1105	0.5189	-1.2065
H	1.1866	0.2021	-2.5795
H	0.3220	1.7372	-2.4725
H	3.1878	3.0730	-2.8500
H	3.9774	1.4956	-2.7446
H	4.4377	2.7870	-1.6292
H	0.6587	4.1979	-1.5562
H	2.3128	4.7709	-1.2890
H	1.0160	5.2632	-0.1929
H	0.5042	1.9834	1.5199
H	-0.4349	2.6114	0.1714
H	0.0456	3.6949	1.4855
H	2.4394	4.4367	1.7616
H	3.7035	3.8639	0.6650
H	2.9765	2.7479	1.8278

Coordinates of **11-c8** (6.27 kJ/mol).

Atom	Coordinates x / A	y / A	z / A
C	2.2211	0.7265	0.3893
C	2.2505	0.3061	-0.9581
C	3.1385	-0.7046	-1.3249
C	3.9893	-1.3085	-0.3944
C	3.9475	-0.8577	0.9239
C	3.0818	0.1589	1.3466
C	1.3981	1.0130	-2.0037
H	3.1664	-1.0317	-2.3582
C	4.9312	-2.4265	-0.8053
H	4.6091	-1.3127	1.6569
C	3.1756	0.6048	2.8026
C	1.2340	1.7893	0.7806
O	0.0032	1.2552	0.9126
C	-1.0998	2.1003	1.3380
C	-1.9963	2.4803	0.1596
C	-2.3682	1.2291	-0.6657
C	-3.2652	1.5236	-1.8839
C	-3.3373	0.3107	-2.8182
C	-4.6627	2.0017	-1.4770
O	1.4859	2.9655	0.9579
C	5.9442	-1.9543	-1.8612
C	4.1534	-3.6587	-1.2970
C	3.9518	1.9303	2.9218
C	1.8388	0.6715	3.5591
C	0.9863	0.1261	-3.1847
C	2.1178	2.2839	-2.4943
C	-1.3722	3.5951	-0.6932
H	-2.9173	2.8652	0.6165
O	-3.0081	0.2792	0.1905
Si	-2.4756	-1.2518	0.6354
C	-1.5959	-1.2103	2.3017
C	-1.2742	-1.9376	-0.6421
C	-4.0529	-2.3121	0.7984
C	-4.8308	-2.3545	-0.5279
C	-3.6670	-3.7498	1.1978
C	-4.9593	-1.7069	1.8881
H	0.4752	1.3294	-1.5122
H	5.4941	-2.7213	0.0891
H	3.7751	-0.1647	3.3035
H	-1.6452	1.4899	2.0552
H	-0.6962	2.9848	1.8322
H	-1.4351	0.8005	-1.0489
H	-2.7735	2.3264	-2.4455

Coordinates of **11-c9** (7.39 kJ/mol).

Atom	Coordinates x / A	y / A	z / A
C	2.0176	-1.0233	-0.1877
C	2.1645	-0.4209	1.0824
C	3.0679	0.6347	1.2089
C	3.8167	1.1044	0.1253
C	3.6562	0.4799	-1.1104
C	2.7657	-0.5827	-1.2941
C	1.3710	-0.8928	2.2960
H	3.1832	1.1138	2.1746
C	4.7686	2.2765	0.2854
H	4.2386	0.8361	-1.9547
C	2.6341	-1.2198	-2.6723
C	1.0059	-2.1143	-0.3458
O	0.0604	-1.7615	-1.2428
C	-1.1133	-2.5986	-1.3927
C	-2.3368	-1.7335	-1.0896
C	-2.3530	-1.1576	0.3489
C	-2.9566	-2.0361	1.4606
C	-2.2874	-3.4119	1.5840
C	-2.8792	-1.2982	2.8036
O	0.9945	-3.1609	0.2754
C	5.8739	1.9767	1.3110
C	4.0072	3.5620	0.6510
C	1.8417	-0.3012	-3.6176
C	3.9988	-1.5936	-3.2724
C	0.9298	0.2560	3.2139
C	2.1694	-1.9495	3.0791
C	-3.6394	-2.4305	-1.4915
H	-2.2296	-0.8519	-1.7315
O	-3.1031	0.0610	0.3061
Si	-2.4539	1.6075	0.2151
C	-0.7039	1.5802	-0.4827
C	-2.4224	2.3696	1.9370
C	-3.6346	2.5632	-0.9311
C	-3.2092	4.0408	-1.0168
C	-3.5958	1.9368	-2.3375
C	-5.0701	2.4741	-0.3800
H	0.4605	-1.3804	1.9366
H	5.2500	2.4383	-0.6871
H	2.0670	-2.1488	-2.5684
H	-1.1306	-2.9265	-2.4357
H	-1.0127	-3.4694	-0.7486
H	-1.3150	-0.9345	0.6289
H	-4.0165	-2.1813	1.2153

H	-3.8093	-0.5419	-2.3264
H	-2.3368	0.0039	-3.1422
H	-3.9223	0.5464	-3.7130
H	-5.2796	2.1869	-2.3619
H	-4.6244	2.9305	-0.8991
H	-5.1607	1.2456	-0.8639
H	6.5131	-1.0911	-1.5034
H	5.4400	-1.6628	-2.7886
H	6.6500	-2.7556	-2.1031
H	4.8393	-4.4769	-1.5396
H	3.5781	-3.4253	-2.1992
H	3.4521	-4.0117	-0.5350
H	4.0887	2.1972	3.9753
H	3.4124	2.7400	2.4252
H	4.9398	1.8442	2.4595
H	1.2478	-0.2363	3.4114
H	1.2326	1.5286	3.2586
H	2.0335	0.7799	4.6308
H	1.8400	-0.1275	-3.8212
H	0.2629	0.6579	-3.8106
H	0.5247	-0.8056	-2.8463
H	1.4922	2.8297	-3.2087
H	3.0568	2.0217	-2.9931
H	2.3497	2.9507	-1.6598
H	-0.9778	4.3888	-0.0538
H	-2.1064	4.0413	-1.3677
H	-0.5412	3.2263	-1.3016
H	-0.6454	-0.6800	2.2141
H	-1.3826	-2.2287	2.6438
H	-2.1964	-0.7197	3.0742
H	-1.6928	-1.9511	-1.6520
H	-0.9920	-2.9622	-0.3780
H	-0.3557	-1.3422	-0.6568
H	-5.1770	-1.3588	-0.8184
H	-5.7151	-2.9992	-0.4316
H	-4.2219	-2.7541	-1.3467
H	-3.0367	-4.2280	0.4400
H	-4.5683	-4.3671	1.3106
H	-3.1289	-3.7818	2.1509
H	-5.2379	-0.6757	1.6480
H	-4.4707	-1.7039	2.8681
H	-5.8850	-2.2905	1.9842

H	-1.2018	-3.3234	1.6940
H	-2.4820	-4.0528	0.7208
H	-2.6707	-3.9334	2.4667
H	-3.3413	-1.8930	3.5979
H	-3.3845	-0.3327	2.7554
H	-1.8345	-1.1217	3.0873
H	5.4538	1.8238	2.3107
H	6.5801	2.8111	1.3704
H	6.4296	1.0743	1.0393
H	3.5151	3.4630	1.6243
H	3.2365	3.7875	-0.0922
H	4.6924	4.4142	0.7069
H	0.8506	-0.0916	-3.2083
H	1.7202	-0.7702	-4.5998
H	2.3622	0.6519	-3.7602
H	4.5671	-2.2356	-2.5928
H	4.6058	-0.7087	-3.4869
H	3.8587	-2.1317	-4.2154
H	1.7756	0.7096	3.7398
H	0.2416	-0.1219	3.9763
H	0.4191	1.0445	2.6537
H	1.5916	-2.3091	3.9373
H	3.1064	-1.5228	3.4535
H	2.4044	-2.8011	2.4378
H	-3.7914	-3.3710	-0.9542
H	-3.6438	-2.6544	-2.5629
H	-4.4901	-1.7778	-1.2793
H	0.0074	1.1047	0.1975
H	-0.3575	2.6066	-0.6464
H	-0.6428	1.0477	-1.4353
H	-1.8149	1.7629	2.6155
H	-3.4280	2.4375	2.3638
H	-1.9949	3.3778	1.9166
H	-3.2390	4.5307	-0.0375
H	-3.8850	4.5956	-1.6815
H	-2.1952	4.1515	-1.4166
H	-2.5959	1.9949	-2.7814
H	-4.2861	2.4618	-3.0115
H	-3.8923	0.8833	-2.3121
H	-5.7698	2.9973	-1.0458
H	-5.1541	2.9346	0.6105
H	-5.3999	1.4339	-0.2957

Coordinates of **11-c10** (7.43 kJ/mol).

	Coordinates		
Atom	x / A	y / A	z / A
C	-1.6481	-1.1835	0.3827
C	-2.1727	-0.4772	1.4823
C	-3.2764	0.3506	1.2654
C	-3.8509	0.4987	0.0014
C	-3.3237	-0.2387	-1.0620
C	-2.2309	-1.0927	-0.8953
C	-1.5943	-0.6791	2.8780
H	-3.6946	0.9114	2.0942
C	-5.0070	1.4601	-0.2119
H	-3.7696	-0.1404	-2.0454
C	-1.7199	-1.9422	-2.0521
C	-0.4265	-2.0349	0.5729
O	0.6646	-1.2579	0.7137
C	1.9323	-1.8849	1.0367
C	2.8966	-1.8954	-0.1515
C	3.5471	-0.5126	-0.4401
C	4.9750	-0.3438	0.1169
C	5.0405	-0.4824	1.6410
C	5.9713	-1.2761	-0.5814
O	-0.4124	-3.2522	0.6008
C	-6.2457	0.7568	-0.7886
C	-4.5773	2.6423	-1.0977
C	-2.4317	-3.3082	-2.0475
C	-1.8474	-1.2650	-3.4226
C	-2.1100	-2.0070	3.4694
C	-1.8670	0.4791	3.8442
C	2.2388	-2.4830	-1.4052
H	3.6948	-2.5845	0.1510
O	2.7353	0.5391	0.0806
Si	2.0482	1.7399	-0.8690
C	0.8175	1.0015	-2.0855
C	3.4126	2.6309	-1.8193
C	1.1784	2.9069	0.3514
C	-0.0607	2.2102	0.9410
C	2.1444	3.2762	1.4919
C	0.7394	4.1870	-0.3851
H	-0.5073	-0.7610	2.7730
H	-5.2793	1.8623	0.7720
H	-0.6551	-2.1319	-1.8844
H	2.3297	-1.3005	1.8653
H	1.7310	-2.9065	1.3618
H	3.6268	-0.4132	-1.5332
H	5.2514	0.6887	-0.1346

H	4.8298	-1.5090	1.9617
H	4.3207	0.1842	2.1231
H	6.0402	-0.2275	2.0067
H	6.9938	-1.0548	-0.2599
H	5.9294	-1.1632	-1.6703
H	5.7775	-2.3285	-0.3466
H	-6.0444	0.3546	-1.7870
H	-7.0811	1.4591	-0.8760
H	-6.5605	-0.0740	-0.1500
H	-5.3927	3.3655	-1.2029
H	-4.3010	2.2983	-2.1001
H	-3.7117	3.1581	-0.6707
H	-2.0531	-3.9416	-2.8569
H	-3.5096	-3.1768	-2.1928
H	-2.2688	-3.8227	-1.0982
H	-1.3940	-0.2704	-3.4235
H	-2.8933	-1.1652	-3.7302
H	-1.3444	-1.8712	-4.1823
H	-1.8683	-2.8524	2.8207
H	-3.1981	-1.9712	3.5897
H	-1.6640	-2.1886	4.4529
H	-1.3198	0.3171	4.7778
H	-2.9292	0.5518	4.0999
H	-1.5494	1.4373	3.4246
H	1.7524	-3.4383	-1.1899
H	2.9845	-2.6430	-2.1896
H	1.4822	-1.7999	-1.7990
H	1.3100	0.3452	-2.8101
H	0.3042	1.7882	-2.6491
H	0.0642	0.4183	-1.5511
H	4.1002	3.1469	-1.1415
H	2.9944	3.3734	-2.5069
H	4.0009	1.9256	-2.4162
H	-0.8070	1.9793	0.1751
H	-0.5441	2.8598	1.6838
H	0.2031	1.2697	1.4300
H	1.6575	3.9625	2.1981
H	3.0460	3.7747	1.1181
H	2.4591	2.3874	2.0466
H	1.5935	4.7411	-0.7889
H	0.2077	4.8576	0.3033
H	0.0583	3.9670	-1.2151
